# Strain-Controlled Synthesis of [*n*]Catenanes from Dipyrromethane-Stoppered Rotaxanes

**DOI:** 10.1021/acs.orglett.5c01442

**Published:** 2025-06-05

**Authors:** Rafał A. Grzelczak, Jędrzej P. Perdek, Miłosz Siczek, Piotr J. Chmielewski, Bartosz Szyszko

**Affiliations:** Faculty of Chemistry, 49572 University of Wrocław, 14 F. Joliot-Curie St., 50-383 Wrocław, Poland

## Abstract

The synthesis of rotaxanes featuring two dipyrromethane stoppers, which differ in thread length and rigidity, is presented. The condensation of the precursor rotaxanes with acetone was found to be influenced by the axle’s structural facets. As a result, the calix[4]­phyrin-based macrocyclization strategy produced [2]-, [3]-, and [4]­catenanes, depending on the specific structure of the rotaxane substrate.

## Graphical abstract

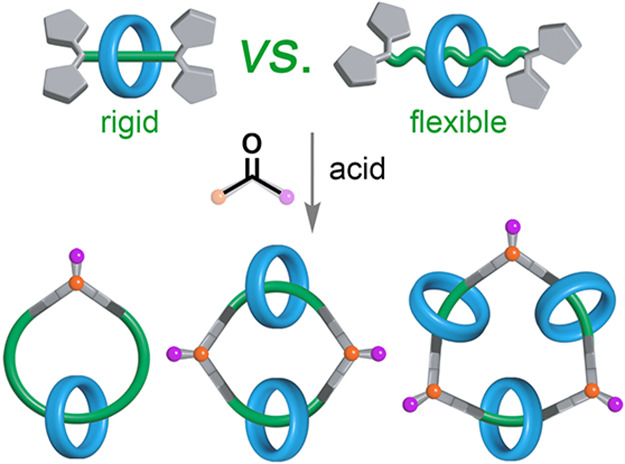

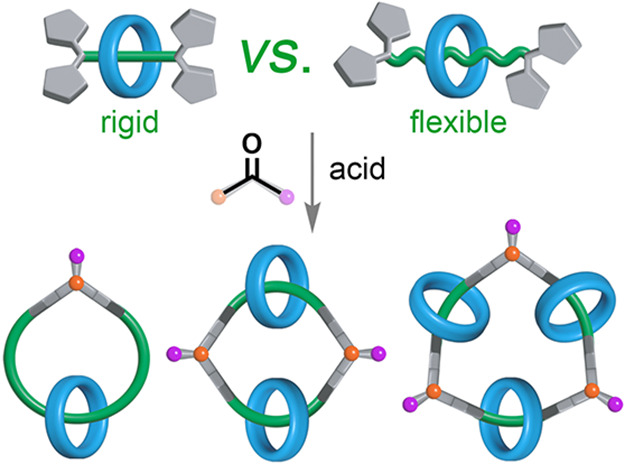

Mechanically Interlocked Molecules (MIMs) are a group of compounds characterized by the presence of a mechanical bond, with rotaxanes and catenanes being the most recognized examples.^1–3^ Among various applications in biomedicine,^4^ catalysis,^5^ stimuli-responsive polymers,^6^ and ion recognition,^7^ those systems were intensively examined as elements of molecular machines.^8^

Several methods were developed to synthesize catenanes, enabling the controlled formation of links of proper size and operations.^9^ Following the statistical synthesis,^10^ protocols based on the covalent,^11^ and anion templation were described,^12^ as well as metal-based passive^7,13^ and active template approaches.^14,15^ Methods that exploit intermolecular interactions,^16,17^ the hydrophobic effect,^18^ or employ self-assembly have enabled the formation of intricate molecular links.^19–23^ While multiple synthetic strategies were developed for [2]­catenanes, the formation of higher-order links, namely [*n*]­catenanes (*n* ≥ 3), still poses a significant challenge.^24–26^

Porphyrin-incorporating catenanes received considerable interest due to their energy and electron-transfer properties^27^ and versatile coordination chemistry.^28^ While their syntheses were developed, the access to interlocked architectures embedding other porphyrinoids is limited.^24,29–31^ Our group demonstrated the synthetic scenario encompassing [2]­rotaxanes incorporating dipyrromethane stoppers, which yielded calix[4]­phyrin-incorporating MIMs.^32^ Unlike the planar porphyrin, the V-shaped porphyrinoid^33,34^ in the axle undergoes a toggling motion, activating the conformational reconfigurations termed *fluttering*.

Here, we demonstrate that the macrocyclization of dipyrromethane-stoppered [2]­rotaxanes, varying in axle length and rigidity, can yield higher-order catenanes. The intrinsic curvature of calix[4]­phyrin facilitates product formation, with the reaction outcome being governed by the strain introduced during macrocyclization. Despite the well-established methods for catenane synthesis, reactions that transform [2]­rotaxanes into higher-order MIMs remain relatively scarce, highlighting the significance of the proposed approach.^35^

The synthesis of catenanes incorporating a porphyrinoid within one of the interlocked macrocycles requires suitable building blocks. The [2]­rotaxanes possessing two dipyrromethane stoppers were identified as synthons for the construction of [*n*]­catenanes embedding calix[4]­phyrin moiety.^32^ Employing [2]­rotaxanes with threads varying in length and rigidity was sought to help overcome the strain building within the system upon introducing consecutive components.

The synthesis of [2]­rotaxanes **4**–**7** was achieved using the CuAAC active template approach. The reaction between **2** and **3**, incorporating azide and alkyne groups, and **1** ^36^ was carried out with tetrakis­(acetonitrile)­copper­(I) hexafluorophosphate in the presence of *N*,*N*-diisopropylethylamine (DIPEA), providing rotaxane **4** in a 45% yield (Scheme 1).^26^ The compounds **5**–**7**, differing in length and rigidity of the linker connecting dipyrromethane termini, were obtained in 43–72% yield. The identity of **4**–**7** was confirmed by a combination of mass spectrometry (Figures S7, S15, S23, Supporting Information (SI)), and NMR spectroscopy (Figures S1, S9, S17, SI). The ^1^H NMR spectra of **4**–**7** revealed two distinct series of resonances corresponding to the macrocyclic and thread components of the rotaxane. Two broad NH resonances were observed in the 7.80–8.00 ppm range. The α- and β-pyrrolic protons resonated between 5.83 and 6.72 ppm, accompanied by two *meso*-CH singlets at 5.35 ppm. The triazole resonances appeared in the 8.02–9.35 ppm region. A downfield (0.4–1.8 ppm) shift of these signals, compared to the noninterlocked axle, suggested the CH···N hydrogen bonding (HB) with the bipyridine of **1**.^37^

**Scheme 1:**
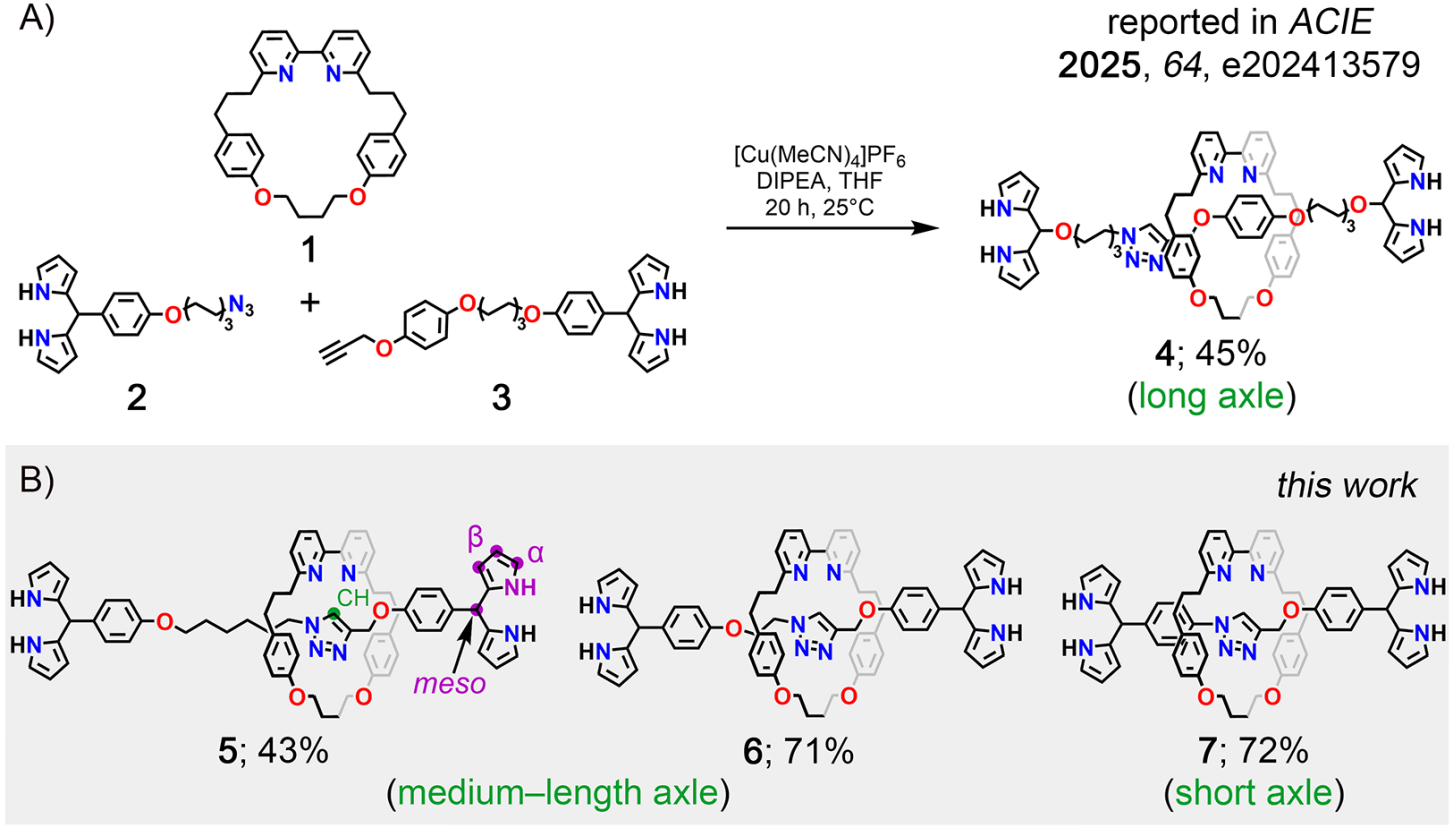
A) Synthesis of **4** and B) [2]­Rotaxanes **5**–**7**

With precursors **4–7** in hand, the synthesis of molecular links could be evaluated. It was anticipated that the structure of the building block would influence the type and ratio of products formed. Specifically, [2]­rotaxanes with longer and more flexible axles were expected to react with acetone, yielding predominantly lower-order catenanes, resulting from the self-condensation of the opposite-side dipyrromethanes or dimerization of the substrate (Figure 1A). In contrast, [2]­rotaxanes with short and rigid threads were expected to produce higher-order links because the strain incorporated into the newly formed macrocycle can be minimized only upon oligomerization (Figure 1B).^37^

**Figure 1:**
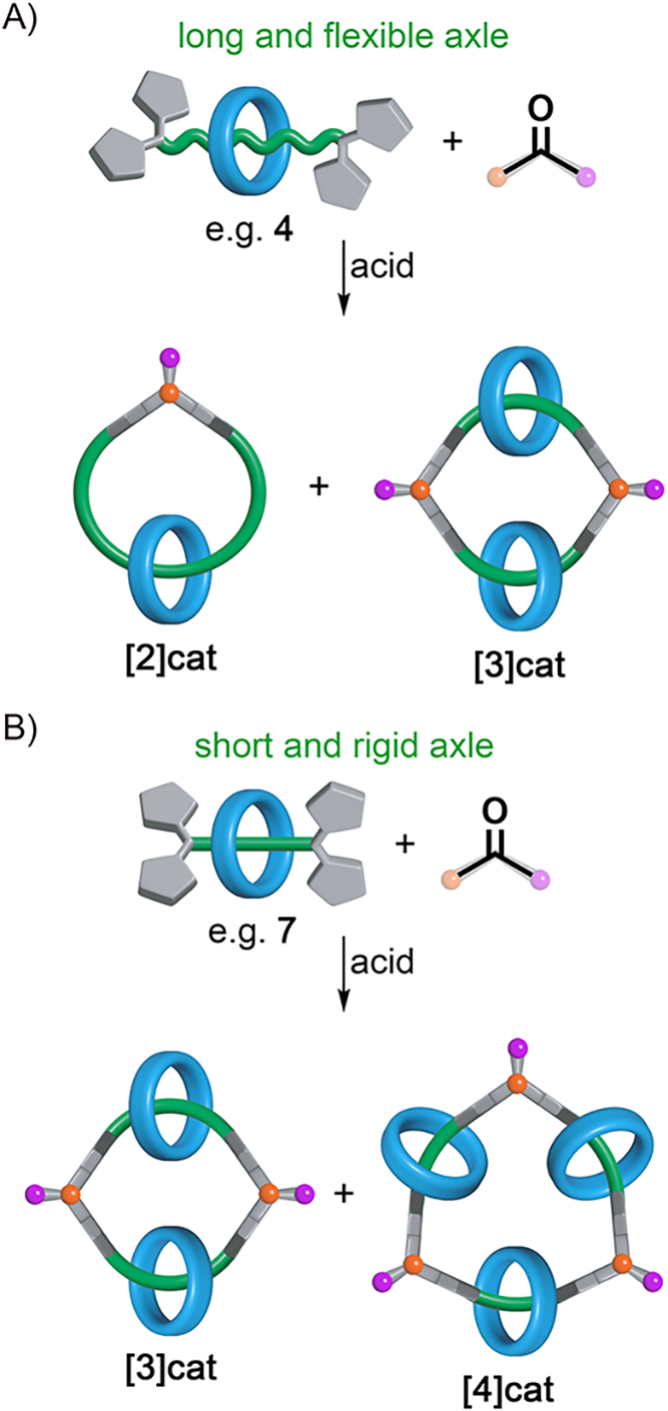
Strain-dependent formation of [*n*]­catenanes incorporating A) flexible and B) rigid threads. The structural formulas of all [*n*]­catenanes were shown in Scheme S7.

The condensation of **4** with acetone at 1.25 × 10^–3^ M catalyzed by boron trifluoride diethyl etherate (BF_3_·Et_2_O), followed by the oxidation with 2,3-dichloro-5,6-dicyanobenzoquinone (DDQ), yielded [2]­catenane **[2]­cat** ^**4**^ in 31%.^32^ Hence, various reaction conditions were investigated to assess the potential formation of larger links, including the catalyst amount, reaction time, and reagent concentration. The latter was identified as a key factor influencing the composition of product mixtures. Consistently, the reaction was carried out at a higher concentration (2.87 × 10^–3^ M). Under such conditions, aside from **[2]­cat** ^**4**^ (26%), the additional product, i.e., [3]­catenane **[3]­cat** ^**4**^ (2.6%), was isolated by column chromatography. The **[3]­cat** ^**4**^ formation occurred through condensing two molecules of **4** with four equivalents of acetone. The elemental composition of **[3]­cat** ^**4**^ was confirmed through ESI-MS (Figure S34, SI).

The ^1^H NMR spectrum of **[3]­cat** ^**4**^ revealed two distinct series of resonances with very close chemical shifts (Figure S25, SI). The two singlets of *meso*-protons in **4** were absent in the spectrum of **[3]­cat** ^**4**^, confirming the formation of the new product(s). The formation of calix[4]­phyrins in **[3]­cat** ^**4**^ led to a downfield relocation of the NH resonances from 7.91 ppm in **4** to 14.19 ppm, suggesting the HB between the intracavity NH protons. The corresponding β-pyrrolic resonances were found in the 6.37–6.18 ppm range. The overlapping triazole signals arose at ca. 7.99 ppm, indicating the CH···N HB with the macrocyclic bipyridine.^37,32^ The observation of two sets of signals in the spectrum suggested inhomogeneity of the **[3]­cat** ^**4**^ fraction. A detailed analysis revealed the presence of two isomers resulting from the dissymmetry of the thread in the precursor rotaxane, which stems from the two sides of the axle being linked to the central 1,2,3-triazole *via* either C–C or C–N bonds (Figure 2A). Consequently, the macrocyclization of **4** proceeded through two pathways, leading to the head-to-tail (HT, **[3]­cat**_**HT**_^**4**^) and head-to-head (HH, **[3]­cat**_**HH**_^**4**^) isomers of [3]­catenane, which could not be separated (Figure 2B).

**Figure 2:**
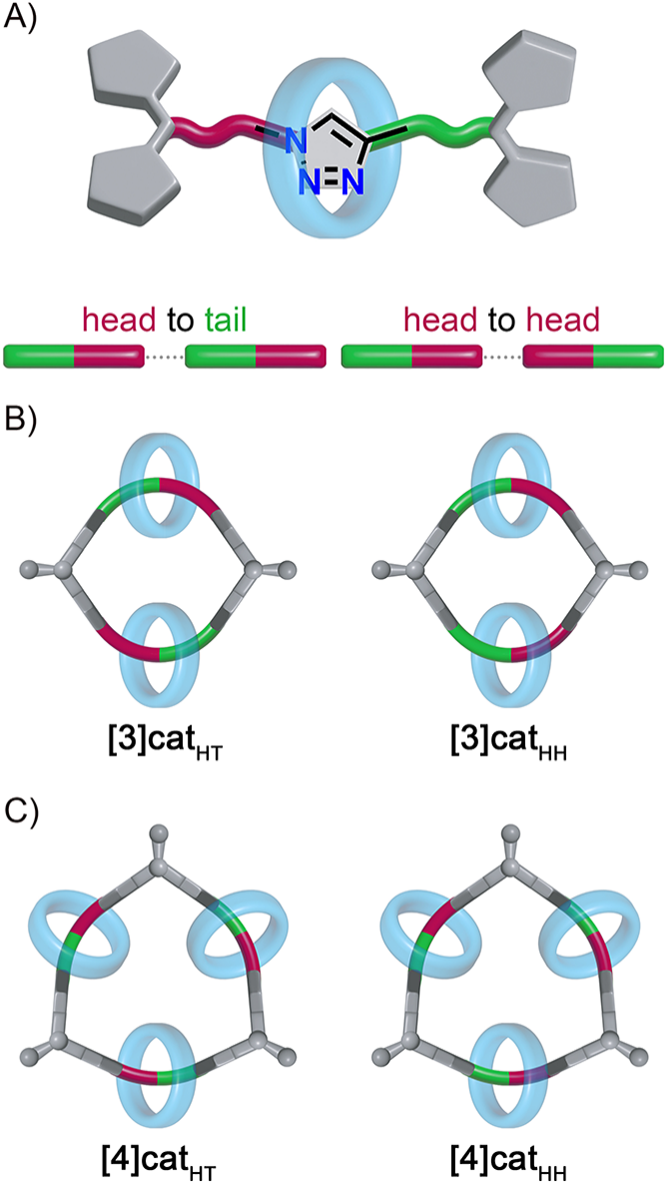
A) Dissymmetry in **4**–**7** resulting in the formation of isomers of B) [3]­catenanes and C) [4]­catenanes.

Once the reaction conditions utilizing **4** were developed, the focus shifted toward the formation of calix[4]­phyrin-incorporating [*n*]­catenanes from **5**–**7**, which differed in length and rigidity of the thread (Figure 1). First, **5** and **6** were reacted with acetone in the presence of BF_3_·Et_2_O catalyst. The analysis of products confirmed the formation of [3]­catenanes in both cases. Consequently, the mixtures of **[3]­cat**_**HT**_^**5**^/**[3]­cat**_**HH**_^**5**^ and **[3]­cat**_**HT**_^**6**^/**[3]­cat**_**HH**_^**6**^ were isolated in 12 and 5% yield, respectively. Interestingly, the reaction yield seemed dependent on the substrate structure, with **5** containing the longer and more flexible thread producing more of the [3]­catenane.

The elemental composition of **[3]­cat**_**HT**_^**5**^/**[3]­cat**_**HH**_^**5**^ was confirmed by ESI-MS (Figure S47, SI). Furthermore, the ^1^H NMR spectroscopy corroborated the transformation of bis-dipyrromethane into the macrocyclic species through the disappearance of α- and *meso*-H resonances of **5**, as well as a downfield relocation of the NH signals (Figure 3A). The mixture of **[3]­cat**_**HT**_^**5**^ and **[3]­cat**_**HH**_^**5**^ was separated by HPLC (Figure S49–50, SI) allowing to record the spectra of individual isomers (>85% purity). The major difference between the two species included the chemical shift of the NH resonances at 14.19/14.18 and 14.24/14.14 ppm, respectively. The remaining signals were found at nearly identical positions with the β-pyrrolic lines at the 6.34–6.13 ppm, and triazole ones at 8.33 and 8.32 ppm.

**Figure 3:**
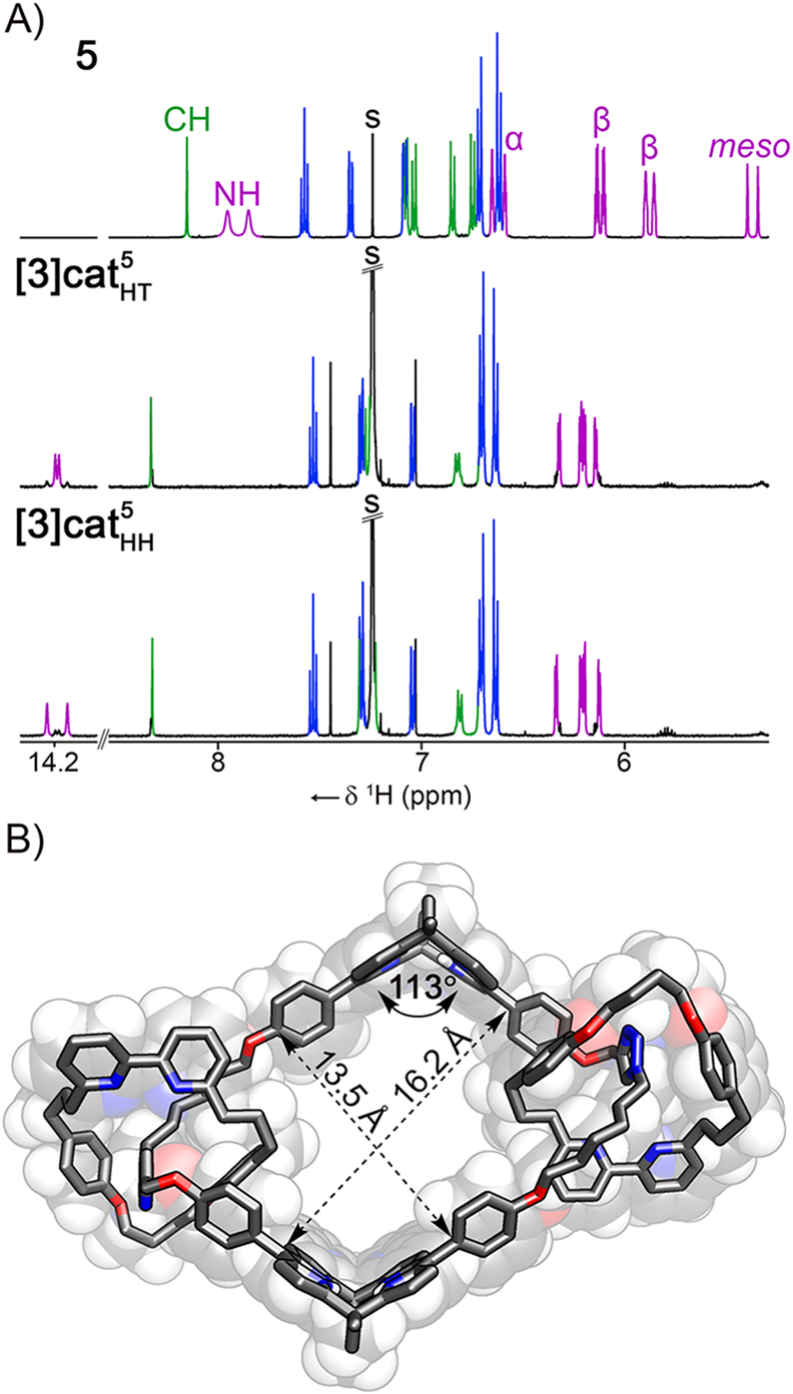
A) The ^1^H NMR spectrum (CDCl_3_, 300 K, 500 MHz) of **5**, **[3]­cat**_**HT**_^**5**^, and **[3]­cat**_**HH**_^**5**^ with signals of the bpy-macrocycle in blue, thread in green, and pyrrole in magenta. Resonance assignments follow the numbering system given in Scheme 1, corresponding to the rotaxanes and catenanes derived therefrom. B) The X-ray molecular structure of **[3]­cat**_**HT**_^**5**^.

The X-ray structure confirmed the identity of **[3]­cat**_**HT**_^**5**^, featuring a macrocycle incorporating two calix[4]­phyrins, with macrocyclic bipyridines positioned at the linkers separating the *meso*-aryl substituents of porphyrinoids (Figure 3B). The large ring (16.2 Å × 13.5 Å) adopted a parallelogram conformation, with the two V-shaped calix[4]­phyrins located at opposite vertices. The dihedral angle between dipyrrin planes equaled 113°. The macrocyclic bipyridines were situated near the triazole moieties, stabilizing the coconformation through CH···N HB (2.513, 2.730 Å).

The condensation of **6** with acetone also yielded a mixture of HT and HH isomers, whose identities were confirmed by a combination of ESI-MS (Figure S62, SI) and NMR (Figures S51–61, SI). The head-to-tail [3]­catenane was crystallized as a hexacation **[3]­cat**_**HT**_^**6**^ **·6HBF**_**4**_, featuring two monoprotonated bipyridyls and two diprotonated calix[4]­phyrins engaged in the NH···FBF_3_^–^ interactions (1.926–2.142 Å) with tetrafluoroborates on either side of the porphyrinoid (Figure 4A). Two additional tetrafluoroborates were identified close to the protonated bipyridines. The latter formed HB with triazoles, exhibiting a (bpy)­NH···N­(triazole) distance of 2.109 Å. The protonation of calix[4]­phyrin triggered a conformational change of the V-shaped neutral macrocycle to a 1,3-alternated dication, with two pairs of oppositely oriented pyrroles (Figure 4B). This alteration of the porphyrinoid influenced the geometry of the large macrocyclic component, the cavity of which acquired a roughly rectangular shape with approximate dimensions of 19 Å in length and 8–10 Å in width. Two small monoprotonated rings were positioned at the shorter sides, orienting their bipyridine moieties toward the center of the large ring.

**Figure 4:**
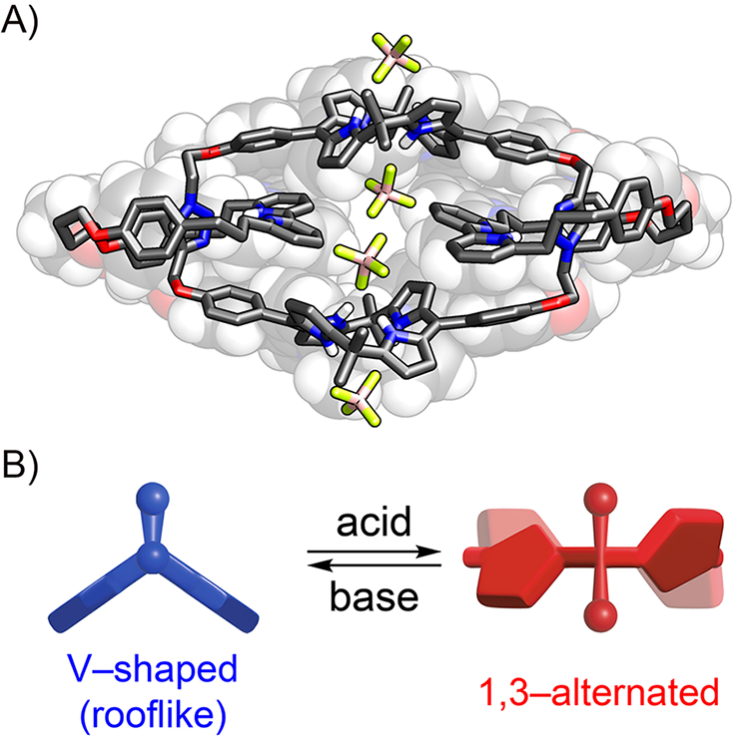
A) X-ray molecular structure of **[3]­cat**_**HT**_^**6**^ **·6HBF**_**4**_. Two noncoordinating anions were omitted for clarity. B) Conformational transformation of calix[4]­phyrin upon protonation.

When **7**—featuring the shortest and most rigid thread—was subjected to catenane-forming conditions, its reactivity proved low. Despite employing a previously optimized synthetic protocol, **7** formed only traces of mechanically interlocked products. Careful chromatographic separation afforded [3]­catenane **[3]­cat** ^**7**^ and radial [4]­catenane **[4]­cat** ^**7**^ in 0.6% and 0.6% yield, respectively. [5]­Catenane was detected only through ESI-MS (Figure S90, SI). This suggested that the rigidity of the thread in **7** redirected the reaction toward higher-order links to minimize the strain incorporated in the MIM upon macrocyclization. While the mixture of **[3]­cat**_**HT**_^**7**^ and **[3]­cat**_**HH**_^**7**^ was successfully separated by thin-layer chromatography, isolation of **[4]­cat** ^**7**^ isomers proved challenging.

The elemental compositions of both **[3]­cat** ^**7**^ and **[4]­cat** ^**7**^ were confirmed by ESI-MS (Figures S77, S87 SI). The formation of catenated structures was further supported by the NMR spectroscopy. The ^1^H NMR spectra of **[3]­cat**_**HT**_^**7**^ and **[3]­cat**_**HH**_^**7**^ (Figures S64, S70, SI) revealed substantial differences in the chemical shifts of several protons, reflecting their more compact and rigid structures, relative to those observed for **[3]­cat** ^**4–6**^. The calixphyrin NH protons of **[3]­cat**_**HT**_^**7**^ and **[3]­cat**_**HH**_^**7**^ appeared as two sharp signals at 14.67/14.46 ppm and 14.42/14.27 ppm, respectively. The triazole resonance in **[3]­cat**_**HT**_^**7**^ at 8.88 ppm was upfield-shifted compared to **[3]­cat**_**HH**_^**7**^ (9.66 ppm). The β-pyrrolic resonances in the latter produced four lines in the 6.23–6.07 ppm region, whereas those of **[3]­cat**_**HT**_^**7**^ resonated at 6.23–5.78 ppm.

The X-ray structures of isomers differed significantly in terms of geometry (Figures S79–80, SI). The large, calixphyrin-embedded ring in **[3]­cat**_**HT**_^**7**^ adopted a rectangular shape (10 × 13.7 Å), while the cavity of **[3]­cat**_**HH**_^**7**^ was more square. The macrocyclic bipyridines, in both isomers, were positioned close to triazoles, along the linkers connecting the calixphyrin *meso*-positions. Remarkably, despite the more compact dimensions of the calix[4]­phyrin-embedded macrocycles in **[3]­cat**_**HT**_^**7**^/**[3]­cat**_**HH**_^**7**^ than **[3]­cat** ^**4**^, the dihedral angle between the mean planes of the dipyrrin units exhibited only minimal variation, ranging from 109° to 112°.

Interestingly, the isolation of [4]­catenane **[4]­cat** ^**7**^ from a product mixture suggested that, for [2]­rotaxane featuring short and rigid threads, the generation of higher-order structures may serve as a means of strain relief. The separation of the radial [4]­catenane isomers proved challenging, and they were analyzed in a mixture by ^1^H NMR spectroscopy (Figure S81–86, SI). Given that the isomers differed in effective symmetry (*C*_3_ vs *C*_1_), significant signal overlap led to spectra dominated by poorly defined multiplets. Nonetheless, key signals could be assigned in the expected regions: NH resonances at 14.18–14.08 ppm, triazole CHs at 9.81–9.77 ppm, and β-pyrrolic in the 6.25–6.06 ppm range.

A facile and high-yielding synthesis of [2]­rotaxanes featuring dipyrromethane stoppers has been developed, providing key precursors for interlocked porphyrinoids. Subsequent macrocyclization yielded a series of [*n*]­catenanes, with the central ring component incorporating calix[4]­phyrin as a curvature-inducing scaffold. The outcome of the reaction was governed by the length and rigidity of the rotaxane’s axle: long and flexible threads favored the formation of [2]- and [3]­catenanes, whereas short and rigid axles promoted [3]- and [4]­catenanes. The inherent conformational flexibility of calix[4]­phyrin played a crucial role in accommodating strain within the interlocked ring system.

Our methodology offers a direct route to relatively complex mechanically interlocked porphyrinoids, providing a valuable platform for accessing sophisticated nanometer-scale MIMs. It is expected that the developed strategy will enable the formation of novel MIMs benefiting from the distinctive electronic, stereochemical, and redox properties of various porphyrinoids, leading to intriguing supramolecular materials and molecular electronic devices.

## Supplementary Material



## Data Availability

The data underlying this study are available in the published article and its Supporting Information.

## Notes

Experimental procedures, NMR, MS spectra, and HPLC chromatograms (PDF)
