# Supplementary material for: Strain-Controlled Synthesis of [n]Catenanes from Dipyrromethane-Stoppered Rotaxanes
Source: Org Lett. 2025 Jun 5;27(24):6310–5. doi: 10.1021/acs.orglett.5c01442 (PMC12186669; doi:10.1021/acs.orglett.5c01442)
Supplement: Supplementary file 1 [file ol5c01442_si_001.pdf]

Supporting Information

Strain-Controlled Synthesis of [*n*]Catenanes from Dipyrromethane-Stoppered Rotaxanes

Rafał A. Grzelczak, Jędrzej P. Perdek, Miłosz Siczek, Piotr J. Chmielewski, and Bartosz Szyszko*

Abstract: The synthesis of rotaxanes featuring two dipyrromethane stoppers, which differ in thread length and rigidity, is presented. The condensation of the precursor rotaxanes with acetone was found to be influenced by the axle's structural facets. As a result, the calix[4]phyrin-based macrocyclization strategy produced [2]-, [3]-, and [4]catenanes, depending on the specific structure of the rotaxane substrate.

Experimental procedures....3
Instrumentation....3
Synthesis....5
Macrocycle synthesis....5
Half axle synthesis....6
Compound **S9**....11
Compound **S10**....11
Compound **S16**....12
Dipyrromethane stoppered rotaxane synthesis....13
[2]Rotaxane **5**....13
[2]Rotaxane **6**....22
[2]Rotaxane **7**....31
Catenanes **[2]cat^{4}** and **[3]cat^{4}**....40
Catenane **[3]cat^{5}**....53
Catenane **[3]cat^{6}**....69
Catenanes **[3]cat^{7}** and **[4]cat^{7}**....83
Catenane **[3]cat$^{7}_{HT}$**....84
Catenane **[3]cat$^{7}_{HH}$**....92
References....113

Experimental procedures

Instrumentation

NMR spectroscopy

^{1}H and 13C NMR spectra were recorded on a high-field Bruker spectrometers (^{1}H 600.15 MHz and 500.13 MHz), equipped with a broadband inverse gradient probe head, and a high-field-JEOL (^{1}H 500 MHz) spectrometer, equipped with a 5 mm wide broadband probe. Spectra were referenced to the residual solvent signal (chloroform-*d* – 7.24 ppm, dichloromethane-d_2 – 5.32 ppm). Structural assignments were made with additional information from gCOSY, gHSQC, and gHMBC experiments.

Mass spectrometry

ESI TOF mass spectra were recorded on Bruker qTOF compact.

X-ray diffraction data

Single-crystal X-ray diffraction data for all crystals were collected at 100 K using Cu Kα radiation on a Rigaku XtaLAB Synergy R DW system equipped with a HyPix-Arc 150 hybrid detector and an Oxford Cryosystems 800 temperature unit. Data reduction was carried out using the CrysAlis Pro program.[1] The crystal structures were solved with ShelxT[2] and refined with ShelXL[3] programs. All crystal structures include some disorder.

[3]cat$^{5}_{HT}$: monocrystals were grown by slow evaporation from **[3]cat^{5}** solution in a mixture of 1,2-dichloroethane and diisopropyl ether. Some 1,2-dichloroethane molecules were restrained with C–C, C–Cl, and 1,3-C–Cl distances of 1.490(2), 1.770(2), and 2.720(4) Å, respectively. One of the 1,2-dichloroethane molecules was disordered over four sites. The structure was deposited in CCDC with deposition number **#2441086**.

[3]cat$^{6}_{HT}$·6HBF$_4$: monocrystals were grown by slow evaporation from **[3]cat^{6}** solution in a mixture of dichloromethane and ethyl acetate with a drop of HBF_4 (50% in H_2O). Disorder was modelled on the bipyridine macrocycle. One of the dichloromethane molecules was disordered over two positions. One of the tetrafluoroborate anions was disordered over two positions. The structure was deposited in CCDC with deposition number **#2441087**.

[3]cat$^{7}_{HT}$: monocrystals were grown by slow evaporation from **[3]cat$^{7}_{HT}$** solution in a mixture of chloroform and acetonitrile. One of the chloroform molecules was disordered over three positions and thus the C–Cl distances were restrained to 1.770(2) Å. Another chloroform molecule was disordered over an inversion center and was modelled by restraining C–Cl distances to 1.77(2) and 1.770(5) Å, adequately. Additionally, its 1,3-Cl–Cl distances were

restrained to 2.92(4) and 2.92(2) Å, adequately. The structure was deposited in CCDC with deposition number **#2441088**.

[3]cat$^{7}_{HH}$: monocrystals were grown by slow evaporation from **[3]cat$^{7}_{HH}$** solution in a mixture of chloroform and acetonitrile. The triazole-containing arms connecting the calix[4]phyrin moieties were disordered over two positions (Figure S80). Atoms C1 to C15 along with C1A to C15A atoms (occupancy 0.5) constitute the first part of the molecule, in which the triazole rings are oriented in a head-to-head fashion. Consequently, the remaining atoms C1(-X, 2-Y, 2-Z) to C15(-X, 2-Y, 2-Z) and C1A(-X, 2-Y, 2-Z) to C15A(-X, 2-Y, 2-Z) make up the second part of the molecule (occupancy 0.5), in which the triazole rings are also oriented in a head-to-head fashion. Furthermore, the disorder in the bipyridine macrocycle was modelled. The C30N–C31M and C31N–C32N distances were fixed to 1.540(5) Å. The chloroform molecule was disordered over three positions. Hence, the C–Cl and 1,3-Cl–Cl distances were restrained to 1.77(2) and 2.92(4) Å, respectively. The acetonitrile molecule was disordered over two sites. The structure was deposited in CCDC with deposition number **#2441089**.

Albeit unequivocal distinction between the species present in the crystal (**[3]cat$^{7}_{HT}$** *vs* **[3]cat$^{7}_{HH}$**) cannot be made based on the crystallographic data alone, there is conclusive evidence pointing at **[3]cat$^{7}_{HH}$**:

(A) the monocrystal was obtained from a sample of pure **[3]cat$^{7}_{HH}$**, as determined by NMR analysis (*cf*. Figures S70);

(B) the geometry of this molecule vastly differs from that of **[3]cat$^{7}_{HT}$** (Figure S79-80).

Detailed information on disorder treatment is included in the CIF file.

HPLC chromatography

HPLC resolutions were performed using the LaChrom Merck Hitachi I-7420 system using the Phenomenex Chirex 3014 column (25 cm x 0.46 cm) with an eluent flow rate of 2 mL/min (hexane/dichloromethane 75/25 v/v).

Synthesis

Macrocycle synthesis

Compounds **S3**,[4] **S4-1**[5] were synthesized as described in the literature.

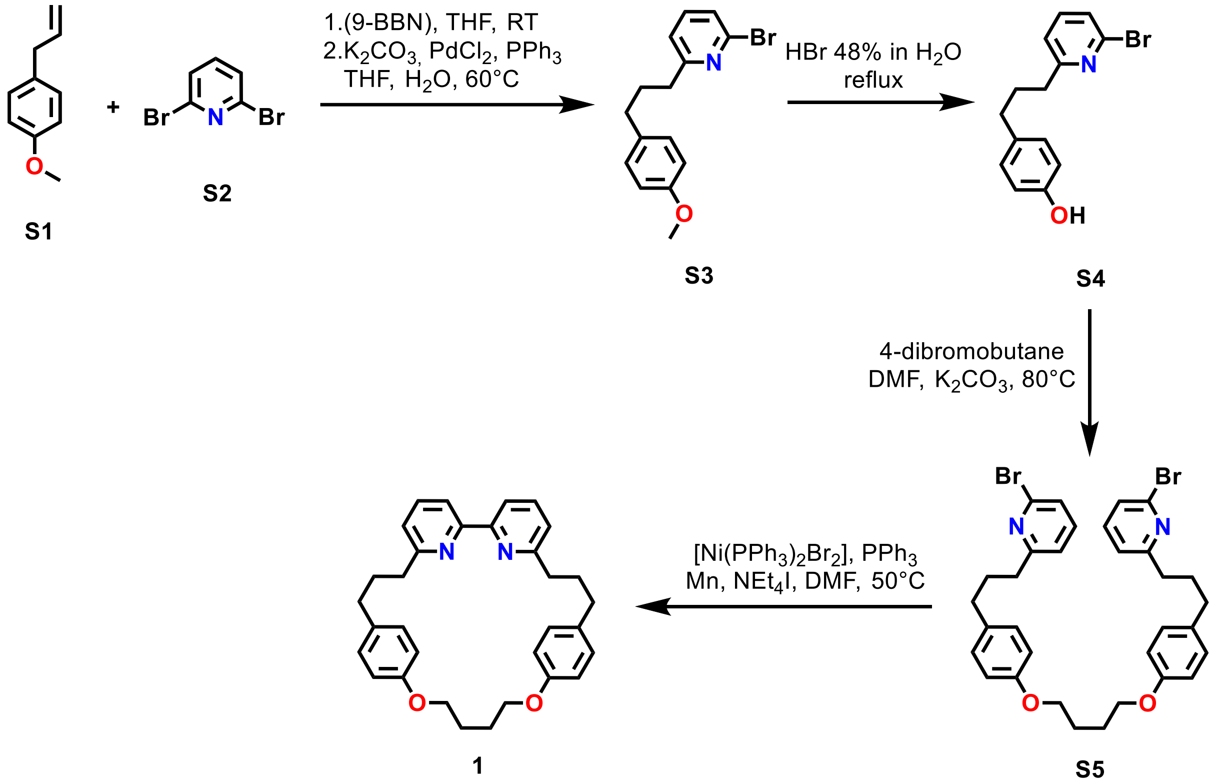

Scheme S1. The synthesis of **1**.[5]

Half axle synthesis

Compounds **S8**,[6] **S13**,[7] **S15**,[8] **S18**,[7] **2**,[7] **3**[7] were synthesized as described in the literature.

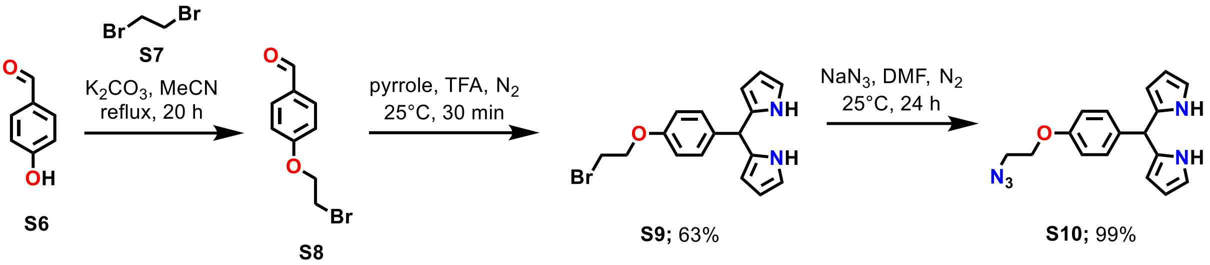

Scheme S2. The synthesis of **S10**.

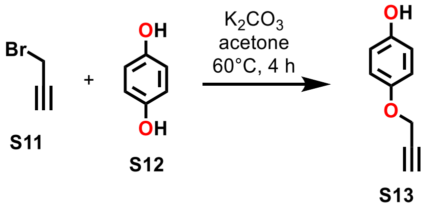

Scheme S3. The synthesis of **S13**.[7]

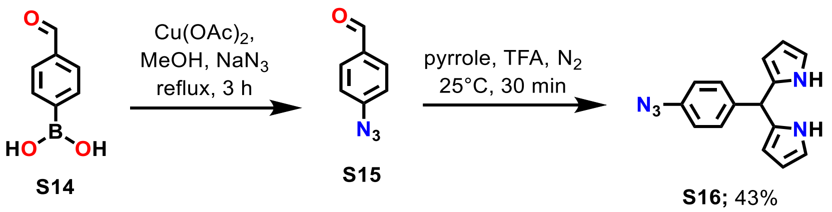

Scheme S4. The synthesis of **S16**.

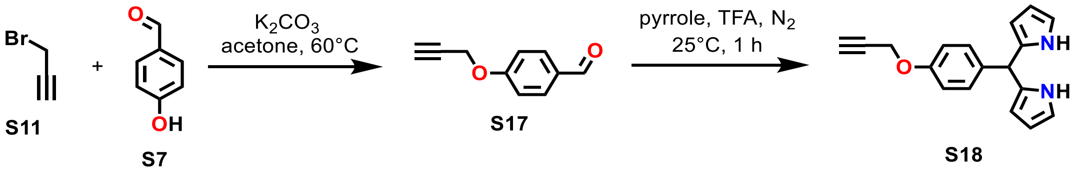

Scheme S5. The synthesis of **S18**.[7]

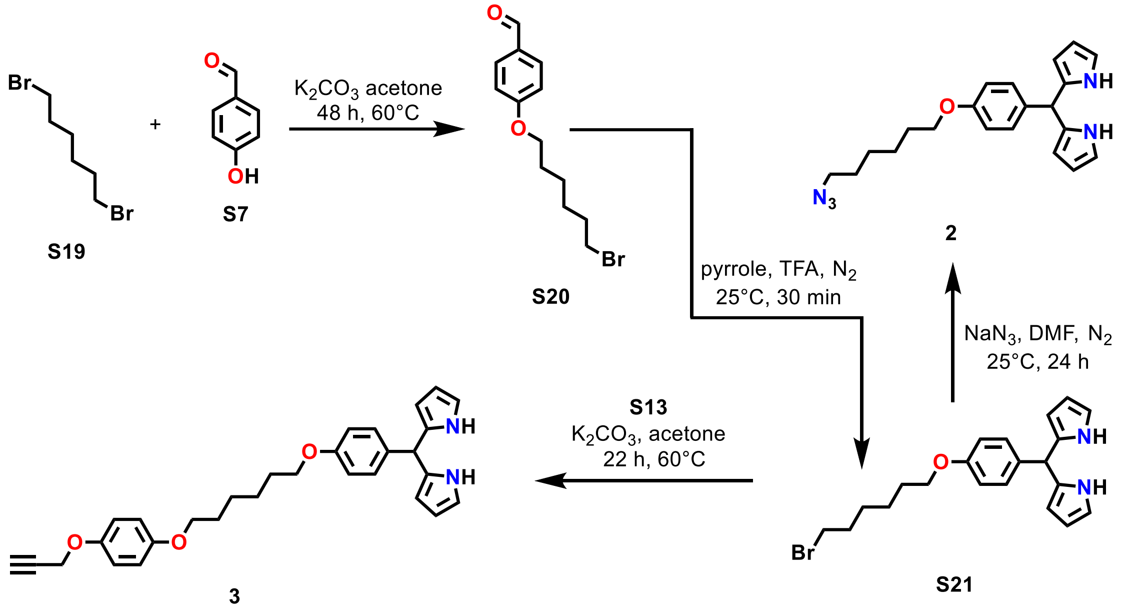

Scheme S6. The synthesis of **2** and **3**.[7]

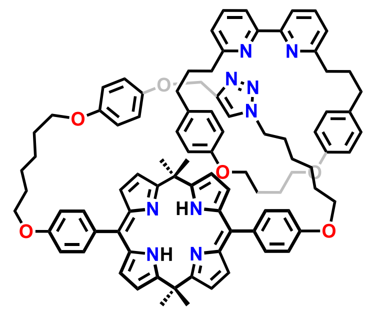

[2]cat^{4}

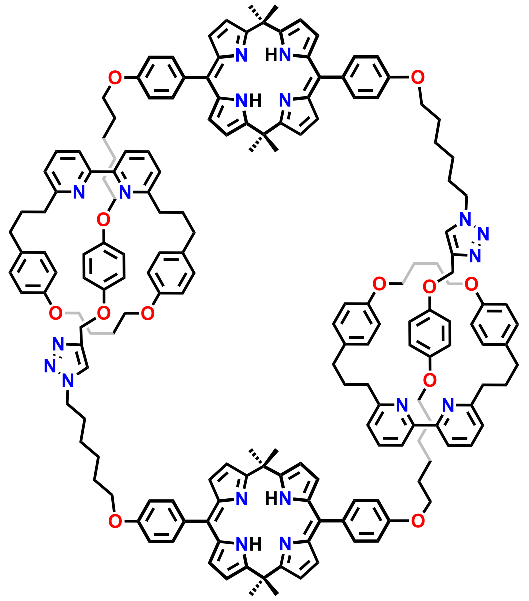

[3]cat$^{4}_{HT}$

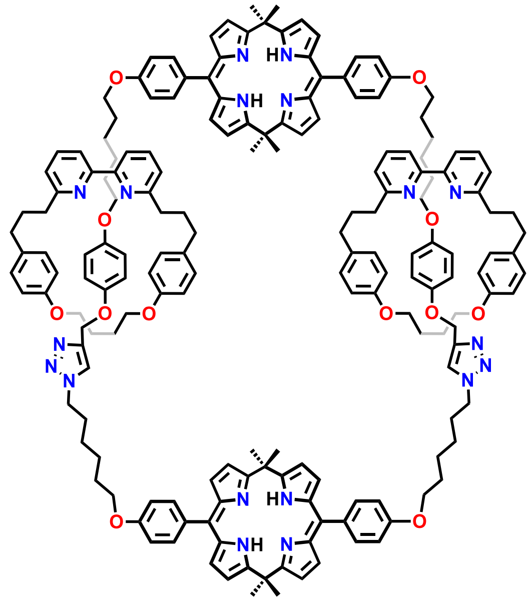

[3]cat$^{4}_{HH}$

Scheme S7. Structural formulae of the [*n*]catenanes described in the manuscript.

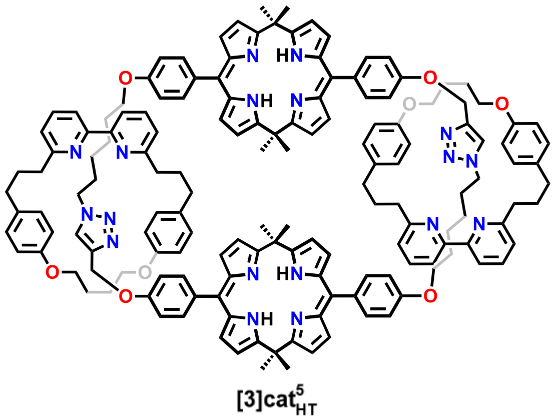

[3]cat$^{5}_{HT}$

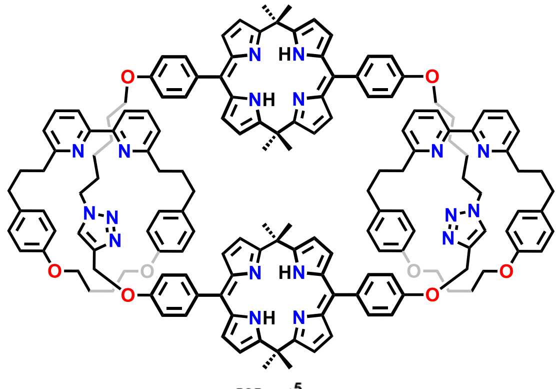

[3]cat$^{5}_{HH}$

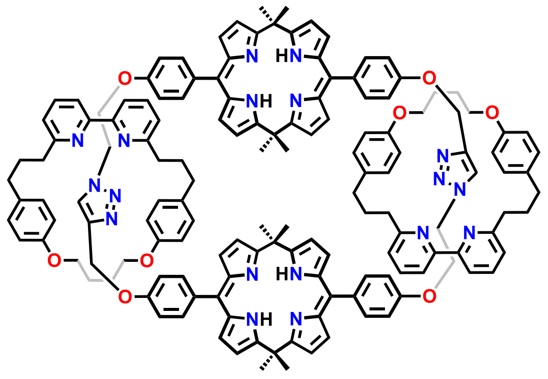

[3]cat$^{6}_{HT}$

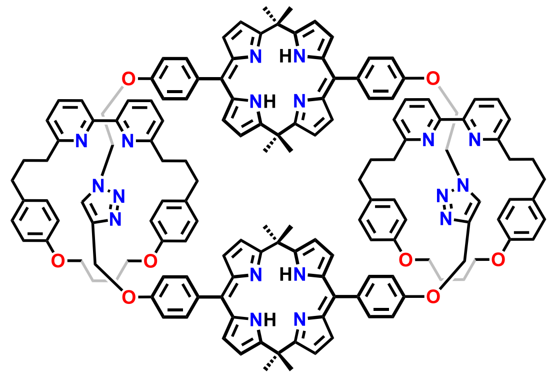

[3]cat$^{6}_{HH}$

Scheme S7. Structural formulae of the [*n*]catenanes described in the manuscript (continuation).

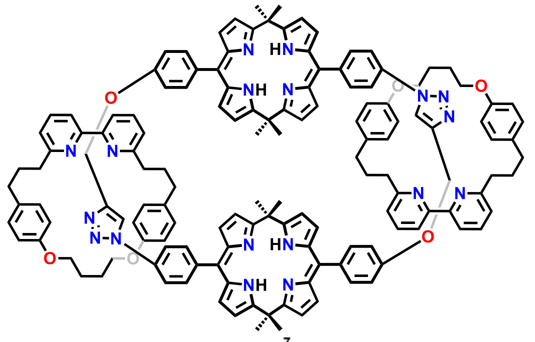

[3]cat$^{7}_{HT}$

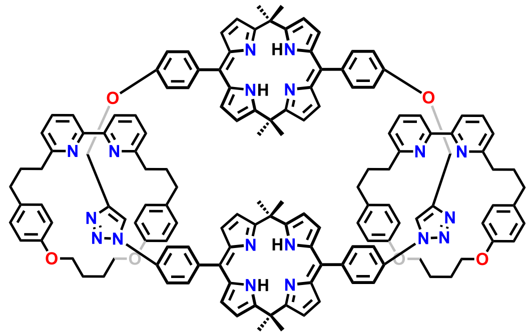

[3]cat$^{7}_{HH}$

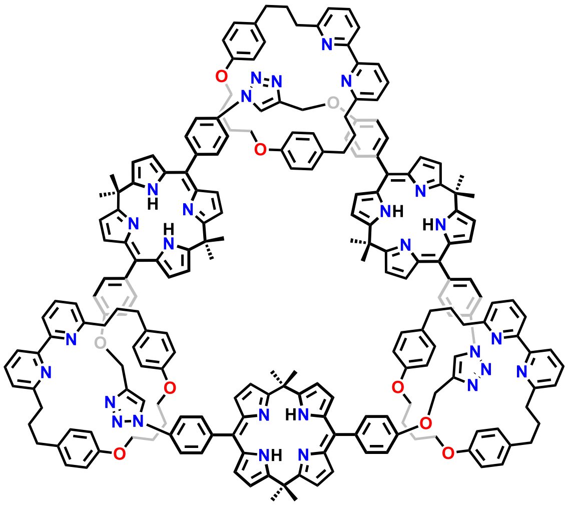

[4]cat$^{7}_{HT}$

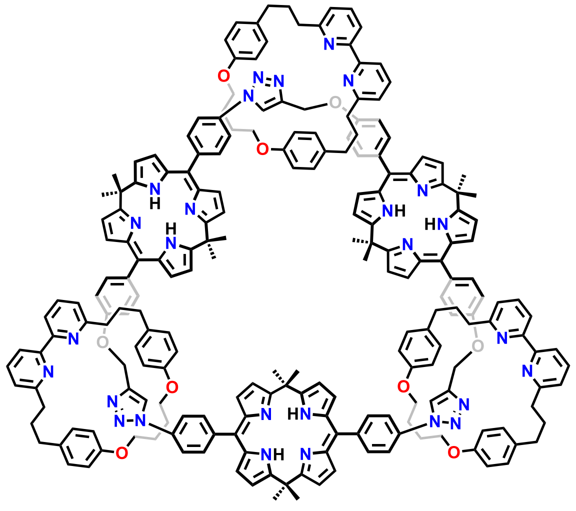

[4]cat$^{7}_{HH}$

Scheme S7. Structural formulae of the [*n*]catenanes described in the manuscript (continuation).

Compound S9

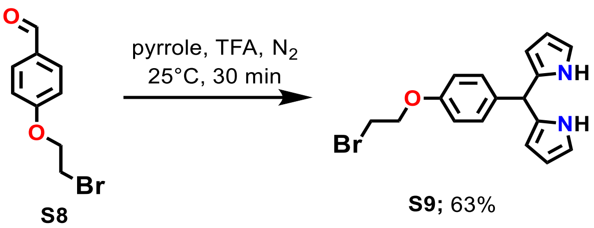

S8 S9; 63%

***S9** was obtained under modified literature conditions.[9] The analytical data are in agreement with the published data.[9]*

In a 50 mL round bottom flask, **S8** (2.335 g, 10.20 mmol) and pyrrole (30 mL, 433 mmol) were introduced. Pyrrole was purified by passing through a column filled with aluminum oxide before the reaction. The mixture was deoxygenated *via* nitrogen bubbling for 10 minutes. Then, TFA (79 µL, 1.03 mmol) was added by a syringe. The mixture was then stirred for 30 minutes under a nitrogen atmosphere. After this, the reaction was quenched by adding 1M NaOH (25 mL). The product was extracted with ethyl acetate (100 mL). The organic layer was washed with distilled water and then with brine. The aqueous phase was again extracted with ethyl acetate (100 mL). Collected organic layers were dried over Na_2SO_4. The filtrate was collected *via* gravity filtration, and the solvent was removed under reduced pressure. The dark oil was purified *via* column chromatography (silica gel, DCM:hexane 8:2) to provide **S9** (2.207 g, 6.40 mmol, 63%) as a yellow oil, which turned dark over time. **S9** was stored in a vial with a cap wrapped with parafilm in the fridge.

Compound S10

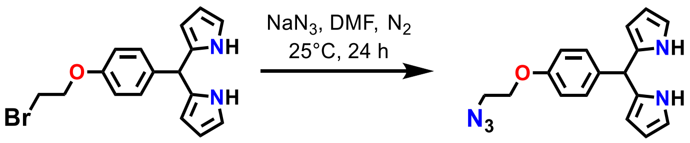

S9 S10; 99%

***S10** was obtained under modified literature conditions.[10] The analytical data are in agreement with the published data.[10]*

In a 25 mL round-bottom flask, **S9** (1.634 g, 4.74 mmol), sodium azide (387 mg, 5.92 mmol), and DMF (25 mL) were introduced. The flask was sealed with a rubber septum, and the solution was stirred at room temperature for 24 hours under a nitrogen atmosphere. The solvent was removed under reduced pressure. The residue was partitioned between ethyl acetate (100 mL) and distilled water (100 mL). The water phase was extracted with ethyl acetate (2 x 50 mL). Collected organic extracts were washed with brine and dried over anhydrous Na_2SO_4. The

drying agent was removed *via* gravity filtration, and the filtrate was evaporated to dryness to provide **S10** (1.438 g, 4.68 mmol, 99%) as a greenish oil. The product was used without further purification. **S10** was stored in a vial with a cap wrapped with parafilm in the fridge.

Compound S16

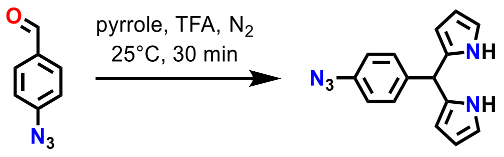

S15 **S16**; 43%

***S16** was obtained under modified literature conditions.[10] The analytical data are in agreement with the published data.[10]*

In a 25 mL round bottom flask, **S15** (962 mg, 6.54 mmol) and pyrrole (12 mL, 173.2 mmol) were introduced. Pyrrole was purified by passing through a column filled with aluminum oxide before the reaction. The mixture was deoxygenated *via* nitrogen bubbling for 10 minutes. Then, TFA (70 µL, 0.91 mmol) was added by a syringe. The mixture was then stirred for 30 minutes under a nitrogen atmosphere. After this, the reaction was quenched by adding 1M NaOH (25 mL). The product was extracted with ethyl acetate (100 mL). The organic layer was washed with distilled water and then with brine. The aqueous phase was again extracted with ethyl acetate (100 mL). Collected organic layers were dried over Na_2SO_4. The filtrate was collected *via* gravity filtration, and the solvent was removed under reduced pressure. The dark oil was purified *via* column chromatography (silica gel, DCM:hexane 7:3) to provide **S16** (732 mg, 2.78 mmol, 43%) as a burgundy oil, which turned dark over time. **S16** was stored in a vial with a cap wrapped with parafilm in the fridge.

Dipyrromethane stoppered rotaxane synthesis.

[2]Rotaxane 5

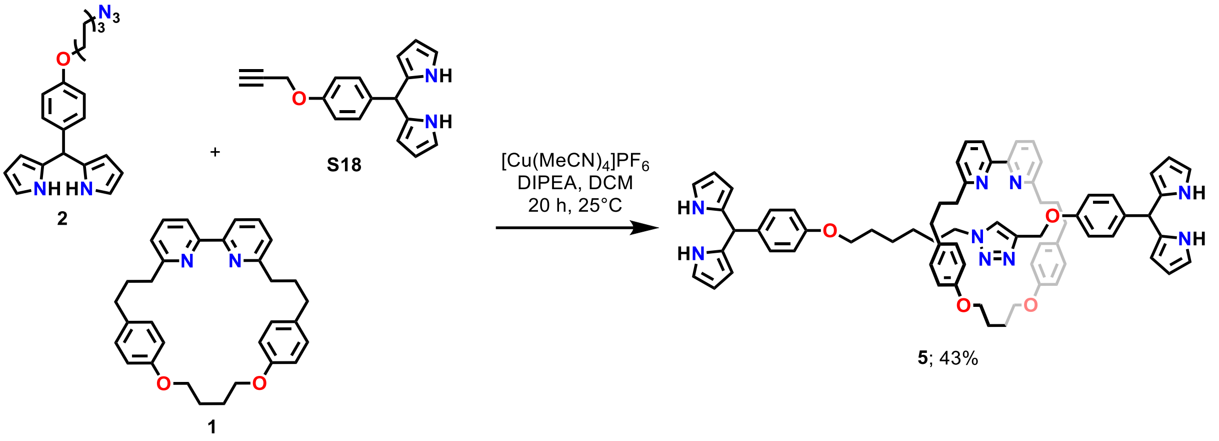

In a 10 mL vial with a cap, macrocycle **1** (100 mg, 210 µmol), azide **2** (110 mg, 303 µmol), alkyne **S18** (80 mg, 290 µmol), [Cu(CH_3CN)$_4$]PF_6 (15.5 mg, 42 µmol), DIPEA (36 µL, 207 µmol) were dissolved in DCM (2 mL). After mixing the reagents, the solution immediately turned dark orange. The vial was sealed, and the cap was secured with parafilm. The mixture was stirred for 20 hours at room temperature. After this time, the mixture was transferred into a separatory funnel, and DCM (50 mL) was introduced. Subsequently, aqueous ammonia solution (50 mL) and EDTA (200 mg, 685 µmol) were added to the solution in a separatory funnel, and upon the one-minute-long shaking, the solution turned dark. The aqueous phase was extracted with DCM (50 mL). The collected organic extracts were washed with water and brine. The aqueous phase was once more extracted with DCM (50 mL). The collected organic layers were combined and dried over anhydrous Na_2SO_4. The filtrate was collected *via* gravity filtration, and the solvent was removed under reduced pressure. The dark green oil was purified *via* flash chromatography (DCM with 5-25% ethyl acetate gradient) to provide 5 (101 mg, 90 µmol, 43%) as a yellowish solid that turned dark after time. **5** was stored in a vial with a cap wrapped with parafilm in the fridge.

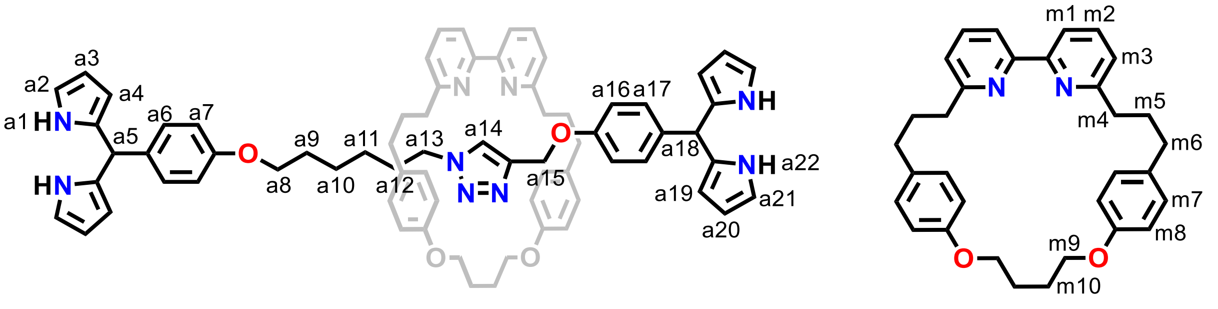

^{1}H NMR (500 MHz, $CDCl_3$, 300 K) δ (ppm) 8.15 (s, 1H, Ha14), 7.95 (b, 2H, Ha1), 7.85 (b, 2H, Ha22), 7.58 (t, 2H, ^3J = 7.7 Hz, Hm2), 7.35 (dd, 2H, ^3J = 7.8, ^4J = 0.7 Hz, Hm1), 7.10–7.06 (overlapping, 4H, Ha6, Hm3), 7.04 (d, 2H, ^3J = 8.6 Hz, Ha17), 6.85 (d, 2H, ^3J = 8.7 Hz, Ha16), 6.75 (d, 2H, ^3J = 8.8, Ha7), 6.71 (d, 4H, ^3J = 8.6 Hz, Hm7), 6.66–6.64 (m, 2H, Ha2), 6.62 (d,

4H, ^3J = 8.6 Hz, Hm8), 6.60–6.58 (m, 2H, Ha21), 6.14 (dd, ^3J = 6.4 Hz, ^3J = 2.8 Hz, Ha3), 6.10 (dd, ^3J = 6.4 Hz, ^3J = 2.8 Hz, Ha20), 5.91–5.88 (m, 2H, Ha4), 5.87–5.84 (m, 2H, Ha19), 5.40 (s, 1H, Ha5), 5.35 (s, 1H, Ha18), 4.92 (s, 2H, Ha15), 4.27–4.19 (m, 2H, Hm9), 4.02–3.95 (m, 2H, Hm9), 3.64 (t, 2H, ^3J = 6.6 Hz, Ha8), 3.27–3.23 (m, 2H, Ha13), 2.62–2.42 (overlapping m, 8H, Hm4, Hm6), 2.11–2.00 (m, 2H, Hm10), 1.93–1.64 (overlapping m, 6H, Ha10, Hm5), 1.37–1.29 (m, 2H, Ha9), 0.92–0.83 (m, 2H, Ha10), 0.73–0.56 (overlapping m, 4H, Ha11, Ha12).
^{13}C NMR (125 MHz, $CDCl_3$, 300 K) δ (ppm) 162.5, 158.1, 157.7, 157.41, 157.38, 142.3, 136.8, 134.3, 133.9, 133.2, 132.9, 132.8, 129.34, 129.32 (2 overlapping signals), 124.6, 121.4, 120.3, 117.07, 117.06, 114.9, 114.8, 114.6, 108.42, 108.36, 107.01, 106.99, 67.6, 66.4, 61.7, 49.4, 43.2, 43.1, 36.9, 34.7, 31.8, 28.7 (2 overlapping signals), 25.8, 25.0, 24.8.
HRMS (ESI+, TOF): [2]rotaxane **5** was oxidized with DDQ to record the MS spectrum; *m/z*: $[M+H]^+$ calcd. for $C_{71}H_{72}N_9O_4^+$, 1114.5702; found, 1114.5596.

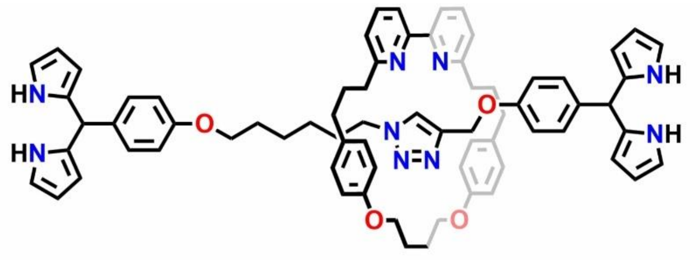

5

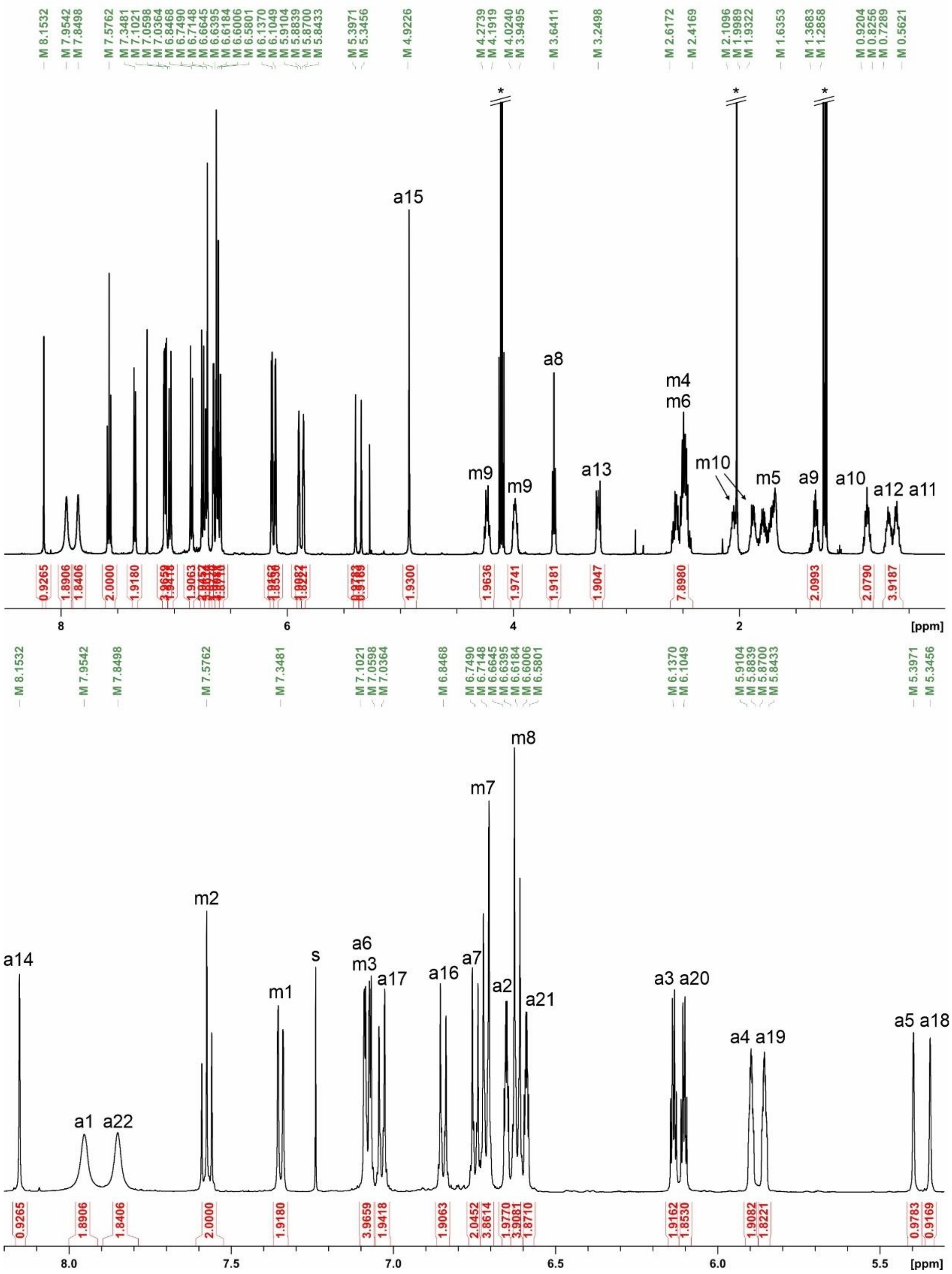

Figure S1. The ^{1}H NMR spectrum of **5** (500 MHz, $CDCl_3$, 300 K).

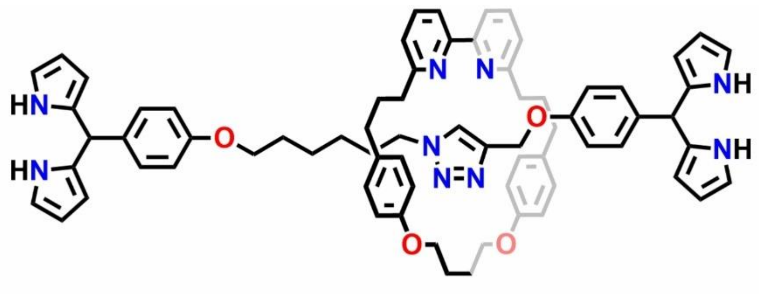

5

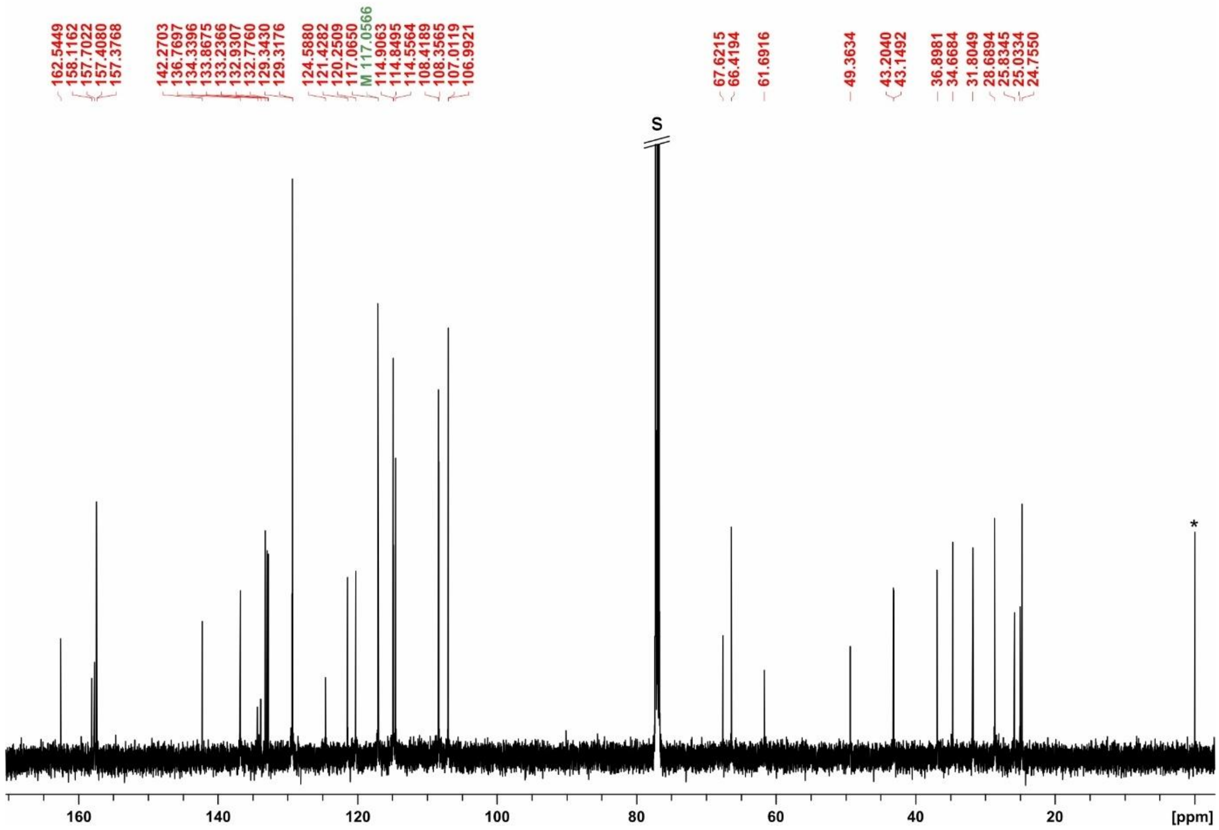

Figure S2. The ^{13}C NMR spectrum of **5** (125 MHz, $CDCl_3$, 300 K).

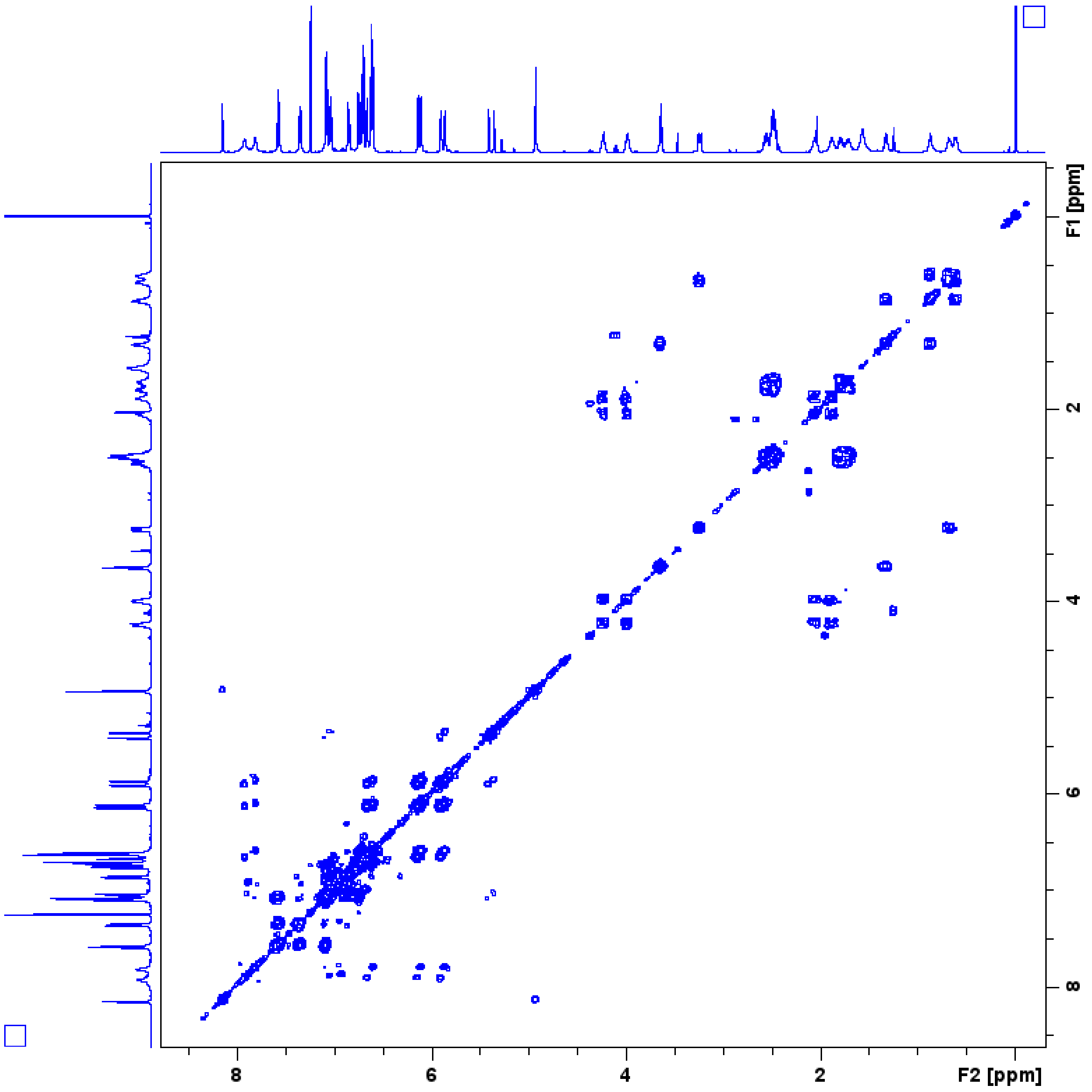

Figure S3. The ^{1}H-^{1}H COSY NMR spectrum of **5** (500 MHz, $CDCl_3$, 300 K).

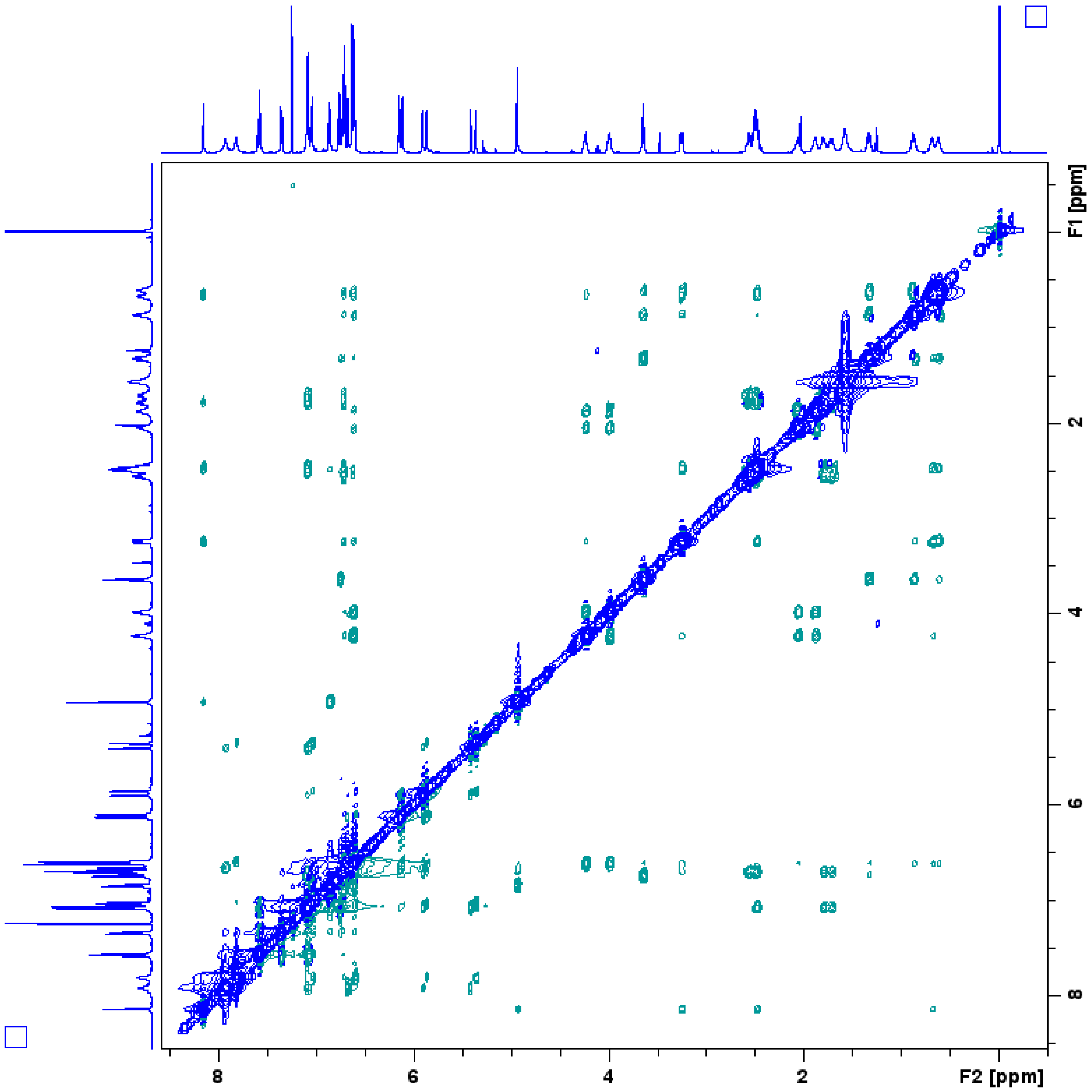

Figure S4. The ^{1}H-^{1}H NOESY NMR spectrum of **5** (500 MHz, $CDCl_3$, 300 K).

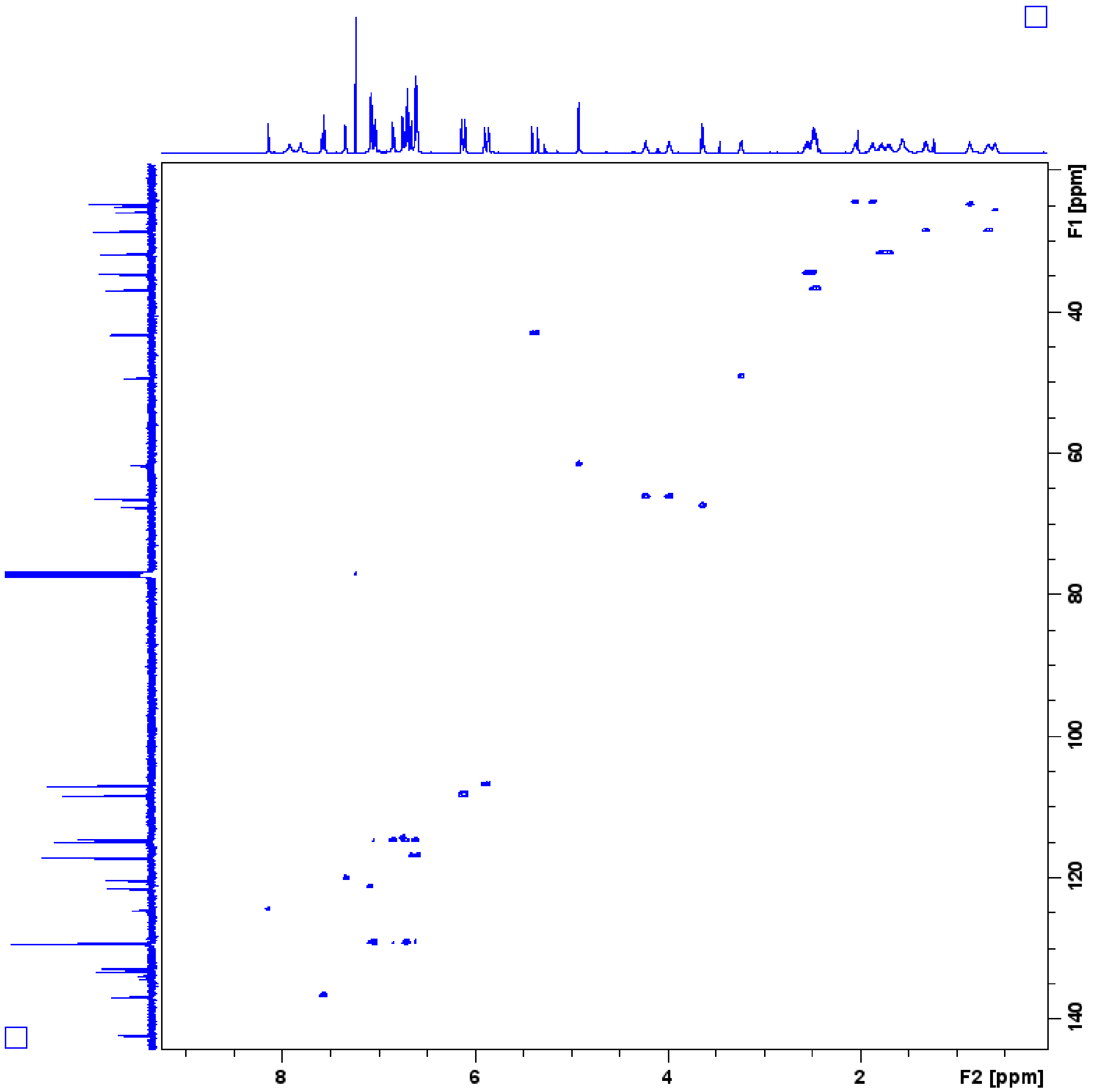

Figure S5. The ¹H-¹³C HSQC NMR spectrum of **5** (500 MHz, $CDCl_3$, 300 K).

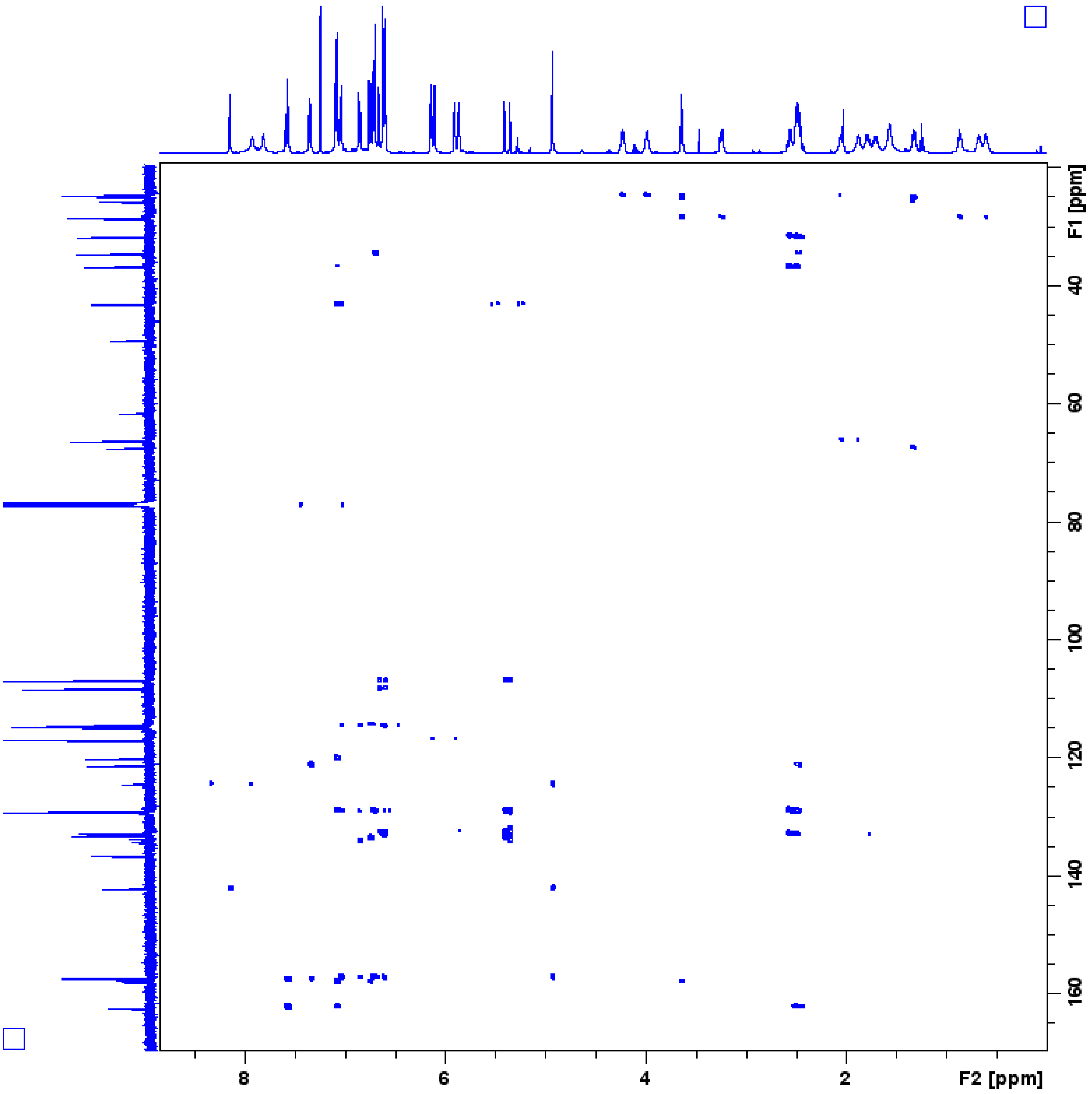

Figure S6. The ^{1}H-^{13}C HMBC NMR spectrum of **5** (500 MHz, $CDCl_3$, 300 K).

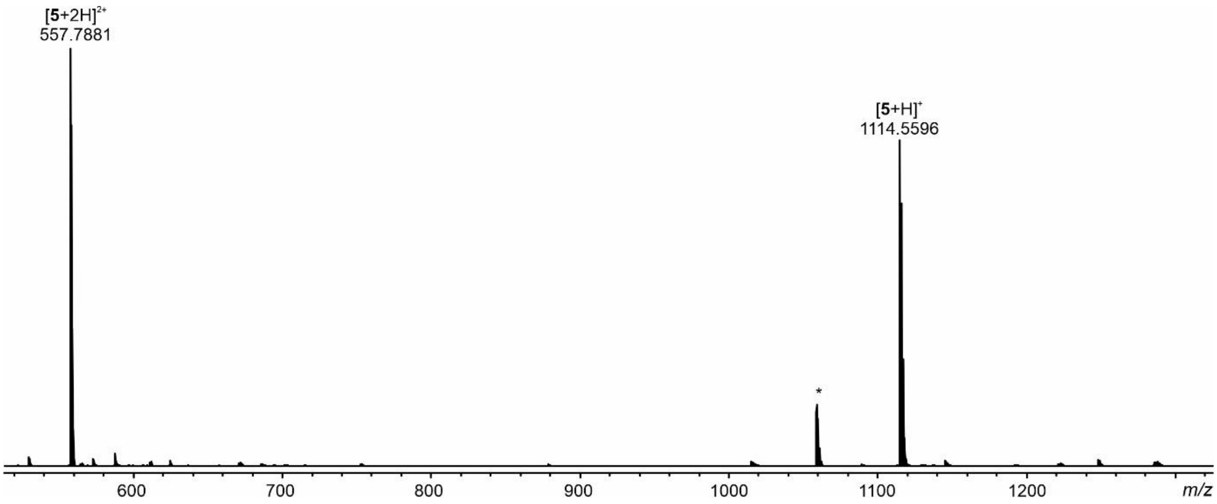

Figure S7. The ESI (TOF) mass spectrum of **5**.

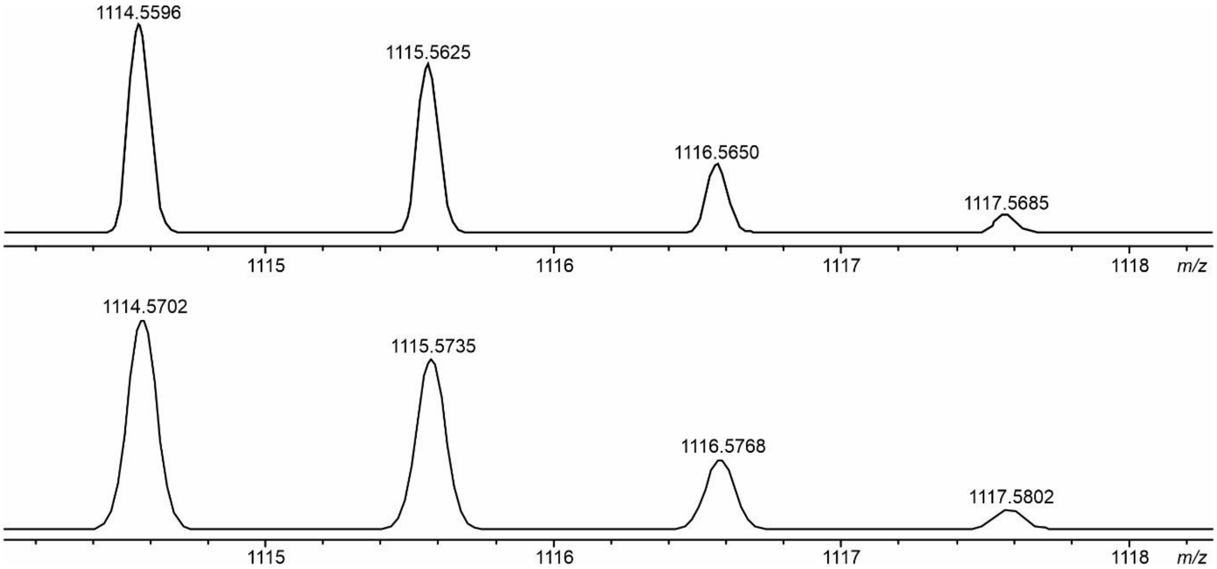

Figure S8. The ESI (TOF) mass spectrum of **5.** Top: experimental, bottom: simulated isotopic pattern.

[2]Rotaxane 6

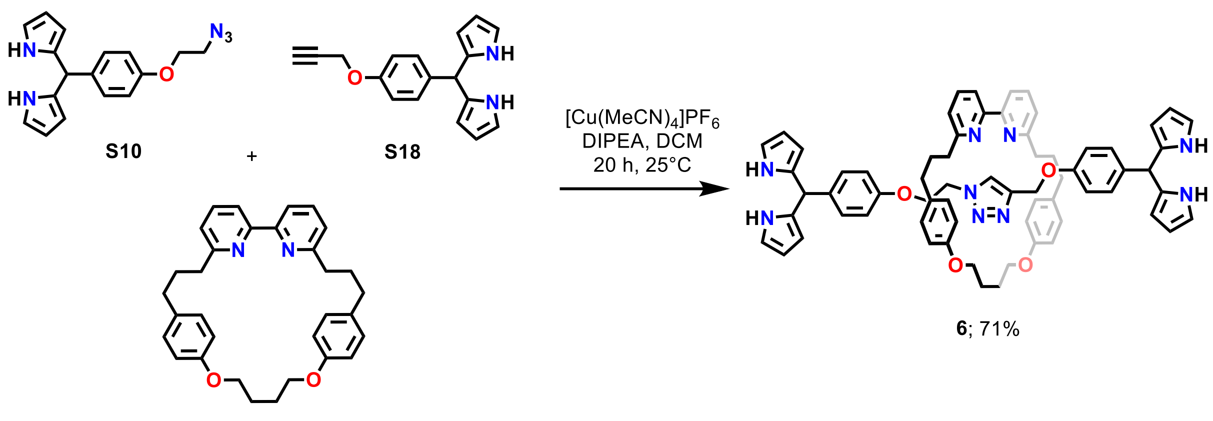

In a 10 mL vial with a cap, macrocycle **1** (100 mg, 210 μmol), azide **S10** (89 mg, 290 μmol), alkyne **S18** (80 mg, 290 μmol), $[Cu(CH_3CN)_4]PF_6$ (15.5 mg, 42 μmol), DIPEA (36 μL, 207 μmol) were dissolved in DCM (2 mL). After mixing the reagents, the solution immediately turned dark orange. The vial was sealed, and the cap was secured with parafilm. The mixture was stirred for 20 hours at room temperature. After this time, the mixture was transferred into a separatory funnel, and DCM (50 mL) was introduced. Subsequently, aqueous ammonia solution (50 mL) and EDTA (200 mg, 685 μmol) were added to the solution in a separatory funnel, and upon the one-minute-long shaking, the solution turned dark. The aqueous phase was extracted with DCM (50 mL). The collected organic extracts were washed with water and brine. The aqueous phase was once more extracted with DCM (50 mL). The collected organic layers were combined and dried over anhydrous Na_2SO_4. The filtrate was collected *via* gravity filtration, and the solvent was removed under reduced pressure. The obtained dark green oil was purified *via* flash chromatography (DCM with 5-25% ethyl acetate gradient) to provide **6** (158 mg, 149 μmol, 71%) as a yellowish solid, which turned dark after time. **6** was stored in the fridge in *a* vial with a cap wrapped with parafilm.

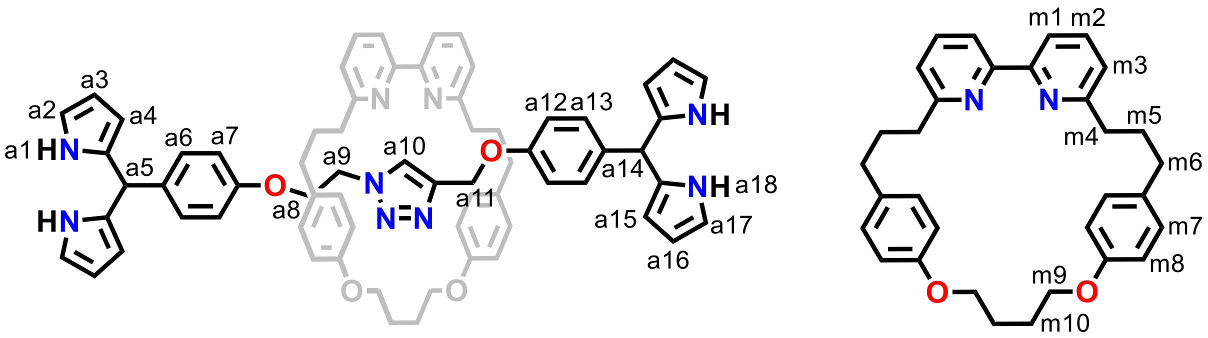

^{1}H NMR (500 MHz, $CDCl_3$, 300 K) δ (ppm): 8.66 (s, 1H, Ha10), 7.89 (b, 2H, Ha1/Ha18), 7.82 (b, 2H, Ha1/Ha18), 7.56 (t, 2H, ^3J = 7.8 Hz, Hm2), 7.34 (dd, 2H, ^3J = 7.8 Hz, ^4J = 0.7 Hz, Hm1), 7.03 (dd, 2H, ^3J = 7.8 Hz, ^4J = 0.7 Hz, Hm3), 7.00 (d, 2H, ^3J = 8.6 Hz, Ha13), 6.86 (d, 2H, ^3J =

8.6 Hz, Ha6), 6.77 (d, 2H, ^3J = 8.7 Hz, Ha12), 6.68–6.66 (m, 2H, Ha2/Ha17), 6.64 (d, 4H, ^3J = 8.6 Hz, Hm7), 6.62–6.60 (m, 2H, Ha2/Ha17), 6.58 (d, 4H, ^3J = 8.6 Hz, Hm8), 6.44 (d, 2H, ^3J = 8.7 Hz, Ha7), 6.16–6.12 (m, 2H, Ha3/Ha16), 6.12–6.08 (m, 2H, Ha3/Ha16), 5.87–5.83 (overlapping m, 4H, Ha4, Ha15), 5.34 (s, 1H, Ha14), 5.33 (s, 1H, Ha5), 4.84 (s, 2H, Ha11), 4.34–4.24 (m, 2H, Hm9), 4.06–3.99 (m, 2H, Hm9), 3.61 (t, 2H, ^3J = 7.4 Hz, Ha9), 3.31 (t, 2H, ^3J = 7.2 Hz, Ha8), 2.58–2.49 (m, 2H, Hm6), 2.49–2.35 (m, 4H, Hm4, Hm6), 2.35–2.25 (m, 2H, Hm4), 2.16–2.06 (m, 2H, Hm10), 1.99–1.88 (m, 2H, Hm10), 1.77–1.57 (overlapping, 4H, Hm5)
^{13}C NMR (125 MHz, $CDCl_3$, 300 K) δ (ppm): 162.7, 157.6, 157.34, 157.27, 157.0, 142.5, 136.8, 134.2, 133.8, 133.1, 132.9, 132.8, 129.3, 129.2, 128.8, 126.0, 121.5, 120.3, 117.3, 117.0, 114.9 (2 overlapping signals), 114.8, 108.4 (2 overlapping signals), 107.0 (2 overlapping signals), 66.4, 64.7, 61.5, 47.8, 43.17, 43.13, 37.0, 34.9, 31.9, 24.8.
HRMS (ESI+, TOF): *m/z*: $[M+H]^+$ calcd. for $C_{67}H_{68}N_9O_4^+$, 1062.5389; found, 1062.5193.

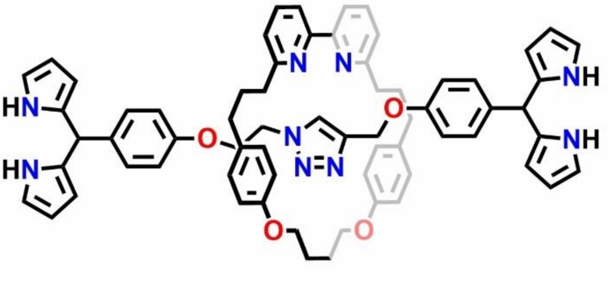

6

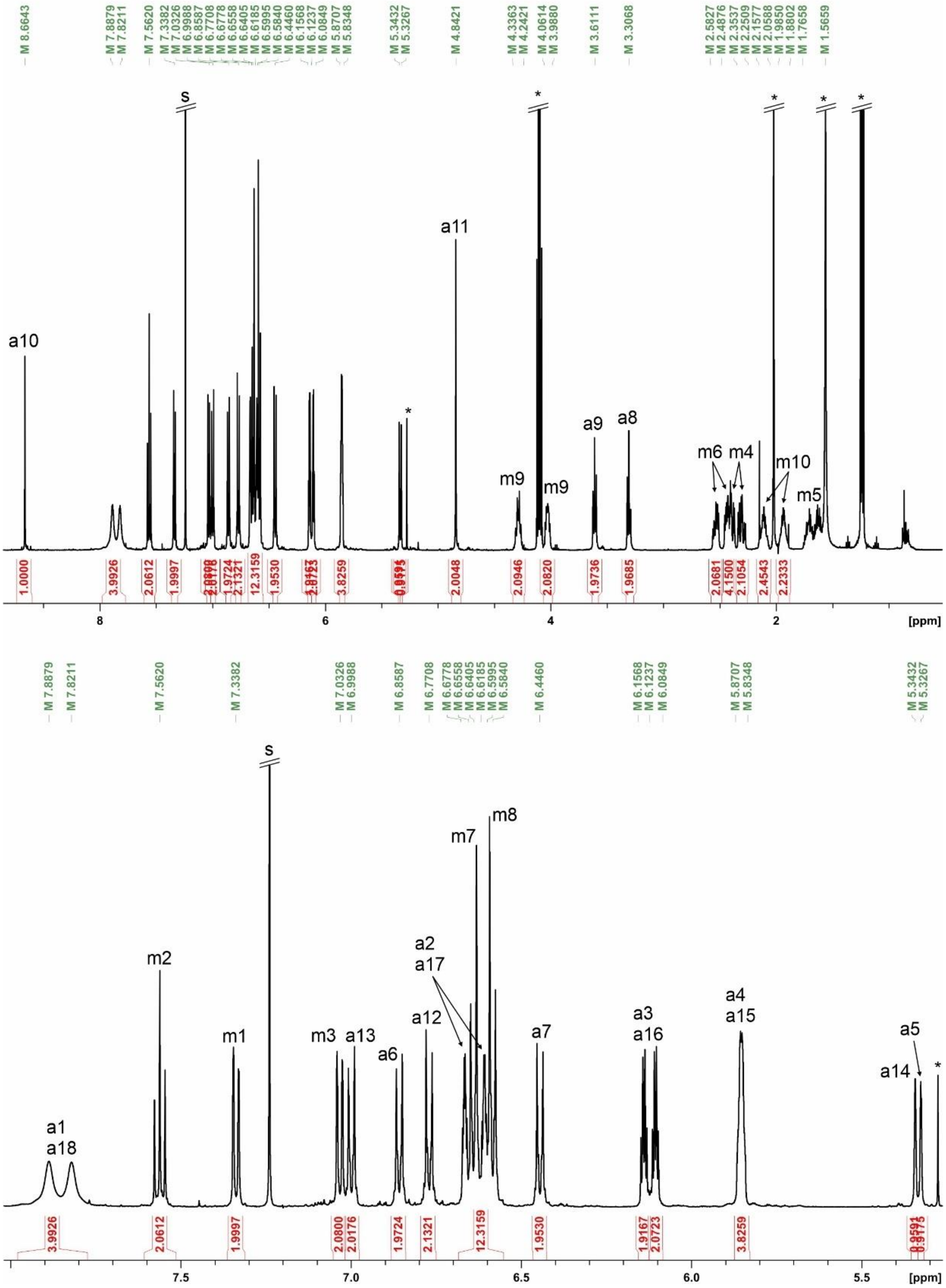

Figure S9. The ^{1}H NMR spectrum of **6** (500 MHz, $CDCl_3$, 300 K).

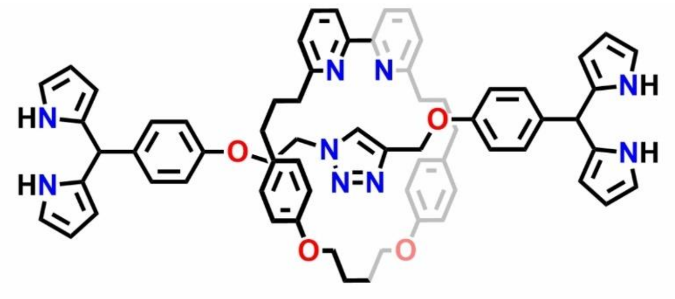

6

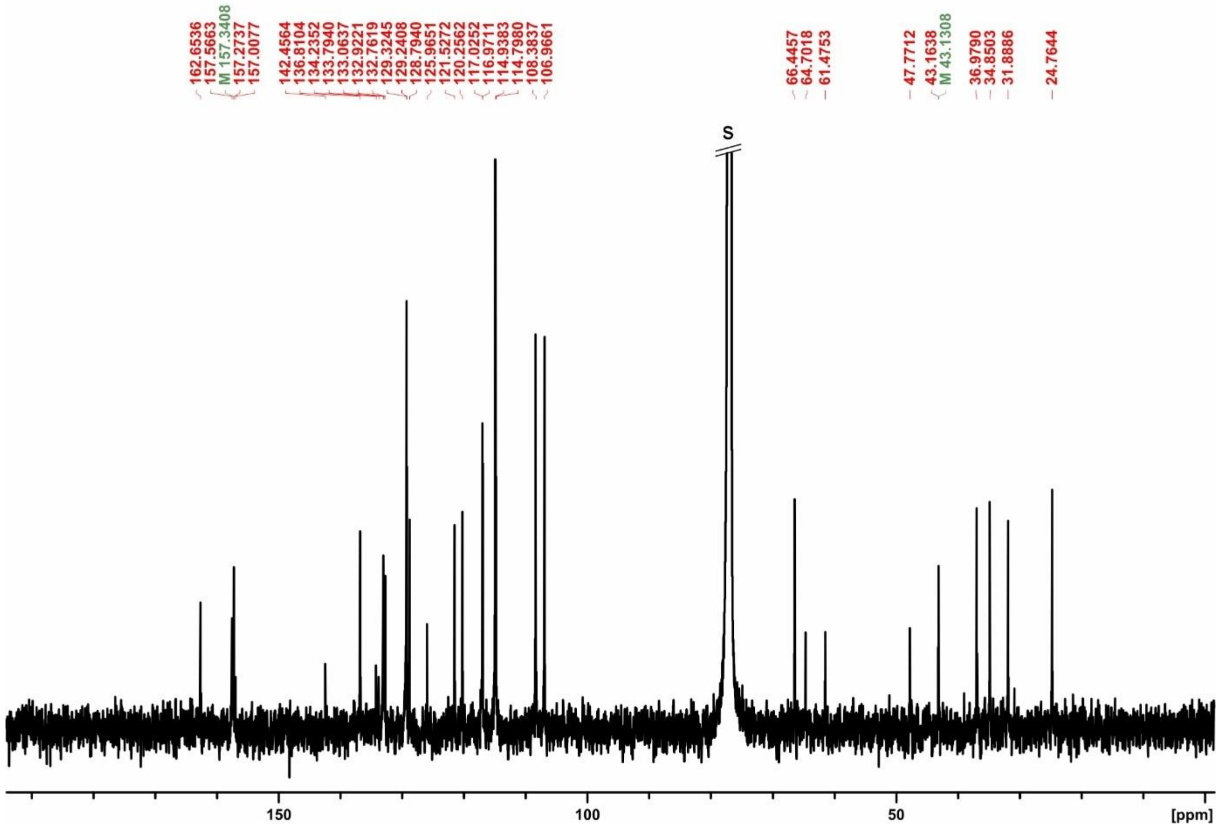

Figure S10. The ^{13}C NMR spectrum of **6** (125 MHz, $CDCl_3$, 300 K).

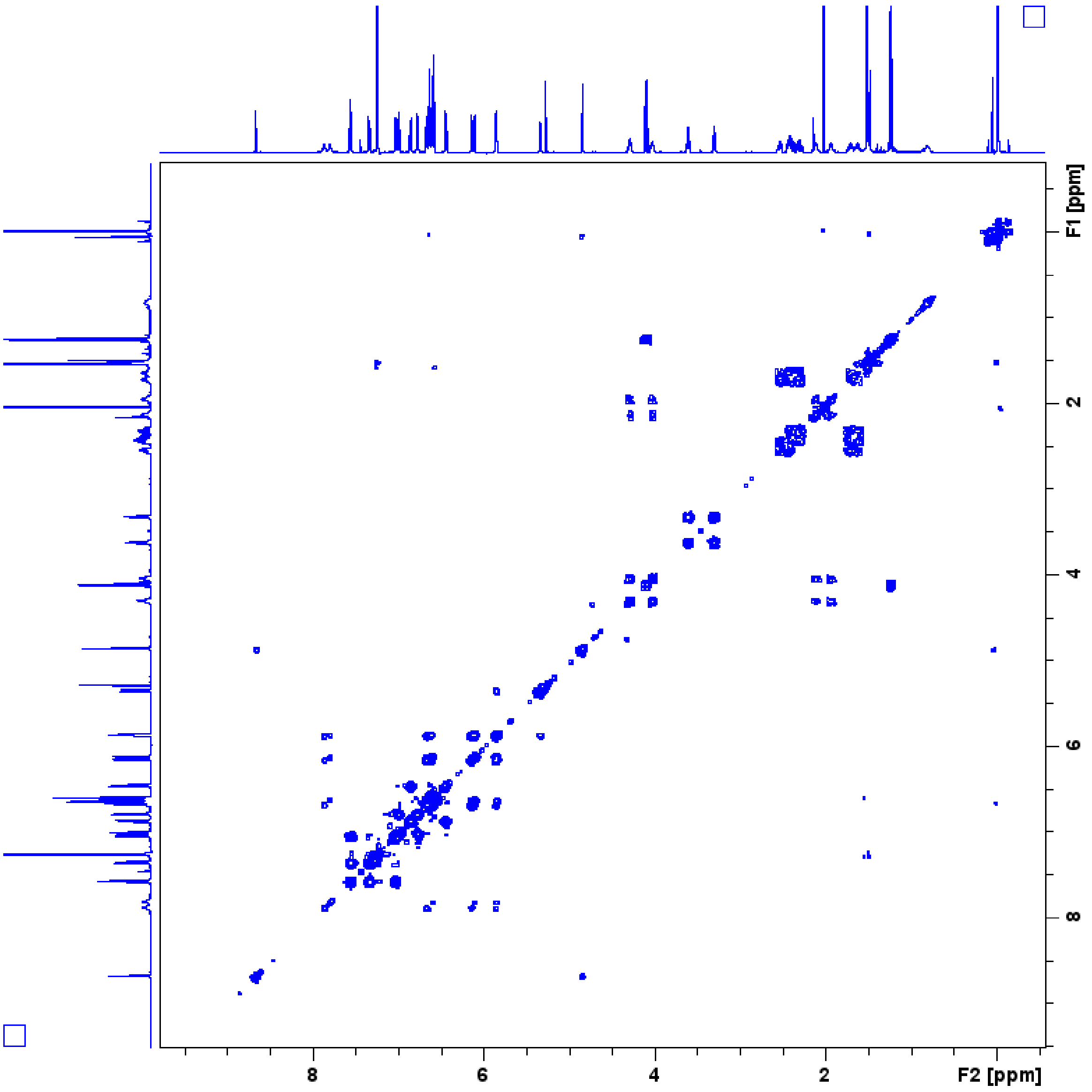

Figure S11. The ^{1}H-^{1}H COSY NMR spectrum of **6** (500 MHz, $CDCl_3$, 300 K).

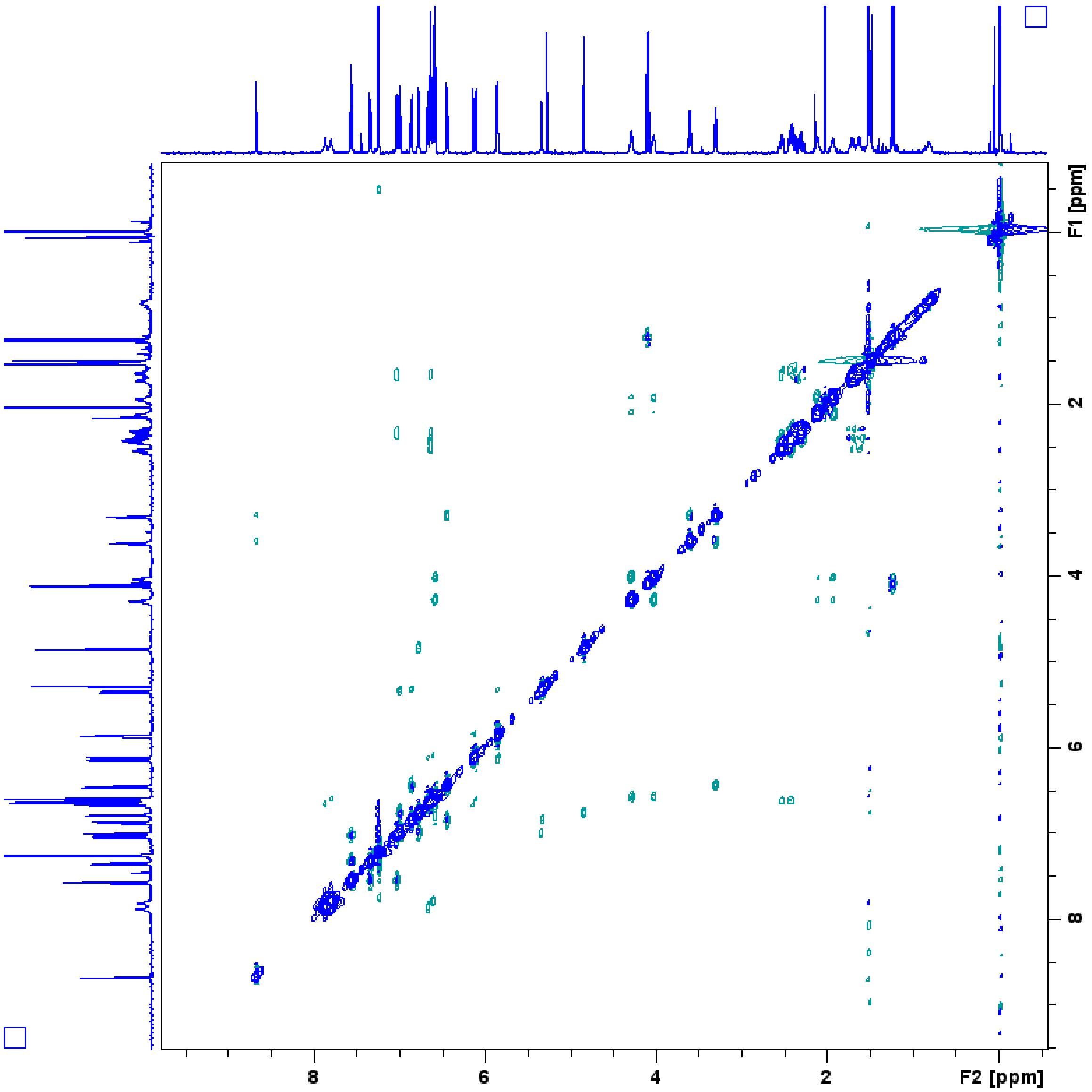

Figure S12. The ^1H-^1H NOESY NMR spectrum of **6** (500 MHz, $CDCl_3$, 300 K).

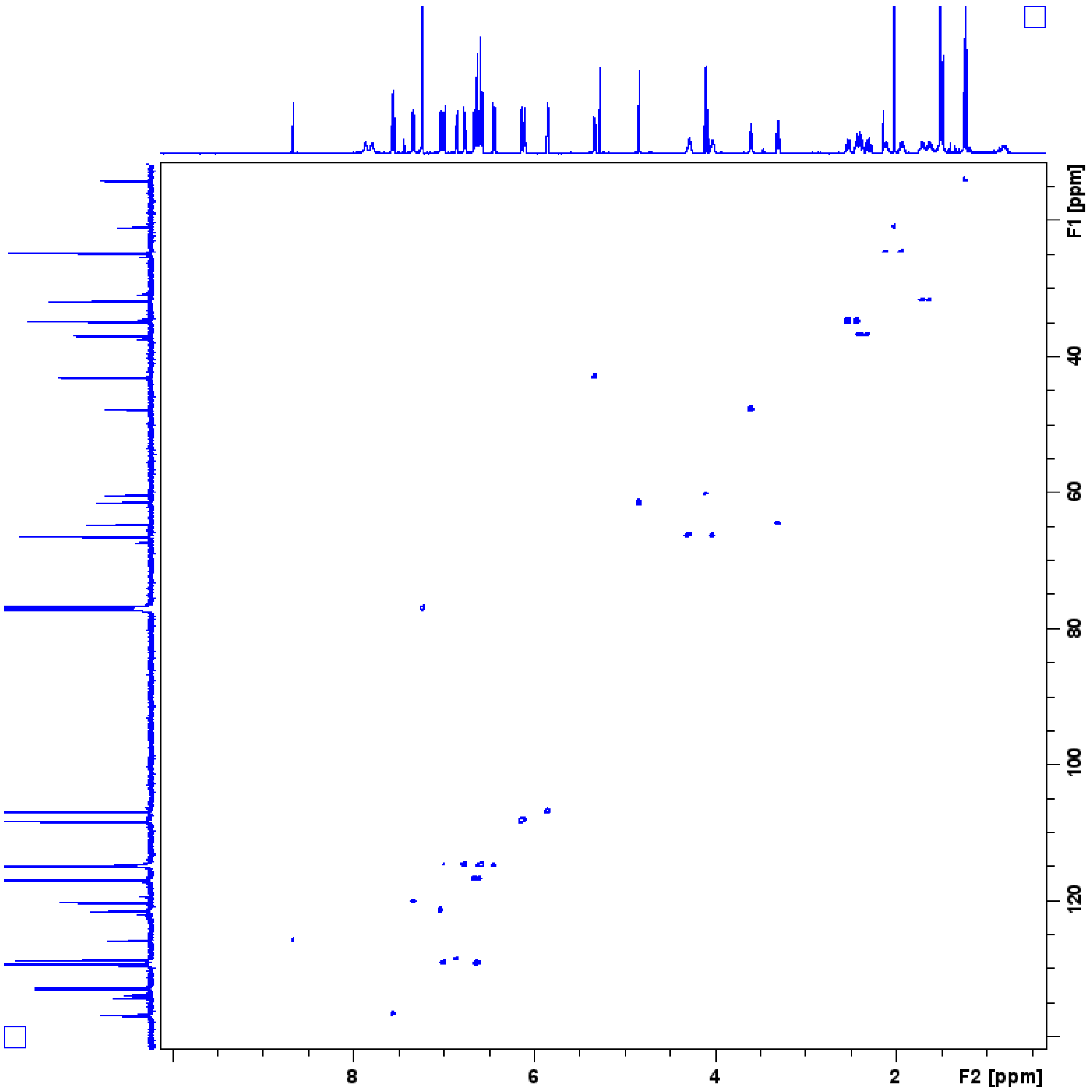

Figure S13. The ^{1}H-^{13}C HSQC NMR spectrum of **6** (500 MHz, $CDCl_3$, 300 K).

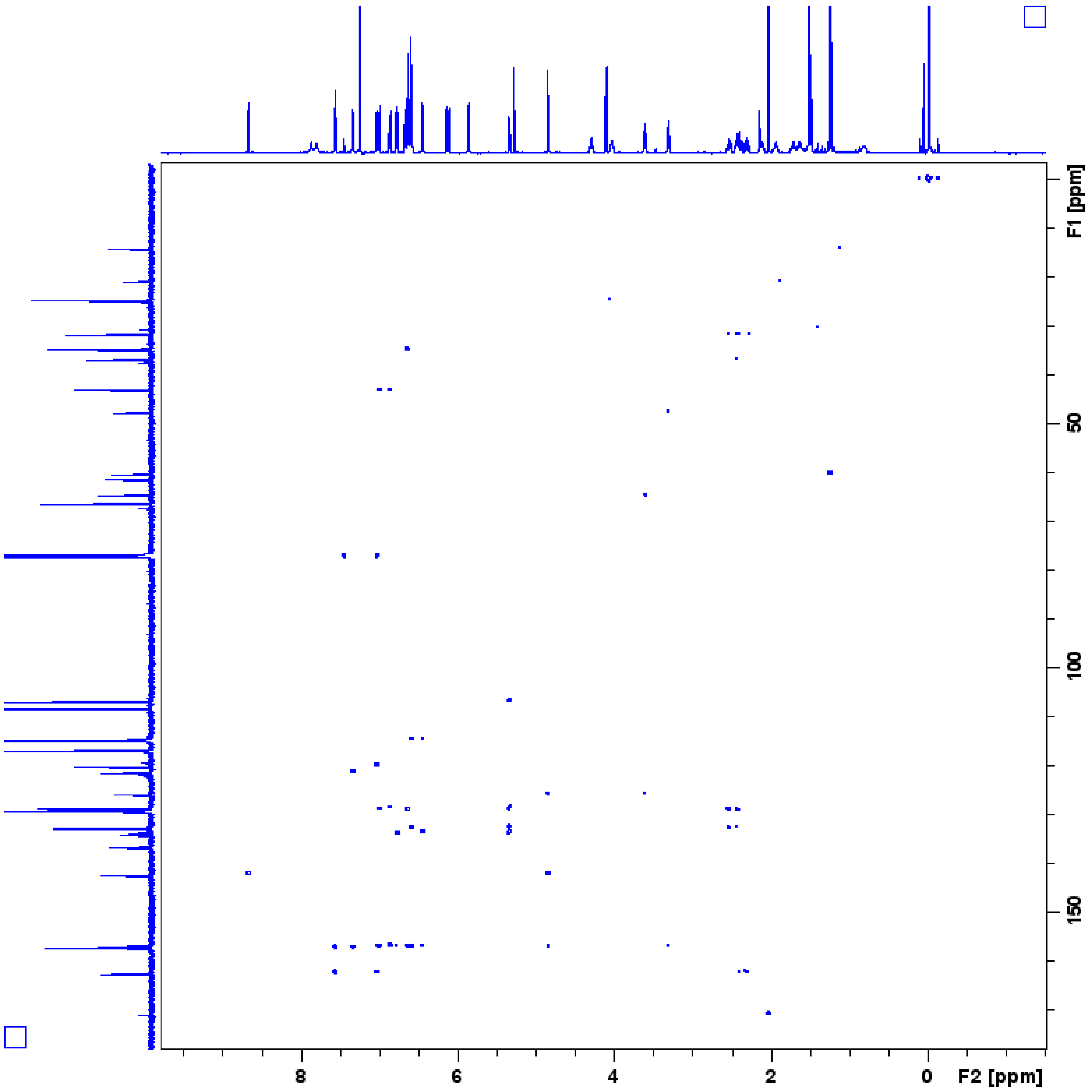

Figure S14. The ^{1}H-^{13}C HMBC NMR spectrum of **6** (500 MHz, $CDCl_3$, 300 K).

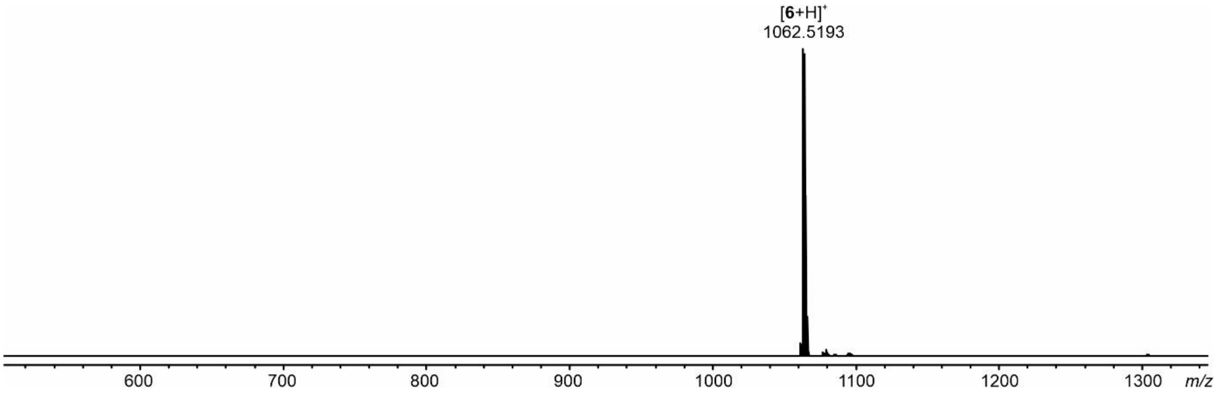

Figure S15. The ESI (TOF) mass spectrum of **6**.

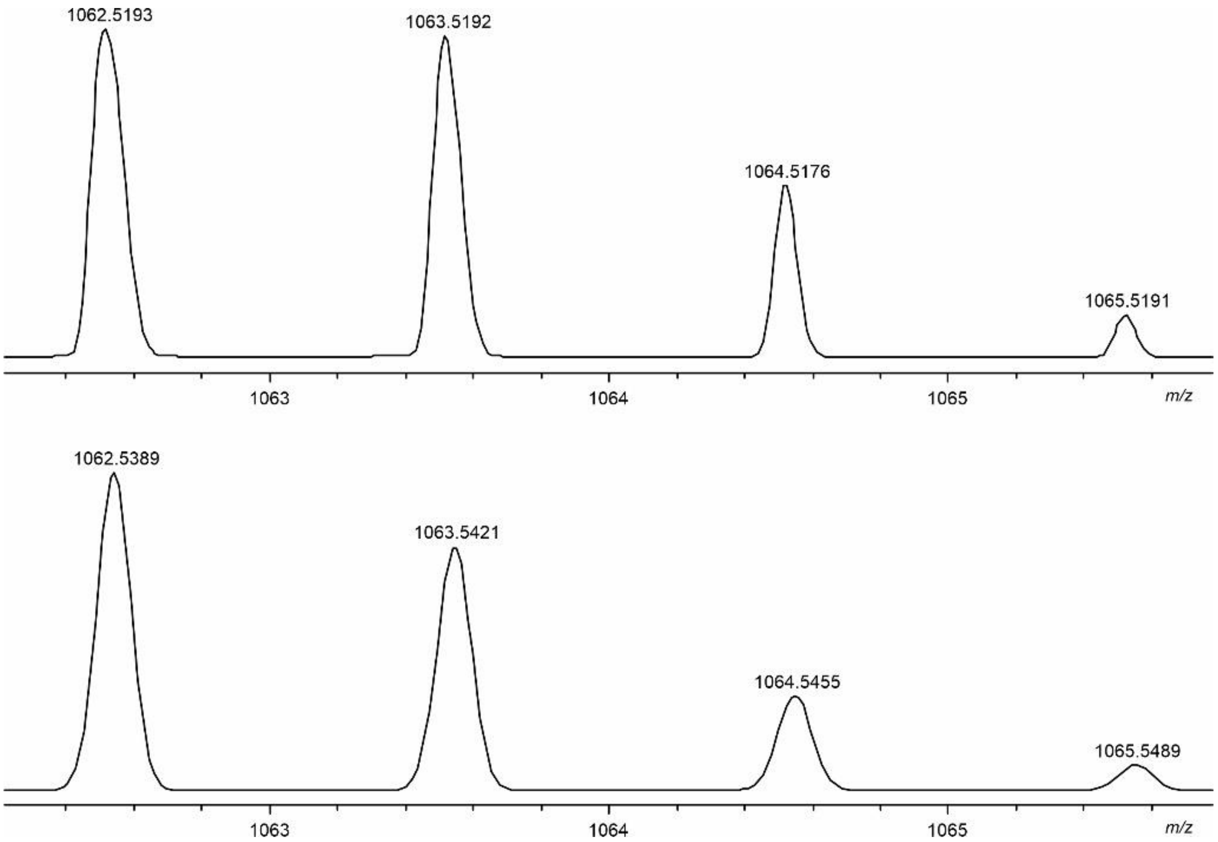

Figure S16. The ESI (TOF) mass spectrum of **6.** Top: experimental, bottom: simulated isotopic pattern.

[2]Rotaxane 7

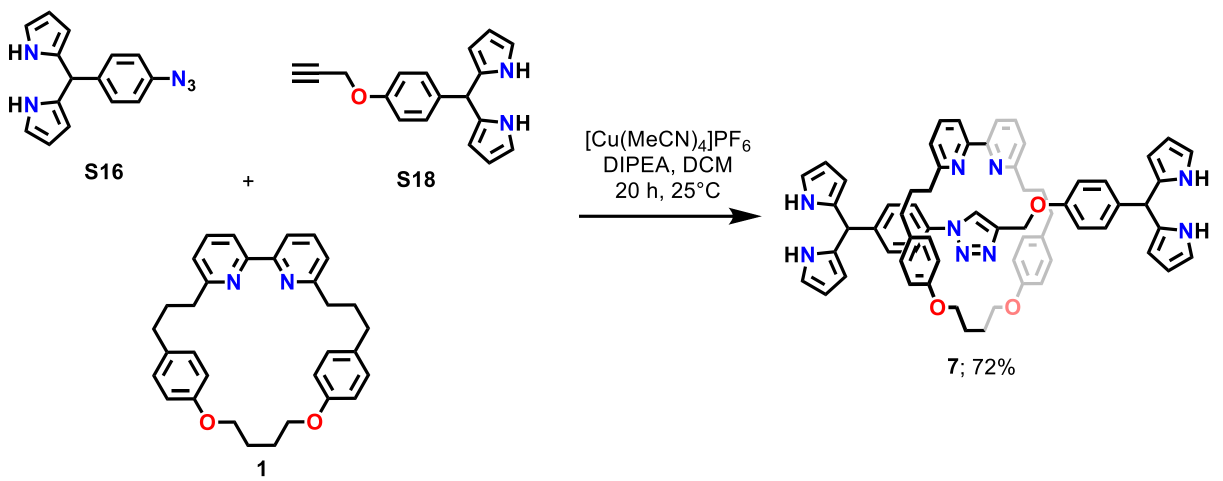

In a 10 mL vial with a cap, macrocycle **1** (100 mg, 210 µmol), azide **S16** (76 mg, 289 µmol), alkyne **S18** (80 mg, 290 µmol), $[Cu(CH_3CN)_4]PF_6$ (15.5 mg, 42 µmol), DIPEA (36 µL, 207 µmol) were dissolved in DCM (2 mL). After mixing the reagents, the solution immediately turned dark orange. The vial was sealed, and the cap was secured with parafilm. The mixture was stirred for 20 hours at room temperature. After this time, the mixture was transferred into a separatory funnel, and DCM (50 mL) was introduced. Subsequently, aqueous ammonia solution (50 mL) and EDTA (200 mg, 685 µmol) were added to the solution in a separatory funnel, and upon the one-minute-long shaking, the solution turned dark. The aqueous phase was extracted with DCM (50 mL). The collected organic extracts were washed with water and brine. The aqueous phase was once more extracted with DCM (50 mL). The collected organic layers were combined and dried over anhydrous Na_2SO_4. The filtrate was collected *via* gravity filtration, and the solvent was removed under reduced pressure. The obtained dark green oil was purified *via* flash chromatography (DCM with 5-25% ethyl acetate gradient) to provide **7** (153 mg, 151 µmol, 72%) as a yellowish solid, which turned dark after time. **7** was stored in the fridge in a vial with a cap wrapped with parafilm.

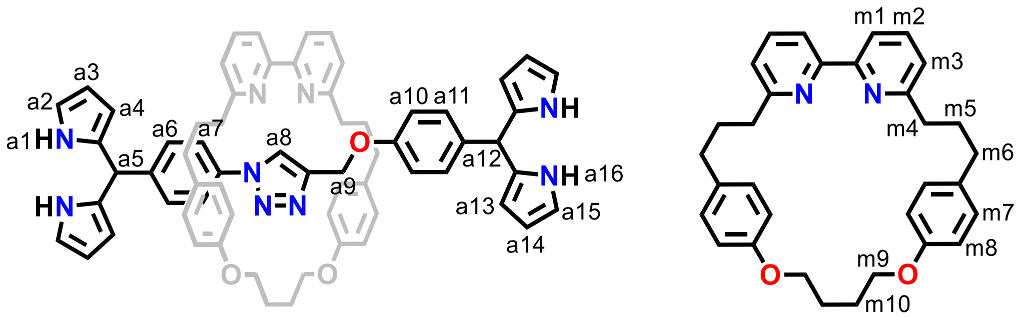

^{1}H NMR (500 MHz, $CDCl_3$, 300 K) δ (ppm): 9.35 (s, 1H, Ha8), 7.93 (b, 2H, Ha1), 7.82 (b, 2H, Ha16), 7.58 (t, 2H, ^3J = 7.8 Hz, Hm2), 7.41 (dd, 2H, ^3J = 7.8 Hz, ^4J = 0.7 Hz, Hm1), 7.30 (d, 2H, ^3J = 8.6 Hz, Ha7), 7.01 (dd, 2H, ^3J = 7.8 Hz, ^4J = 0.7 Hz, Hm3), 6.95 (d, 2H, ^3J = 8.6 Hz, Ha11), 6.81 (d, 2H, ^3J = 8.6 Hz, Ha6), 6.72–6.69 (m, 2H, Ha2), 6.66 (d, 2H,

^{3}J = 8.7 Hz, Ha10), 6.60–6.57 (m, 2H, Ha15), 6.43 (d, 4H, ^{3}J = 8.7 Hz, Hm8), 6.39 (d, 4H, ^{3}J = 8.7 Hz, Hm7), 6.17 (dd, 2H, ^{3}J = 6.4 Hz, ^{3}J = 2.8 Hz, a3), 6.11 (dd, 2H, ^{3}J = 6.4 Hz, ^{3}J = 2.8 Hz, a14), 5.87–5.83 (overlapping m, 4H, Ha4, Ha13), 5.33 (s, 1H, Ha5/Ha12), 5.31 (s, 1H, Ha5/Ha12), 4.61 (s, 2H, Ha9), 4.39–4.31 (m, 2H, Hm9), 4.17–4.09 (m, 2H, Hm9), 2.38–2.23 (overlapping m, 8H, Hm4, Hm6), 2.15–2.06 (m, 2H, Hm10), 2.06–1.93 (m, 2H, Hm10), 1.53–1.36 (m, 4H, Hm5)

^{13}C NMR (125 MHz, $CDCl_3$, 300 K) δ (ppm): 162.8, 157.5, 157.2, 157.0, 143.0, 140.8, 136.8, 135.4, 133.9, 132.9, 132.8, 132.2, 129.0, 128.5, 128.1, 123.7, 121.6, 120.3, 119.9, 117.2, 117.0, 115.0, 114.9, 108.5, 108.3, 107.2, 106.9, 66.5, 61.2, 43.5, 43.1, 36.9, 34.8, 32.0, 24.8.

HRMS (ESI+, TOF) *m/z*: $[M+Na]^+$ calcd. for $C_{65}H_{63}N_9O_3Na^+$, 1040.4946; found, 1040.4752.

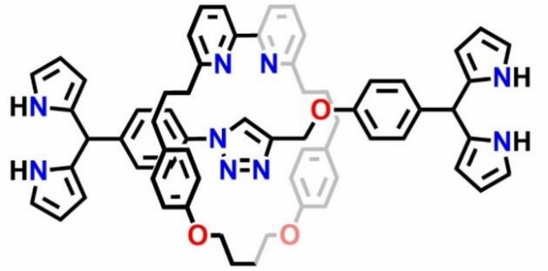

7

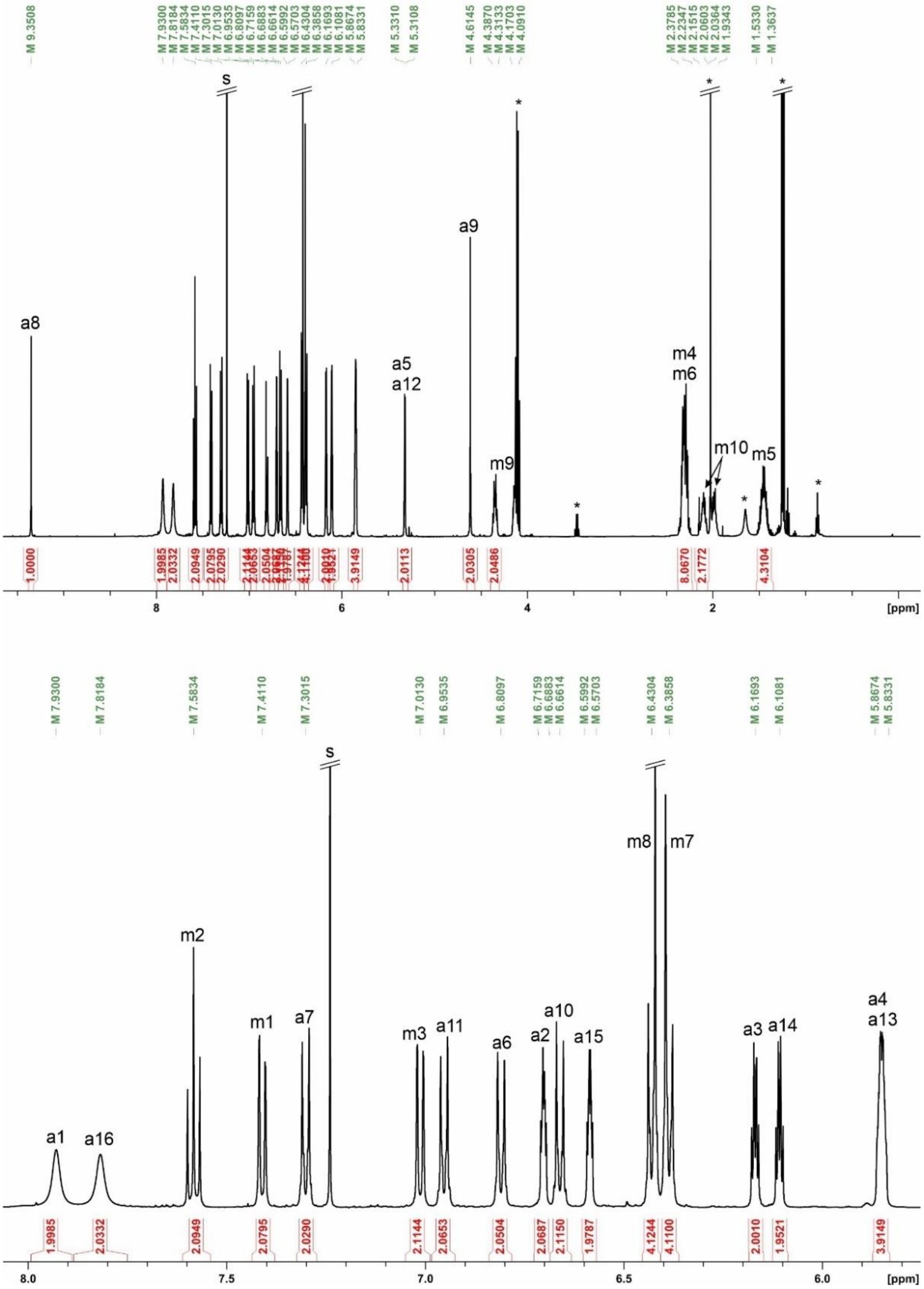

Figure S17. The ^{1}H NMR spectrum of **7** (500 MHz, $CDCl_3$, 300 K).

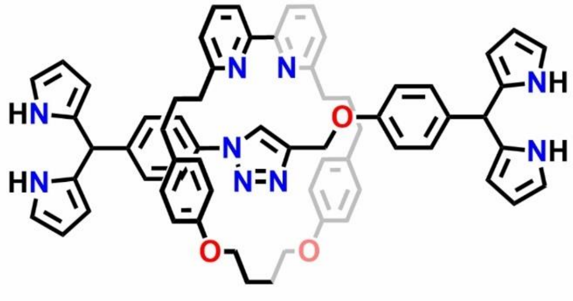

7

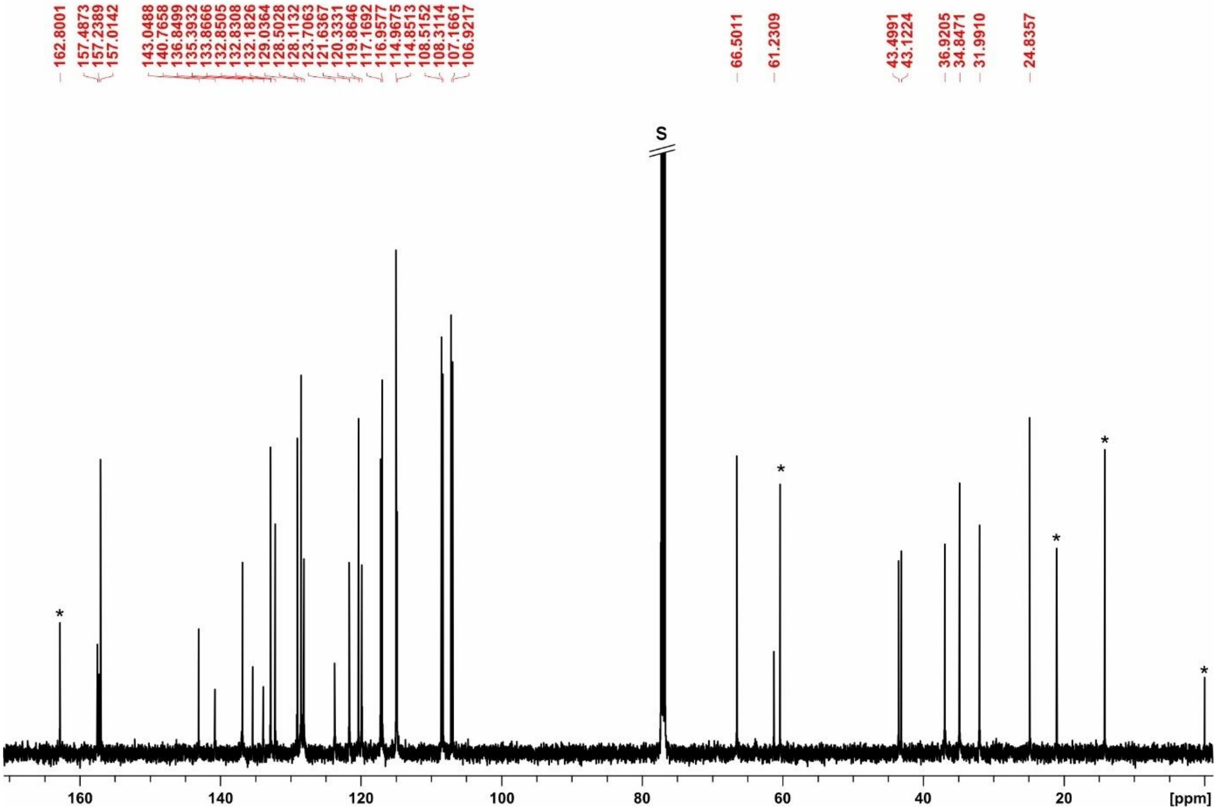

Figure S18. The ^{13}C NMR spectrum of **7** (125 MHz, $CDCl_3$, 300 K). Impurities (mainly EtOAc) were labeled with asterisks.

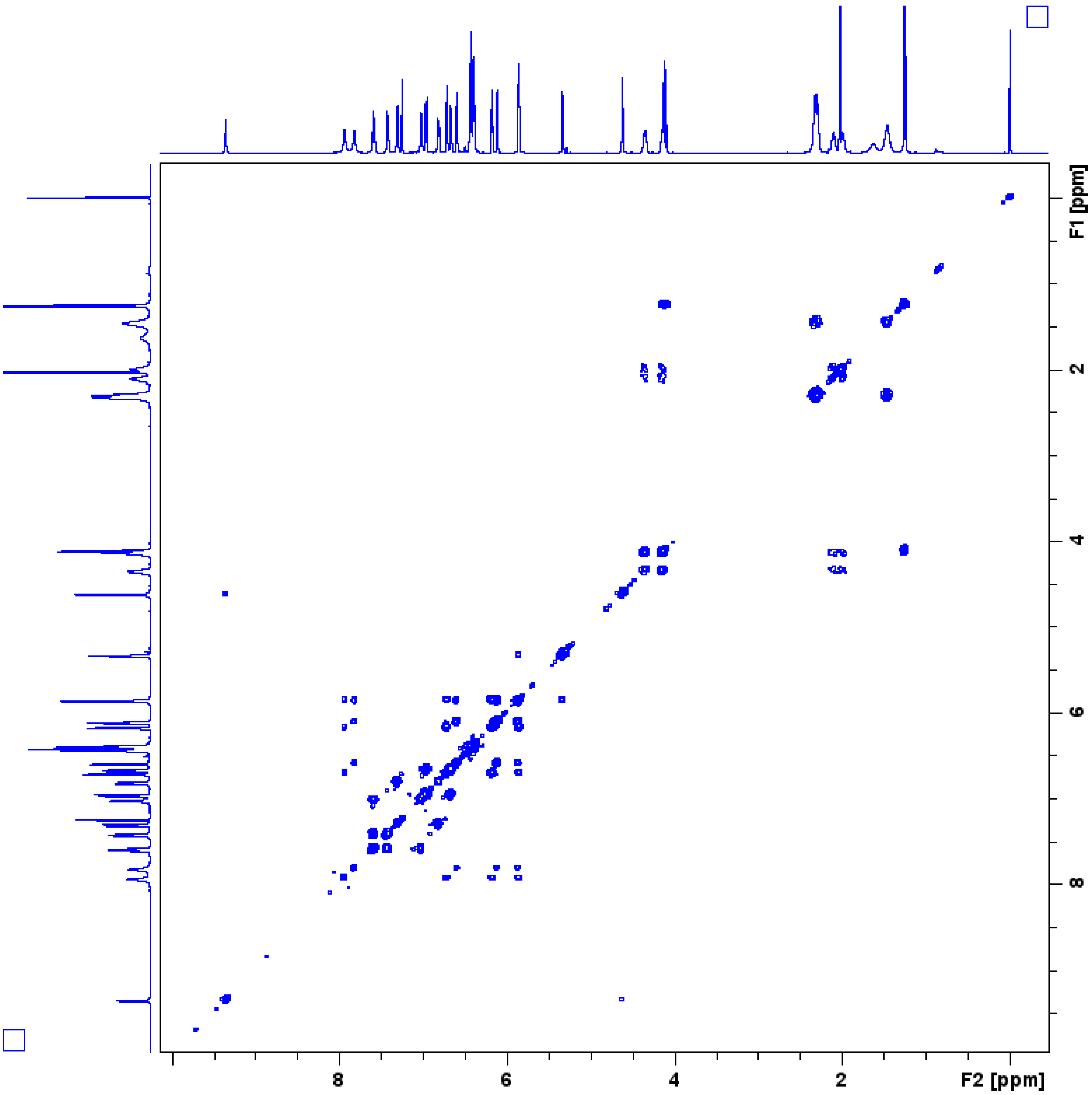

Figure S19. The ^{1}H-^{1}H COSY NMR spectrum of **7** (500 MHz, $CDCl_3$, 300 K).

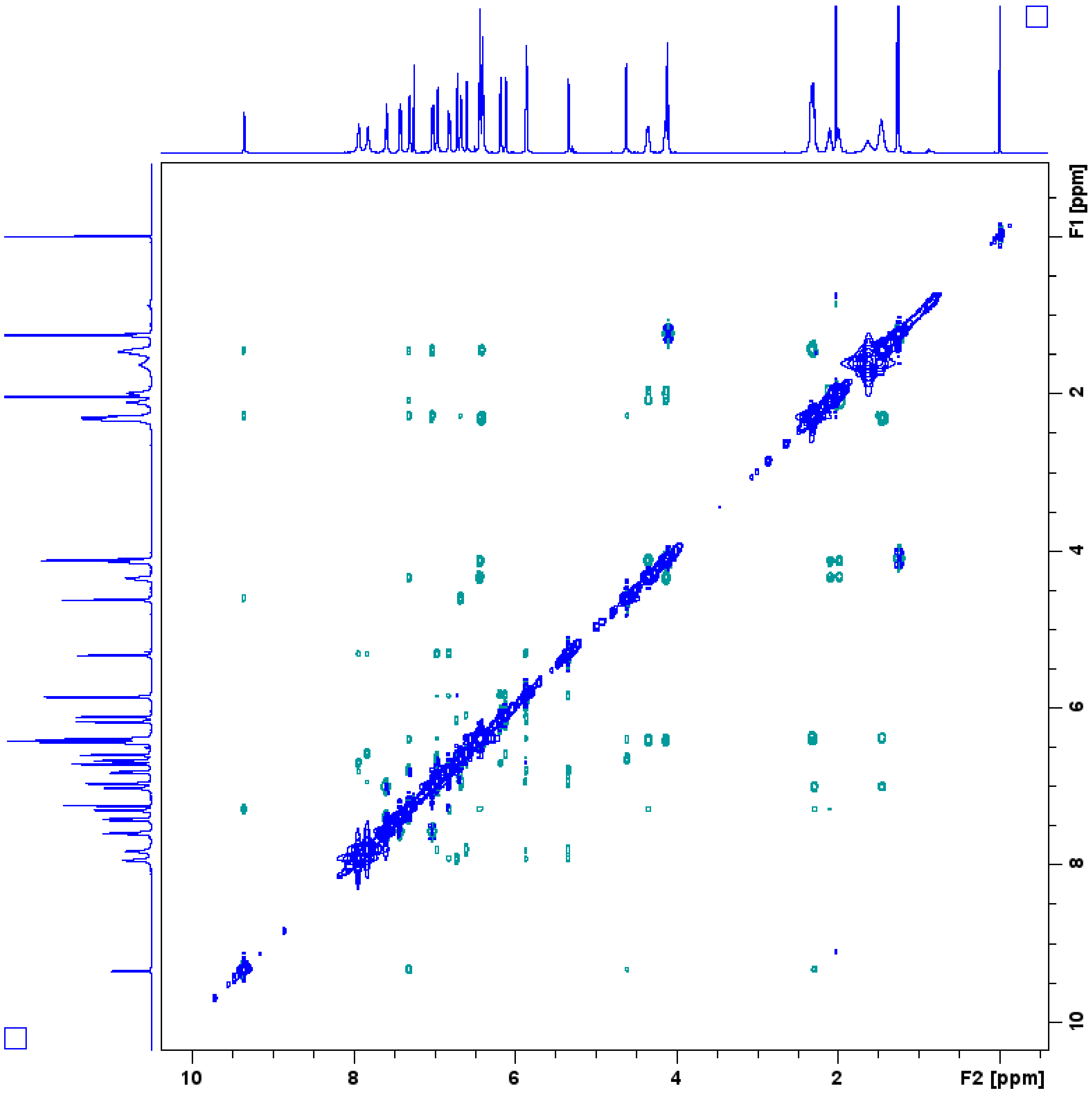

Figure S20. The ^{1}H-^{1}H NOESY NMR spectrum of **7** (500 MHz, $CDCl_3$, 300 K).

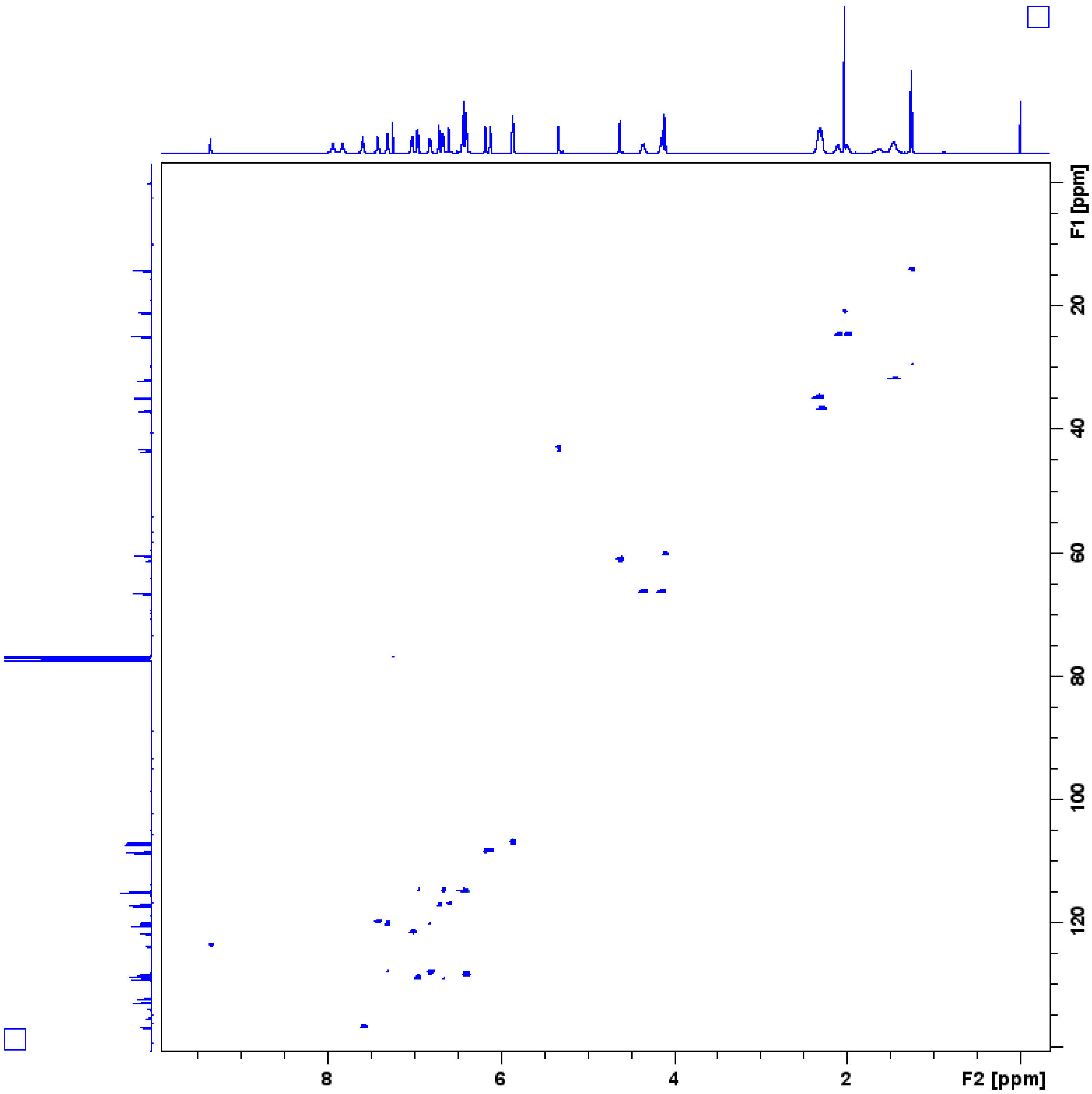

Figure S21. The ^{1}H-^{13}C HSQC NMR spectrum of **7** (500 MHz, $CDCl_3$, 300 K).

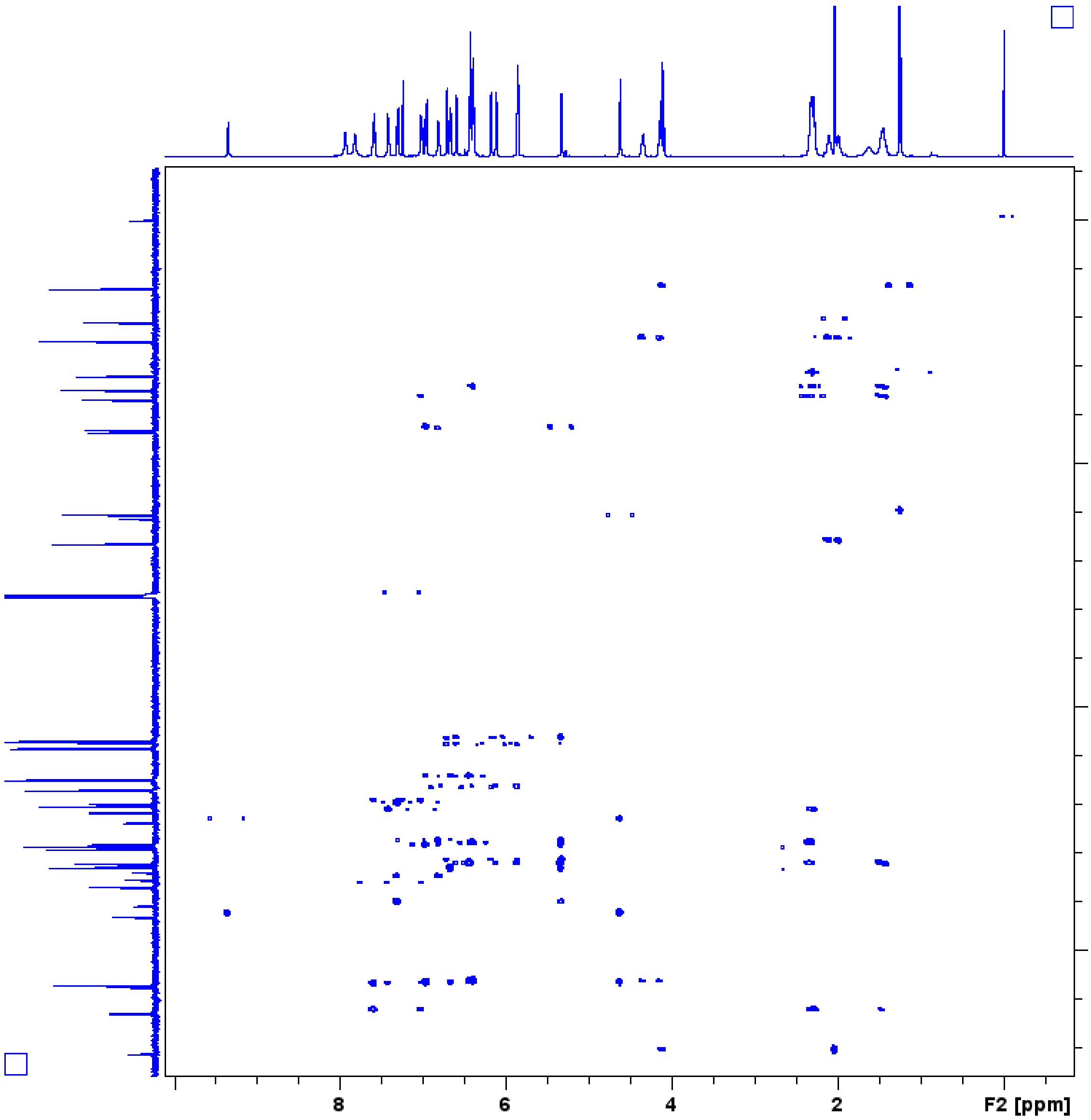

Figure S22. The ^{1}H-^{13}C HMBC NMR spectrum of **7** (500 MHz, $CDCl_3$, 300 K).

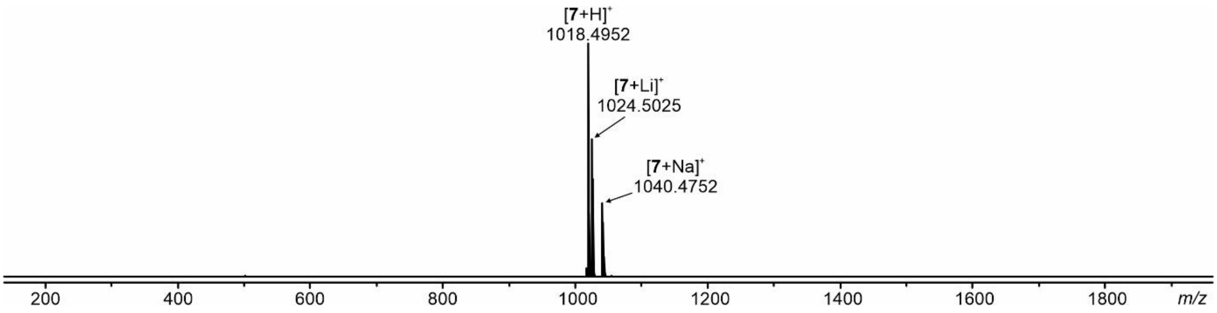

Figure S23. The ESI (TOF) mass spectrum of **7**.

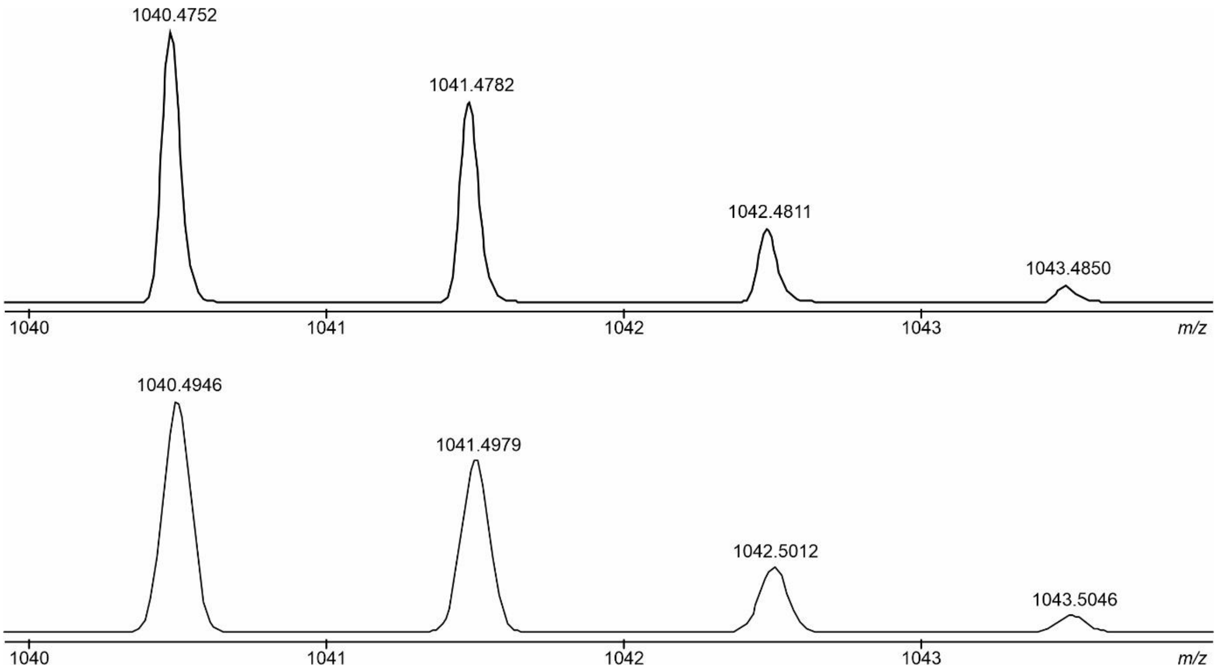

Figure S24. The ESI (TOF) mass spectrum of **7.** Top: experimental, bottom: simulated isotopic pattern.

Catenanes [2]cat^4 and [3]cat^4

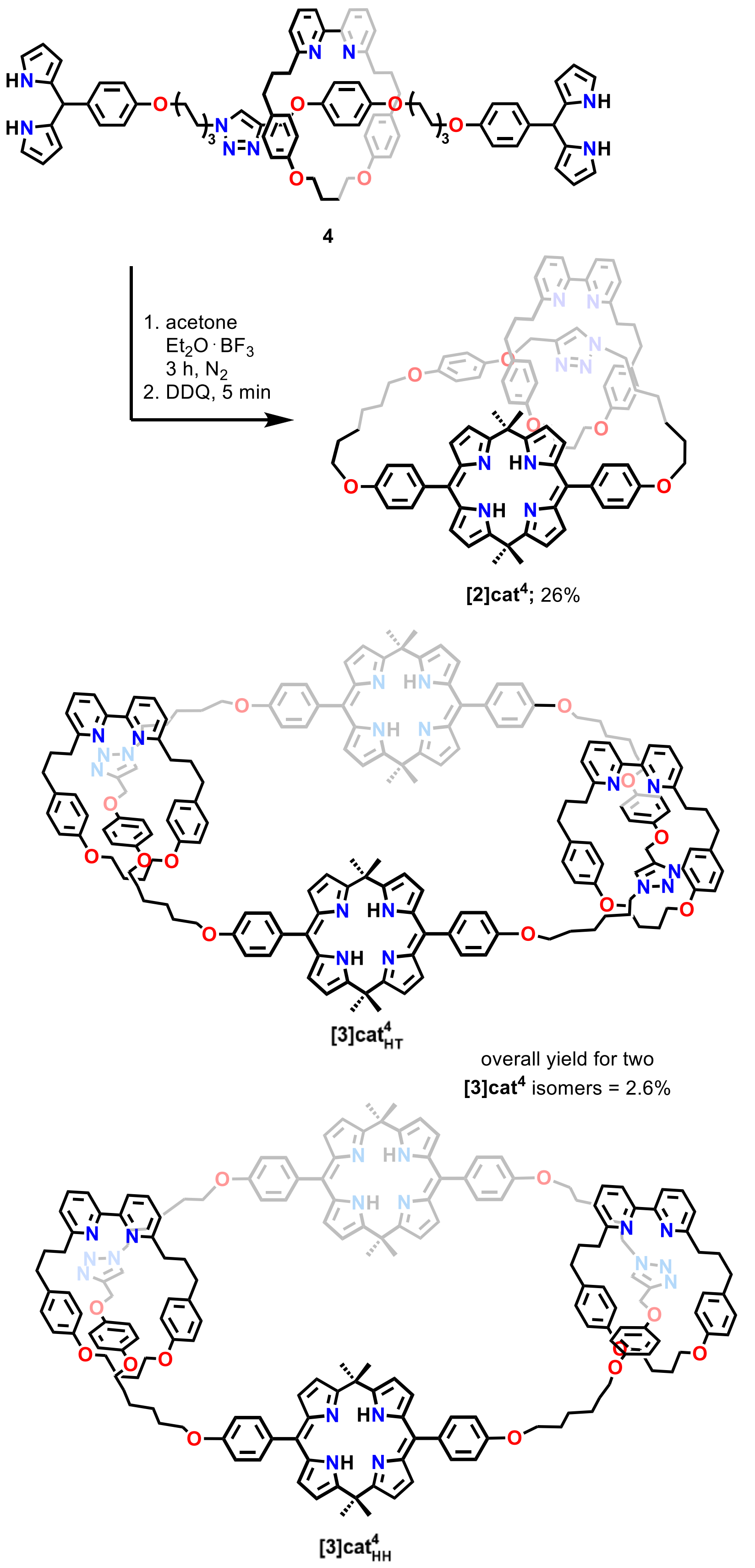

In a 50 mL round bottom flask, **4** (113 mg, 86.2 μmol) and acetone (30 mL) were introduced. The mixture was deoxygenated *via* nitrogen bubbling for 10 minutes. Then, $Et_2O{\cdot}BF_3$ (28 μL, 227 μmol) was added *via* the syringe. The mixture was then stirred for 3 hours under a nitrogen atmosphere. After this flask was opened, DDQ (70 mg, 308 μmol) was introduced, and the reaction was carried out for an additional 5 minutes. Then, the acid was quenched by adding TEA (0.5 mL), and the mixture was passed through a short column with deactivated aluminum oxide. Residues on the column were washed out with ethyl acetate. The solvent was removed under reduced pressure. The reddish oil was purified *via* column chromatography (DCM: hexane 1:1, with 5-40% ethyl acetate gradient) to provide **[2]cat^4^** (31 mg, 22.4 μmol, 26%) as a red crystalline solid, and mixture of isomers **[3]cat^4^** (3 mg, 1.1 μmol, 2.6%) as a orange crystalline solid. *Analytical data for **[2]cat^4^** are in agreement with published data.*[7]

*Analytical data for **[3]cat^4^**:*

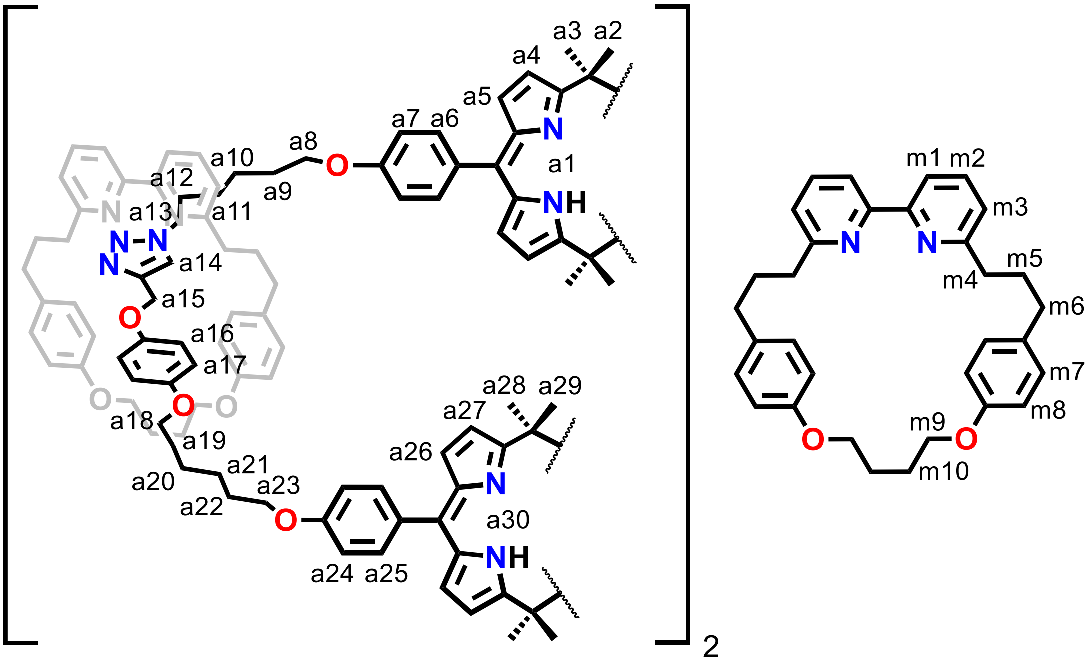

^1^H NMR (600 MHz, $CDCl_3$, 300 K) δ (ppm) for mixture of isomers **[3]cat^4^** (signals from HT and HH isomers overlapping) 14.19 (b, Ha1, Ha30), 8.00–7.98 (2 x s, Ha14), 7.57–7.51 (2 x t, Hm2), 7.35–7.26 (m, Ha6, Ha25, Hm1), 7.07–7.04 (2 x dd, Hm3), 6.84–6.80 (m, Ha24), 6.80–6.68 (m, Ha7, Ha16, Ha17, Hm7), 6.65–6.60 (m, Hm8), 6.37–6.31 (m, Ha5, Ha26), 6.23–6.18 (m, Ha4, Ha27), 4.93 (2 x s, Ha15), 4.24–4.15 (m, Hm9), 4.02–3.93 (m, Hm9), 3.88 (m/t, Ha23), 3.80 (m/t, Ha18), 3.66–3.61 (m, Ha8), 3.39–3.33 (m, Ha13), 2.60–2.40 (m, Hm4, Hm6), 2.08–1.81 (m, Ha2, Ha3, Ha28, Ha29, Hm10), 1.81–1.62 (m, Ha19, Ha22, Hm5), 1.43–1.16 (m, Ha9, Ha20, Ha21), 0.97–0.76 (m, Ha10, Ha12), 0.76–0.63 (m, Ha11).

^{13}**C NMR** (150 MHz, $CDCl_3$, 300 K) δ (ppm) for mixture of isomers **[3]cat^{4}**:164.9, 162.5, 159.7, 157.7 (2 overlapping signals), 157.4, 153.3, 152.3, 142.9, 140.4 (2 overlapping signals), 136.7, 133.3, 132.23, 132.18, 129.3 (2 overlapping signals), 128.3 (2 overlapping signals), 124.1, 121.3, 120.2, 115.6, 115.4, 114.9, 113.9 (2 overlapping signals), 113.5, 113.4, 68.3, 67.8, 67.6, 66.5, 62.3, 49.5, 38.2, 36.9, 34.6, 31.8, 30.9, 29.1, 29.02, 28.8 (2 overlapping signals), 28.5, 25.7, 25.6, 25.0, 24.8.

HRMS (ESI+, TOF): *m/z*: $[M+2H]^{2+}$ calcd. for $C_{178}H_{192}N_{18}O_{12}^{2+}$, 1387.7511; found, 1387.7544.

Attempted **[3]cat$^{4}_{HT}$** from **[3]cat$^{4}_{HH}$** separation:

HPLC resolutions were performed using the LaChrom Merck Hitachi I-7420 system with an eluent flow rate of 2 mL/min (DCM:*n*-hexane from 10:90 to 50:50) and Phenomenex Chirex 3010 column, Phenomenex Chirex 3014 column, and Phenomenex Chirex 3022 column. The isomers' separation via selective crystallisation was also tried, but did not result in the resolution of isomers due to their similar solubility in the tested organic solvents (DCM, MeOH, MeCN, *n*-hexane).

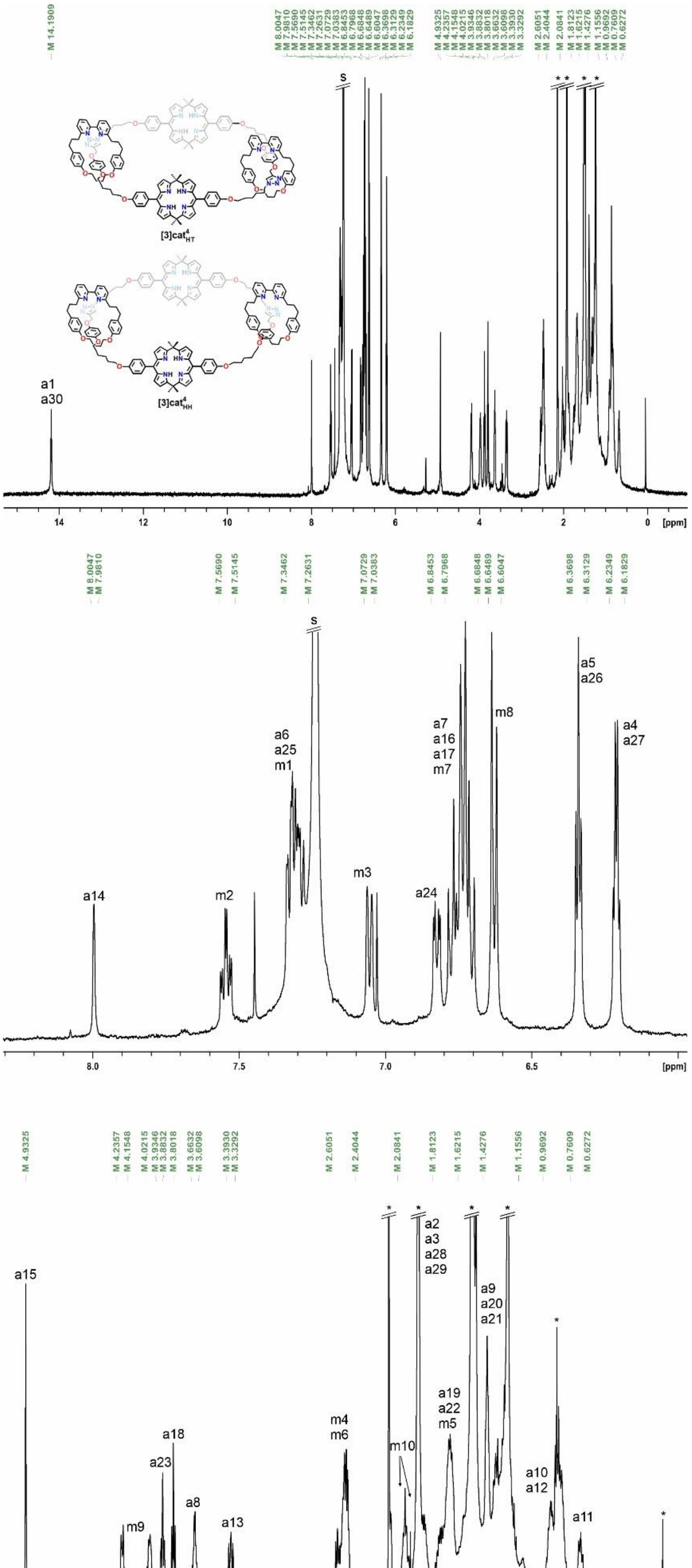

Figure S25. The ^{1}H NMR spectrum of mixture of isomers **[3]cat⁴** (600 MHz, $CDCl_3$, 300 K).

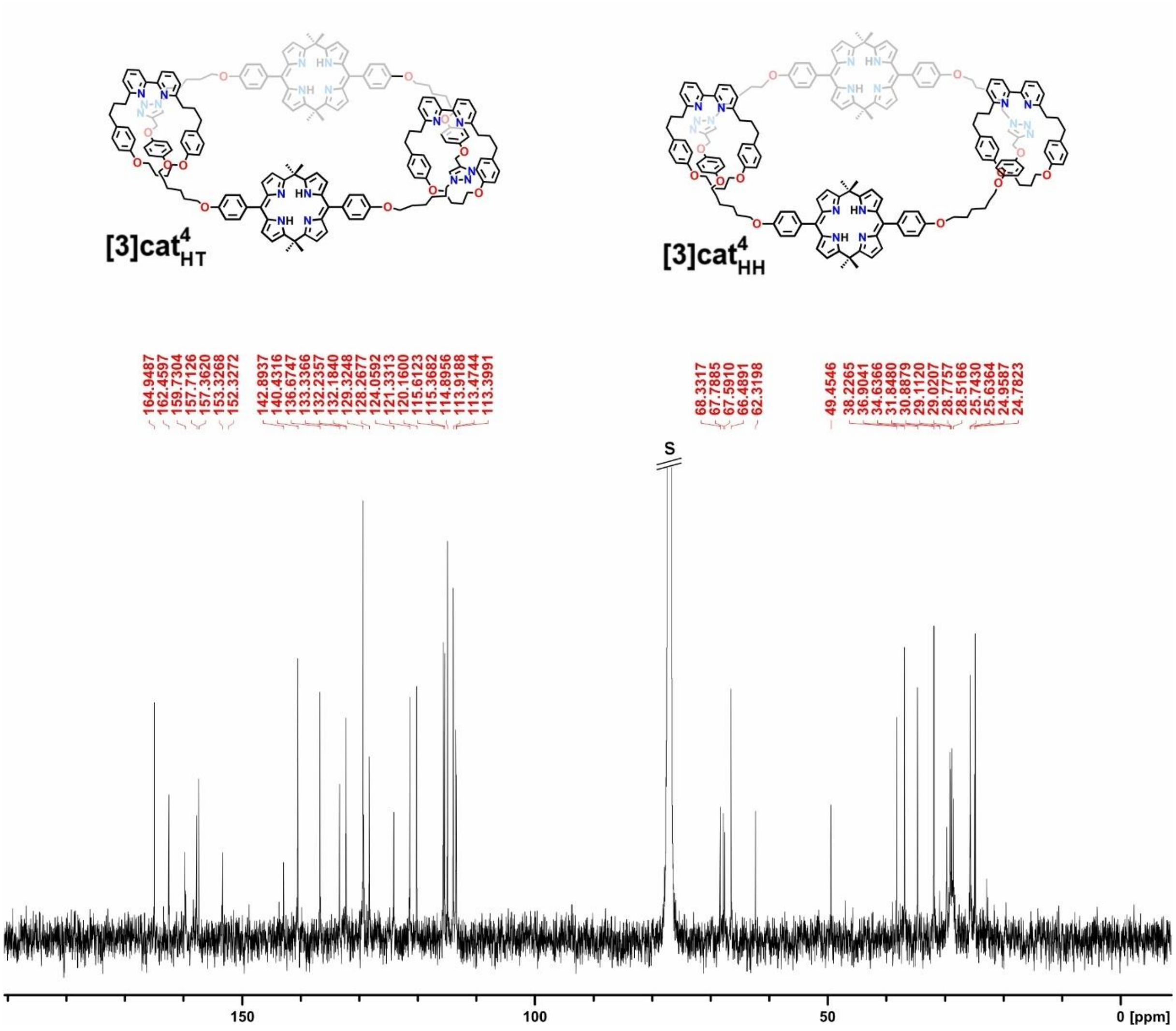

Figure S26. The ^{13}C NMR spectrum of mixture of isomers **[3]cat^{4}** (150 MHz, $CDCl_3$, 300 K).

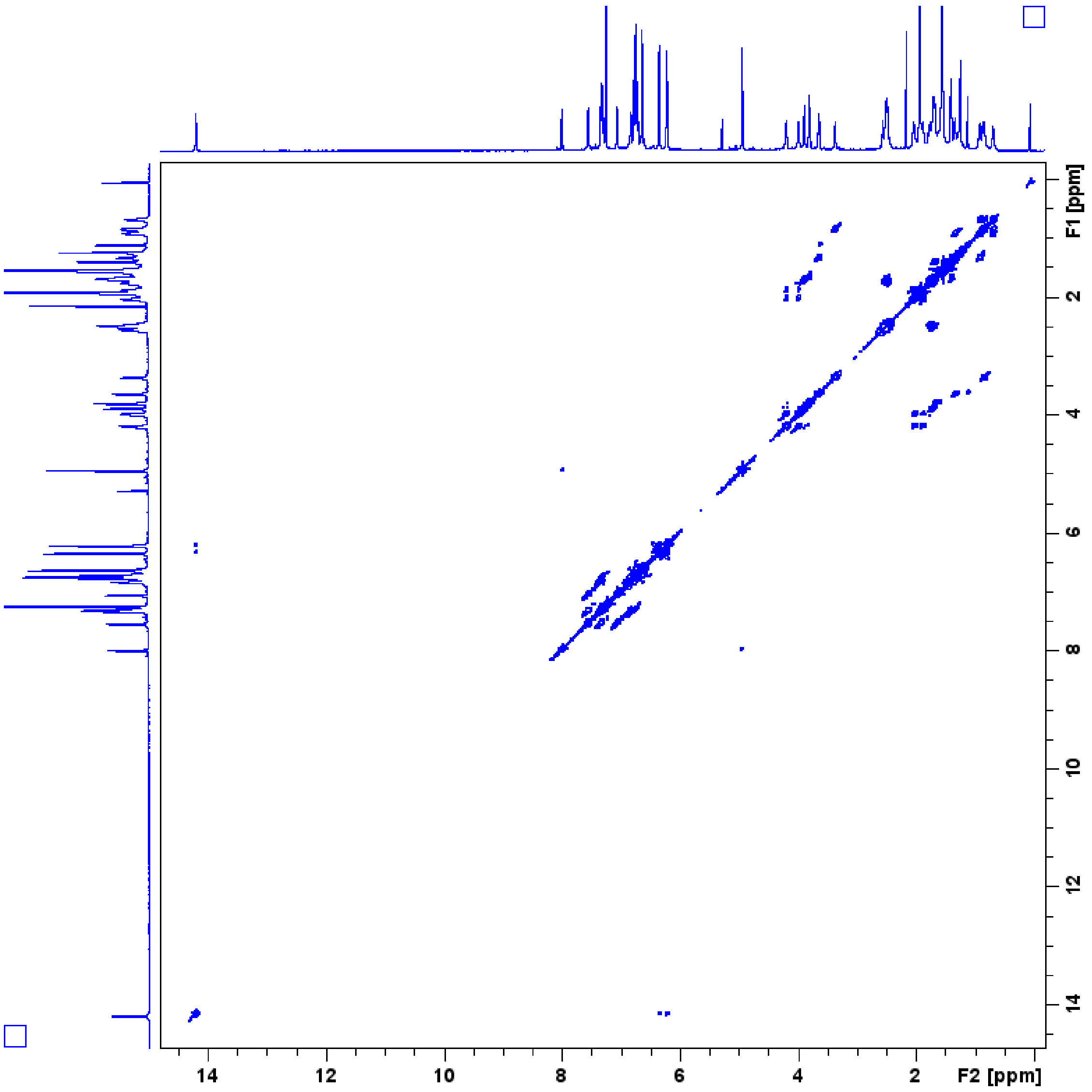

Figure S27. The ^{1}H-^{1}H COSY NMR spectrum of mixture of isomers **[3]cat[4]** (600 MHz, $CDCl_3$, 300 K).

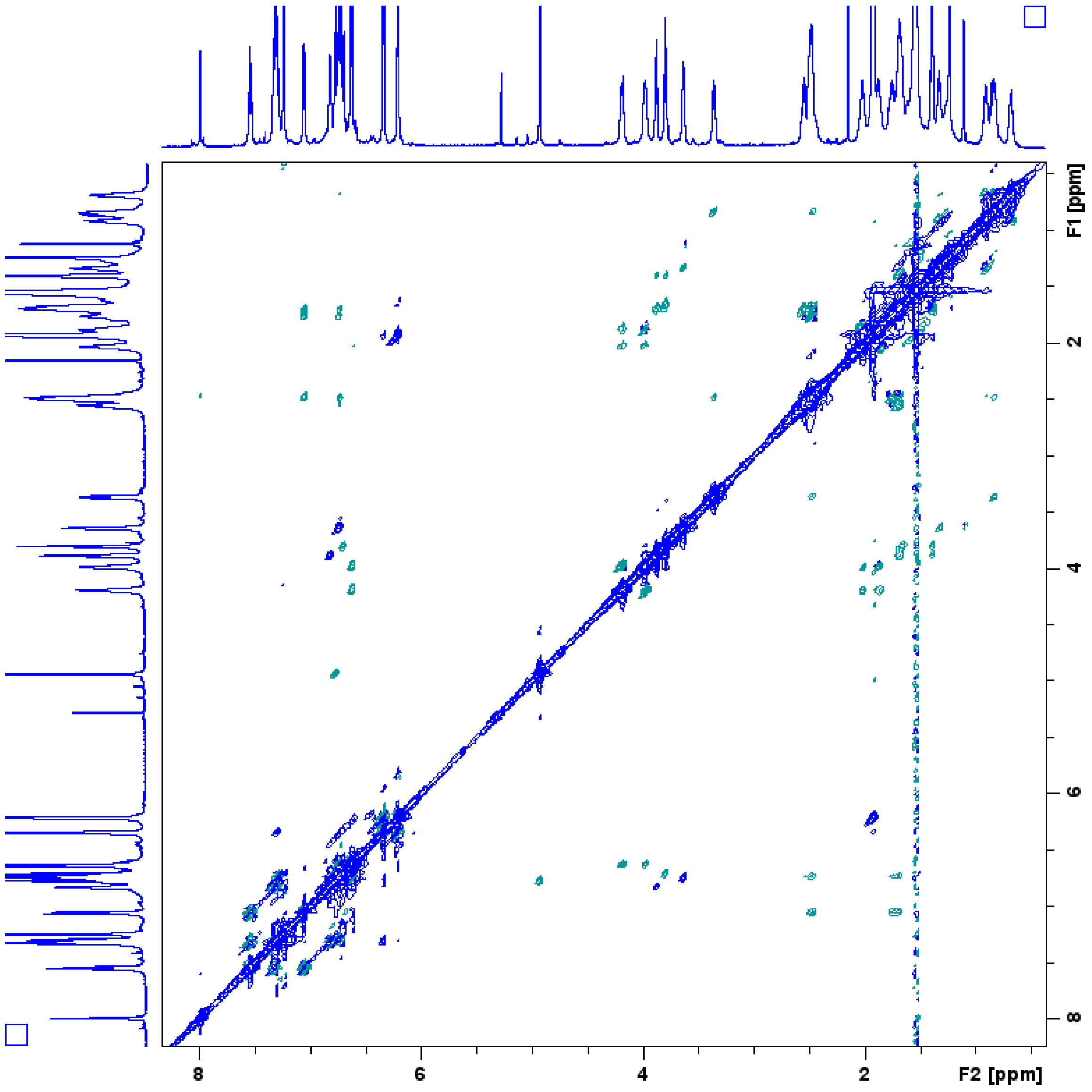

Figure S28. The ^{1}H-^{1}H NOESY NMR spectrum of mixture of isomers **[3]cat^{4}** (600 MHz, $CDCl_3$, 300 K).

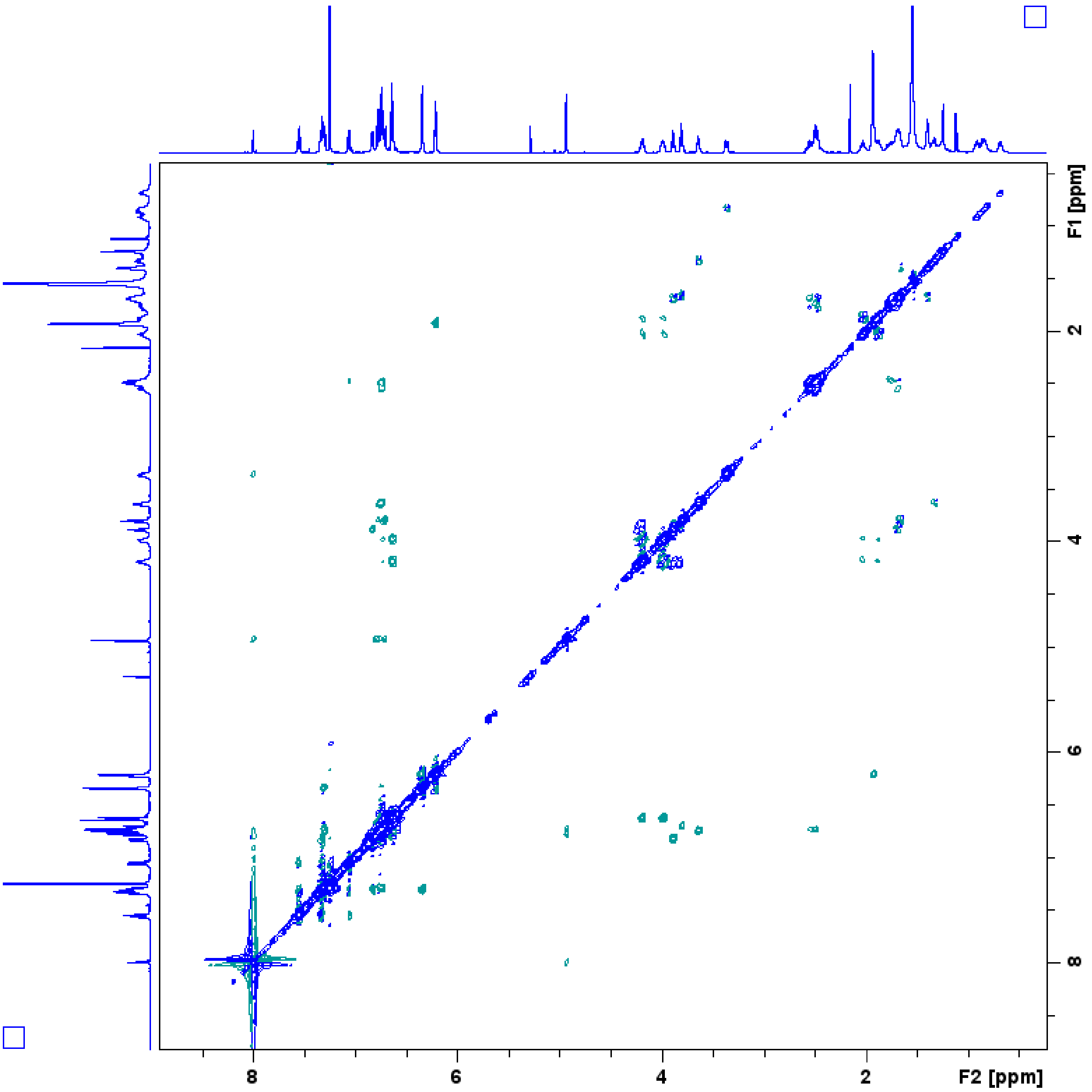

Figure S29. The ^{1}H-^{1}H ROESY NMR spectrum of mixture of isomers **[3]cat^{4}** (600 MHz, $CDCl_3$, 300 K).

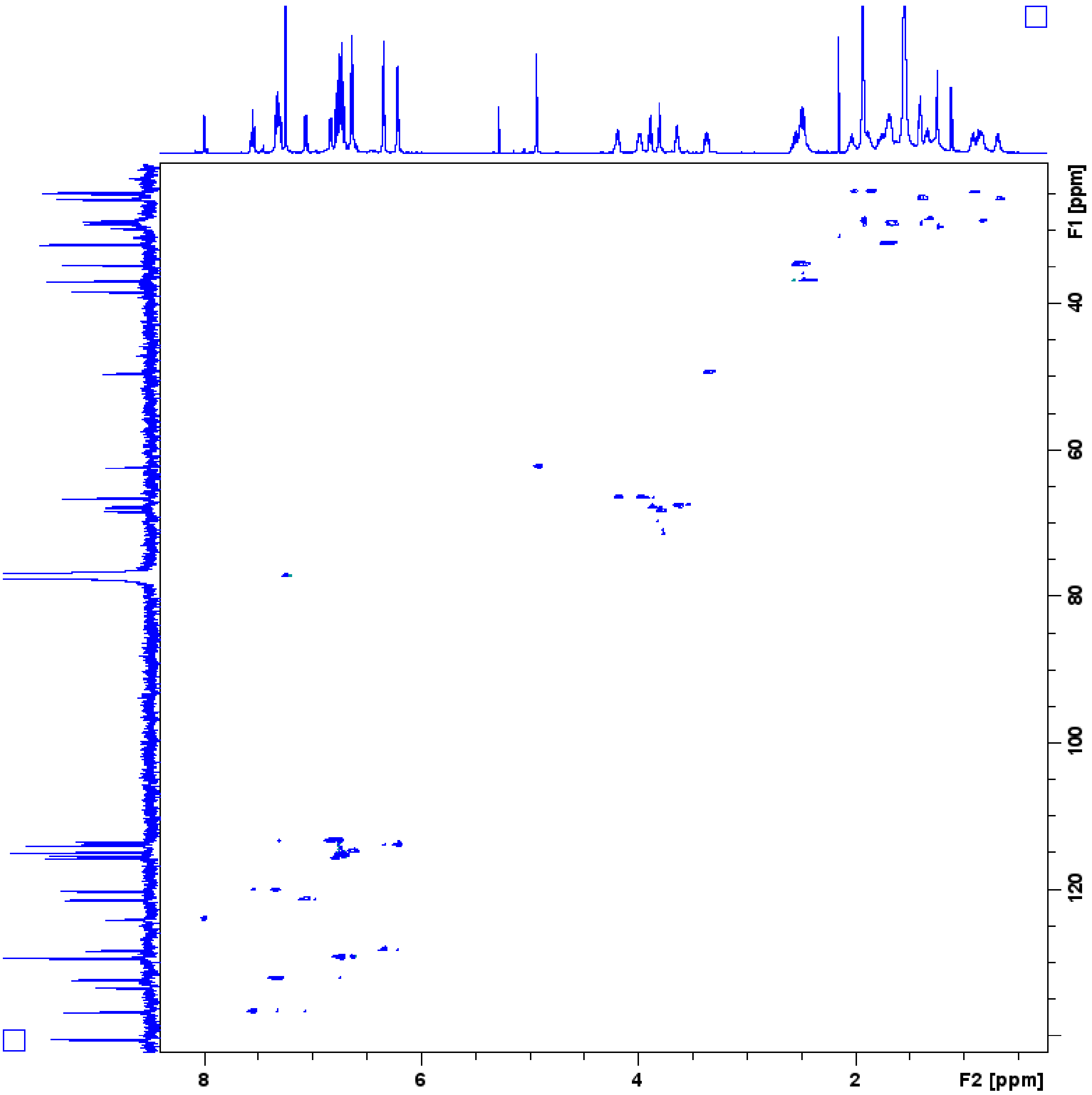

Figure S30. The ^{1}H-^{13}C HSQC NMR spectrum of mixture of isomers **[3]cat^{4}** (600 MHz, $CDCl_3$, 300 K).

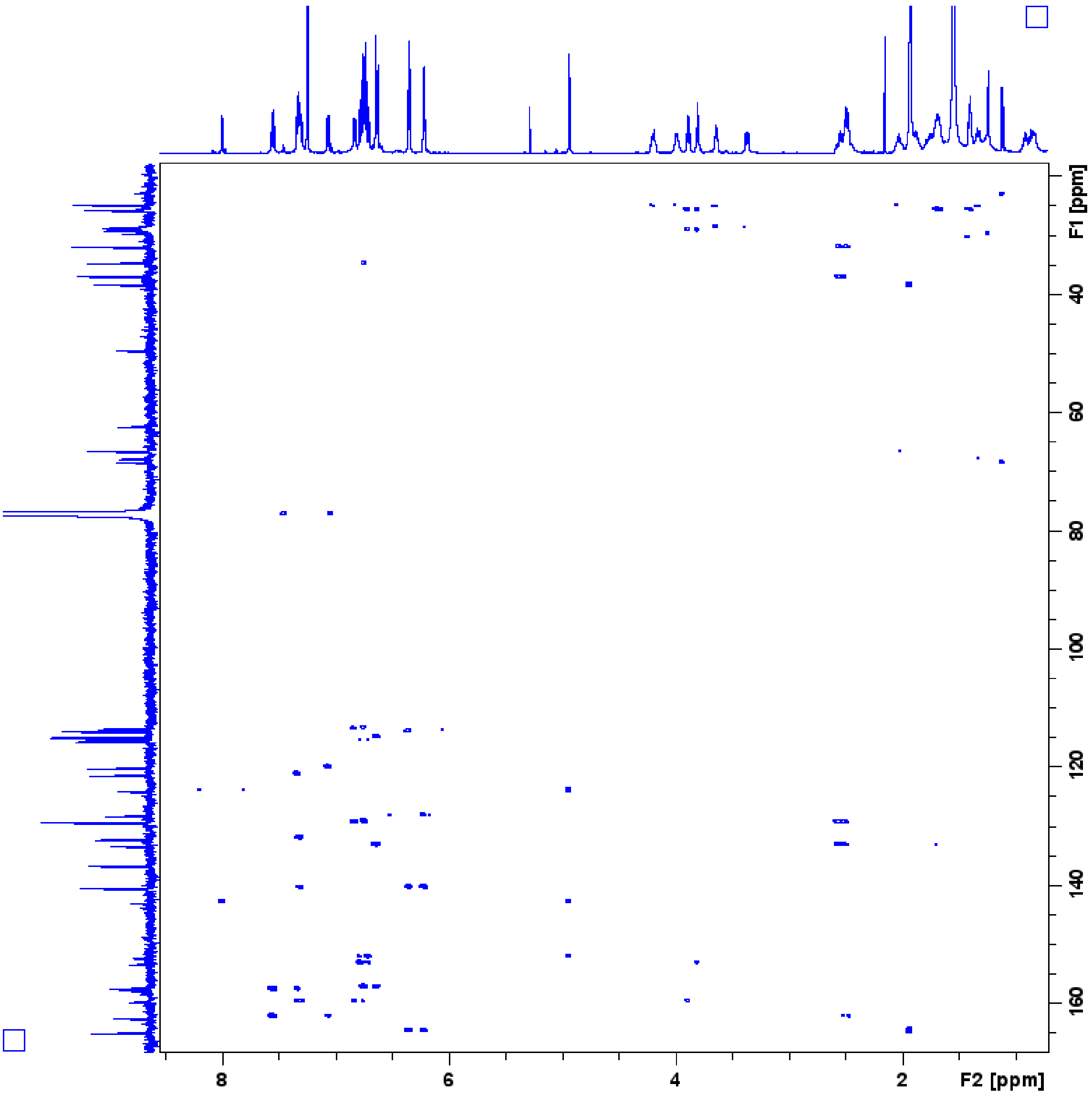

Figure S31. The ^{1}H-^{13}C HMBC NMR spectrum of mixture of isomers **[3]cat^{4}** (600 MHz, $CDCl_3$, 300 K).

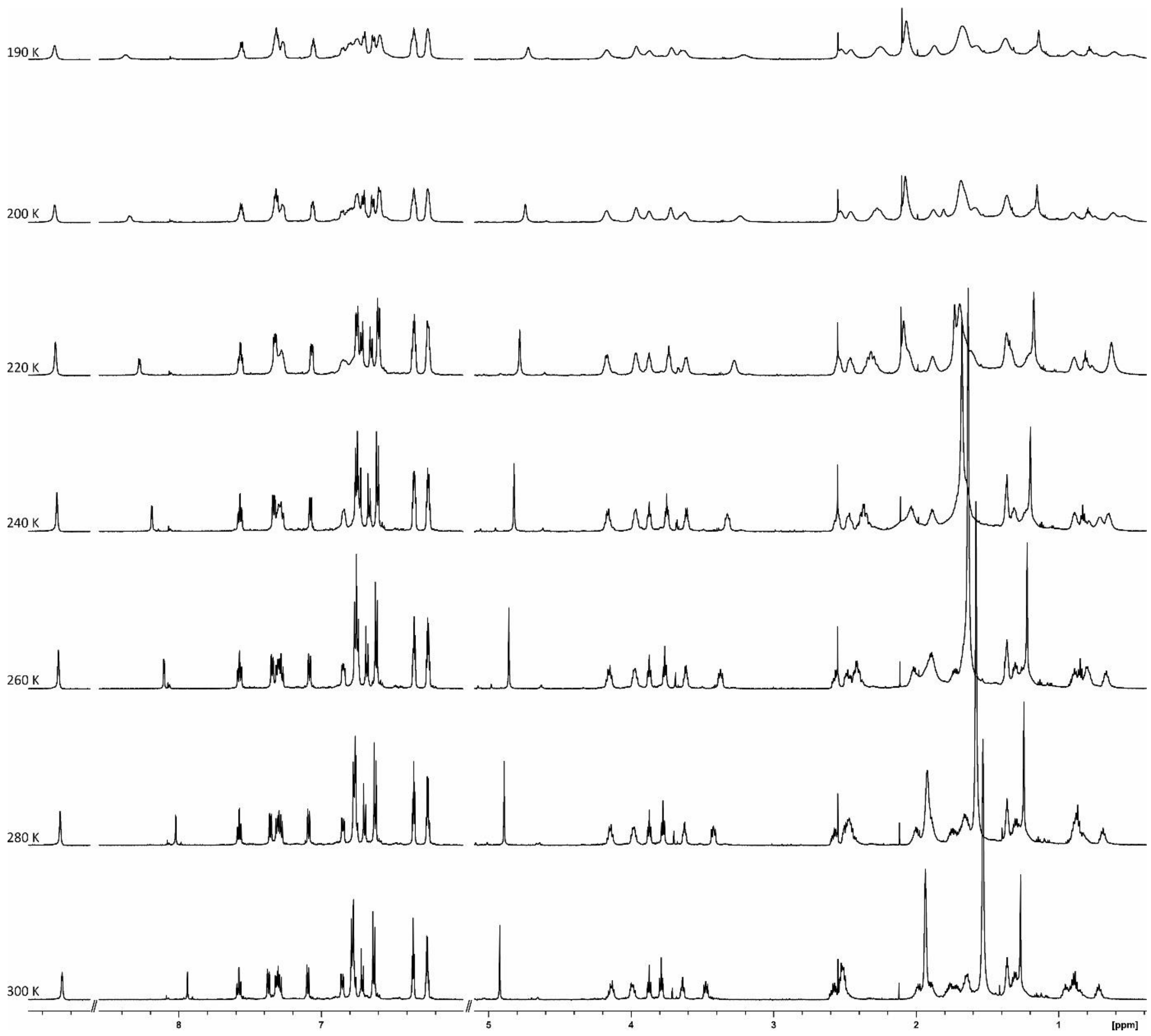

Figure S32. ^{1}H NMR spectra of mixture of isomers **[3]cat^{4}** recorded in the 300–190 K temperature range (600 MHz, CD_2Cl_2).

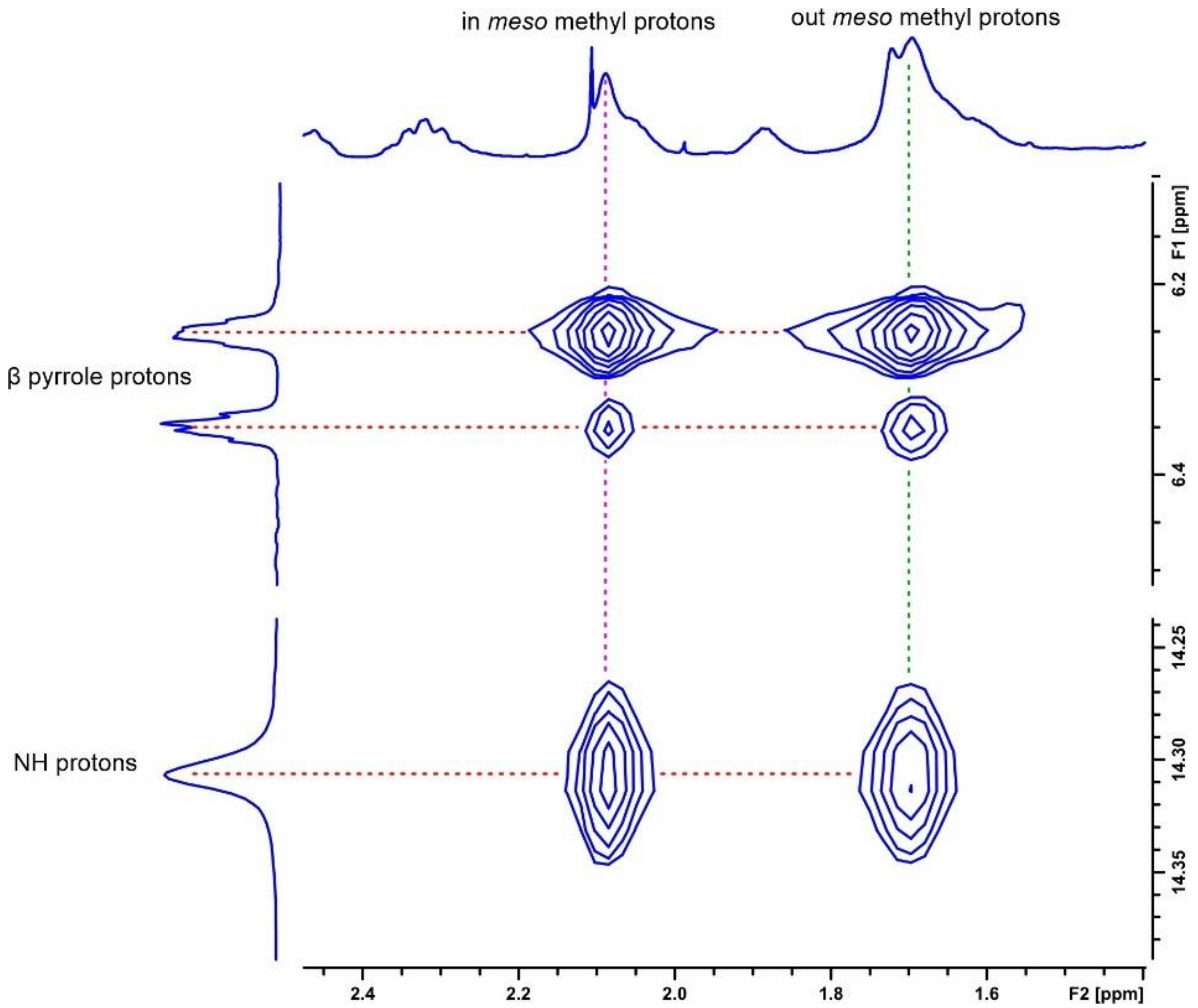

Figure S33. The ^{1}H-^{1}H NOESY NMR spectrum of mixture od isomers **[3]cat^{4}**. Correlation between the NH, β-pyrrolic, and *meso*-methyl protons were marked in green, purple and red dash lines (600 MHz, CD_2Cl_2, 220 K).

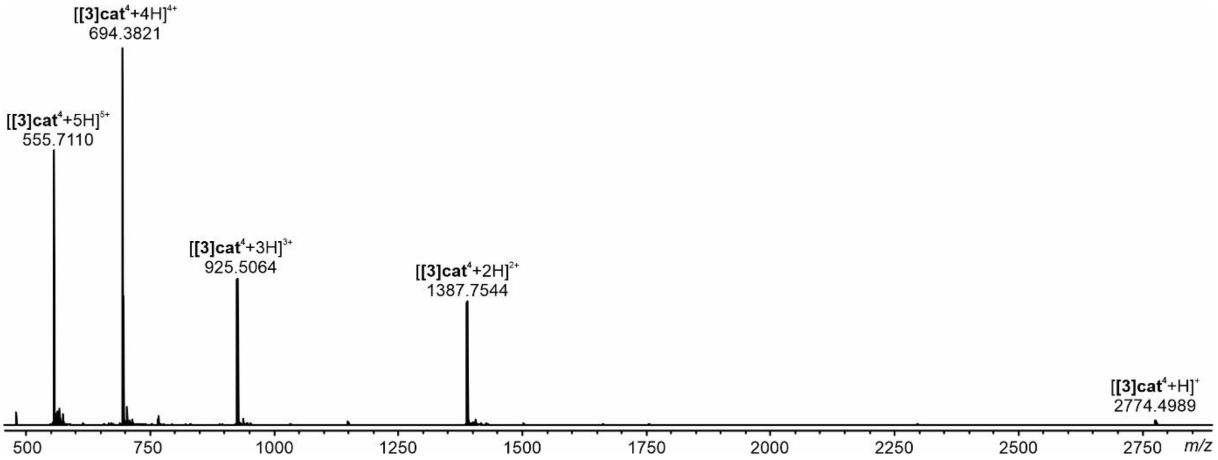

Figure S34. The ESI (TOF) mass spectrum of isomers of **[3]cat^{4}**.

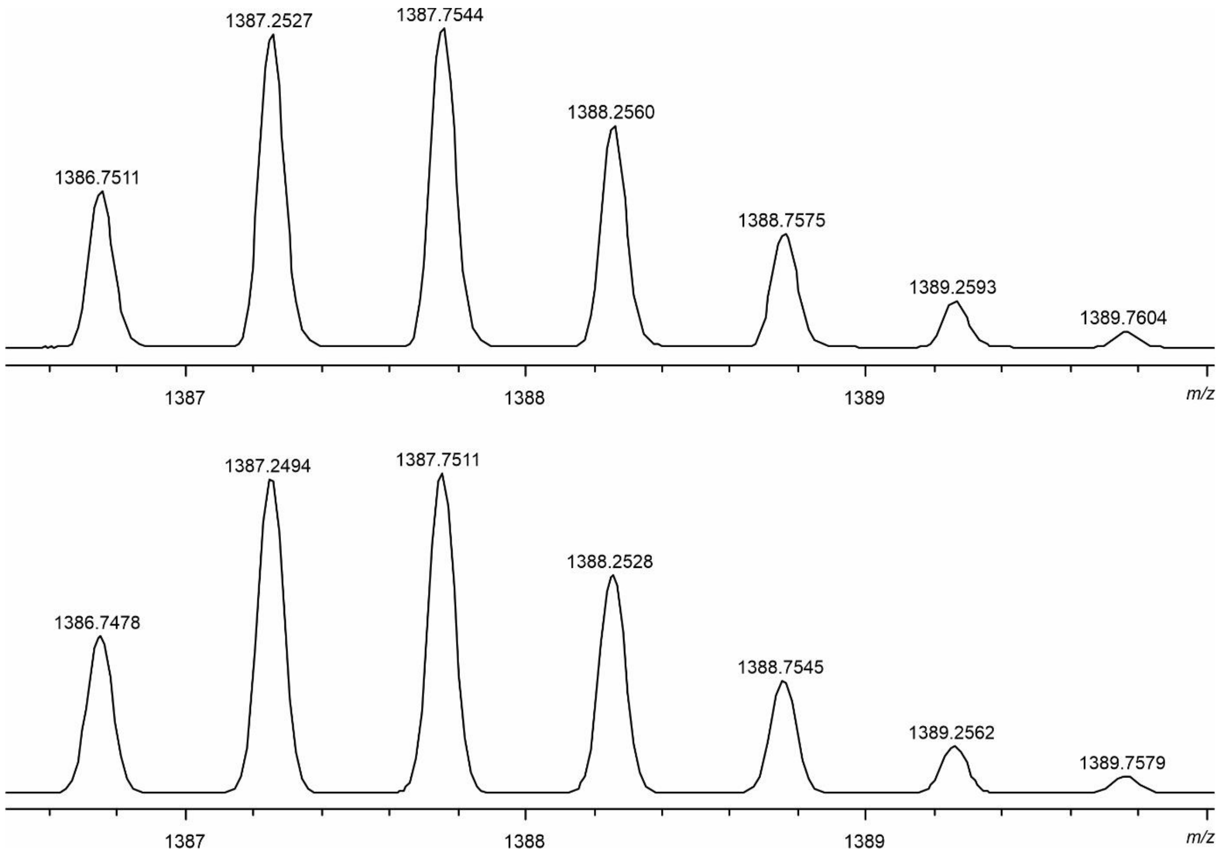

Figure S35. The ESI (TOF) mass spectrum of isomers of **[3]cat[4]**. Top: experimental, bottom: simulated isotopic pattern.

Catenane [3]cat[5]

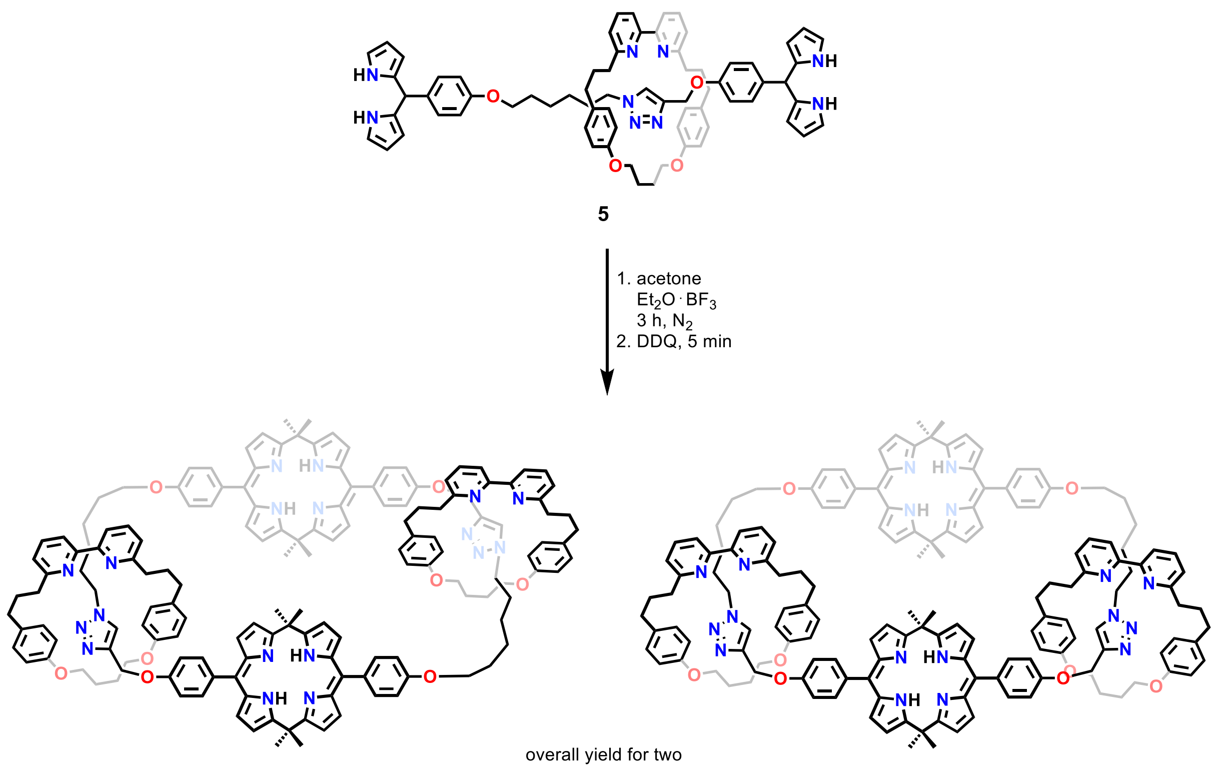

[3]cat$^5_{HT}$ [3]cat$^5_{HH}$

In a 50 mL round bottom flask, **5** (96 mg, 86.2 μmol) and acetone (30 mL) were introduced. The mixture was deoxygenated *via* nitrogen bubbling under a sealed flask for 10 minutes. Then, $Et_2O \cdot BF_3$ (28 μL, 227 μmol) was added *via* the syringe. The mixture was then stirred for 3 hours under a nitrogen atmosphere. After this time, the flask was opened, DDQ (70 mg, 308 μmol) was introduced, and the reaction was carried out for an additional 5 minutes. Then, the acid was quenched by adding TEA (0.5 mL), and the mixture was passed through a short column with deactivated aluminum oxide. Residues on the column were washed out with ethyl acetate. The solvent was removed under reduced pressure. The reddish oil was purified *via* flash chromatography (DCM with 0-20% ethyl acetate gradient) and then recrystallized with DCM:diisopropyl ether to provide the mixture of isomers of **[3]cat[5]** (13 mg, 5.4 μmol, 12%) as an orange crystalline solid. Isomers could be only partially separated *via* HPLC chromatography.

^{13}C NMR (150 MHz, $CDCl_3$, 300 K) for mixture of isomers, δ (ppm): 165.04 and 165.02, 164.98 and 164.97, 162.5, 159.77 and 159.75, 158.97 and 158.96, 157.6, 157.4, 142.4, 140.47, 140.44, 140.42, 140.40, 140.36, 140.35, 140.25, 140.22, 136.7, 133.2, 132.2 (2 overlapping signals), 129.6, 129.3, 129.1, 128.17 and 128.15, 124.7, 121.4, 120.2, 114.8, 113.91 and 113.89. 113.7, 113.40 and 113.37, 67.5, 66.4, 61.9, 49.3, 38.26, 38.23, 38.19, 36.9, 34.7, 31.8,

28.63 and 28.62, 28.33 and 28.32, 25.6, 24.9, 24.7. **HRMS** (ESI+, TOF) *m/z*: $[M+2H]^{2+}$ calcd. for $C_{154}H_{160}N_{18}O_8^{2+}$, 1195.1344; found, 1195.1407.

Attempted **[3]cat$^{5}_{HT}$** from **[3]cat$^{5}_{HH}$** separation:

HPLC resolution was performed using the LaChrom Merck Hitachi I-7420 system using the Phenomenex Chirex 3014 column (25 cm x 0.46 cm) with an eluent flow rate of 2 mL/min (hexane:dichloromethane 75:25). The samples of isomers with ca. 85% purity were obtained.

*Analytical data for **[3]cat$^{5}_{HT}$**:*

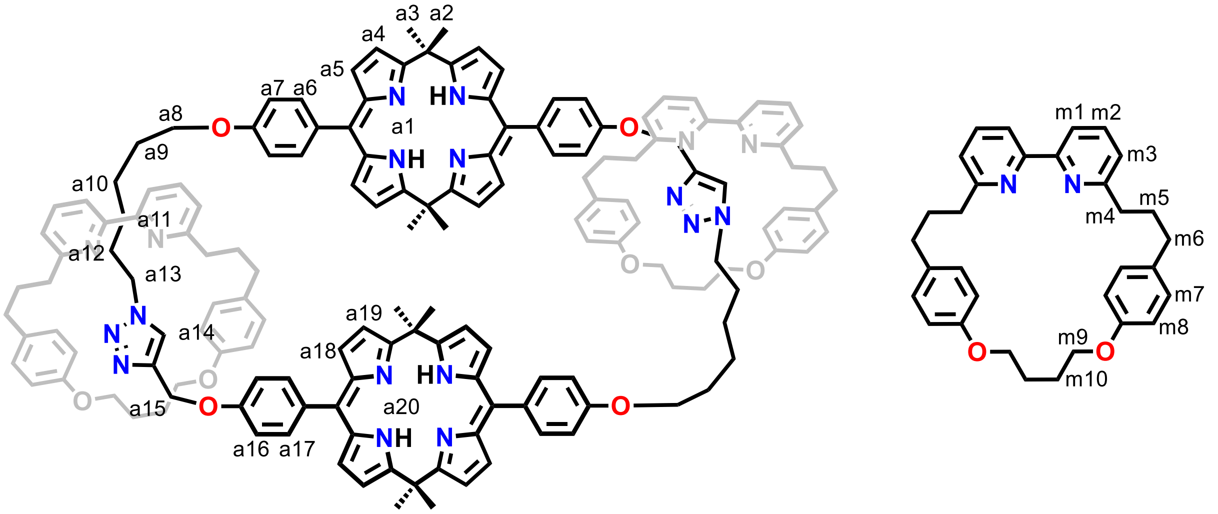

^{1}**H NMR** (600 MHz, $CDCl_3$, 300 K) δ (ppm): 14.19 (s, 2H, Ha1), 14.18 (s, 2H, Ha20), 8.33 (s, 2H, Ha14), 7.53 (t, 4H, ^3J = 7.7 Hz, Hm2), 7.31–7.26 (overlapping m, 8H, Hm1, Ha6), 7.25–7.23 (overlapping m, 4H, Ha17), 7.04 (d, 4H, ^3J = 7.7 Hz, Hm3), 6.85–6.79 (overlapping m, 4H, Ha16), 6.72–6.67 (overlapping m, 12H, Ha7, Hm8), 6.63 (d, 8H, ^3J = 8.4 Hz, Hm7), 6.32 (d, 4H, ^3J = 4.1 Hz, Ha5), 6.22 (d, 4H, ^3J = 4.1 Hz, Ha18), 6.20 (d, 4H, ^3J = 4.1 Hz, Ha4), 6.14 (d, 4H, ^3J = 4.1 Hz, Ha19), 4.96 (s, 4H, Ha15), 4.27–4.22 (m, 4H, Hm9), 4.03–3.97 (m, 4H, Hm9), 3.61 (t, 4H, ^3J = 6.2 Hz, Ha8), 3.26 (t, 4H, ^3J = 8.3 Hz, Ha13), 2.58–2.50 (m, 4H, Hm6), 2.50–2.37 (overlapping, 12H, Hm4, Hm6), 2.11–2.02 (overlapping m, 4H, Hm10), 2.02–1.82 (overlapping, 28H, Ha2, Ha3, Hm10), 1.82–1.72 (m, 4H, Hm5), 1.72–1.62 (m, 4H, Hm5), 1.36–1.19 (overlapping m, 4H, Ha9), 0.94–0.73 (overlapping, 8H, Ha10, Ha12), 0.73–0.64 (m, 4H, Ha11).

Analytical data for ***[3]cat***$^{5}_{HH}$

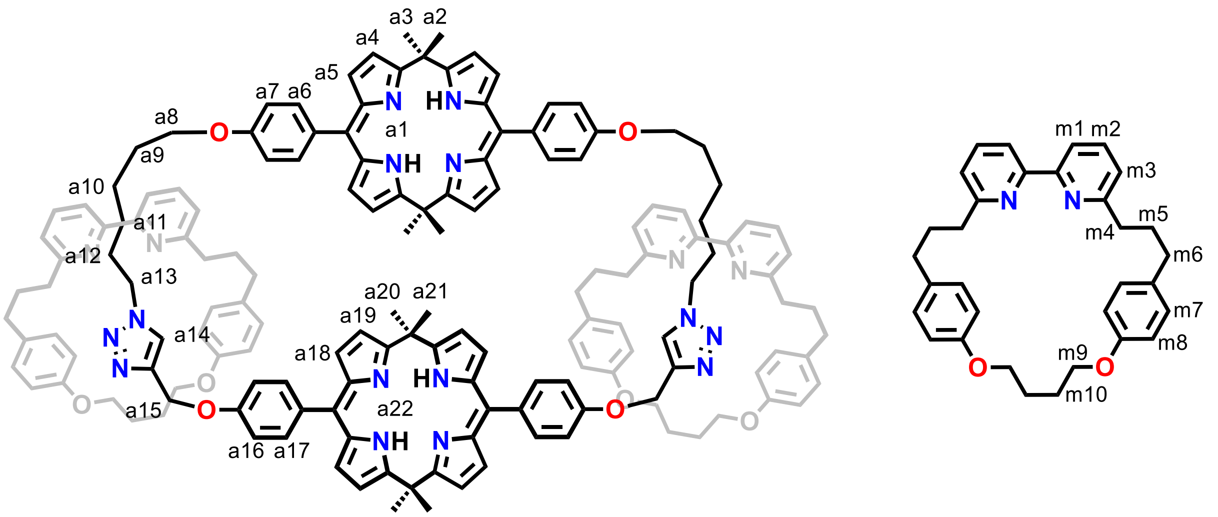

^{1}H NMR (600 MHz, $CDCl_3$, 300 K) δ (ppm): 14.24 (s, 2H, Ha1), 14.14 (s, 2H, Ha22), 8.32 (s, 2H, Ha14), 7.53 (t, 4H, ^3J = 7.7 Hz, Hm2), 7.32–7.27 (overlapping m, 8H, Hm1, Ha6), 7.27–7.21 (overlapping m, 4H, Ha17), 7.04 (dd, 4H, ^3J = 7.7 Hz, ^4J = 0.5 Hz, Hm3), 6.81 (d, 4H, ^3J = 8.5 Hz, Ha16), 6.73–6.67 (overlapping m, 12H, Ha7, Hm8), 6.64 (d, 8H, ^3J = 8.4 Hz, Hm7), 6.34 (dd, 4H, ^3J = 4.1 Hz, ^4J = 0.8 Hz, Ha5), 6.22 (dd, 4H, ^3J = 4.1 Hz, ^4J = 0.8 Hz, Ha4), 6.20 (dd, 4H, ^3J = 4.1 Hz, ^4J = 0.6 Hz, Ha18), 6.13 (dd, 4H, ^3J = 4.1 Hz, ^4J = 0.6 Hz, Ha19), 4.96 (s, 4H, Ha15), 4.28–4.20 (m, 4H, Hm9), 4.04–3.96 (m, 4H, Hm9), 3.62 (t, 4H, ^3J = 6.2 Hz, Ha8), 3.26 (m, 4H, Ha13), 2.58–2.50 (m, 4H, Hm6), 2.50–2.37 (overlapping m, 12H, Hm4, Hm6), 2.12–1.89 (overlapping, 8H, Hm10), 1.89 (b, 24H, Ha2, Ha3, Ha20, Ha21), 1.80–1.61 (m, 4H, Hm5), 1.39–1.18 (overlapping, 4H, Ha9), 0.96–0.63 (overlapping, 12H, Ha10, Ha11, Ha12).

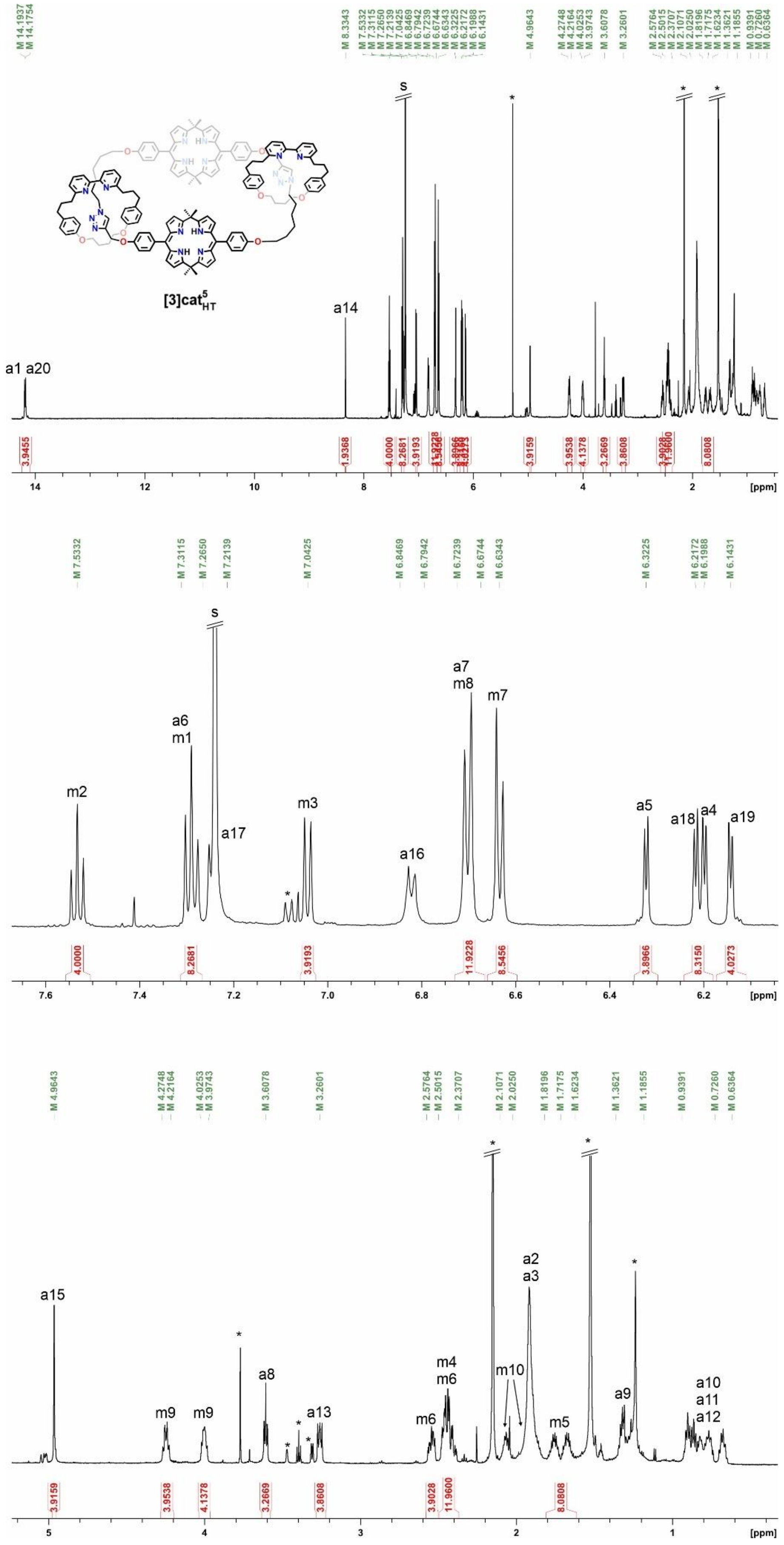

Figure S36. The ^{1}H NMR spectrum of **[3]cat$^5_{HT}$** (600 MHz, $CDCl_3$, 300 K).

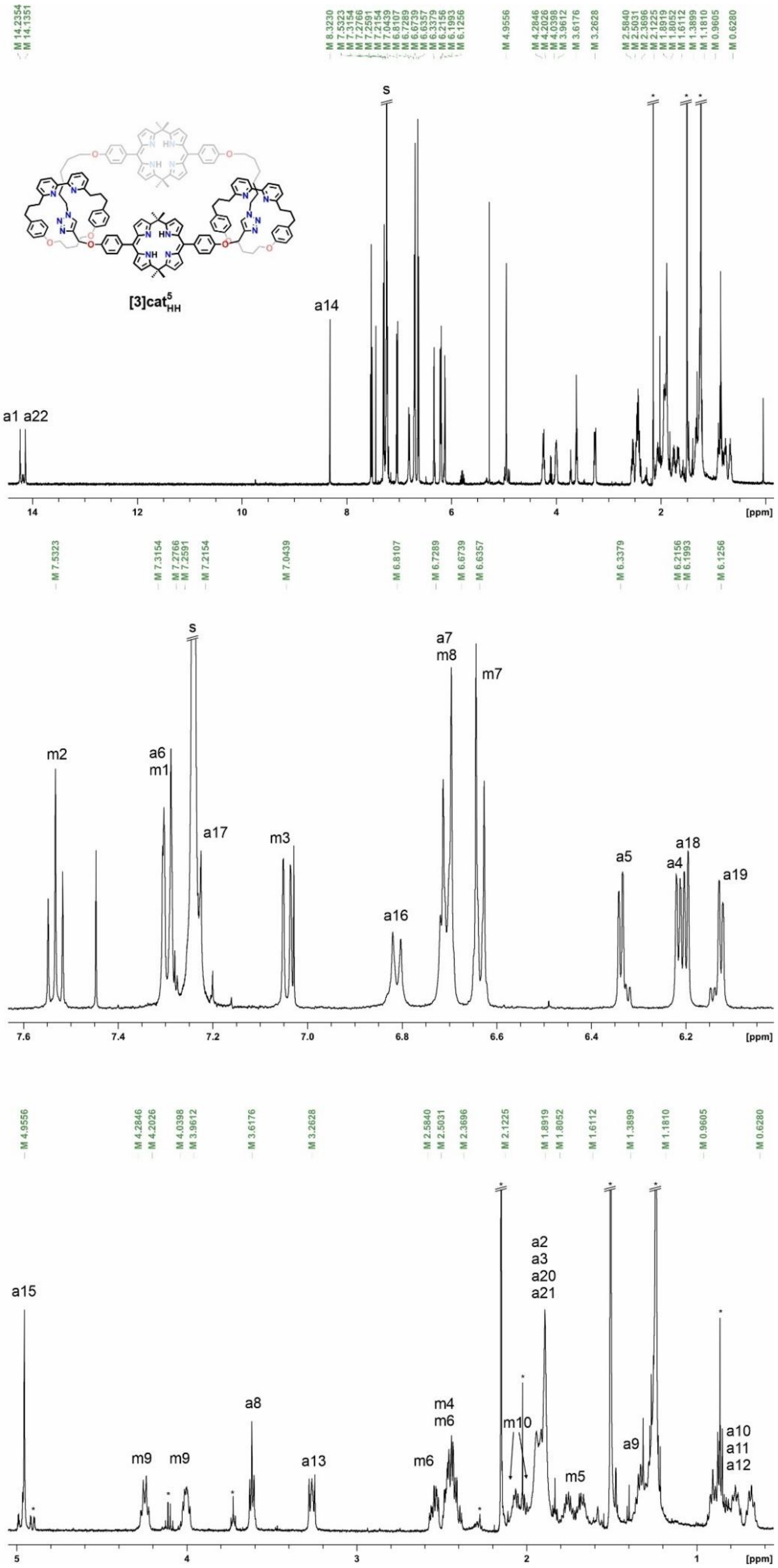

Figure S37. The ^1H NMR spectrum of **[3]cat$^{5}_{HH}$** (500 MHz, $CDCl_3$, 300 K).

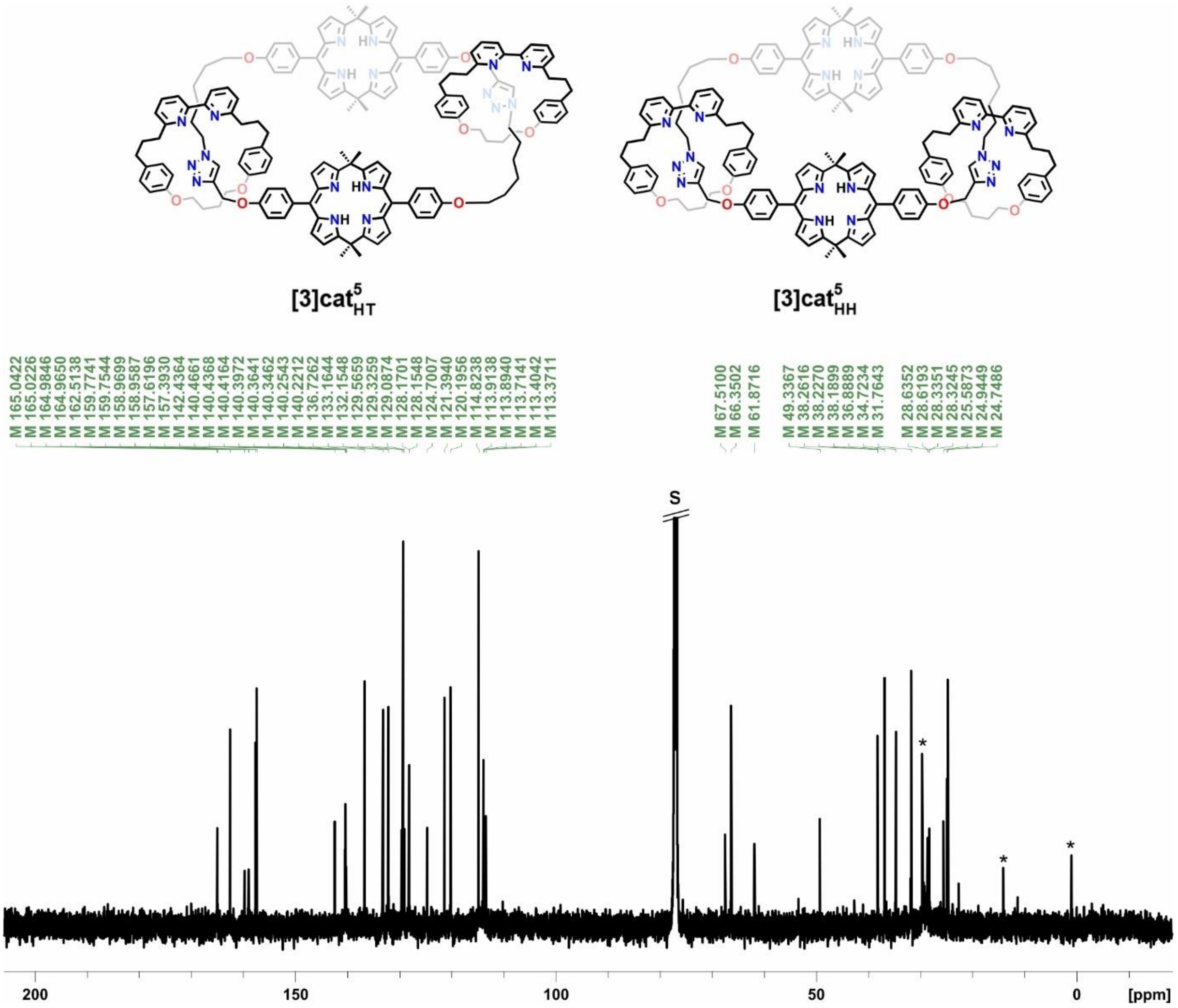

Figure S38. The ^{13}C NMR spectrum of mixture of isomers **[3]cat^5** (150 MHz, $CDCl_3$, 300 K). Impurities (mainly H grease) were labeled with asterisks.

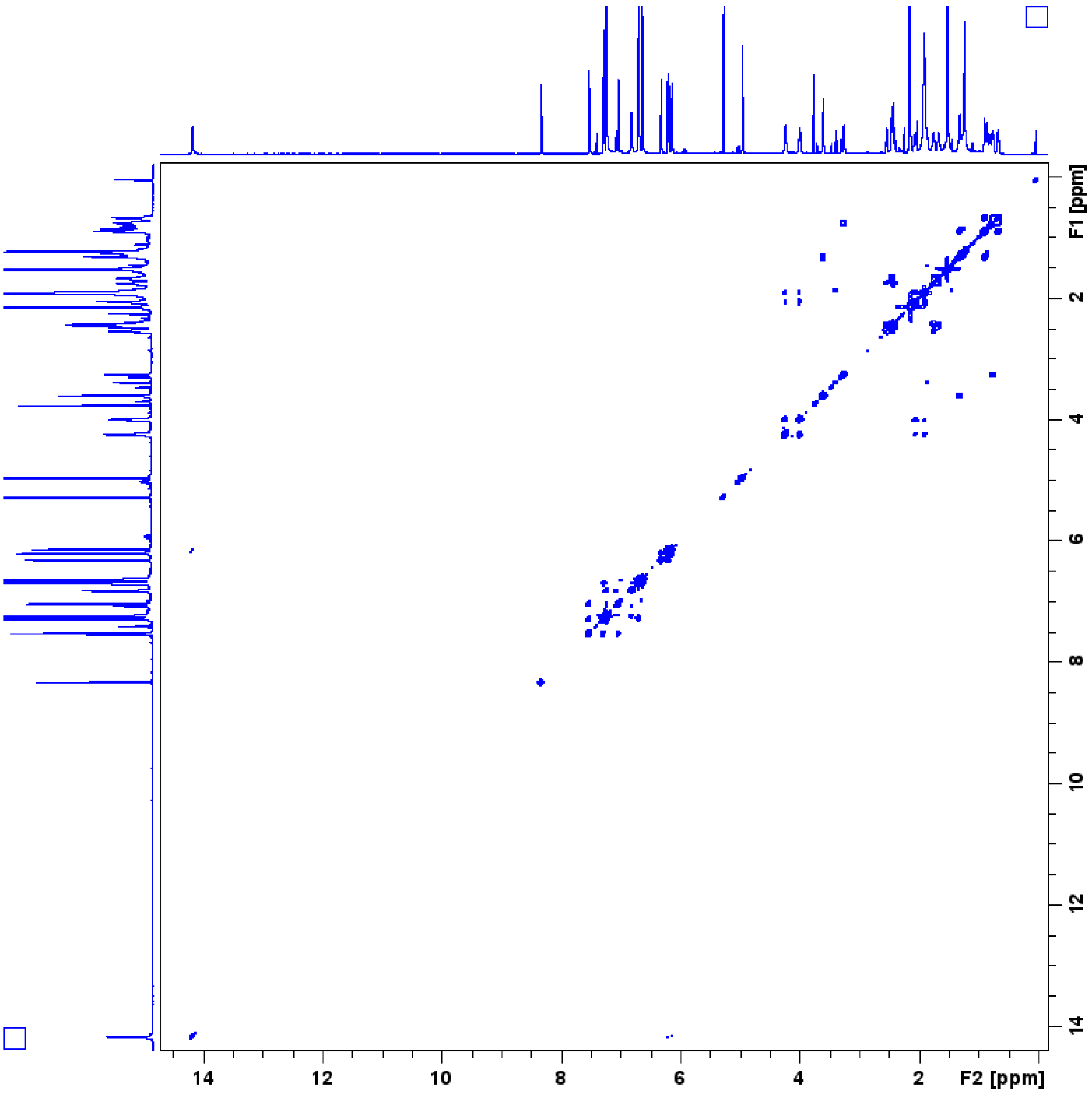

Figure S39. The ^{1}H-^{1}H COSY NMR spectrum of **[3]cat$^5_{HT}$** (600 MHz, $CDCl_3$, 300 K).

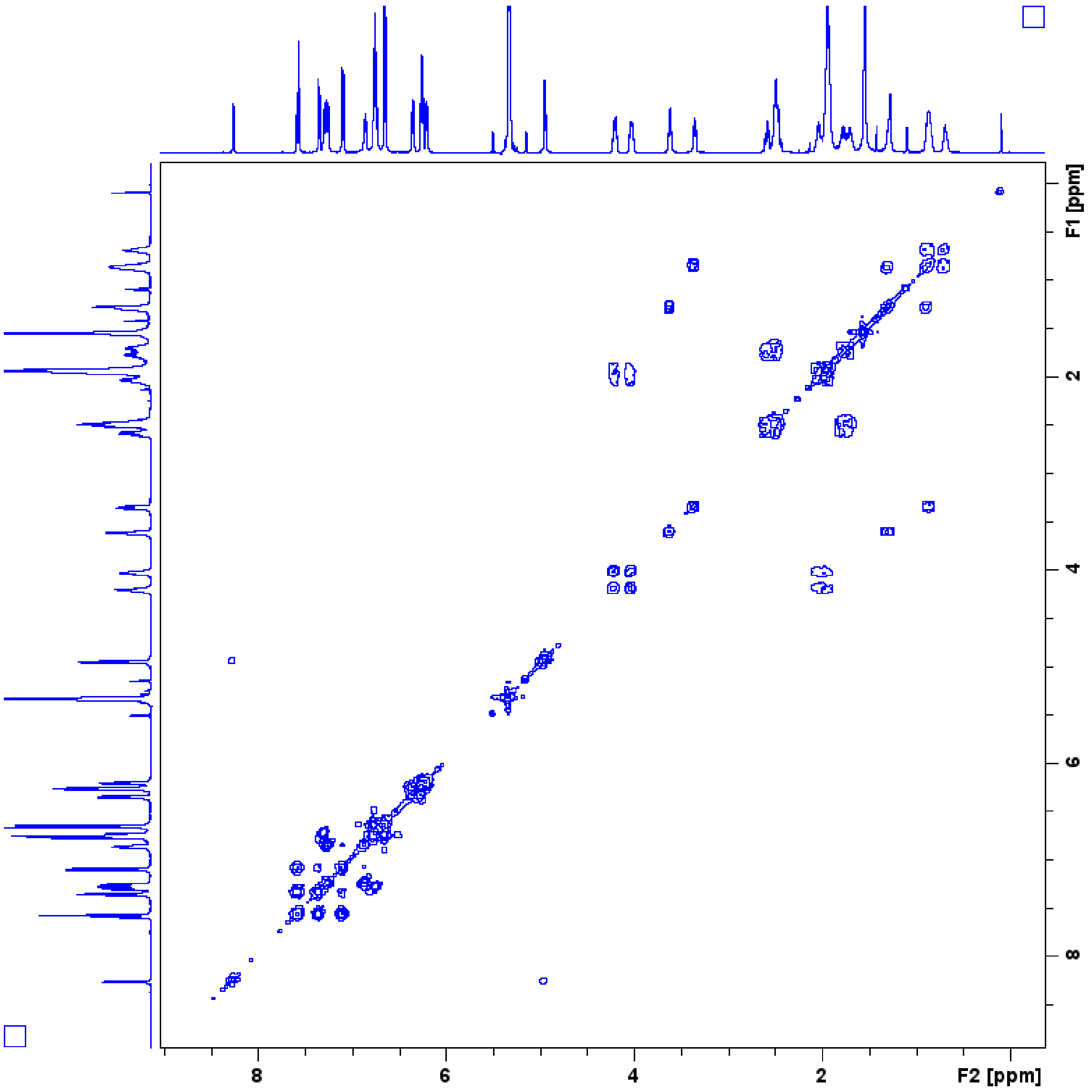

Figure S40. The ^{1}H-^{1}H COSY NMR spectrum of mixture of isomers **[3]cat^{5}** (500 MHz, CD_2Cl_2, 300 K).

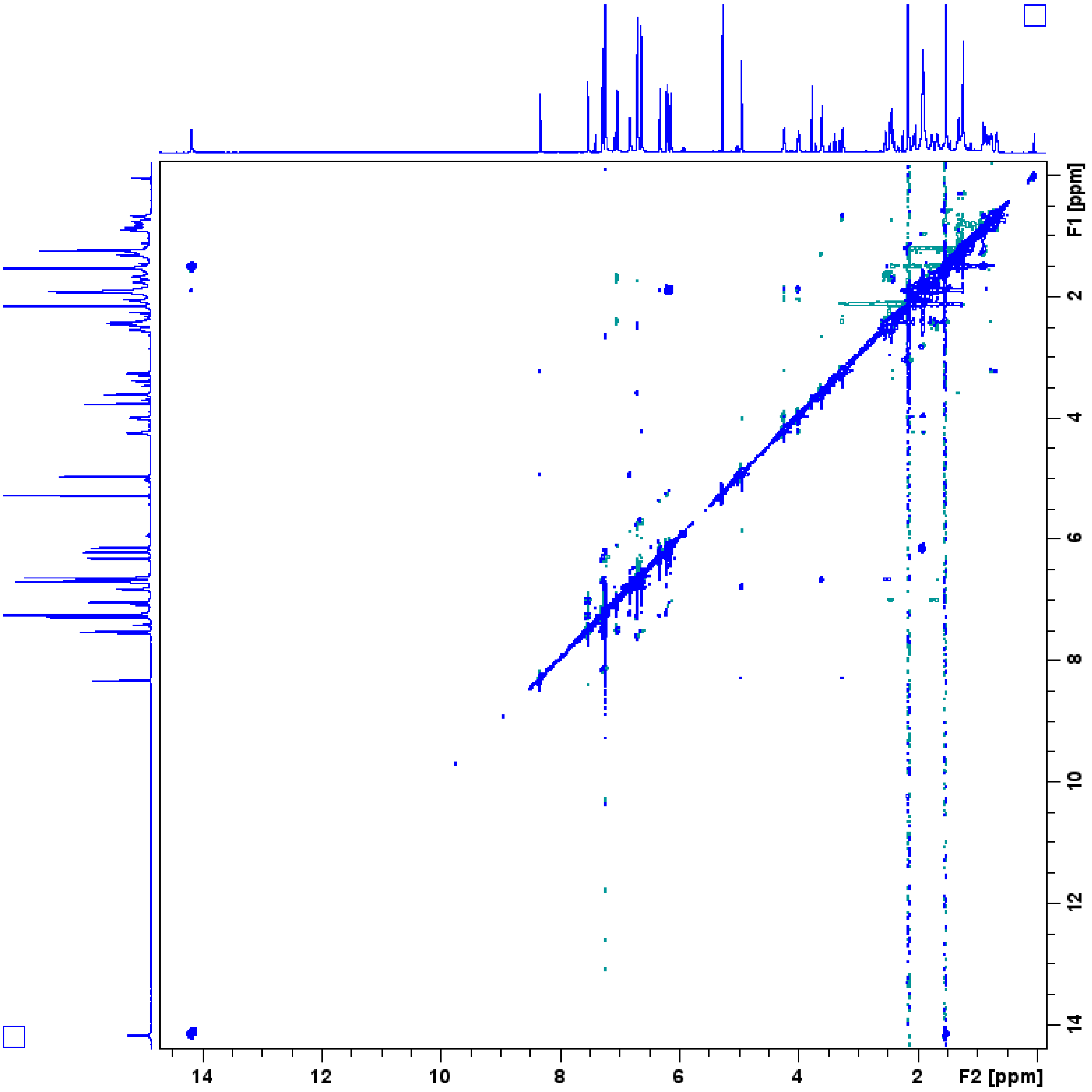

Figure S41. The ^{1}H-^{1}H NOESY NMR spectrum of **[3]cat$^{5}_{HT}$** (600 MHz, $CDCl_3$, 300 K).

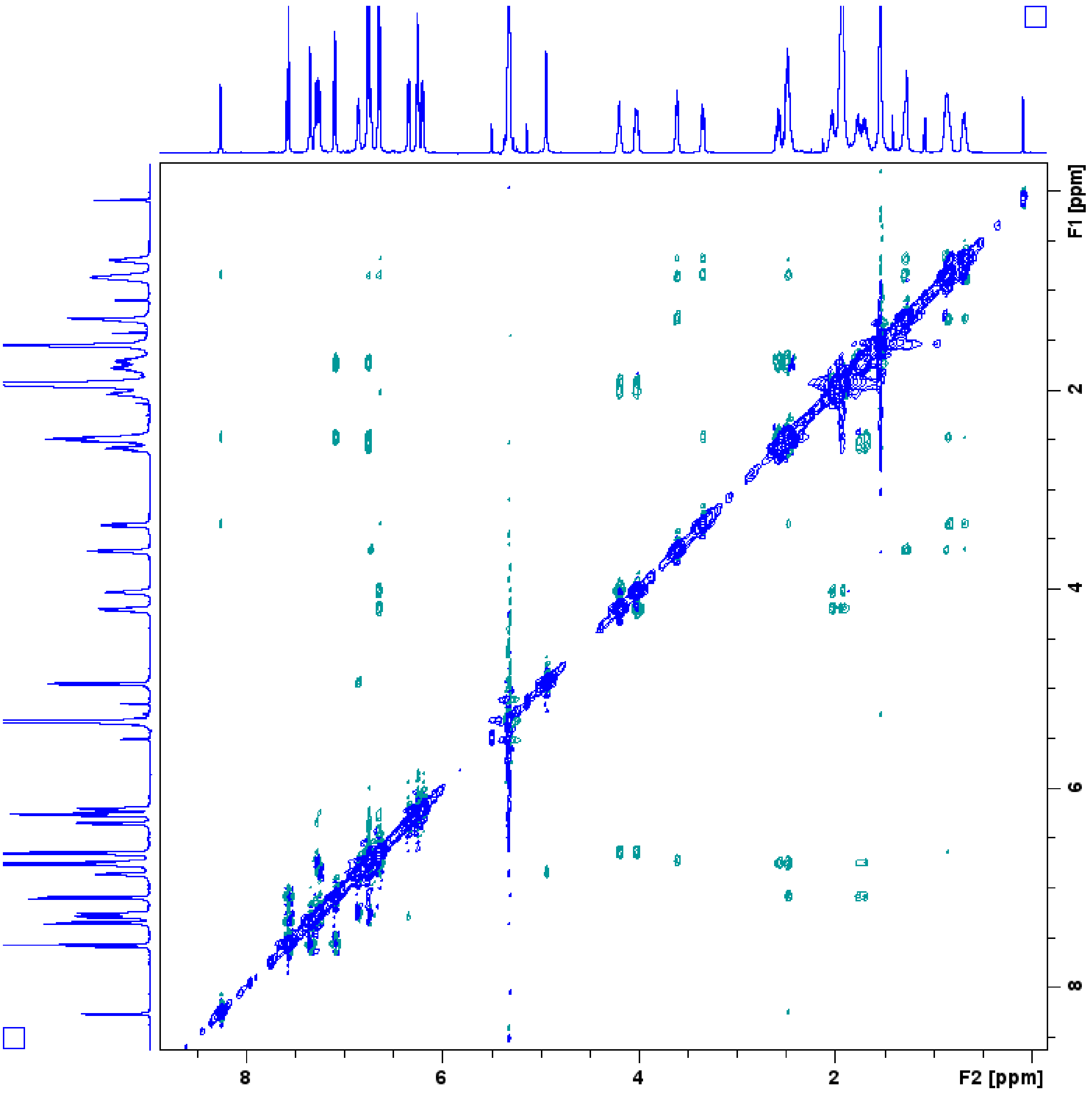

Figure S42. The ^{1}H-^{1}H NOESY NMR spectrum of mixture of isomers **[3]cat[5]** (500 MHz, CD_2Cl_2, 300 K).

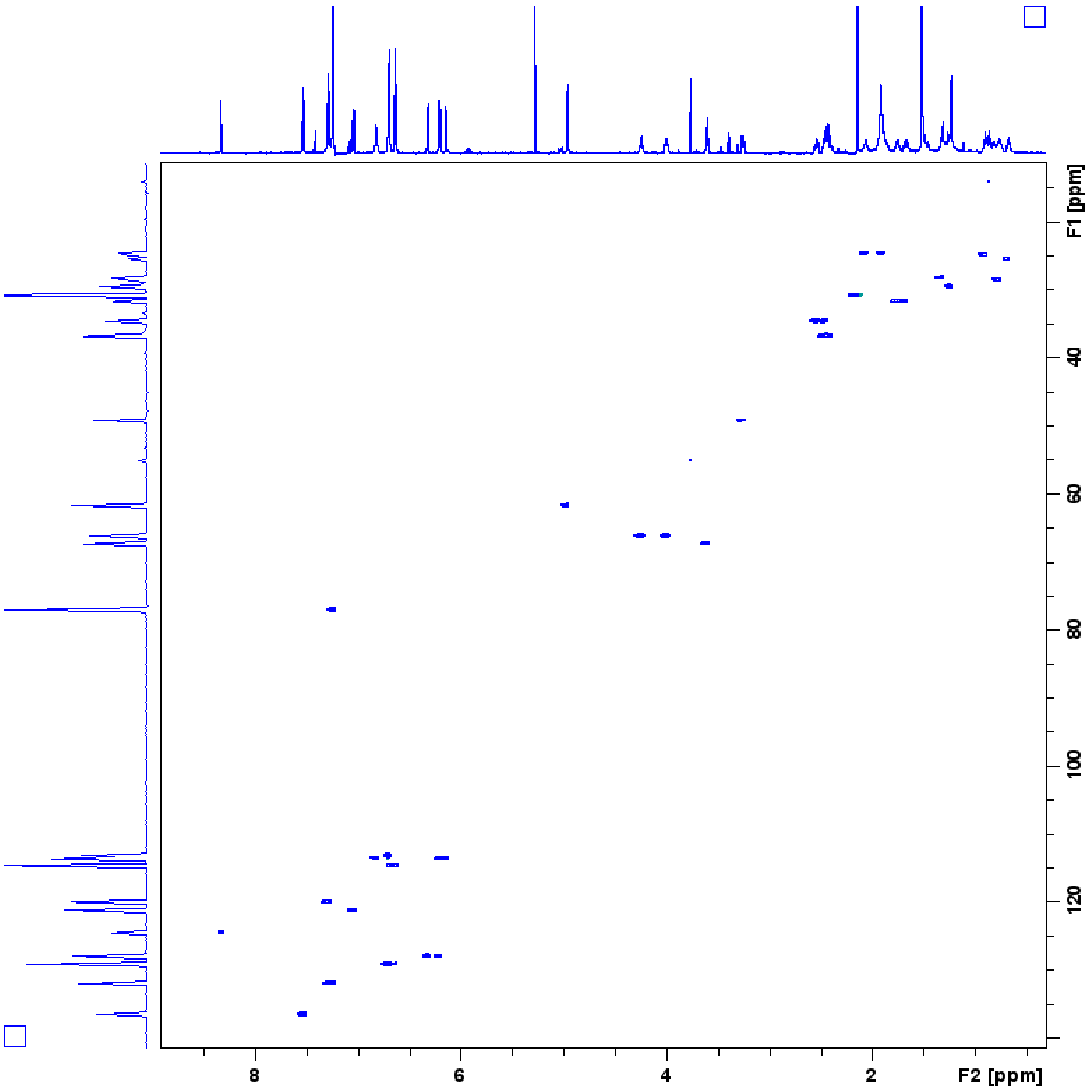

Figure S43. The ^1H-^{13}C HSQC NMR spectrum of **[3]cat$^5_{HT}$** (600 MHz, $CDCl_3$, 300 K).

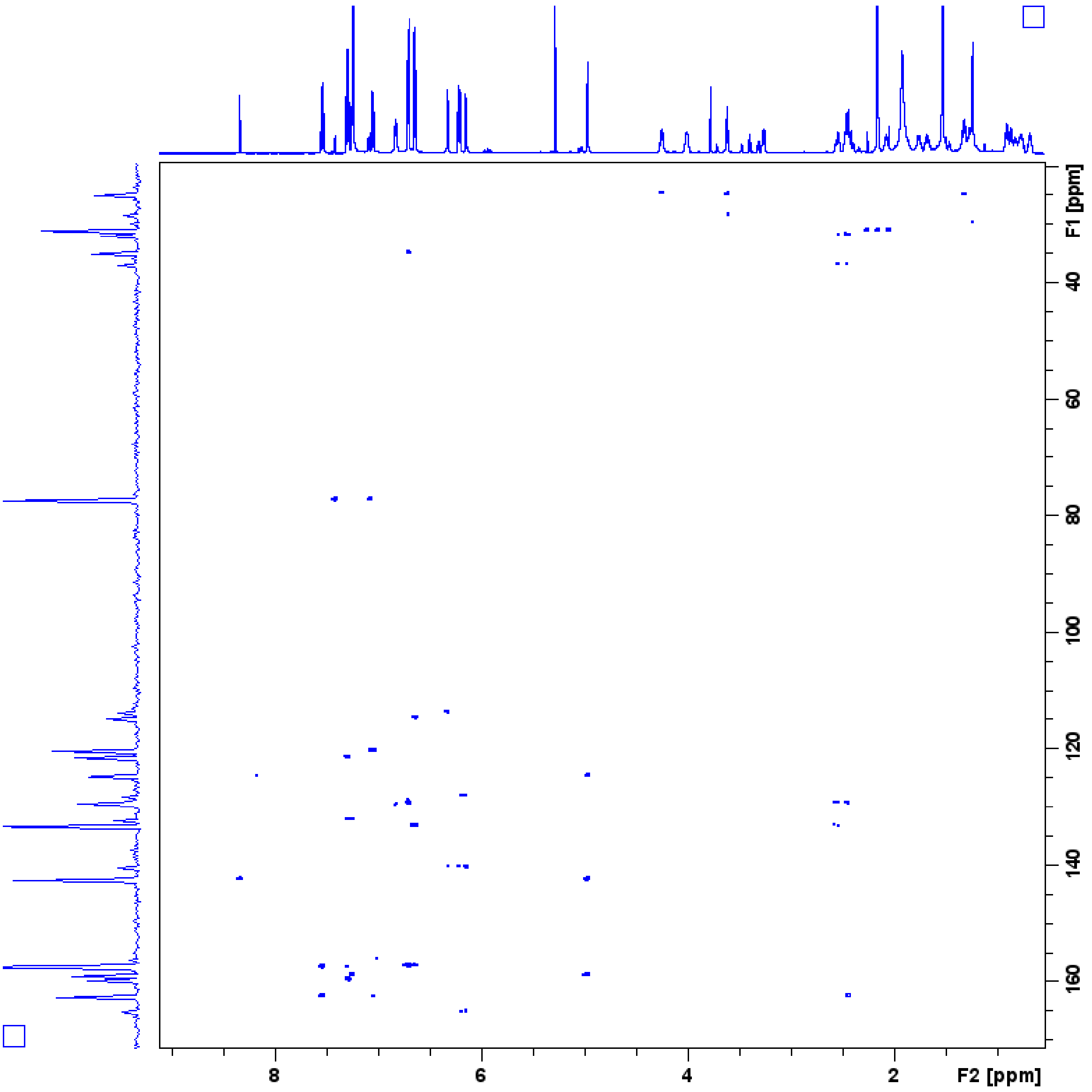

Figure S44. The ^{1}H-^{13}C HMBC NMR spectrum of **[3]cat$^{5}_{HT}$** (600 MHz, $CDCl_3$, 300 K).

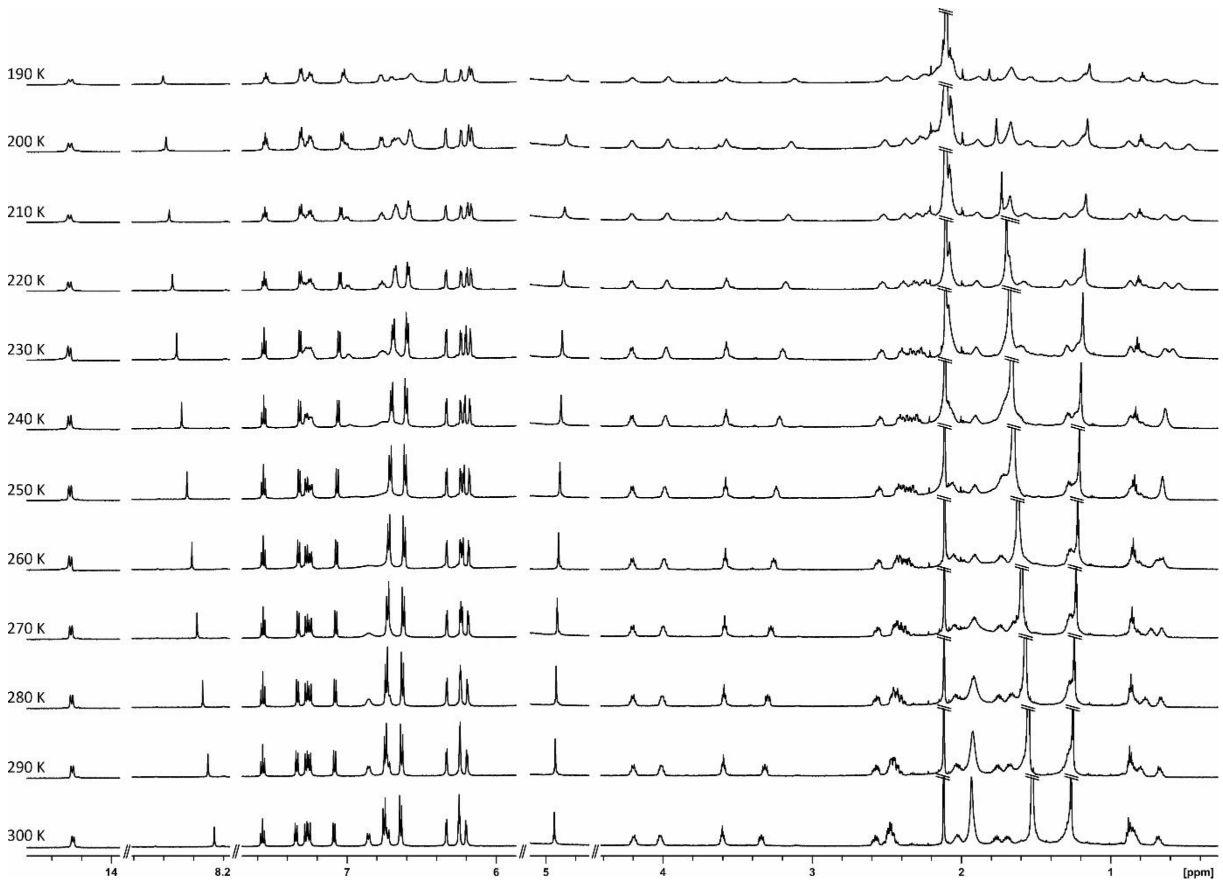

Figure S45. ^{1}H NMR spectra of **[3]cat$^{5}_{HT}$** recorded in the 300–190 K temperature range (600 MHz, CD_2Cl_2).

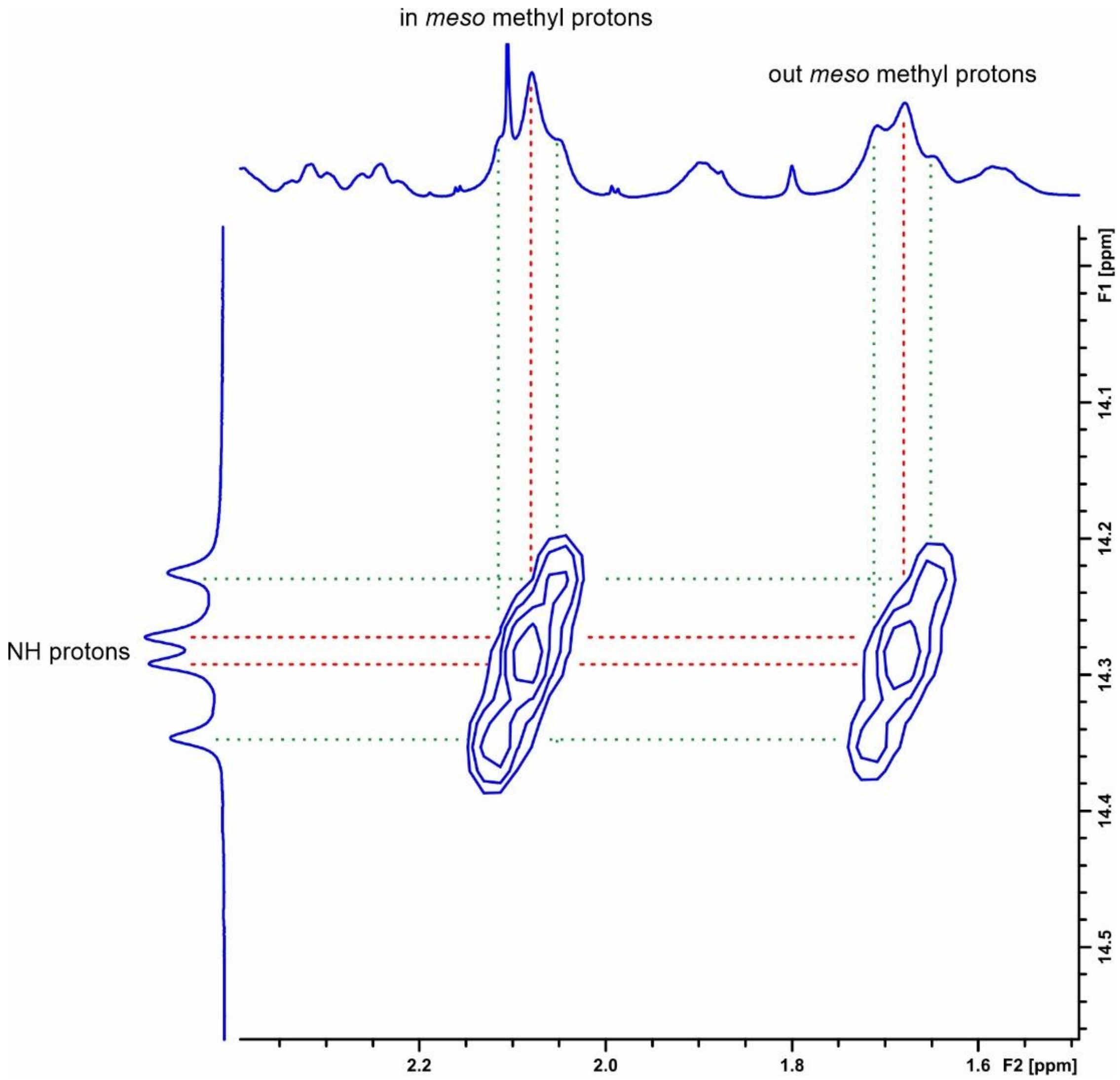

Figure S46. The ^{1}H-^{1}H NOESY NMR spectrum of mixture of isomers **[3]cat^{5}**. Correlation between NH and *meso*-methyl protons for different isomers highlighted by green and red dash lines (600 MHz, CD_2Cl_2, 220 K).

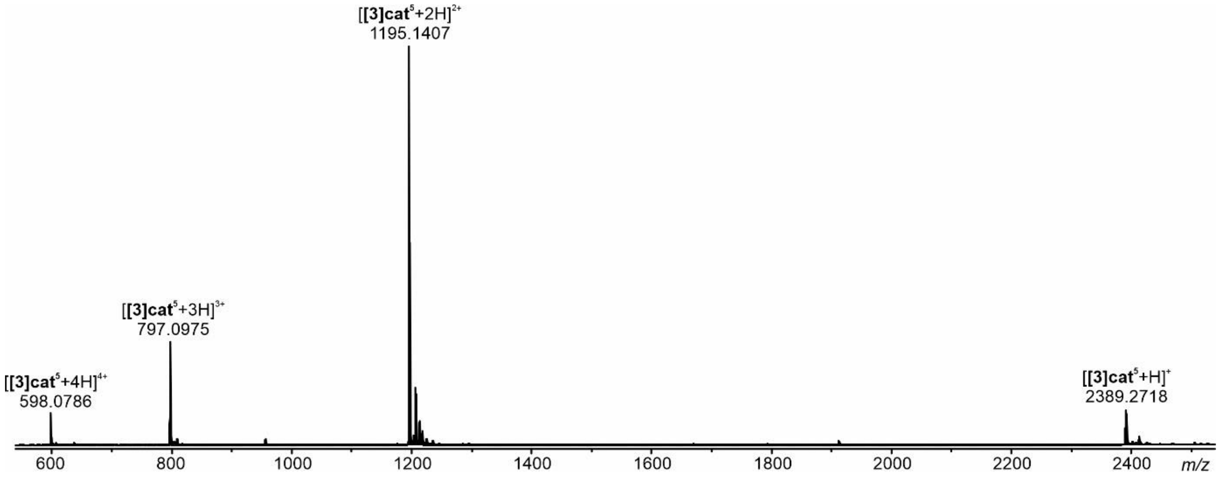

Figure S47. The ESI (TOF) mass spectrum of isomers **[3]cat^{5}**.

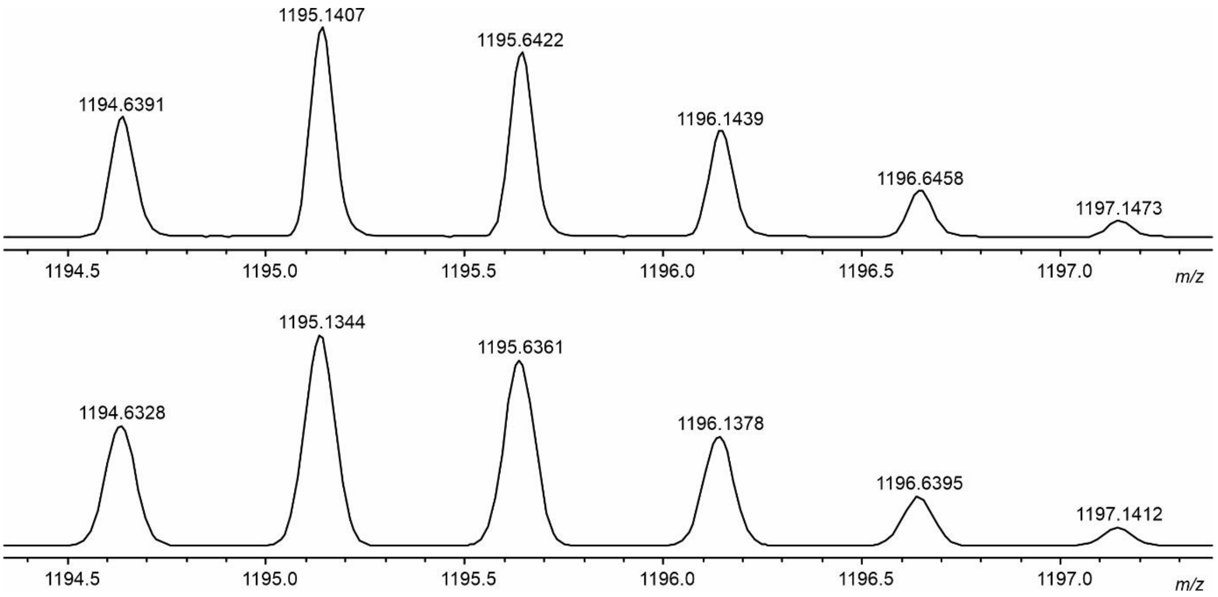

Figure S48. The ESI (TOF) mass spectrum of isomers of **[3]cat^{5}**.Top: experimental, bottom: simulated isotopic pattern.

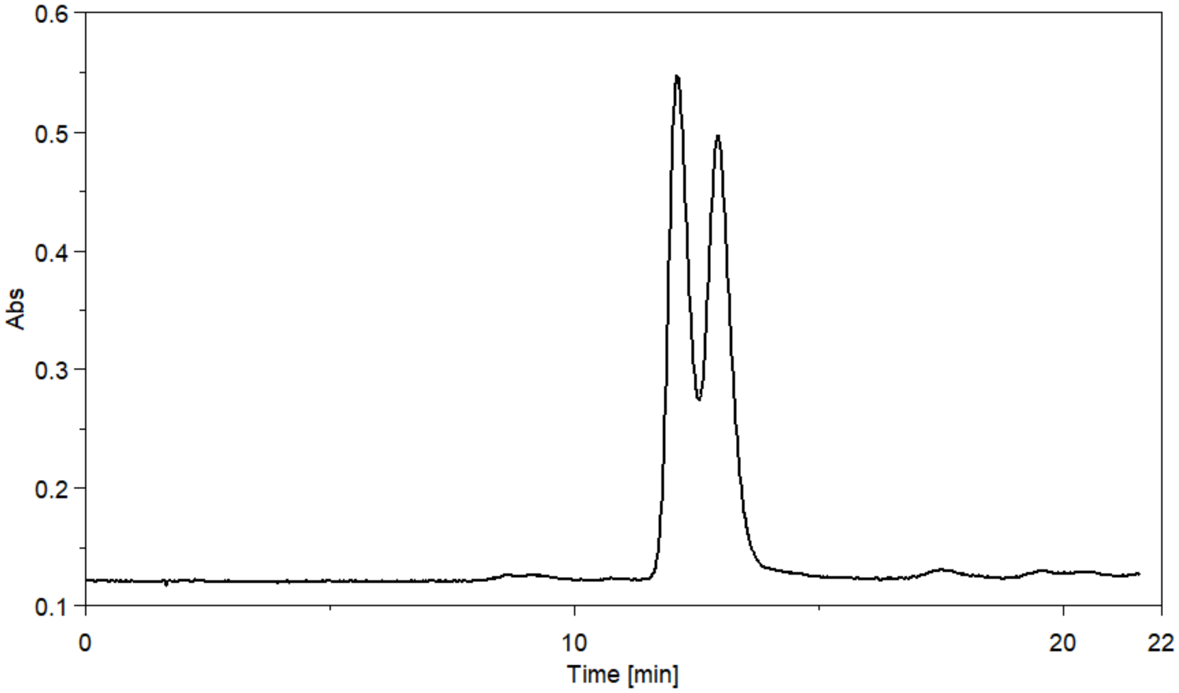

Figure S49. HPLC chromatogram of mixture of **[3]cat^{5}**.

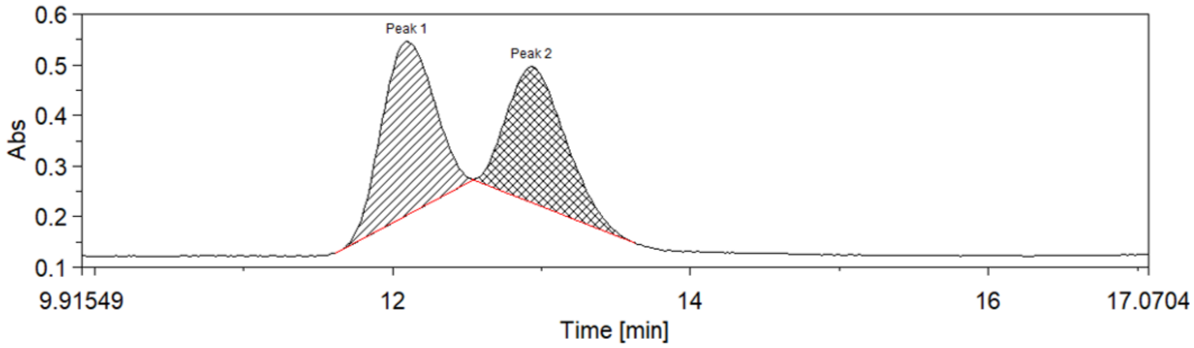

[Result of Peak Area Calculation]

	CalcRange		BaseRange		Area	Ratio
Peak 1	----	----	11.6167	12.55	0.140388	1.13191
Peak 2	----	----	12.5333	13.6333	0.124028	0.883466

Figure S50. HPLC chromatogram of mixture of **[3]cat^{5}**.

Catenane [3]cat^6

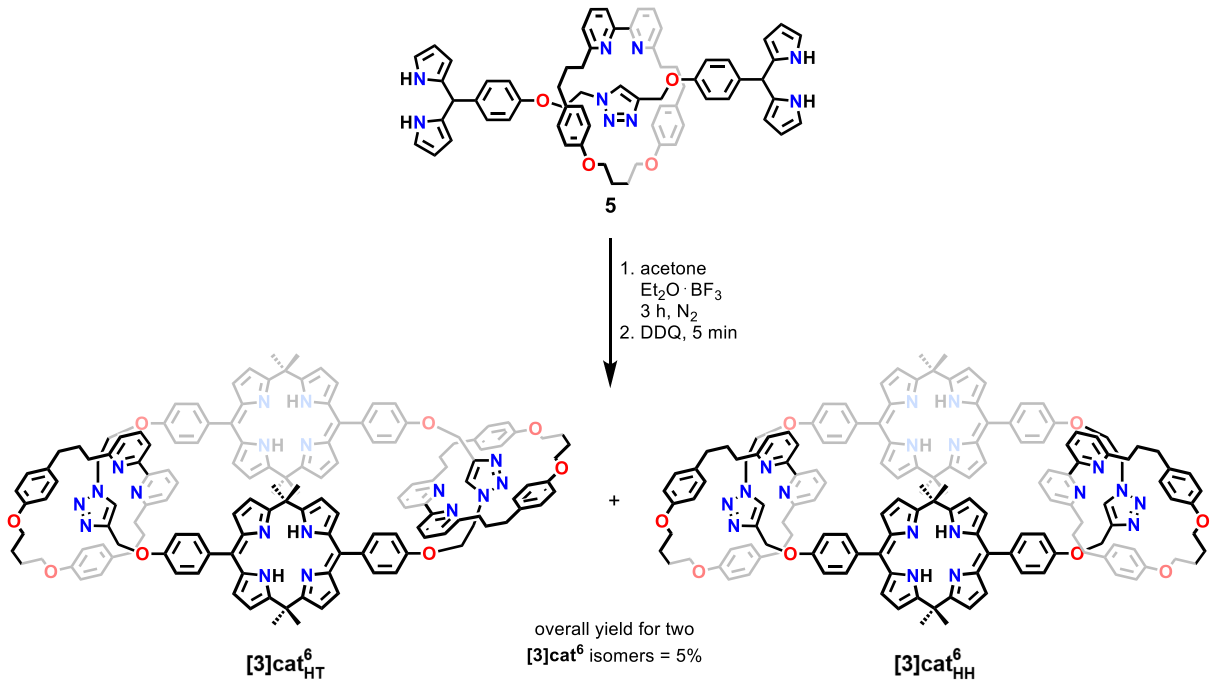

In a 50 mL round bottom flask, **6** (91.6 mg, 86.2 μmol) and acetone (30 mL) were introduced. The mixture was deoxygenated *via* nitrogen bubbling for 10 minutes. Then, $Et_2O·BF_3$ (28 μL, 227 μmol) was added *via* the syringe. The mixture was then stirred for 3 hours under a nitrogen atmosphere. After this time, the flask was opened, DDQ (70 mg, 308 μmol) was introduced, and the reaction was carried out for an additional 5 minutes. Then, the acid was quenched by adding TEA (0.5 mL), and the mixture was passed through a short column with deactivated aluminum oxide. Residues on the column were washed out with ethyl acetate. The solvent was removed under reduced pressure. The reddish oil was purified *via* flash chromatography (DCM with 0-20% ethyl acetate gradient) and then recrystallized with ethyl acetate to provide mixture of isomers **[3]cat^6** (5 mg, 2.2 μmol, 5%) as a orange crystalline solid. Isomers could be partially separated. The isomer **[3]cat$^6_{HT}$** created an insoluble solid. After acidification of this solid with TFA, followed by neutralisation with $NaHCO_3$ and extraction with DCM, the second isomer **[3]cat$^6_{HT}$** could be isolated. Unfortunately, it precipitated over time forming an insoluble solid.

*Analytical data for **[3]cat$^{6}_{HH}$**:*

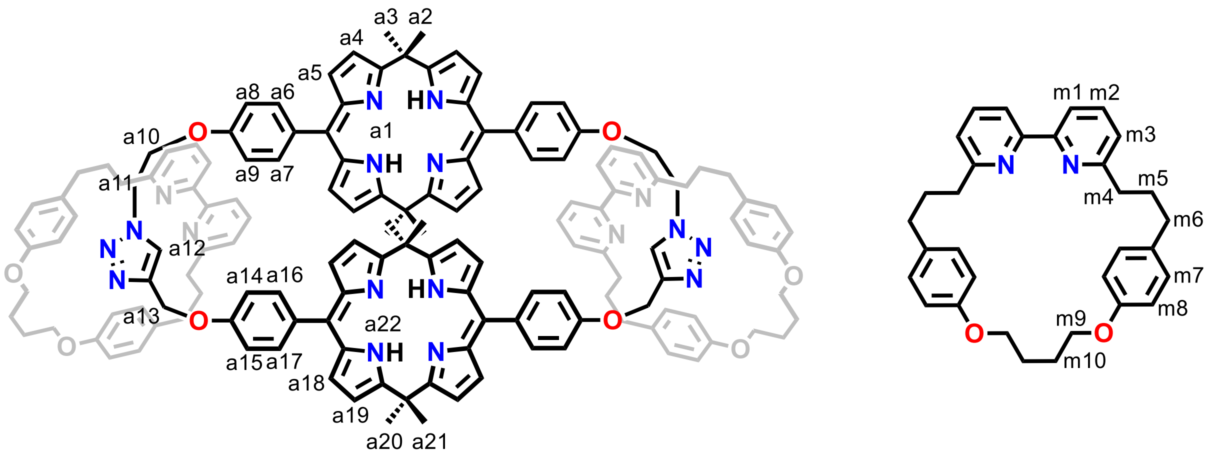

^{1}H NMR (600 MHz, CD_2Cl_2, 270 K) δ (ppm): 14.26 (b, 2H, Ha1), 14.23 (b, 2H, Ha22), 9.00 (s, 2H, Ha12), 7.55 (t, 4H, ^{3}J = 7.7 Hz, Hm2), 7.29 (d, 4H, ^{3}J = 7.7 Hz, Hm1), 7.19 (dd, 2H, ^{3}J = 8.5 Hz, ^{4}J = 1.8 Hz, Ha16/Ha17), 7.10 (dd, 2H, ^{3}J = 8.5 Hz, ^{4}J = 1.8 Hz, Ha6/Ha7), 7.03–6.98 (overlapping d and dd, 6H, Hm3, Ha16/Ha17), 6.91 (dd, 2H, ^{3}J = 8.5 Hz, ^{4}J = 1.8 Hz, Ha14/Ha15), 6.79 (dd, 2H, ^{3}J = 8.5 Hz, ^{4}J = 1.8 Hz, Ha6/Ha7), 6.65–6.60 (overlapping m, 16H, Hm7, Hm8), 6.57 (dd, 2H, ^{3}J = 8.5 Hz, ^{4}J = 1.8 Hz, Ha8/Ha9), 6.42 (dd, 2H, ^{3}J = 8.5 Hz, ^{4}J = 1.8 Hz, Ha14/Ha15), 6.24–6.20 (overlapping m and dd, 6H, Ha4, Ha8/Ha9), 6.17 (dd, 4H, ^{3}J = 4.1 Hz, ^{4}J = 0.7 Hz, Ha5), 6.15 (d, 4H, ^{3}J = 4.1 Hz, ^{4}J = 0.9 Hz, Ha19), 6.11 (d, 4H, ^{3}J = 4.1 Hz, ^{4}J = 0.7 Hz, Ha18), 4.90 (s, 4H, Ha13), 4.31–4.25 (m, 4H, Hm9), 4.08–4.02 (m, 4H, Hm9), 3.66 (t, 4H, ^{3}J = 6.8 Hz, Ha11), 3.36 (t, 4H, ^{3}J = 6.8 Hz, Ha10), 2.53–2.46 (m, 4H, Hm6), 2.35–2.27 (m, 8H, Hm4, Hm6), 2.24–2.18 (m, 4H, Hm4), 2.15–2.05 (overlapping m, 4H, Hm10), 2.12 (s, 6H, Ha2), 2.08 (s, 6H, Ha21), 2.03–1.95 (m, 4H, Hm10), 1.72–1.46 (overlapping m, 8H, Hm5), 1.71 (s, 6H, Ha3), 1.68 (s, 6H, Ha3).

^{13}C NMR (150 MHz, CD_2Cl_2, 270 K) δ (ppm): 164.4, 164.3, 161.8, 158.4, 158.0, 156.7, 156.5, 142.3, 139.34, 139.32, 136.3, 132.7, 131.7, 131.4, 131.03, 130.96, 128.8, 128.2, 128.0, 127.4 (2 overlapping signals), 125.4, 120.9, 119.7, 114.0, 113.8, 113.3, 112.9, 112.4, 65.6, 64.0, 60.7, 47.1, 37.52, 37.48, 36.1, 34.1, 32.3, 32.1, 31.2, 24.0, 23.0 (2 overlapping signals).

HRMS (ESI+, TOF) *m/z*: $[M+3H]^{3+}$ calcd. for $C_{146}H_{145}N_{18}O_8^{3+}$, 759.7169; found, 759.7216.

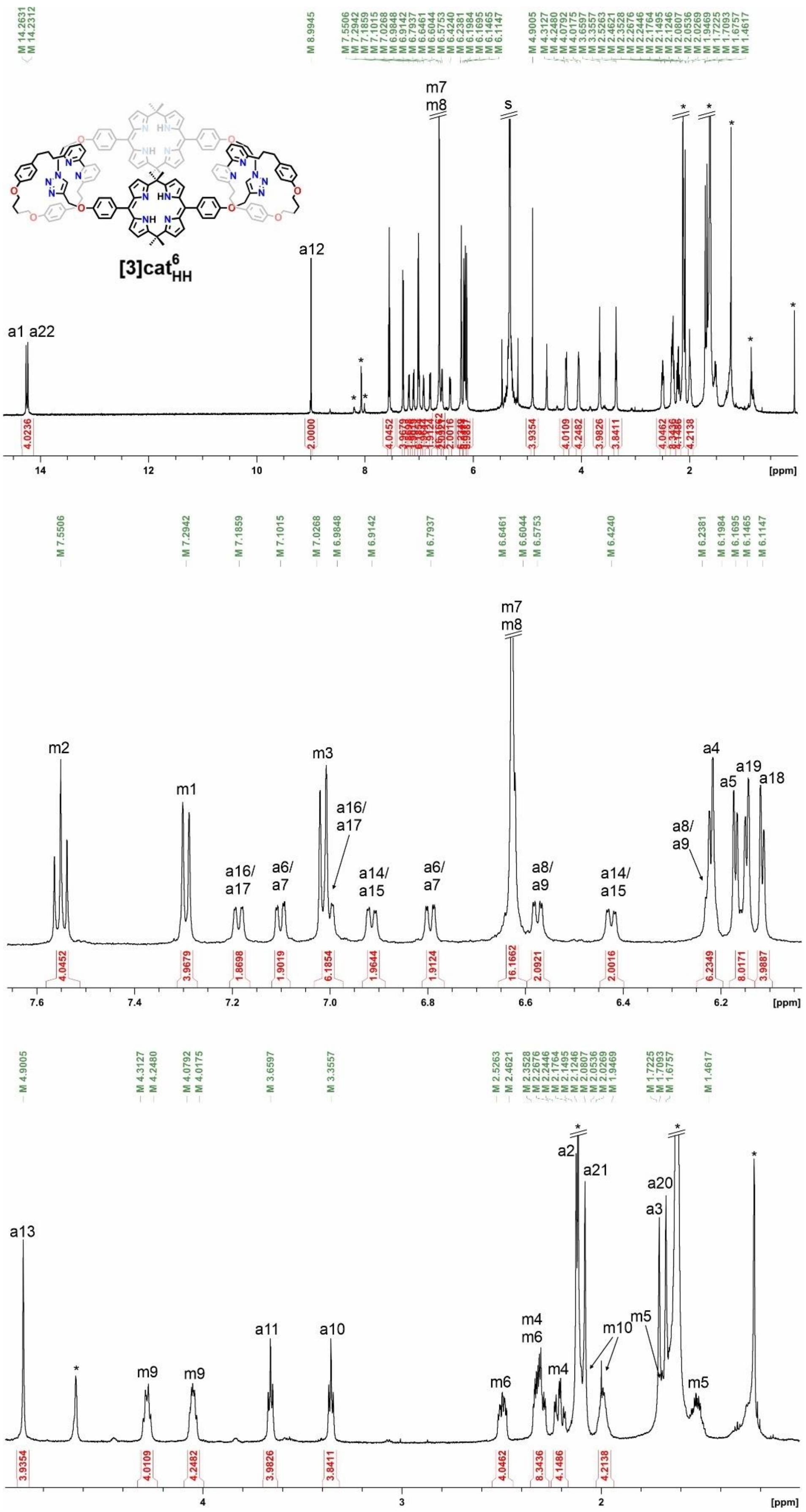

Figure S51. The ^{1}H NMR spectrum of **[3]cat$^{6}_{HH}$** (600 MHz, CD_2Cl_2, 270 K).

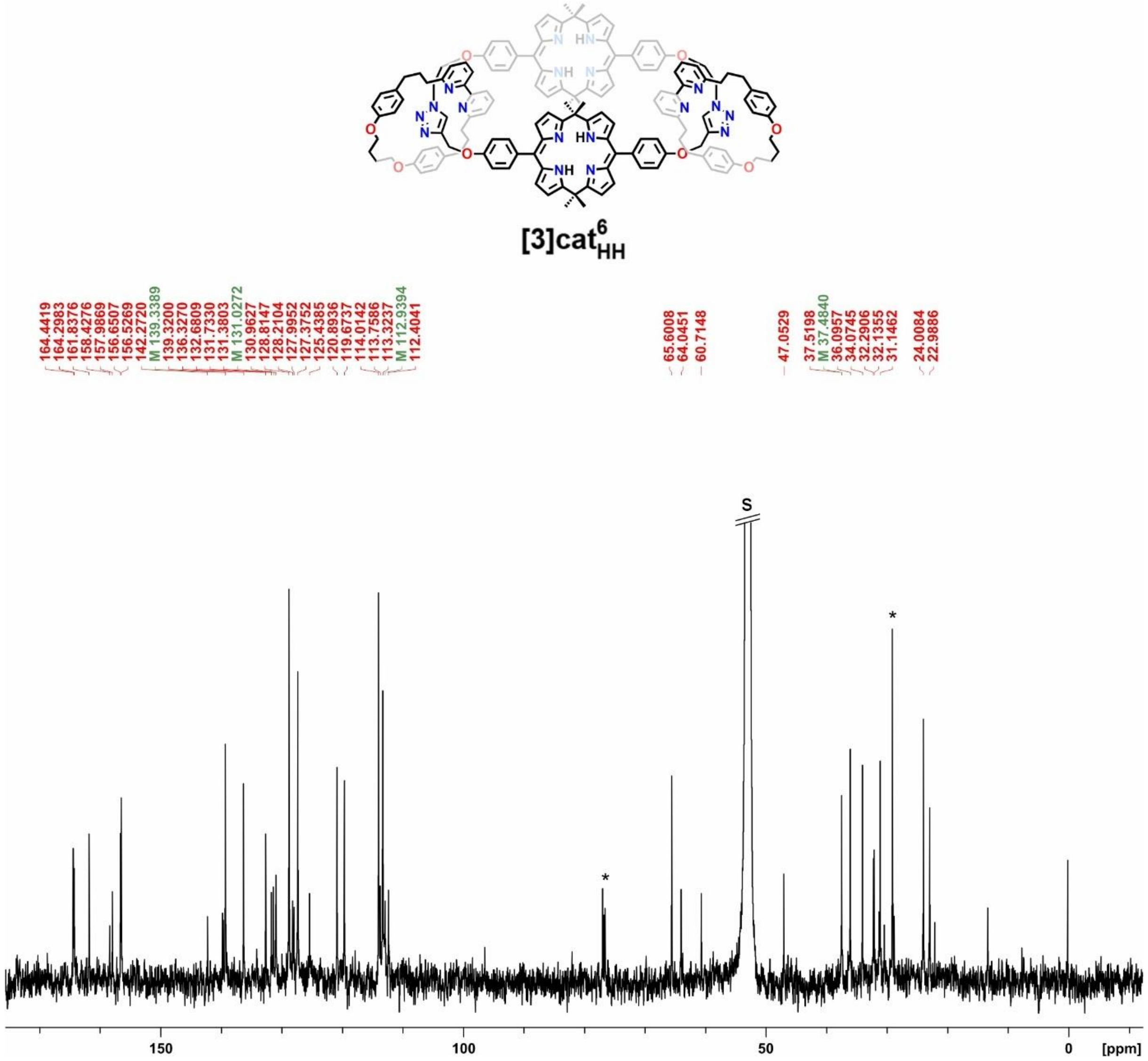

Figure S52. The ^{13}C NMR spectrum **of [3]cat$^{6}_{HH}$** (150 MHz, CD_2Cl_2, 270K). Impurities (mainly H grease) were labeled with asterisks.

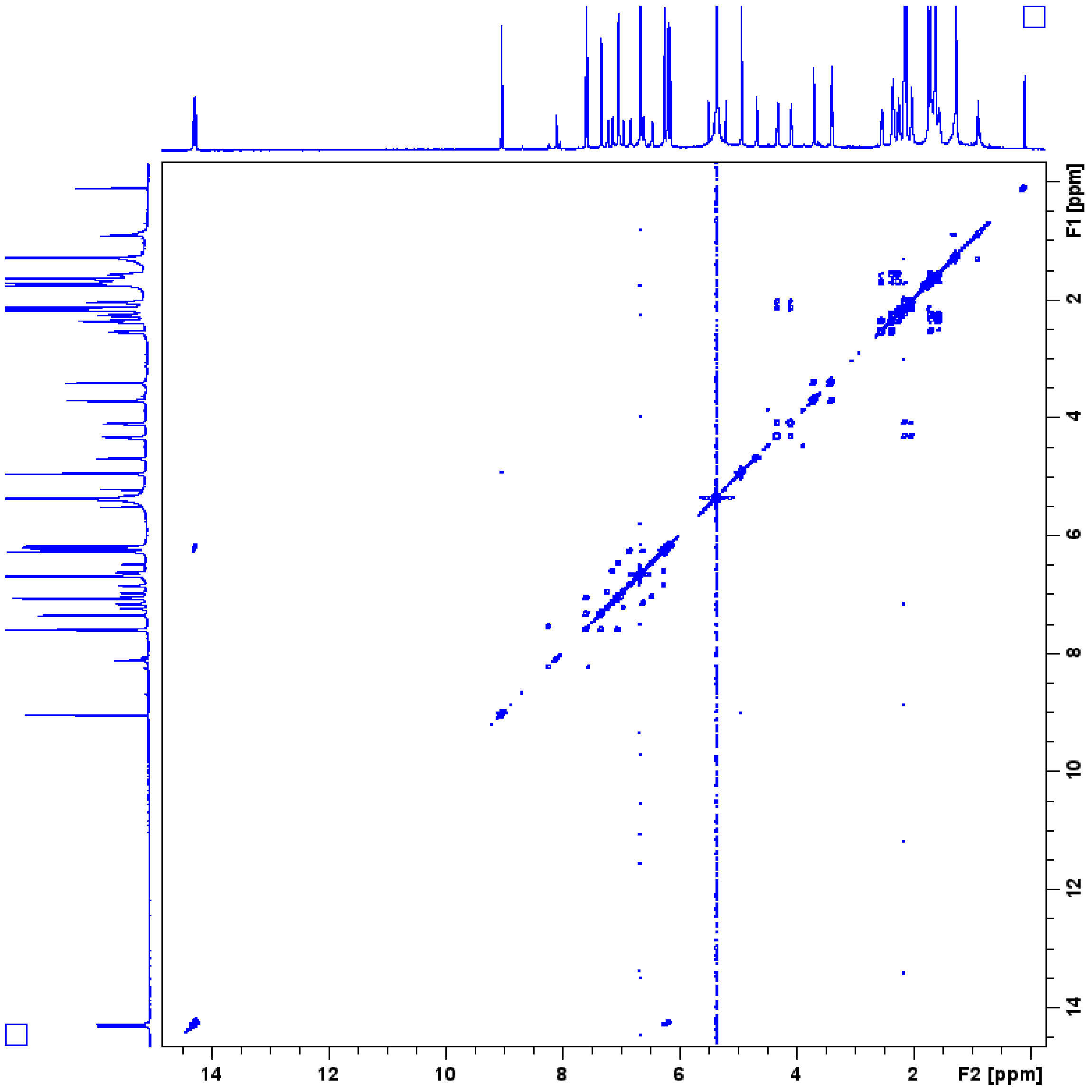

Figure S53. The ^{1}H-^{1}H COSY NMR spectrum of **[3]cat$^{6}_{HH}$** (600 MHz, CD_2Cl_2, 270 K).

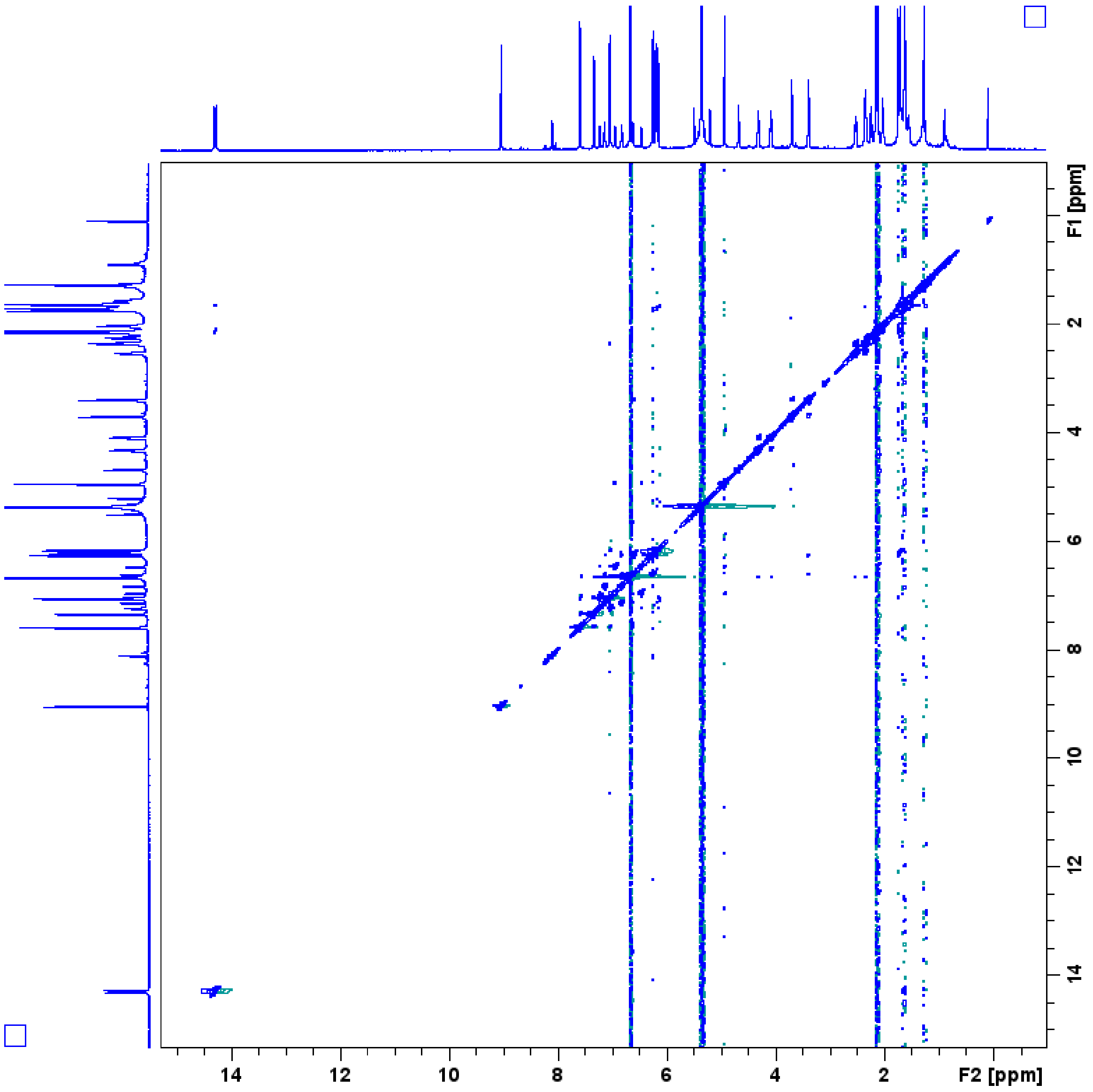

Figure S54. The ^{1}H-^{1}H NOESY NMR spectrum of **[3]cat$^{6}_{HH}$** (600 MHz, CD_2Cl_2, 270 K).

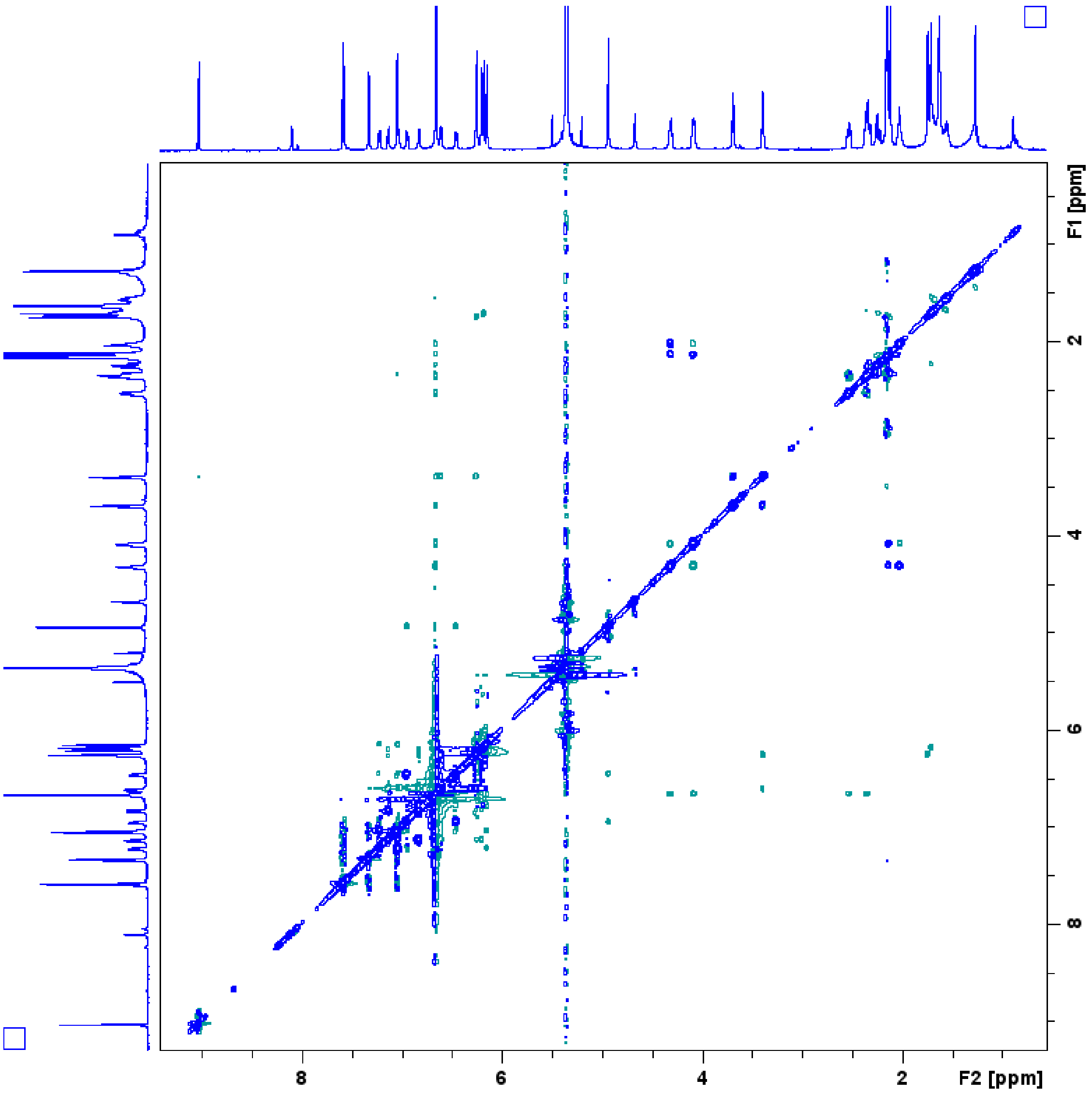

Figure S55. The ^{1}H-^{1}H ROESY NMR spectrum of **[3]cat$^{6}_{HH}$** (600 MHz, CD_2Cl_2, 270 K).

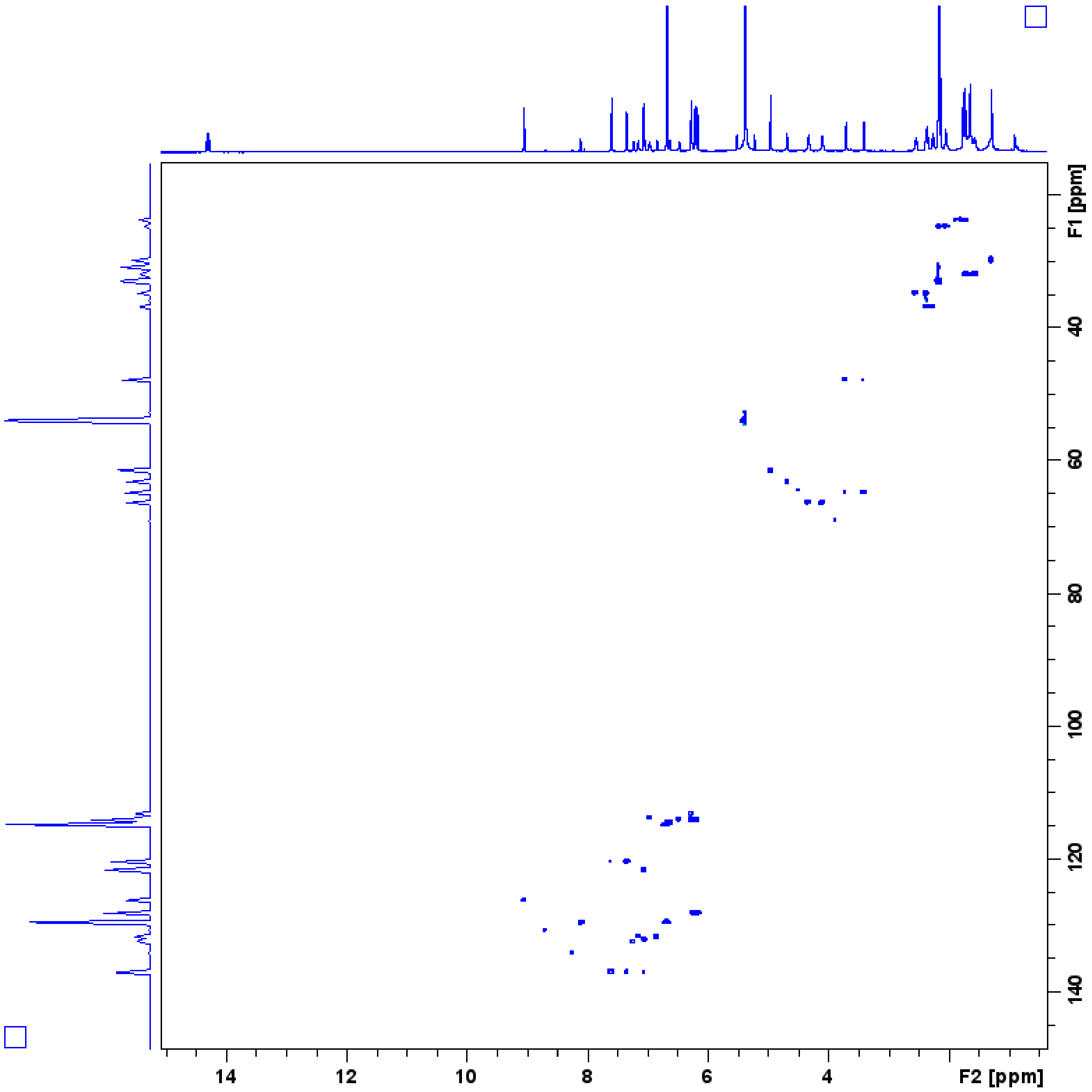

Figure S56. The ^{1}H-^{13}C HSQC NMR spectrum of **[3]cat$^{6}_{HH}$** (600 MHz, CD_2Cl_2, 270 K).

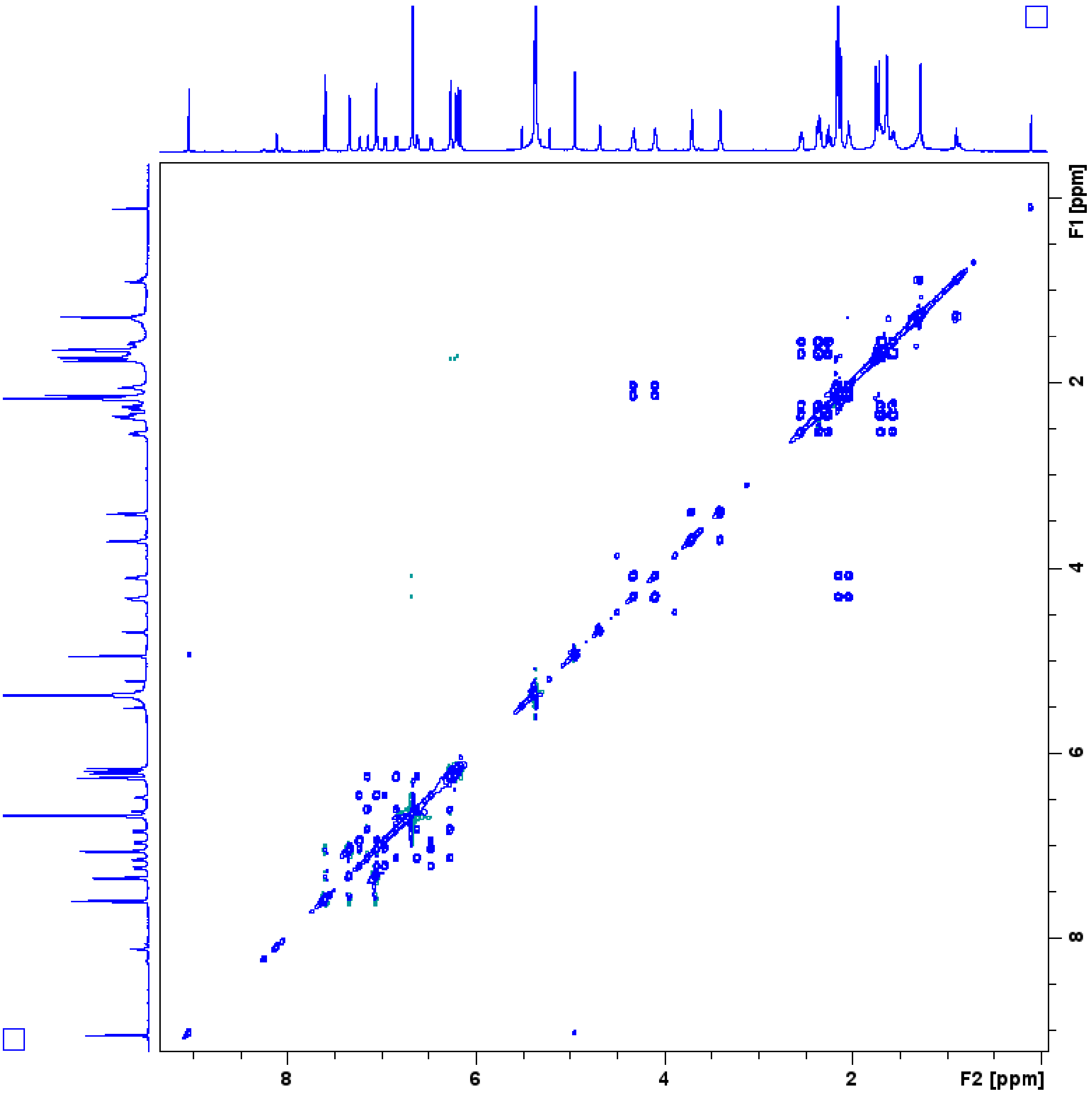

Figure S57. The ^{1}H-^{1}H TOCSY NMR spectrum of **[3]cat$^{6}_{HH}$** (600 MHz, CD_2Cl_2, 270 K).

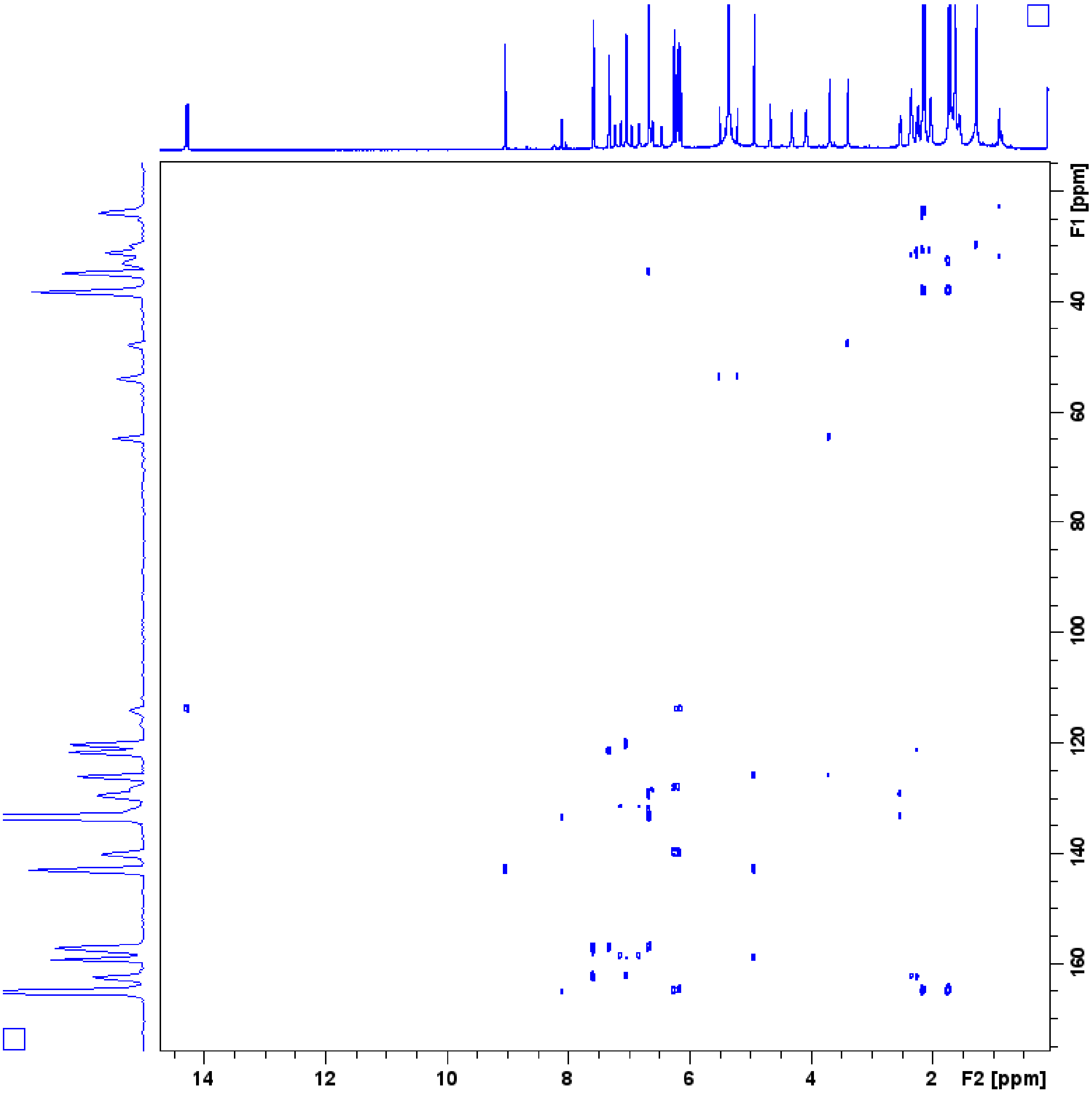

Figure S58. The ^{1}H-^{13}C HMBC NMR spectrum of **[3]cat$^{6}_{HH}$** (600 MHz, CD_2Cl_2, 270 K).

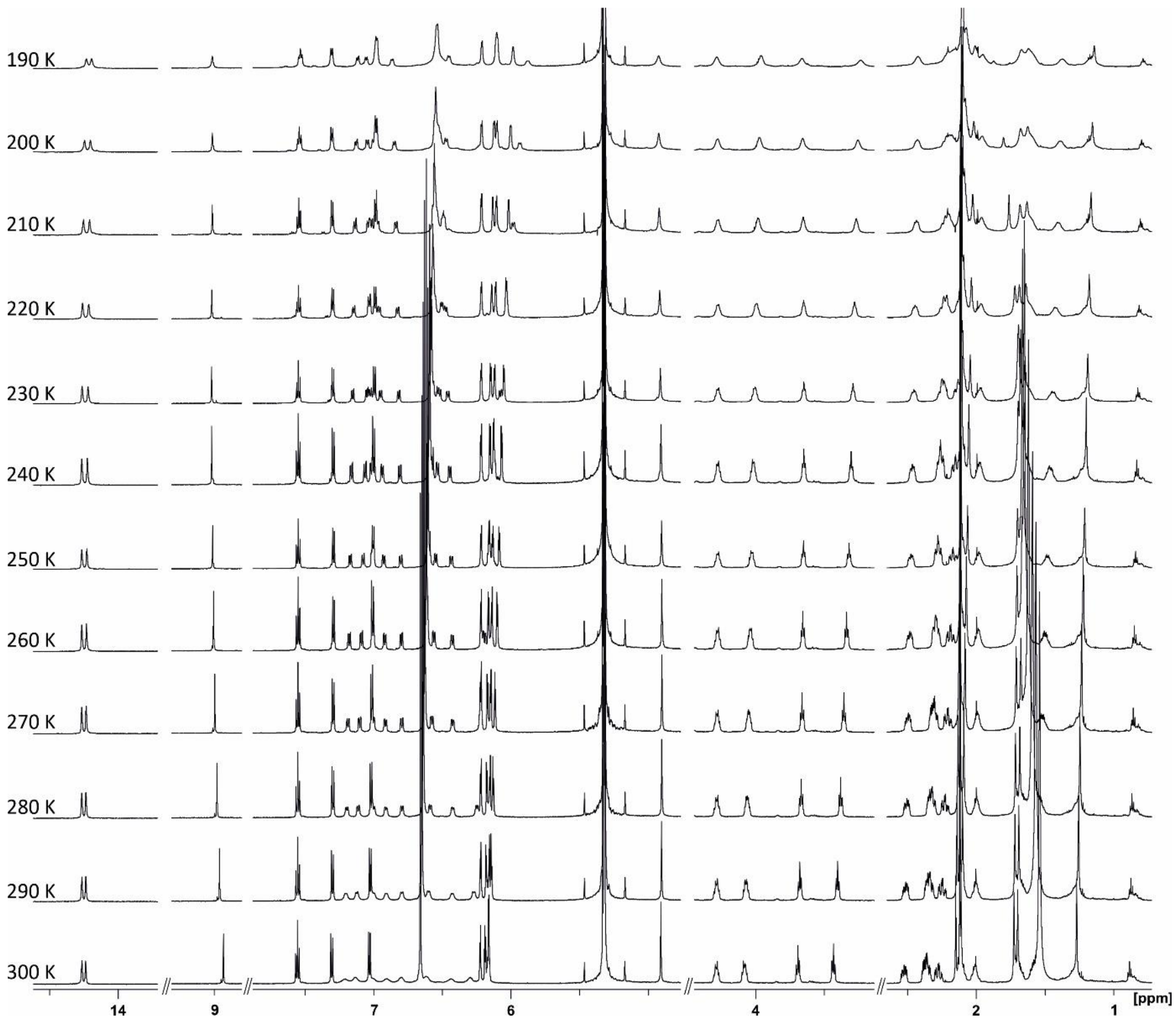

Figure S59. ^{1}H NMR spectra of **[3]cat$^{6}_{HH}$** recorded in the 300–190 K temperature range (600 MHz, CD_2Cl_2).

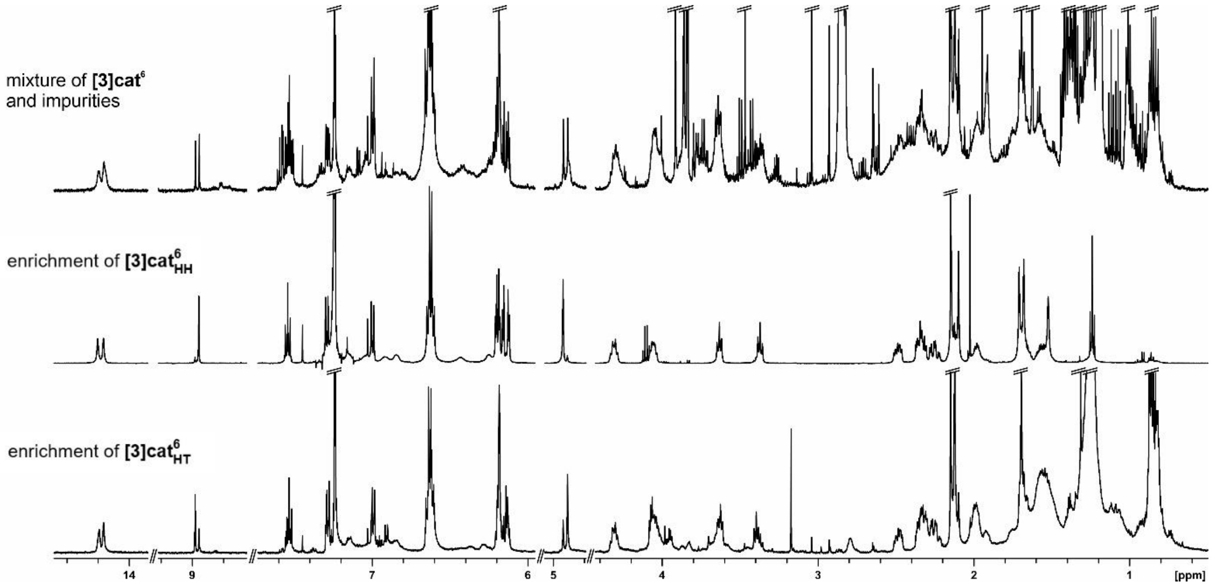

Figure S60. The comparison of the ^{1}H NMR spectra of a mixture of **[3]cat^6** isomers with the spectra recorded for samples enriched in the respective**[3]cat$^6_{HT}$** and **[3]cat$^6_{HH}$** species (500 MHz, $CDCl_3$, 300 K).

*Analytical data for **[3]cat$^6_{HT}$**:*

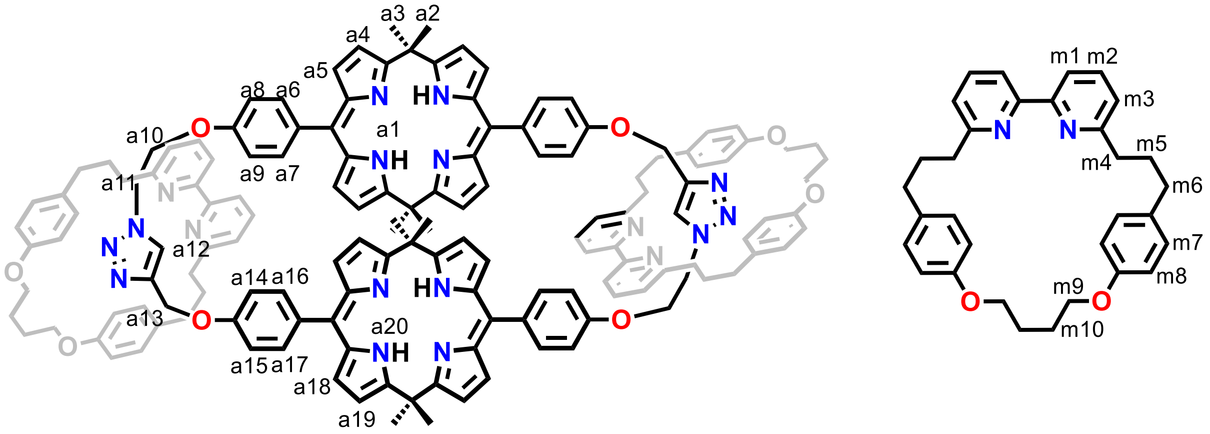

^{1}H NMR (500 MHz, $CDCl_3$, 300 K) δ (ppm): 14.19 (b, 2H, Ha1/Ha20), 14.16 (b, 2H, Ha1/Ha20), 8.98 (s, 2H, Ha12), 7.53 (t, 4H, ^3J = 7.7 Hz, Hm2), 7.28 (d, 4H, ^3J = 7.6 Hz, Hm1), 6.99 (d, 4H, ^3J = 7.7 Hz, Hm3), 6.65 (d, 8H, ^3J = 8.8 Hz, Hm7/Hm8), 6.61 (d, 8H, ^3J = 8.8 Hz, Hm7/Hm8), 6.20–6.11 (overlapping m, 16H, Ha, Ha5, Ha19, Ha18), 4.91 (s, 4H, Ha13), 3.63 (t, 4H, ^3J = 7.2 Hz, Ha11), 3.40 (t, 4H, ^3J = 7.0 Hz, Ha10), 2.12 (s, 12H, Ha2), 1.70 (s, 12H, Ha3). **HRMS** (ESI+, TOF) *m/z*: $[M+3H]^{3+}$ calcd. for $C_{146}H_{145}N_{18}O_8^{3+}$, 759.7169; found, 759.7216.

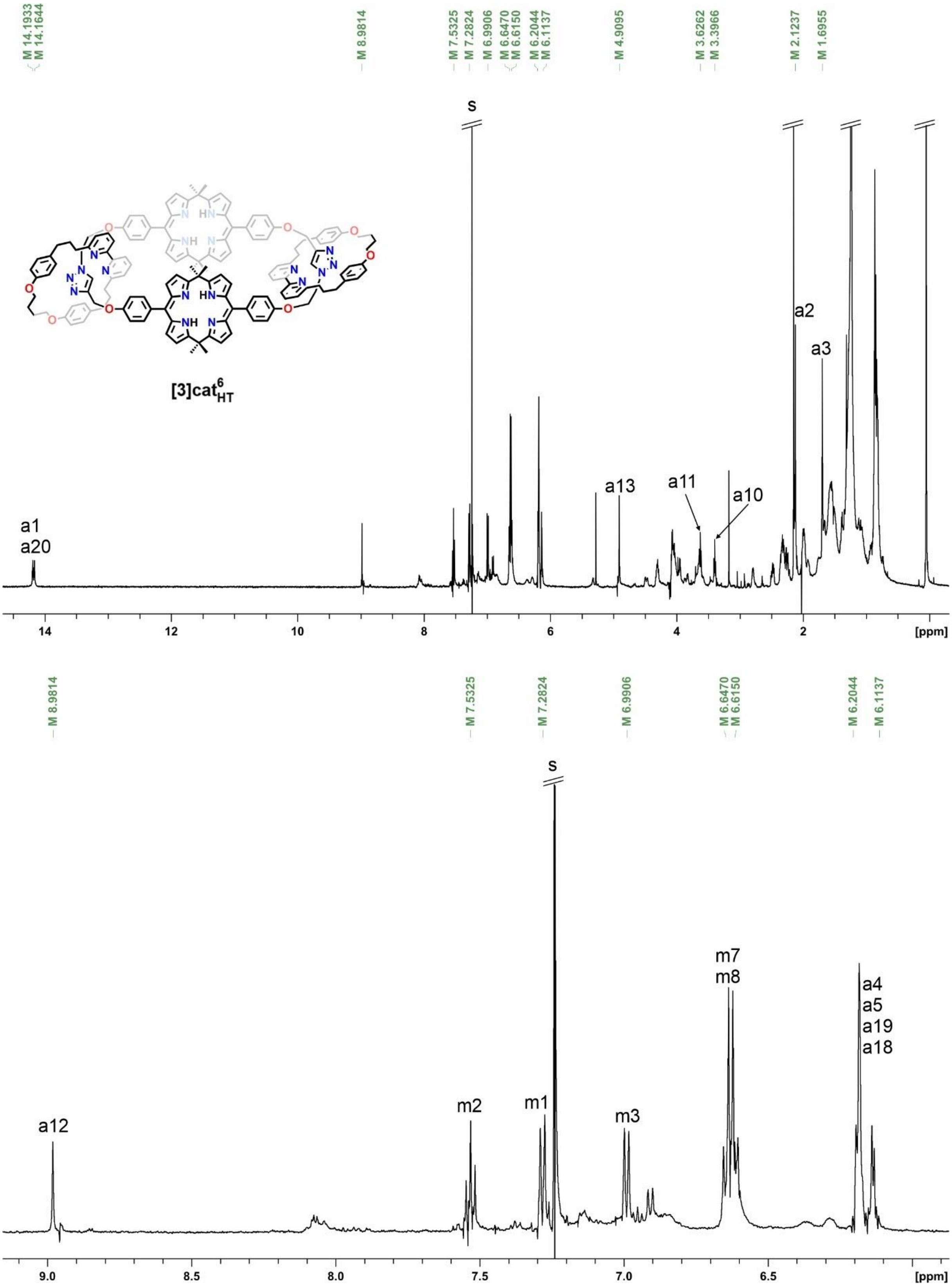

Figure S61. The spectrum of **[3]cat$^{6}_{HT}$** obtained by subtracting the spectrum of isomer **[3]cat$^{6}_{HH}$** from the spectrum of the sample enriched in the isomer **[3]cat$^{6}_{HT}$**.

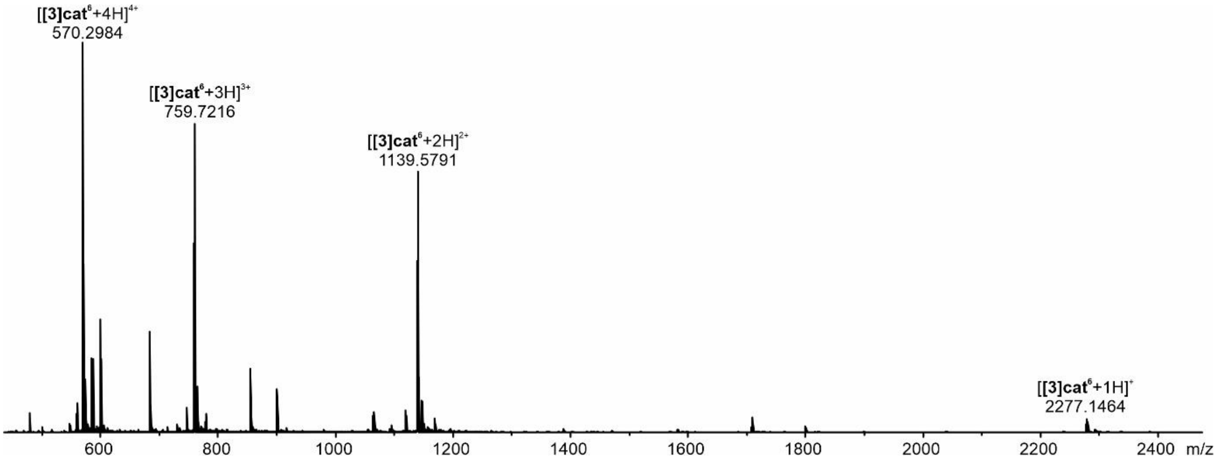

Figure S62. The ESI (TOF) mass spectrum of **[3]cat^{6}**.

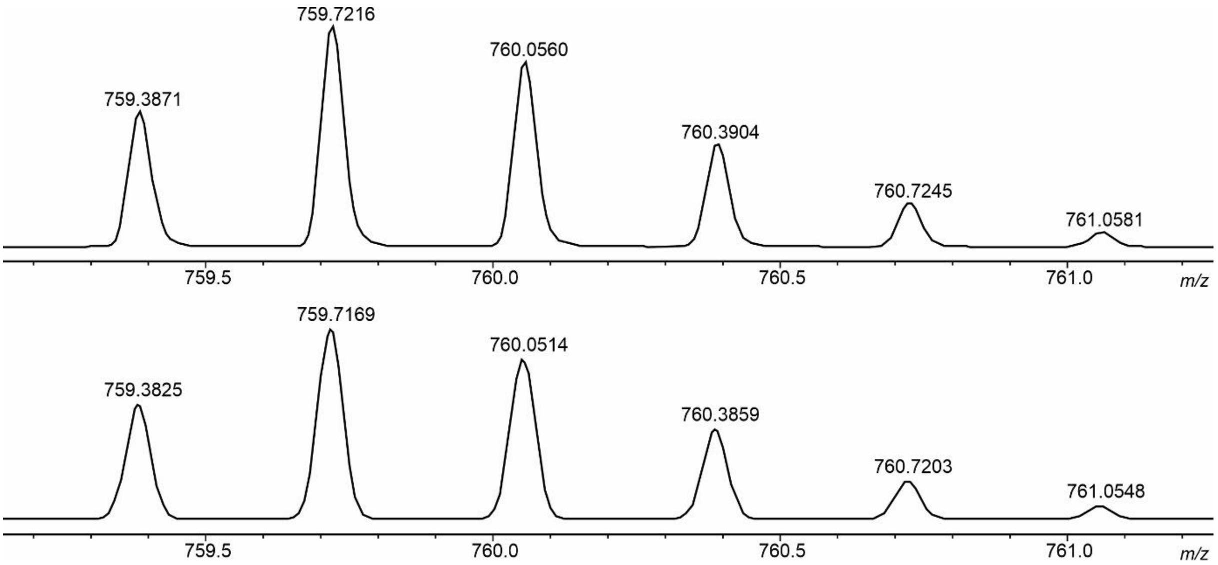

Figure S63. The ESI (TOF) mass spectrum of isomers of **[3]cat^{6}**. Top: experimental, bottom: simulated isotopic pattern.

Catenanes [3]cat^{7} and [4]cat^{7}

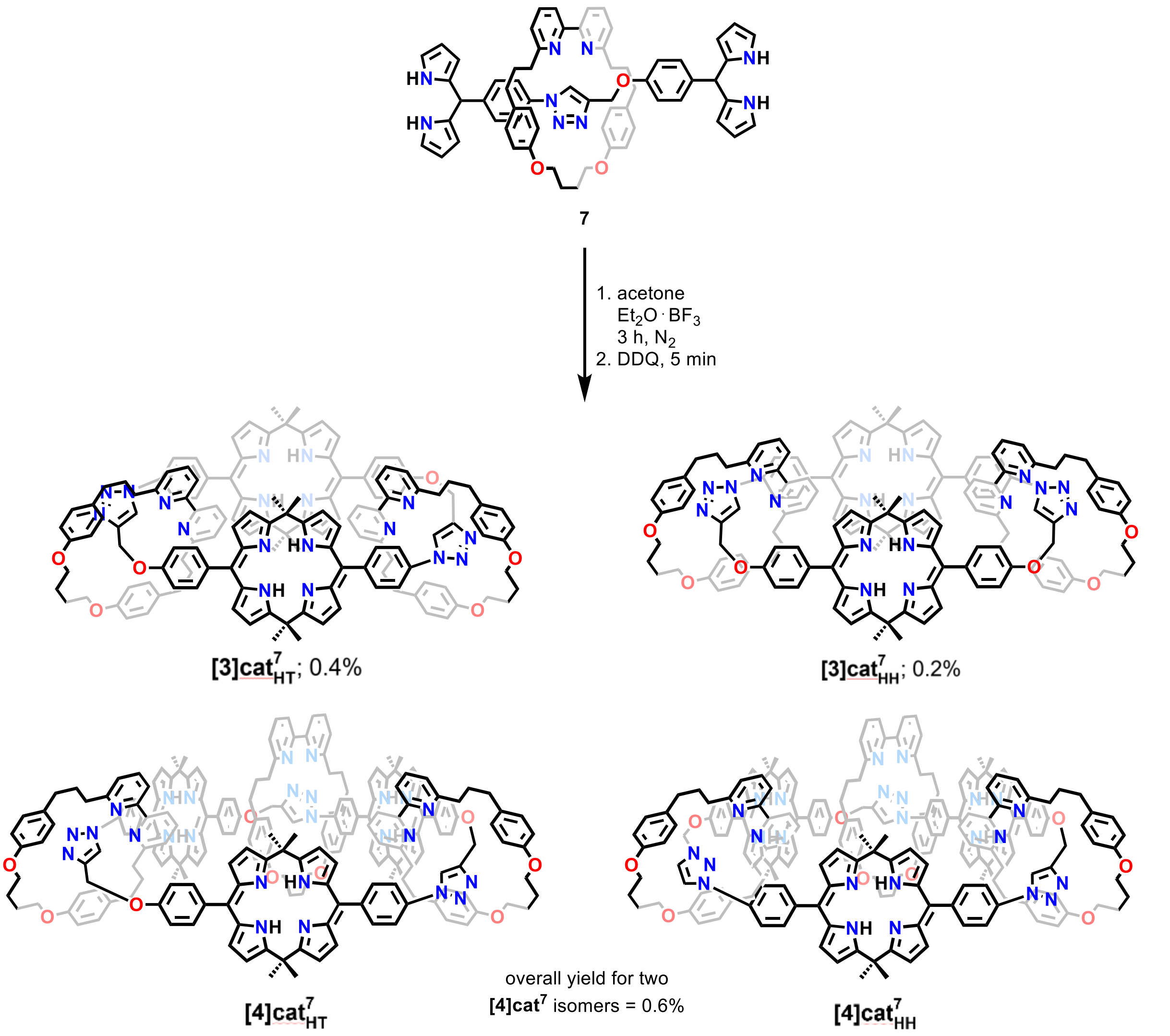

In a 100 mL round bottom flask, **7** (176 mg, 172.4 μmol) and acetone (60 mL) were introduced. The mixture was deoxygenated *via* nitrogen bubbling for 10 minutes. Then, $Et_2O·BF_3$ (56 μL, 454 μmol) was added *via* the syringe. The mixture was then stirred for 3 hours under a nitrogen atmosphere. After this time, the flask was opened, DDQ (140 mg, 616 μmol) was introduced, and the reaction was carried out for an additional 5 minutes. Then, the acid was quenched by adding TEA (0.5 mL). ***Three of the same reactions were prepared in parallel.*** The product mixtures were combined, filtrated through cotton wool and passed through a short column with deactivated aluminum oxide. Residues on the column were washed out with DCM:ethyl acetate 6:4. The solvent was removed under reduced pressure. The reddish oil was purified *via* column chromatography (silica gel). In the first column (DCM), the first fraction was removed, and the residue was eluted (DCM:ethyl acetate, 6:4). In the second chromatographic separation (silica gel, DCM:hexane 1:1, with 5-40% ethyl acetate gradient) the separation of

[3]cat$^{7}_{HT}$ and **[3]cat$^{7}_{HH}$** mixture from of **[4]cat$^{7}_{HT}$** and **[4]cat$^{7}_{HH}$** mixture was achieved. Both resulting mixtures of **[3]cat^{7}** and **[4]cat^{7}** isomers were further purified *via* TLC (10x20 cm silica gel plates, DCM:hexane 1:1, with 10% ethyl acetate gradient) for **[3]cat^{7}** and (DCM: hexane 1:1, with 15% ethyl acetate gradient) for **[4]cat^{7}**. [3]catenanes were eventually recrystallized from DCM/MeCN to provide **[3]cat$^{7}_{HT}$** (2.1 mg, 0.96 µmol, 0.4%) as an orange crystalline solid, and **[3]cat$^{7}_{HH}$** (0.9 mg, 0.4 µmol, 0.2%) as an orange crystalline solid. [4]catenane mixture was recrystallized from DCM/methanol to provide mixture of isomers **[4]cat$^{7}_{HT}$** and **[4]cat$^{7}_{HH}$** (3.6 mg, 1.1 µmol, 0.6%) as an orange crystalline solid. *Yields were calculated for assuming 528 mg of substrate* ***7****.*

Attempted separation of **[4]cat$^{7}_{HT}$** from **[4]cat$^{7}_{HH}$**:

HPLC resolutions were performed using the LaChrom Merck Hitachi I-7420 system with an eluent flow rate of 2 mL/min (DCM:*n*-hexane from 10:90 to 50:50) and Phenomenex Chirex 3010 column, Phenomenex Chirex 3014 column, and Phenomenex Chirex 3022 column. The isomers' separation via selective crystallisation was also tried, but did not result in the resolution of isomers due to their similar solubility in the tested organic solvents (DCM, MeOH, MeCN, *n*-hexane).

Catenane [3]cat$^{7}_{HT}$

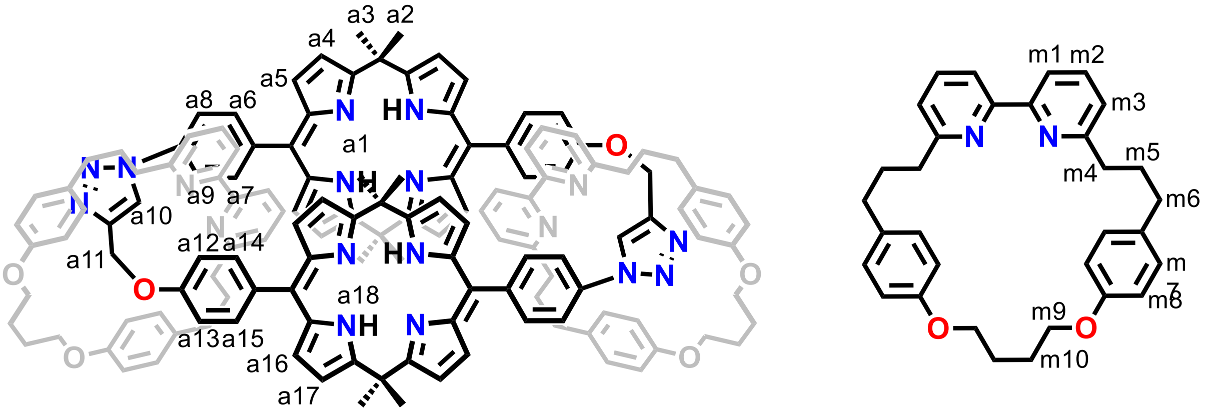

^{1}H NMR (500 MHz, $CDCl_3$, 300 K) δ (ppm): 14.67 (s, 2H, Ha1), 14.46 (s, 2H, Ha18), 8.88 (s, 2H, Ha10), 8.16 (d, 2H, ^{3}J = 8.3 Hz, Ha8/Ha9), 7.07 (overlapping m, 4H, Ha6/Ha7 and Ha8/Ha9), 6.78–6.69 (overlapping t and d, 8H, Hm2, Hm3), 6.56 (d, 4H, ^{3}J = 7.4 Hz, Hm1), 6.45 (d, 8H, ^{3}J = 8.6 Hz, Hm8), 6.33 (d, 8H, ^{3}J = 8.6 Hz, Hm7), 6.23 (d, 4H, ^{3}J = 4.2 Hz, Ha5), 6.10 (d, 2H, ^{3}J = 8.3 Hz, Ha6/Ha7), 5.95 (d, 4H, ^{3}J = 4.2 Hz, Ha4), 5.85 (d, 4H, ^{3}J = 4.2 Hz, Ha16), 5.78 (d, 4H, ^{3}J = 4.2 Hz, Ha17), 5.18 (s, 4H, Ha11), 4.57–4.49 (m, 4H, Hm9), 4.25–4.17

(m, 4H, Hm9), 2.29–1.93 (overlapping m, Hm4, Hm6, Hm10), 2.27 (s, 12H, Ha2), 1.70 (s, 12H, Ha3), 1.42–1.11 (overlapping m, Hm5).

^{13}C NMR (150 MHz, $CDCl_3$, 300 K) δ (ppm): 166.6, 164.9, 161.9, 158.0, 157.1, 156.8, 142.5, 140.1, 139.6, 136.4, 135.2, 132.4, 129.9, 128.8, 128.3, 127.8, 127.6, 123.2, 121.0, 120.9, 120.2, 120.1, 114.9, 114.0, 113.5, 66.1, 61.0, 38.4, 36.7, 34.9, 32.1, 30.9, 24.8, 23.6. *Not all ^{13}C NMR resonances were identified due to high broadening.*

HRMS (ESI+, TOF) *m/z*: $[M+2H]^{2+}$ calcd. for $C_{142}H_{136}N_{18}O_6^{2+}$, 1095.0455; found, 1095.0568.

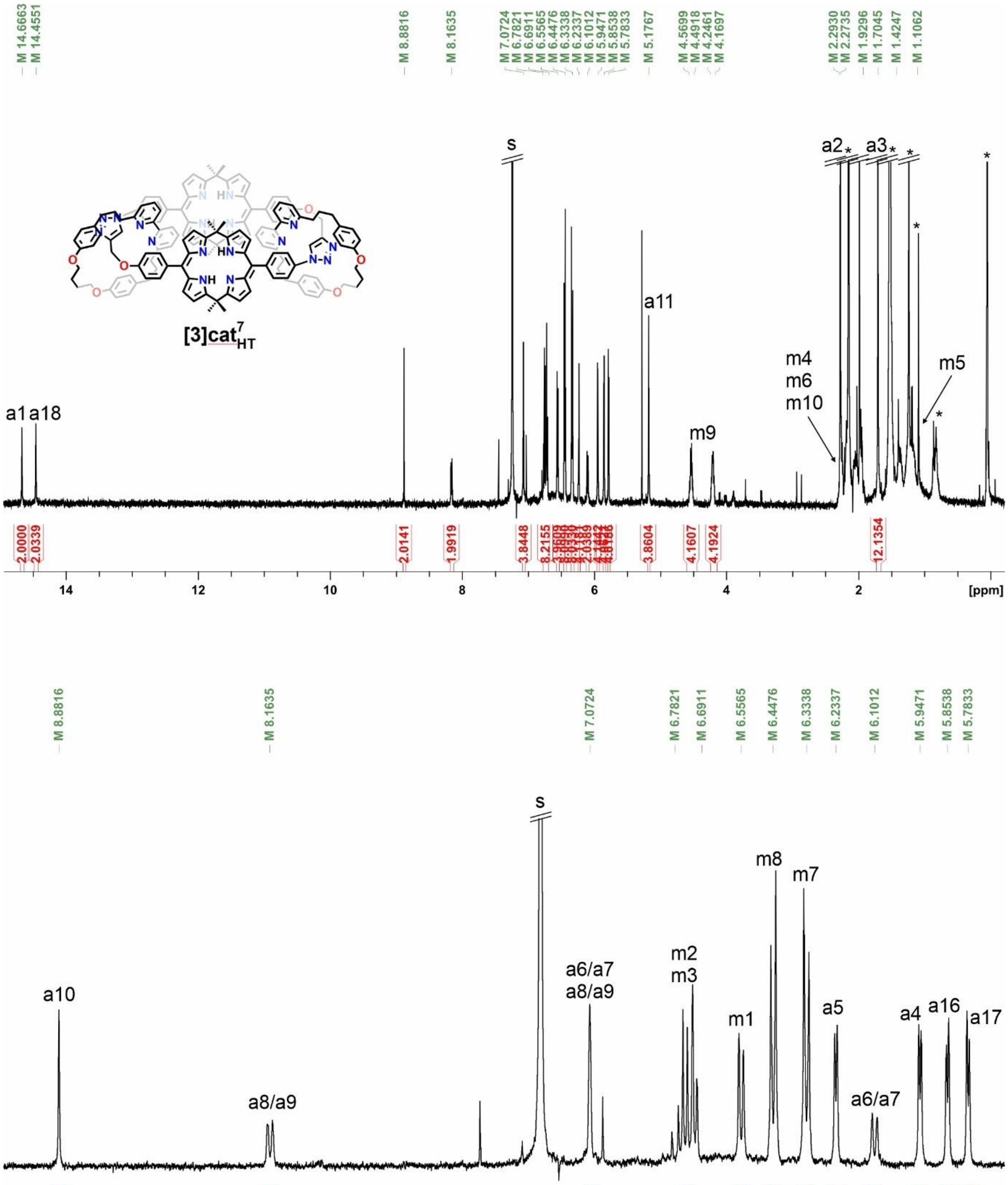

Figure S64. The ^{1}H NMR spectrum of **[3]cat$^{7}_{HT}$** (500 MHz, CDCl$_3$, 300 K).

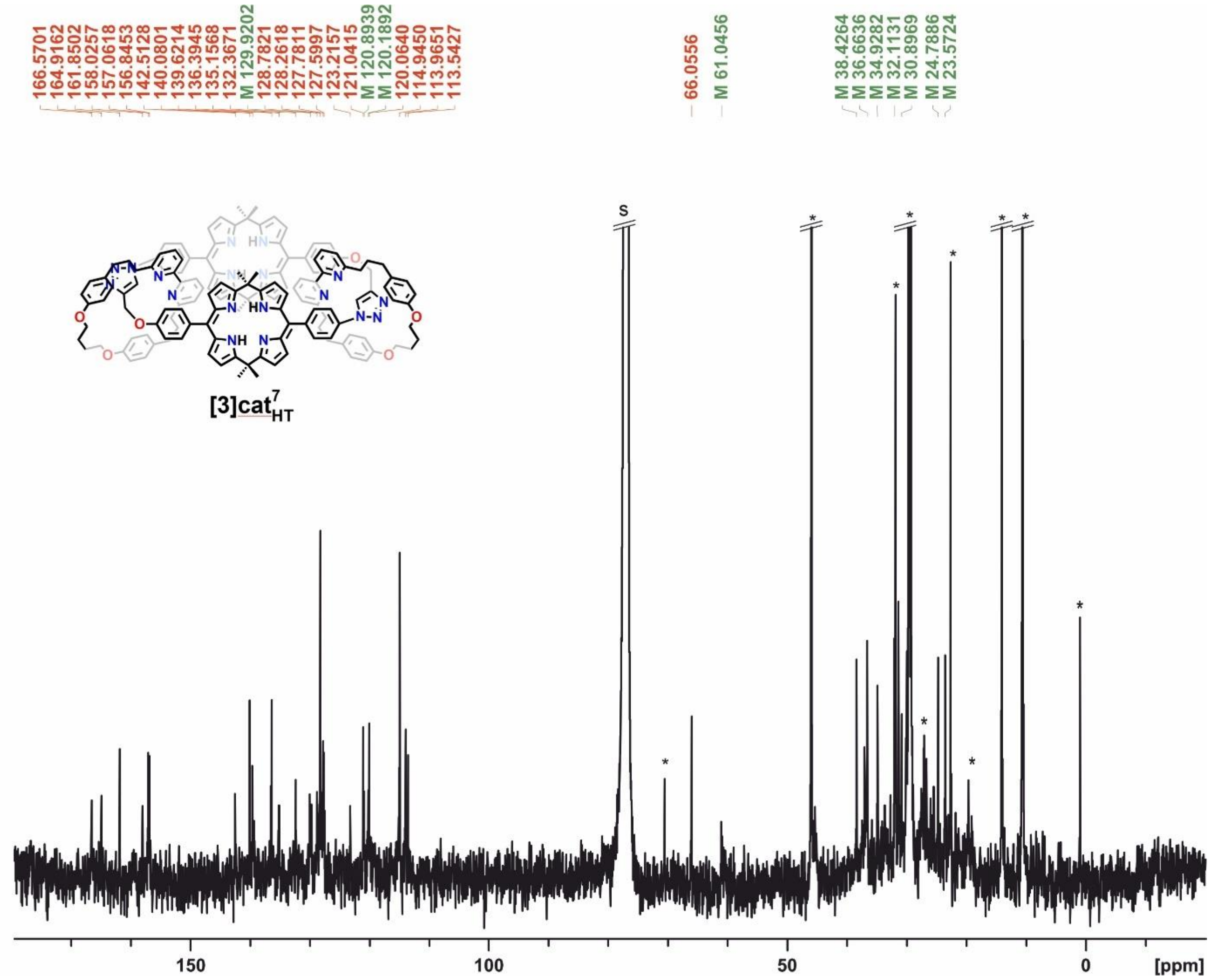

Figure S65. The ^{13}C NMR spectrum of **[3]cat$^{7}_{HT}$** (150 MHz, $CDCl_3$, 300 K). Impurities (mainly triethylamine and H grease) were labeled with asterisks.

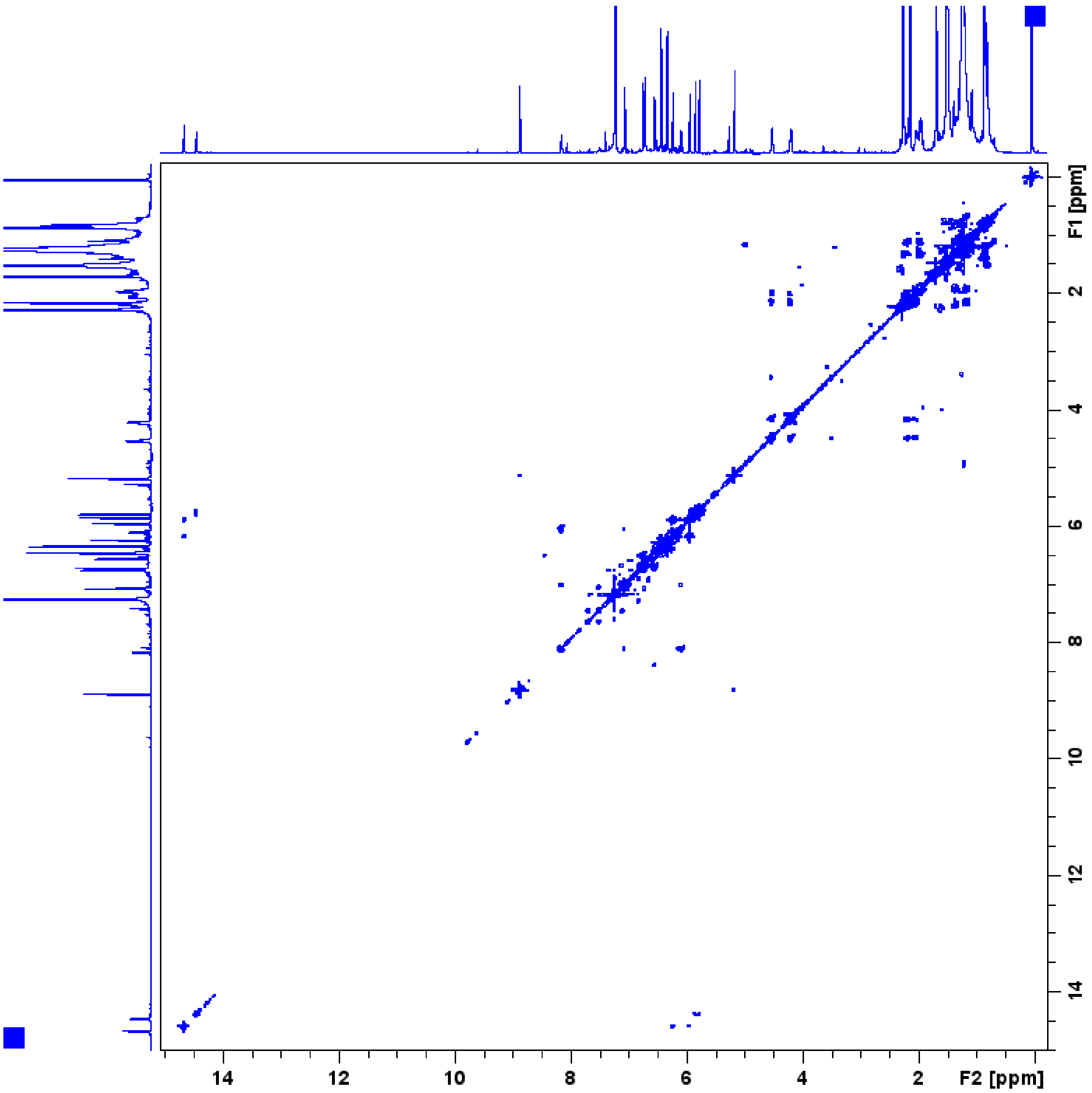

Figure S66. The ^{1}H-^{1}H COSY NMR spectrum of **[3]cat$^{7}_{HT}$** (600 MHz, $CDCl_3$, 300 K).

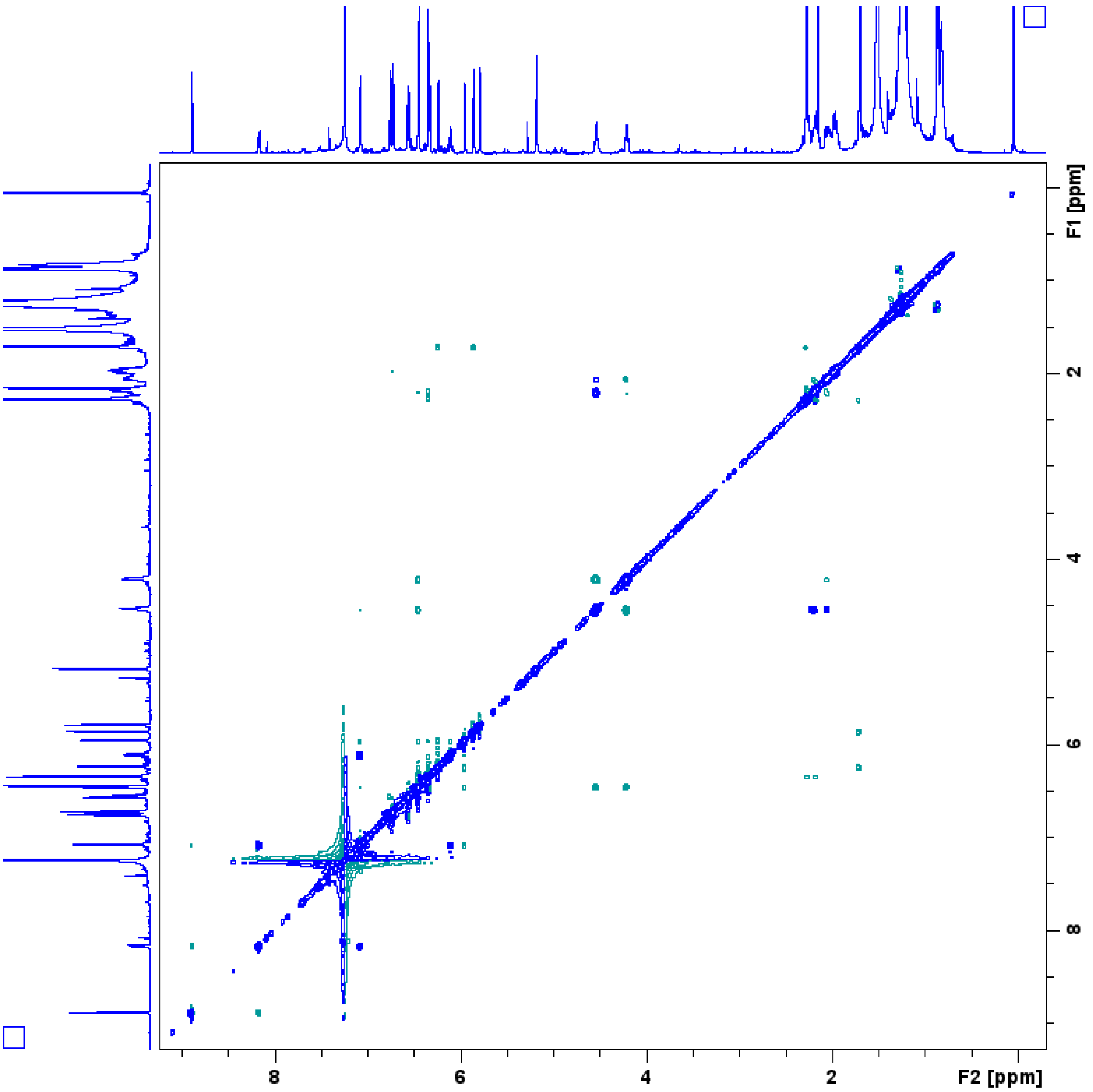

Figure S67. The ^{1}H-^{1}H ROESY NMR spectrum of **[3]cat$^{7}_{HT}$** (600 MHz, $CDCl_3$, 300 K).

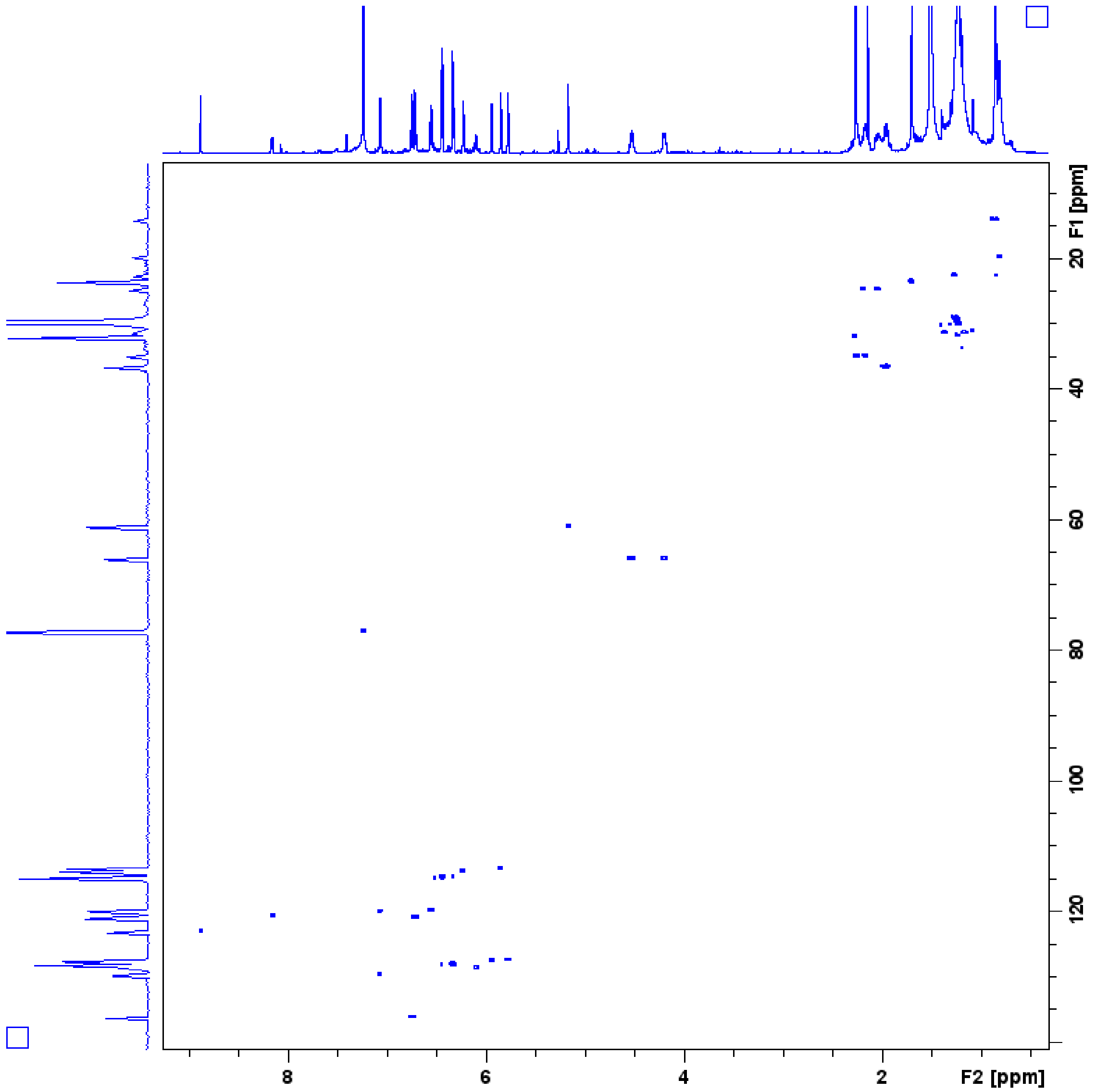

Figure S68. The ^{1}H-^{13}C HSQC NMR spectrum of **[3]cat$^{7}_{HT}$** (600 MHz, $CDCl_3$, 300 K).

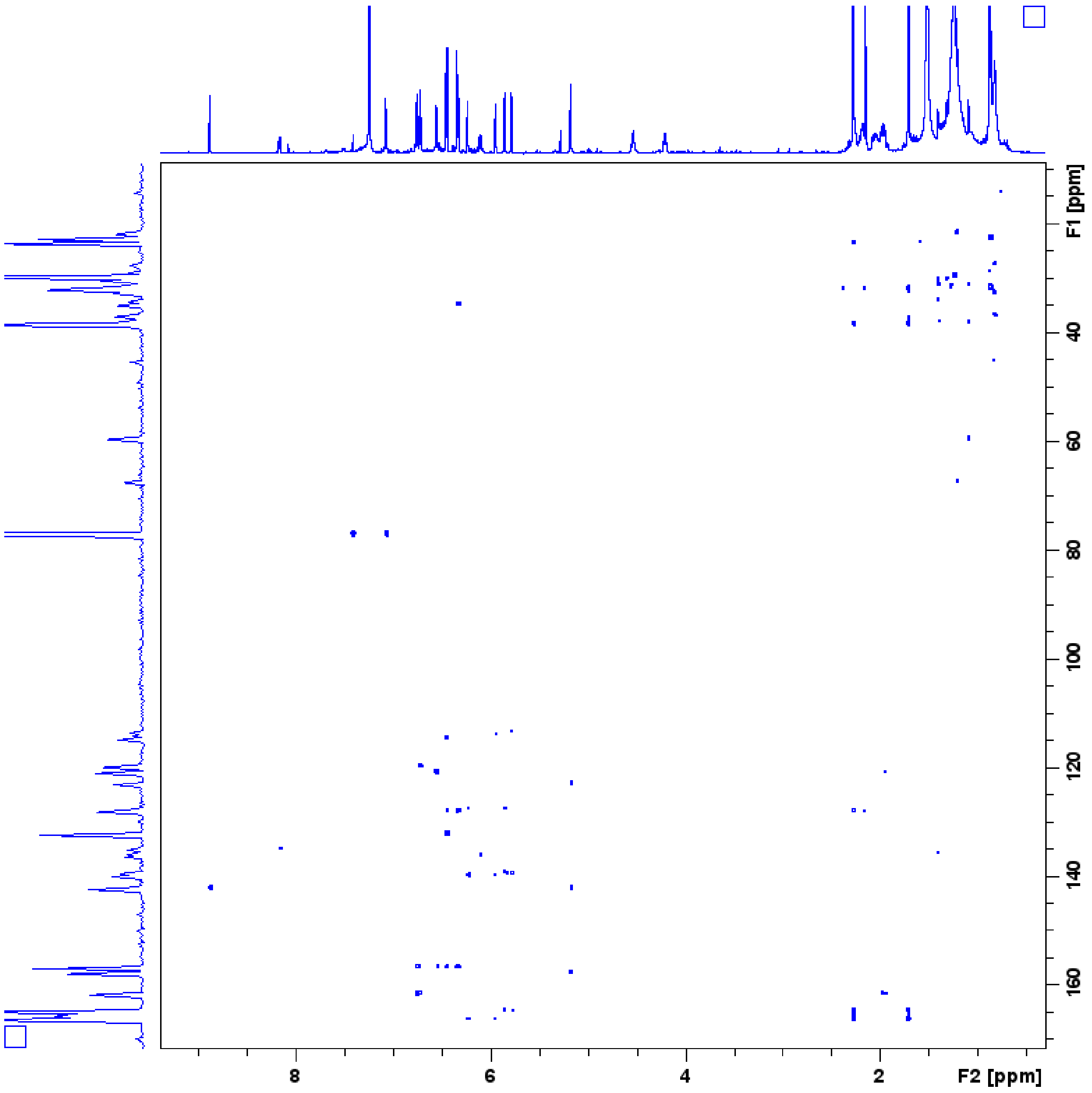

Figure S69. The ^{1}H-^{13}C HMBC NMR spectrum of **[3]cat$^{7}_{HT}$** (600 MHz, $CDCl_3$, 300 K).

Catenane [3]cat$^{7}_{HH}$

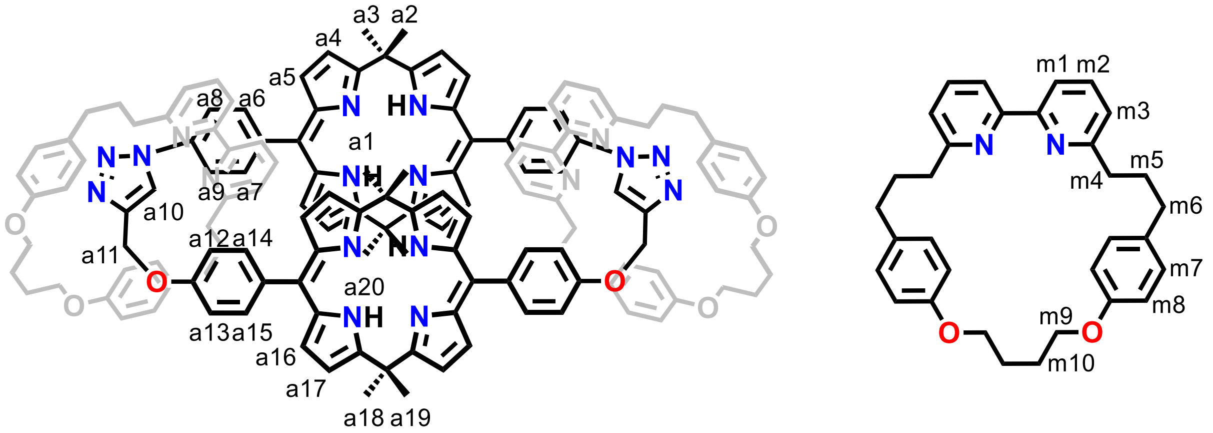

^{1}**H NMR** (600 MHz, $CDCl_3$, 270 K) δ (ppm): 14.42 (s, 2H, Ha1), 14.27 (s, 2H, Ha20), 9.66 (s, 2H, Ha10), 7.84 (d, 2H, ^{3}J = 7.6 Hz, Ha8/Ha9), 7.39 (d, 2H, ^{3}J = 7.7 Hz, Ha8/Ha9), 7.31–7.22 (overlapping m, 2H, Ha6/ Ha7), 7.17–7.13 (overlapping m, Ha14/ Ha15, Hm1, Hm2), 6.78–6.70 (overlapping m, 6H, Ha12/Ha13, Hm3), 6.67–6.58 (overlapping m, 4H, Ha6/Ha7 and Ha12/Ha13), 6.52 (d, 8H, ^{3}J = 8.4 Hz, Hm8), 6.36 (d, ^{3}J = 8.4 Hz, Hm7), 6.24–6.19 (overlapping m, 8H, Ha16, Ha17), 6.11–6.07 (overlapping m, 8H, Ha4, Ha5), 5.98 (b, 2H, Ha14/Ha15), 4.86 (s, 4H, Ha11), 4.56–4.47 (m, 4H, Hm9), 4.25–4.17 (m, 4H, Hm9), 2.35–2.12 (overlapping m, 12H, Hm6, Hm10), 2.30 (s, 6H, Ha2), 2.12–1.91 (overlapping, 12H, Hm4, Hm10), 2.06 (s, 6H, Ha19), 1.75 (s, 6H, Ha18), 1.65 (s, 6H, Ha3), 1.35–1.06 (overlapping, 8H, Hm5).

^{13}**C NMR** (125 MHz, $CDCl_3$, 300 K) δ (ppm): 166.4, 164.3, 162.7, 158.5, 157.2, 156.6, 142.2, 140.1, 139.9, 136.8, 132.3, 128.33, 128.25, 127.5, 124.2, 121.9, 119.7, 115.1, 114.1, 113.5, 66.2, 60.3, 38.6, 38.2, 36.7, 35.0, 33.8, 31.9, 24.8, 24.3, 23.2, 22.7.

Not all ^{13}C NMR resonances were identified due to high broadening.

HRMS (ESI+, TOF) *m/z*: $[M+2H]^{2+}$ calcd. for $C_{142}H_{136}N_{18}O_6^{2+}$, 1095.0455; found, 1095.0568.

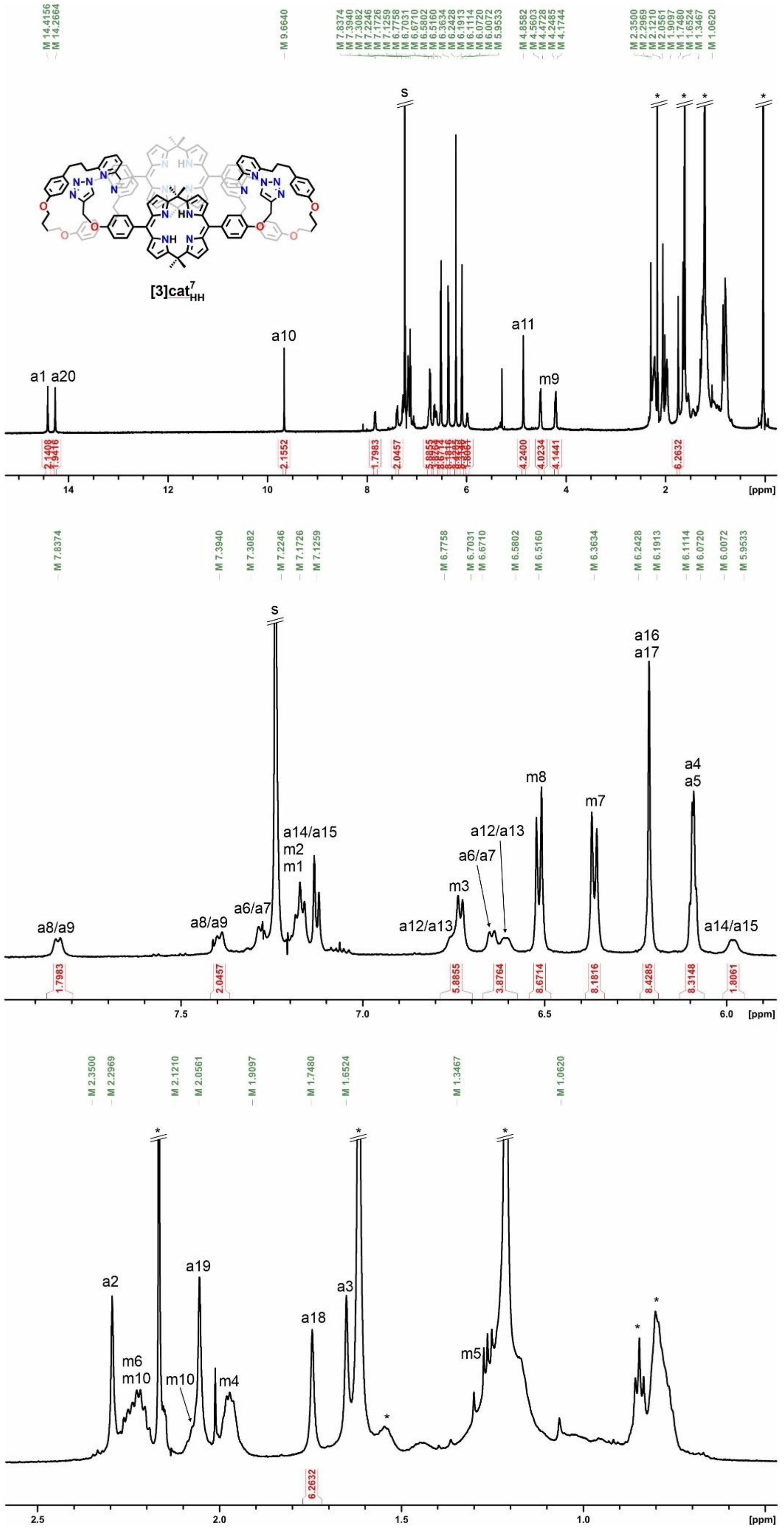

Figure S70. The ^{1}H NMR spectrum of **[3]cat$^{7}_{HH}$** (600 MHz, $CDCl_3$, 270 K).

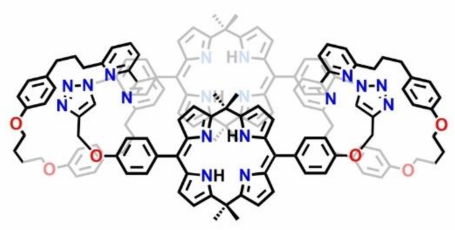

[3]cat$^{7}_{HH}$

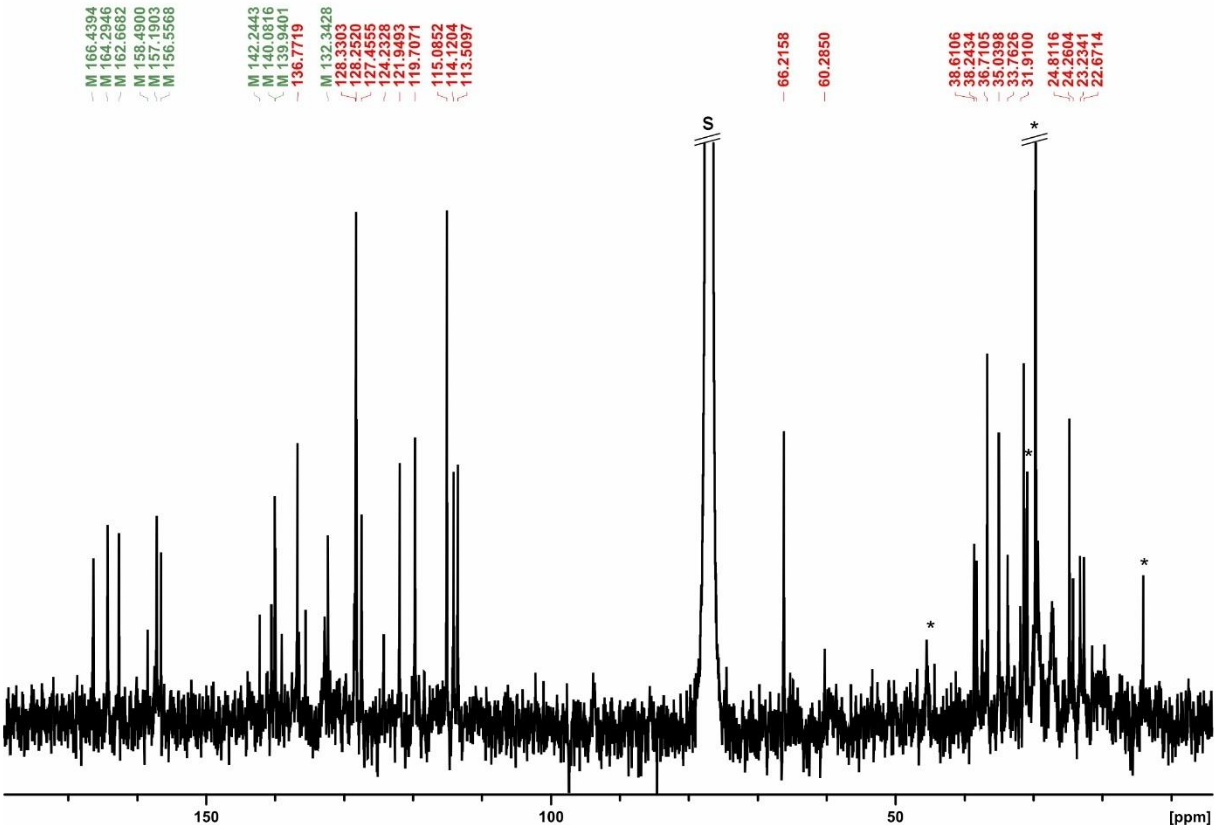

Figure S71. The ^{13}C NMR spectrum of **[3]cat$^{7}_{HH}$** (125 MHz, $CDCl_3$, 300 K). Impurities (mainly acetone and H grease) were labeled with asterisks.

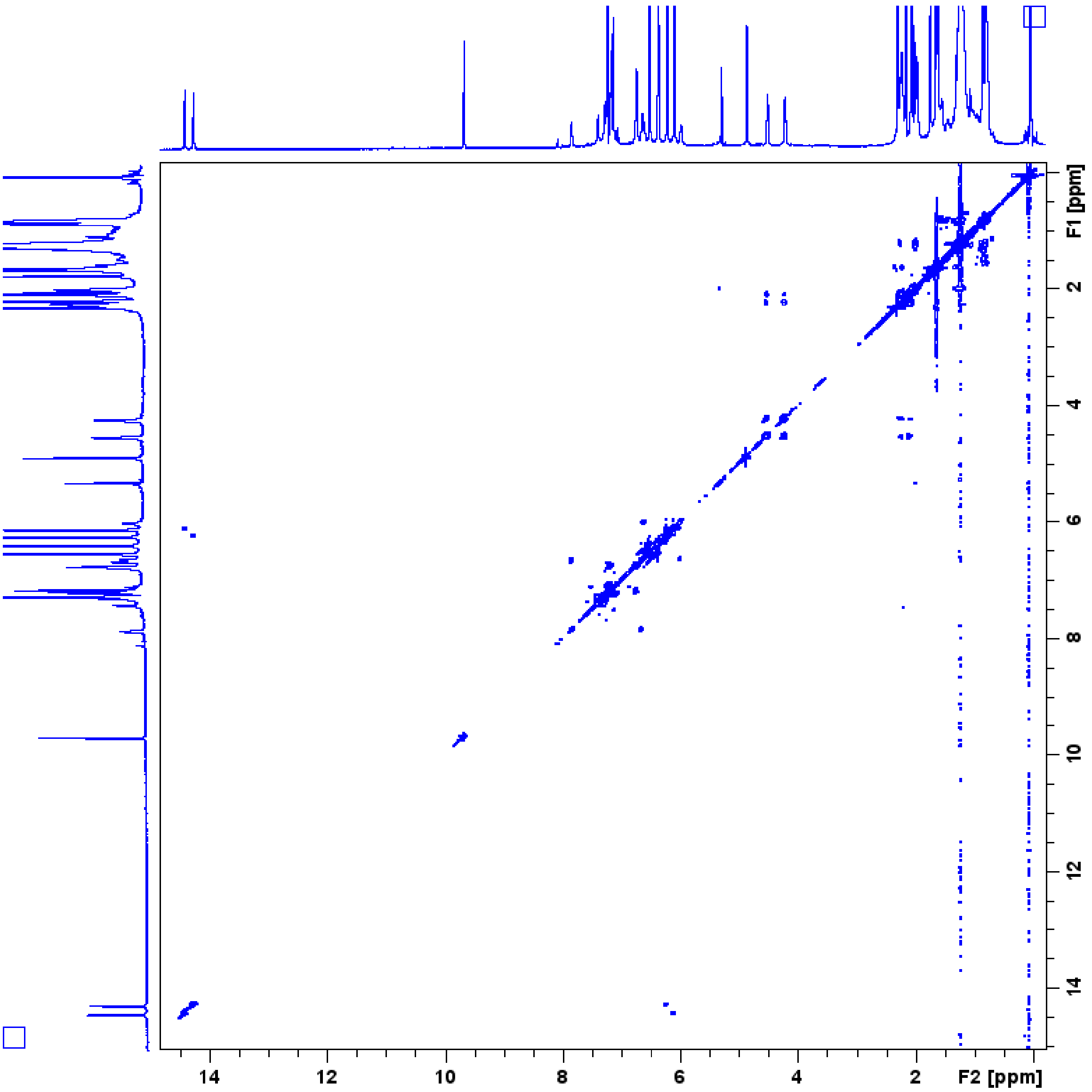

Figure S72. The ^{1}H-^{1}H COSY NMR spectrum of **[3]cat$^{7}_{HH}$** (600 MHz, $CDCl_3$, 270 K).

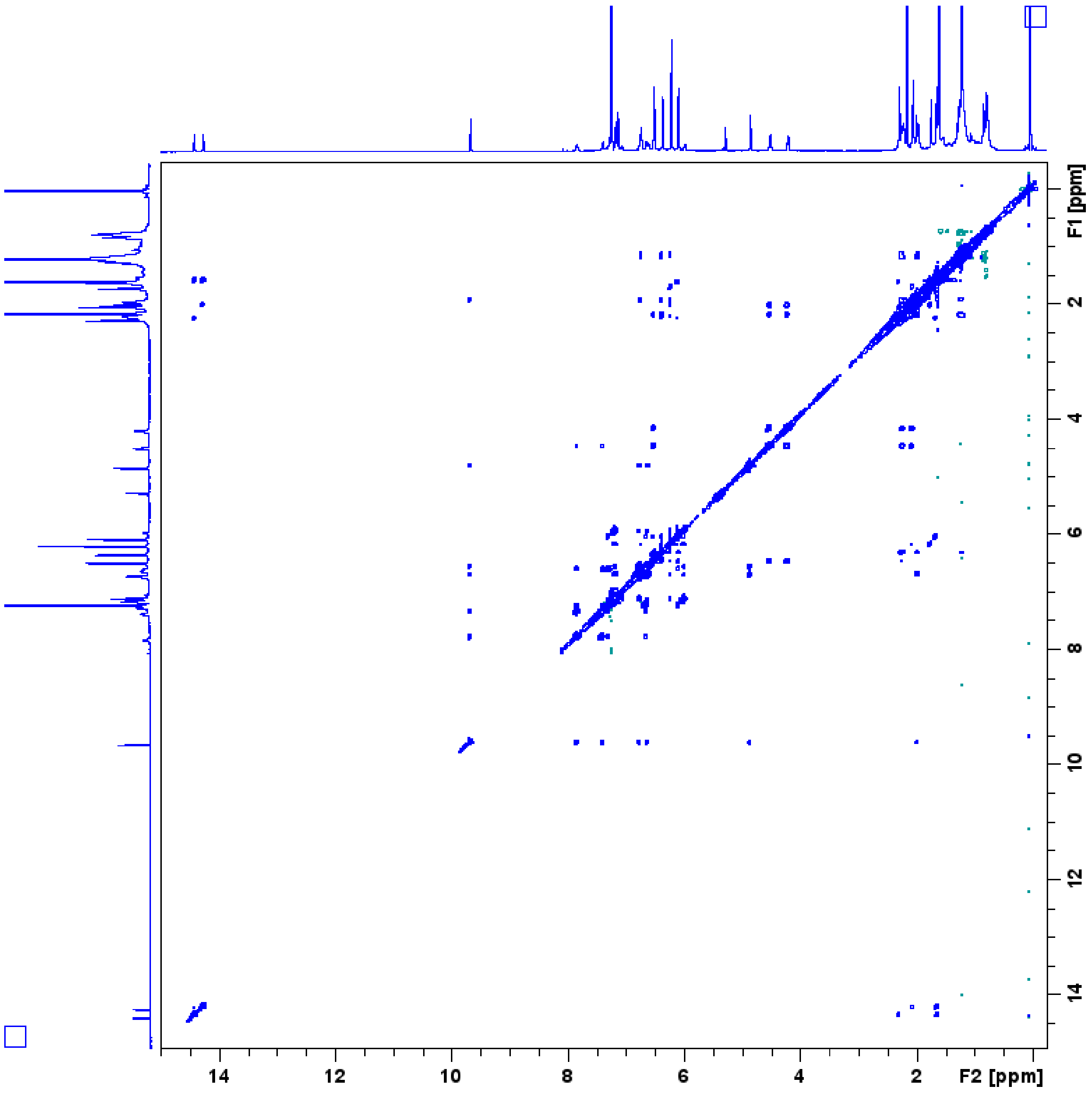

Figure S73. The ^{1}H-^{1}H NOESY NMR spectrum of **[3]cat$^{7}_{HH}$** (600 MHz, $CDCl_3$, 270 K).

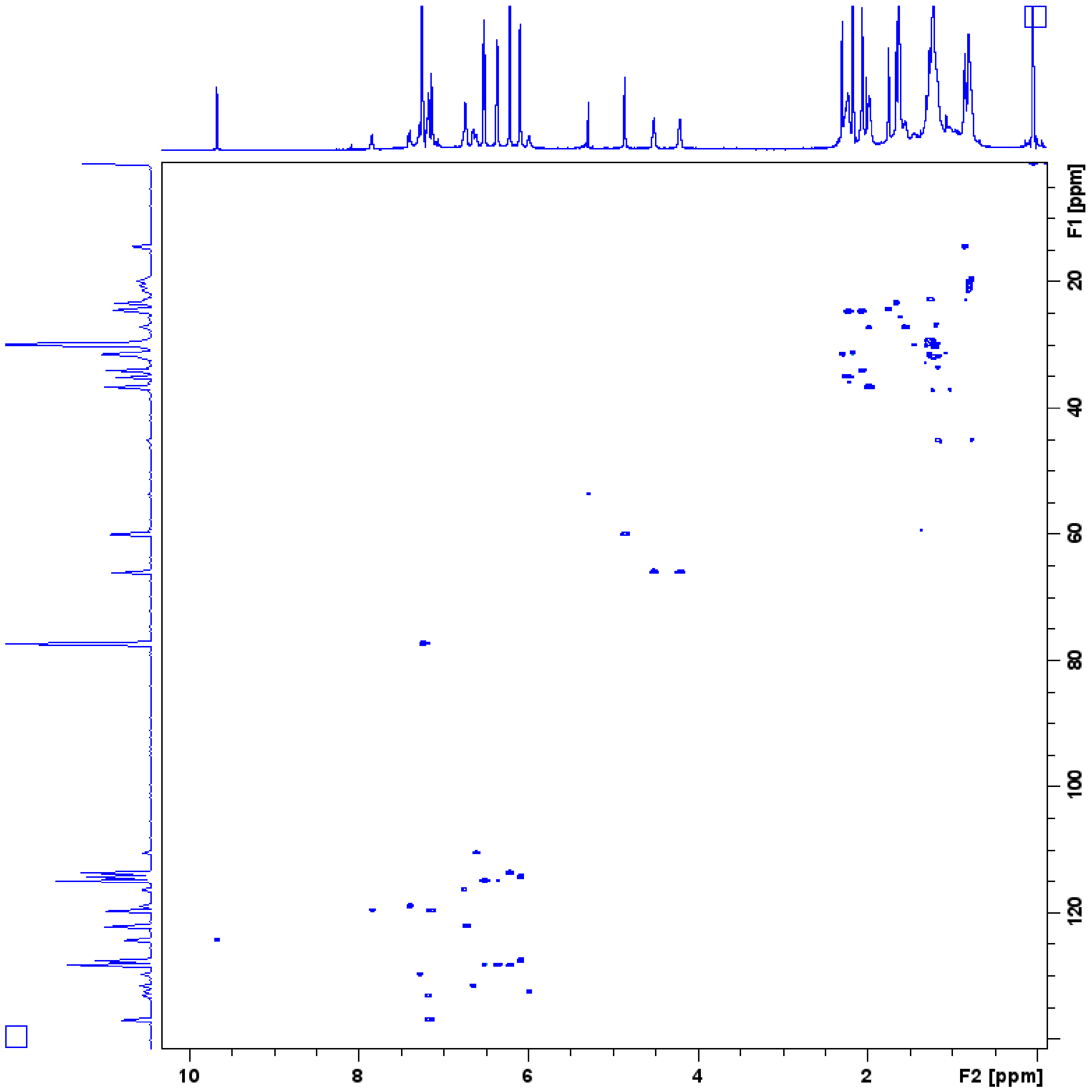

Figure S74. The ^{1}H-^{13}C HSQC NMR spectrum of **[3]cat$^{7}_{HH}$** (600 MHz, $CDCl_3$, 270 K).

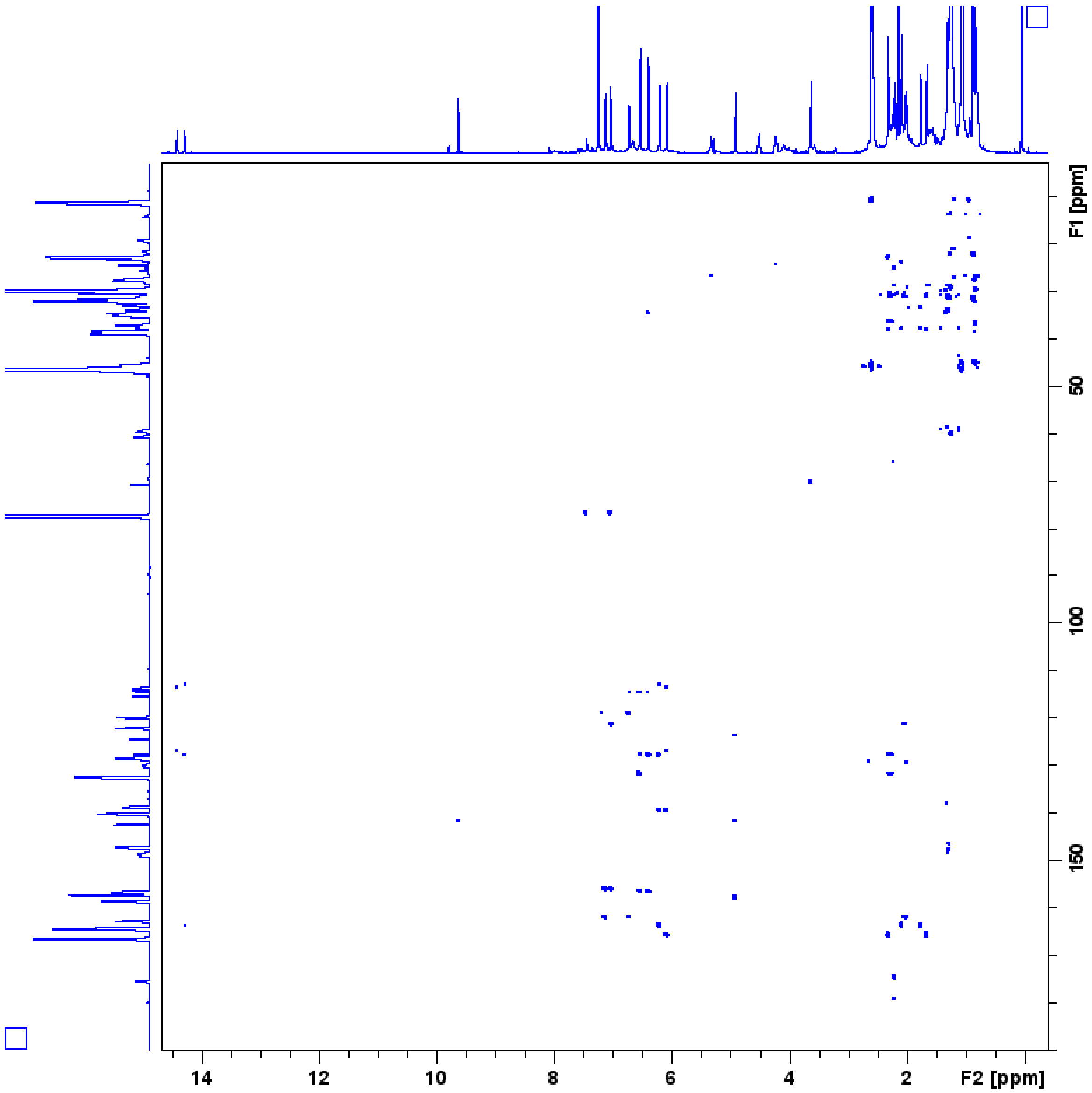

Figure S75. The ^{1}H-^{13}C HMBC NMR spectrum of **[3]cat$^{7}_{HH}$** (500 MHz, CDCl3, 300 K).

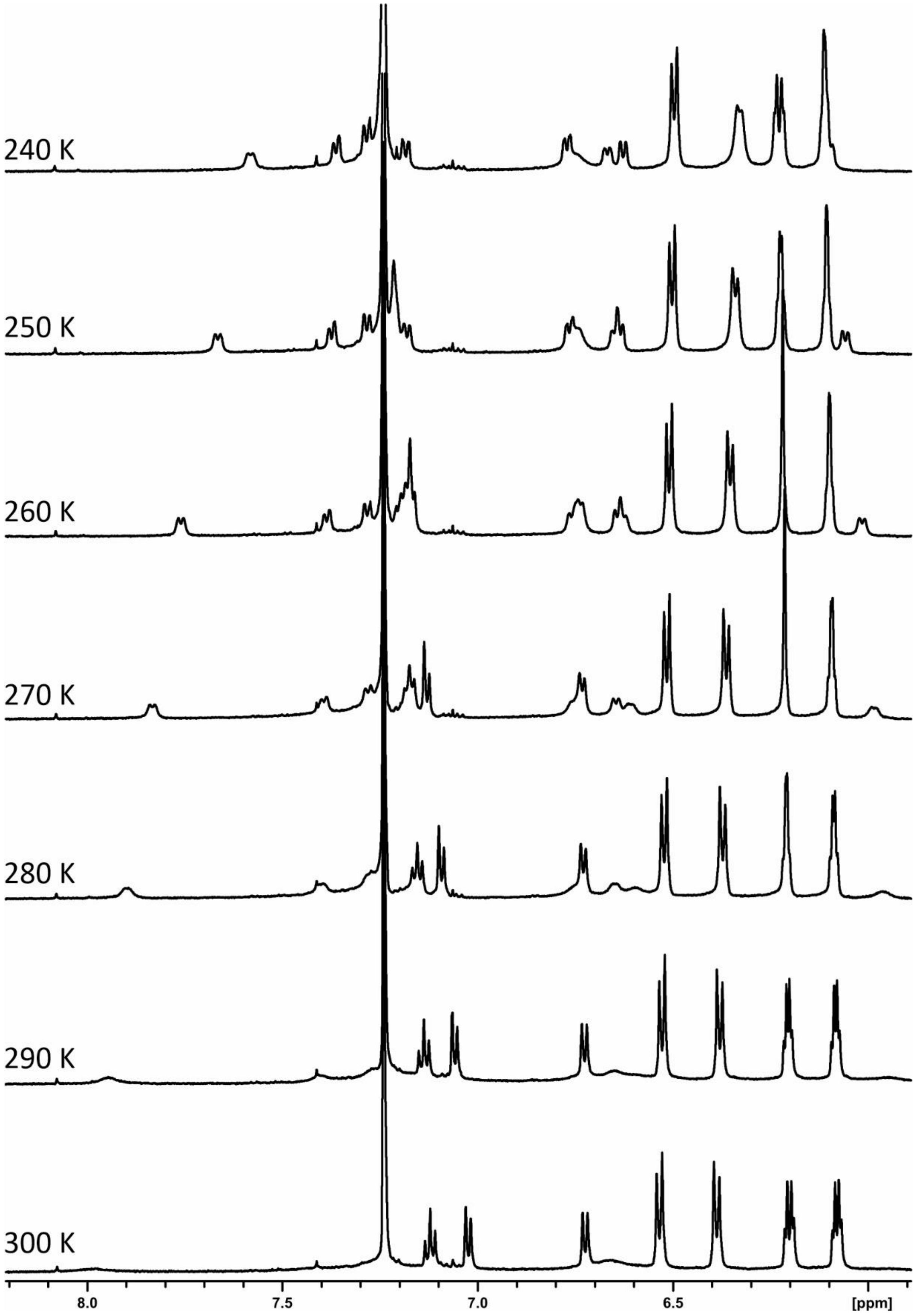

Figure S76. ^{1}H NMR spectra of **[3]cat$^{7}_{HH}$** recorded in the 300–240 K temperature range (600 MHz, $CDCl_3$).

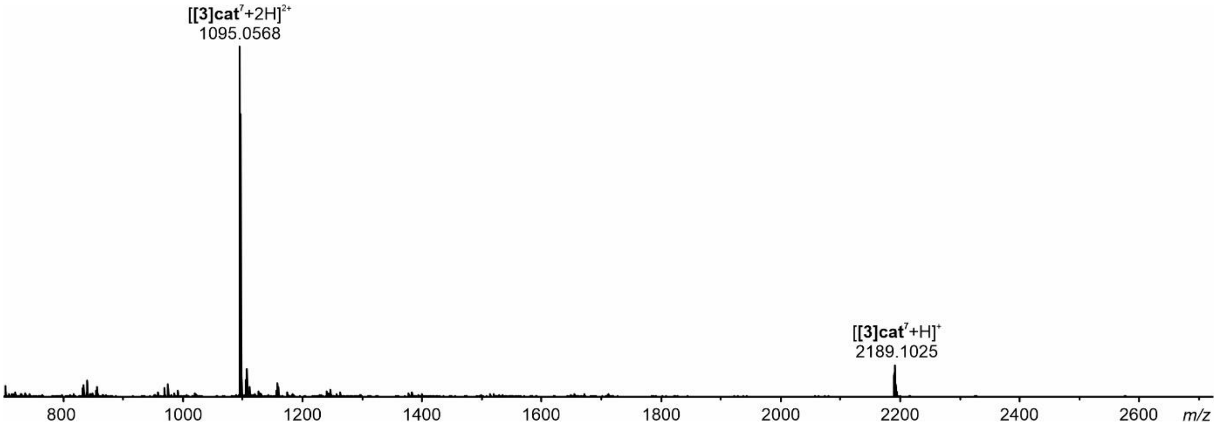

Figure S77. The ESI (TOF) mass spectrum of **[3]cat^{7}**.

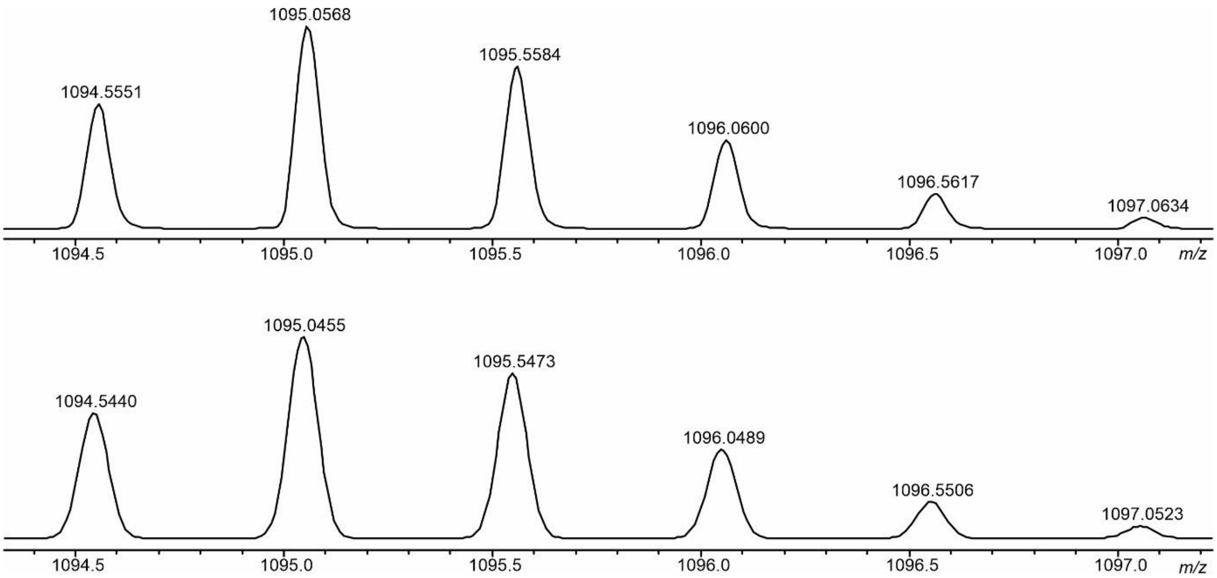

Figure S78. The ESI (TOF) mass spectrum of **[3]cat^{7}**. Top: experimental, bottom: simulated isotopic pattern.

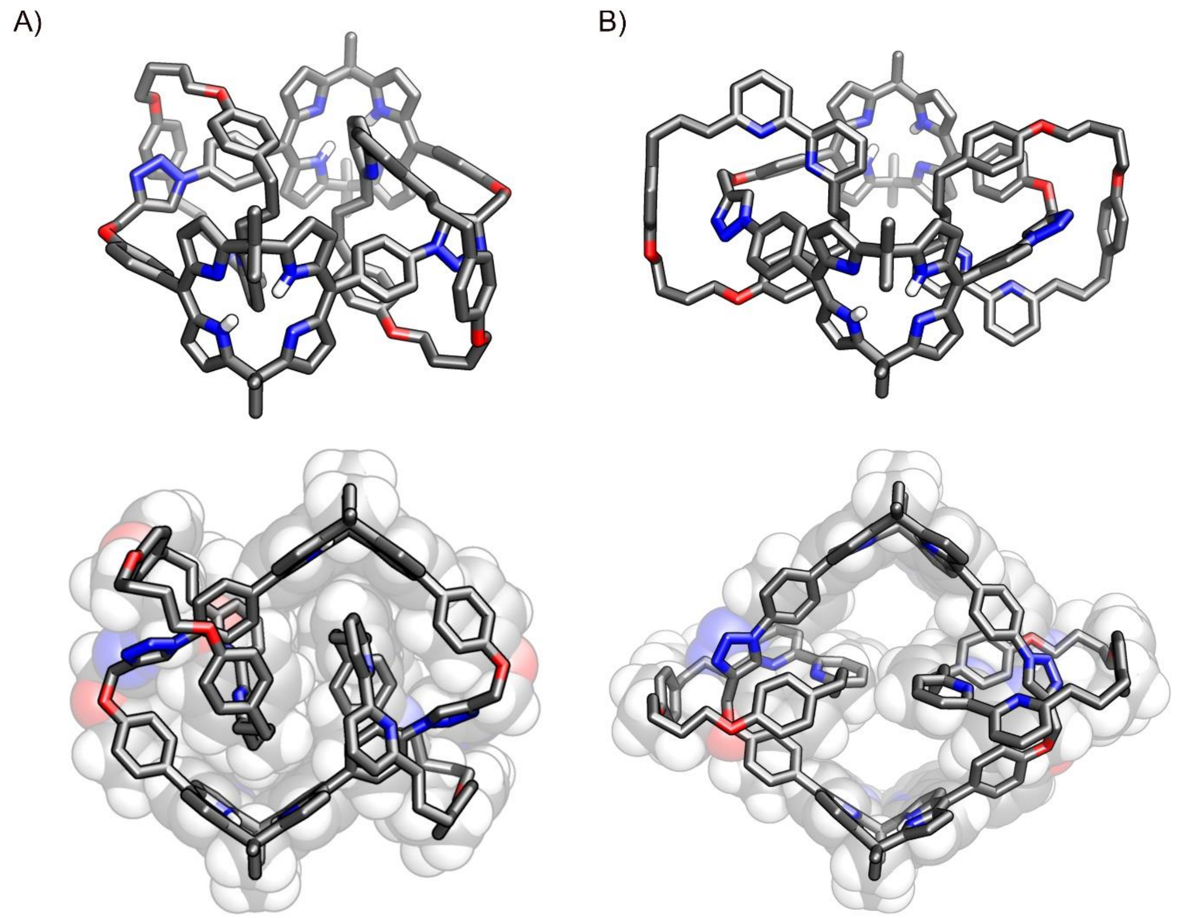

Figure S79. The X-ray molecular structure of A) **[3]cat$^{7}_{HT}$**, and B) **[3]cat$^{7}_{HH}$**. Protons, except for those in the cavity of calix[4]phyrin, were omitted for clarity in stick projection.

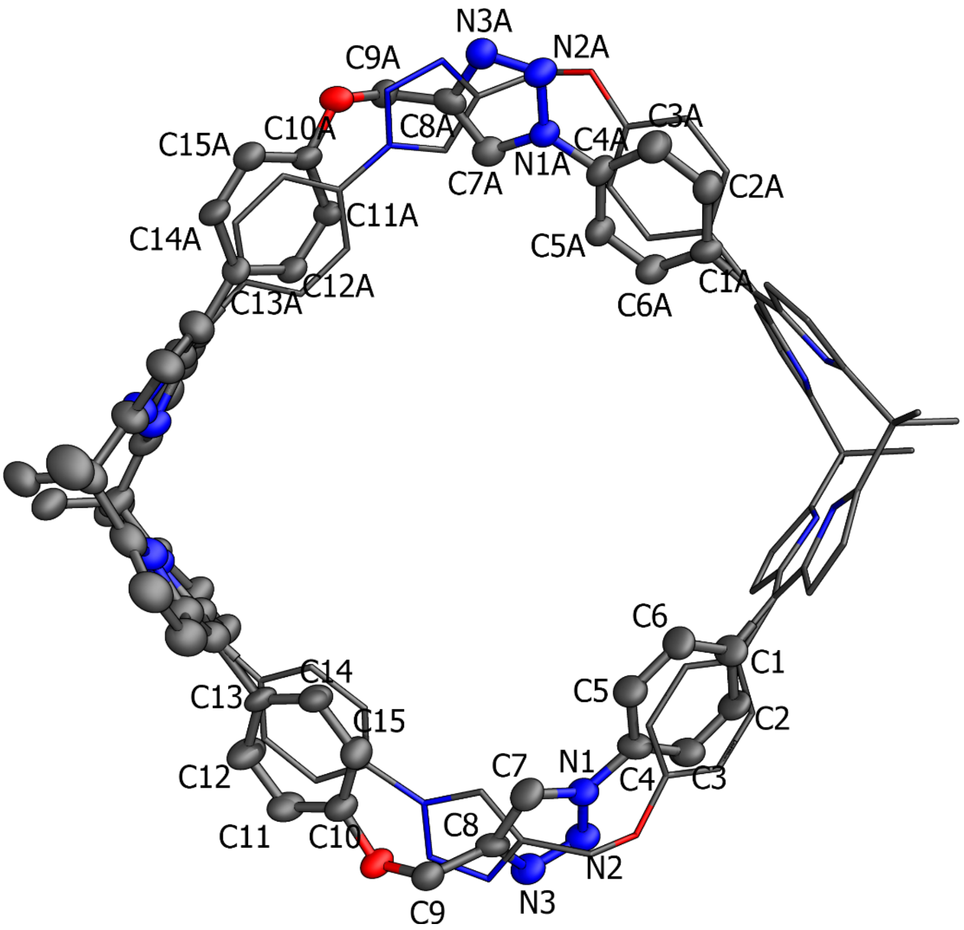

Figure S80. The crystal structure of) **[3]cat$^{7}_{HH}$**. Only the calix[4]phyrin-embedded macrocycle has been shown. Protons have been omitted for clarity. The atoms were depicted with thermal ellipsoids at 50% probability level. The part arising from the symmetry operation (-X, 2-Y, 2-Z) was depicted as sticks for clarity. Only the disordered atoms were labeled.

Catenane [4]cat^{7}

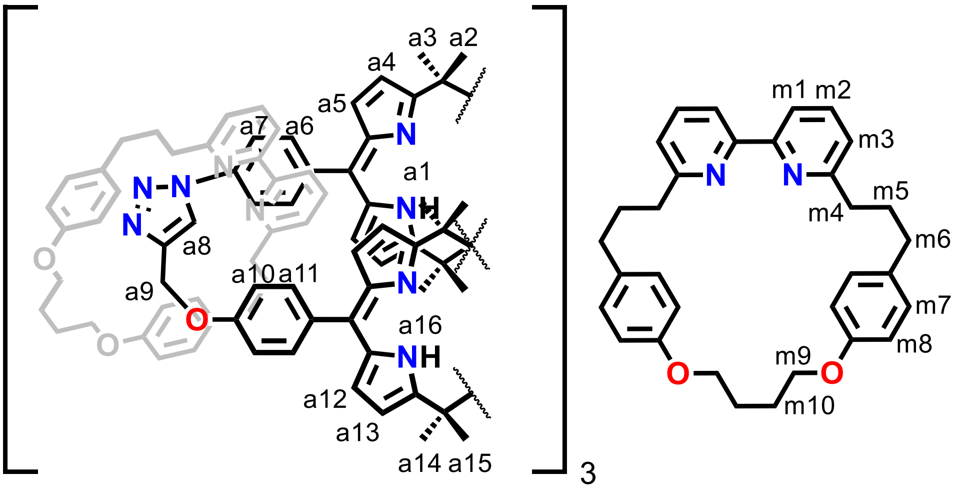

^{1}**H NMR** (500 MHz, CDCl$_3$, 300 K) δ (ppm) for mixture of isomers **[4]cat^{7}**: 14.18–14.08 (Ha1, Ha16), 9.81–9.77 (Ha8), 7.64–7.57 (Hm2), 7.52–7.43 (Ha7), 7.42–7.37 (6H, Hm1), 7.19–7.13 (6H, Ha11), 7.04–6.95 (Ha6, Hm3), 6.76–6.69 (Ha10), 6.48–6.42 (Hm8), 6.36–6.31 (Hm7),

6.25–6.21 (Ha4), 6.18–6.15 (Ha5), 6.13–6.06 (Ha12, Ha13), 4.95–4.86 (Ha9), 4.54–4.46 (Hm9), 4.16–4.06 (Hm9), 2.36–2.08 (Hm4, Hm6, Hm10), 2.08–1.79 (Ha2, Ha3, Ha14, Ha15, Hm10), 1.62–1.33 (Hm5).

^{13}C NMR (125 MHz, $CDCl_3$, 300 K) δ (ppm): 165.5, 164.8, 162.8, 158.8, 157.3, 157.0, 143.3, 140.4, 140.2, 137.0, 136.7, 135.6, 132.6, 132.0, 130.4, 129.4, 128.5, 128.1, 124.4, 121.7 (2 overlapping signals), 119.9, 119.6, 114.9, 114.1, 113.9 (2 overlapping signals), 66.2, 61.5, 38.2, 36.8, 34.9, 32.0, 24.8.

Not all ^{13}C NMR resonances were identified due to high broadening.

HRMS (ESI+, TOF) *m/z*: $[M+3H]^{3+}$ calcd. for $C_{213}H_{204}N_{27}O_9^{3+}$, 1095.2127; found, 1095.2054.

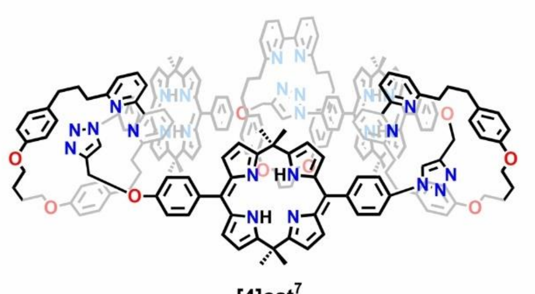

[4]cat$^{7}_{HT}$

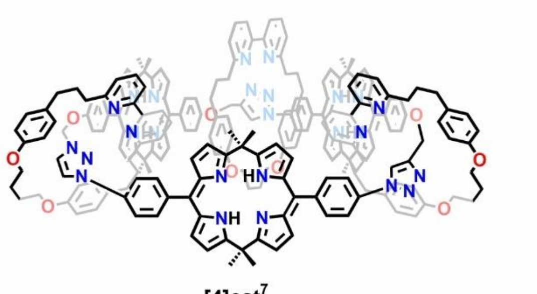

[4]cat$^{7}_{HH}$

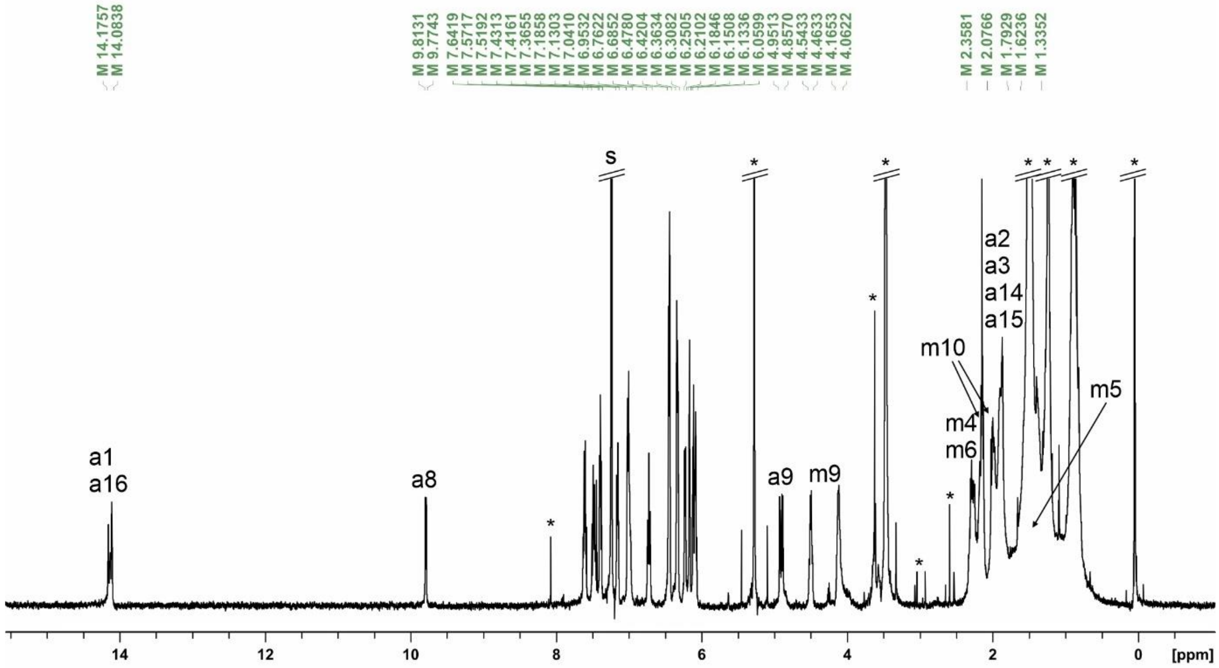

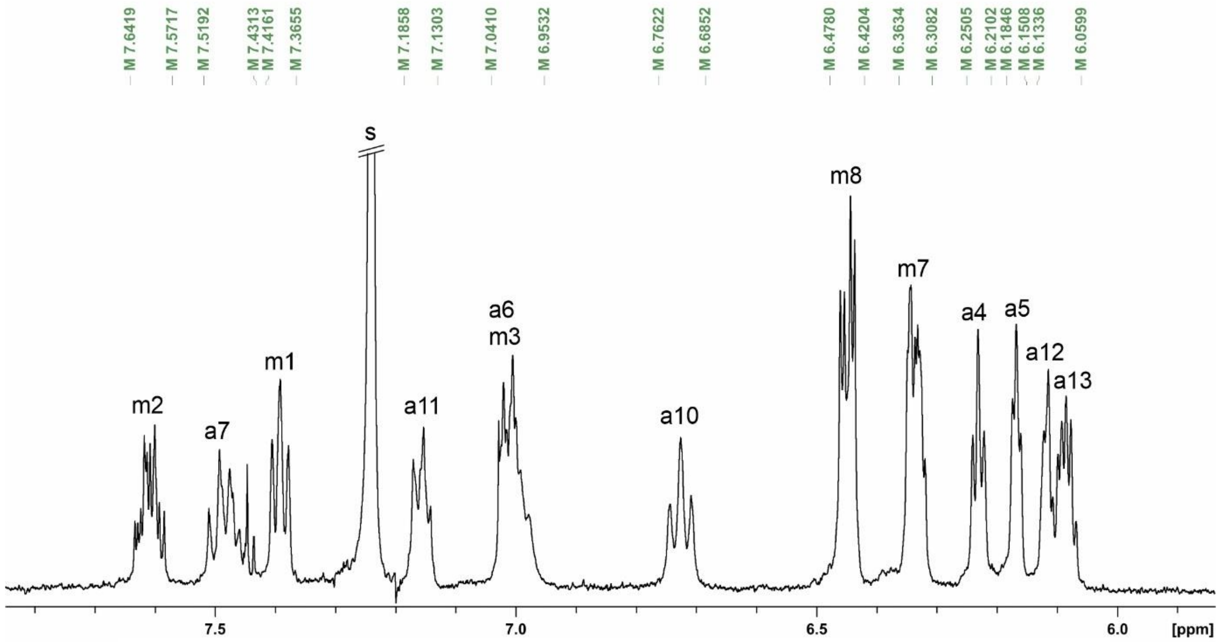

Figure S81. The ^{1}H NMR spectrum of **[4]cat^{7}** (500 MHz, $CDCl_3$, 300 K).

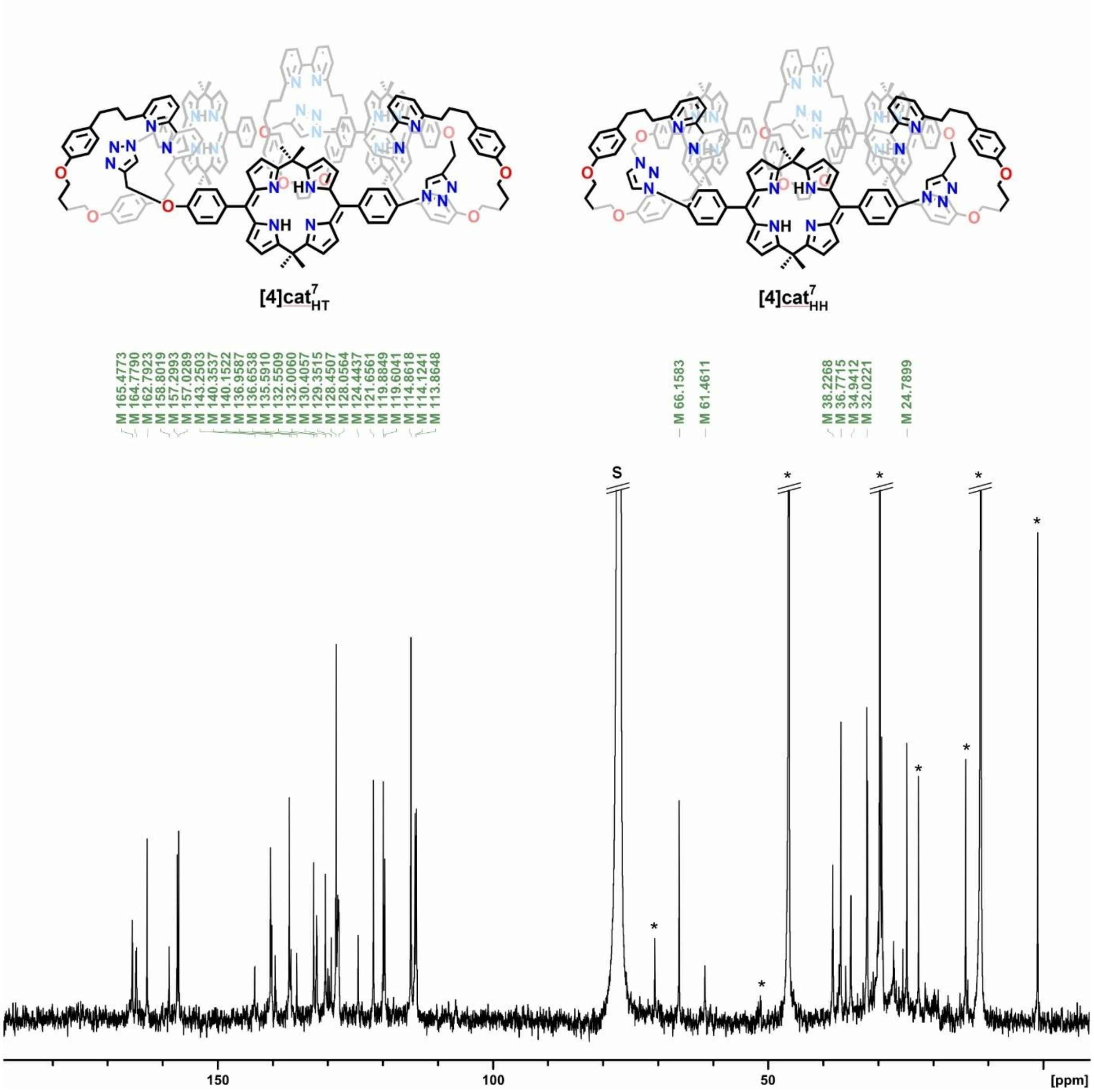

Figure S82. The ^{13}C NMR spectrum of **[4]cat^{7}** (150 MHz, $CDCl_3$, 300 K). Impurities (mainly TEA, MeOH and H grease) were labeled with asterisks.

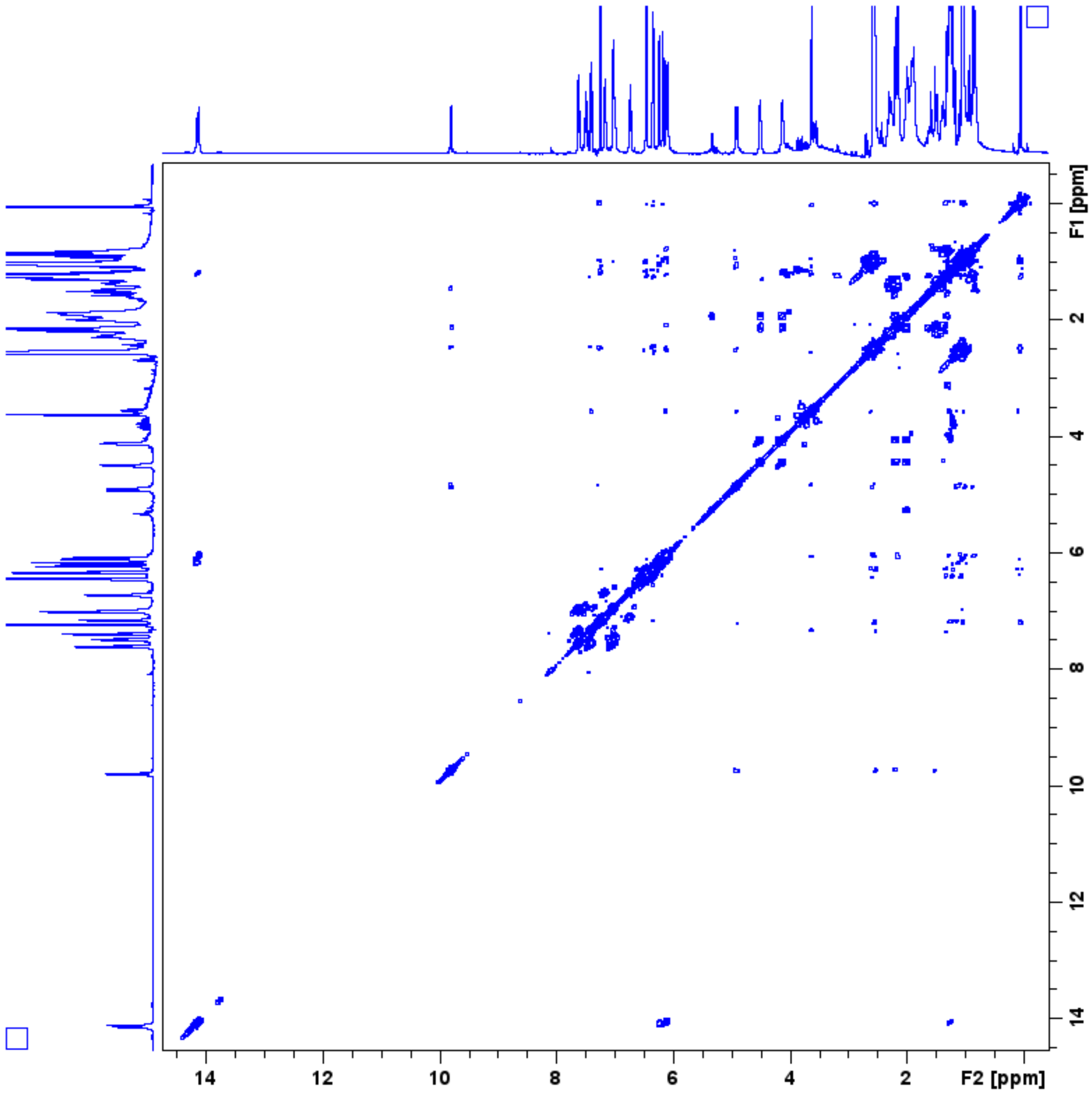

Figure S83. The ^{1}H-^{1}H COSY NMR spectrum of **[4]cat^{7}** (500 MHz, $CDCl_3$, 300 K).

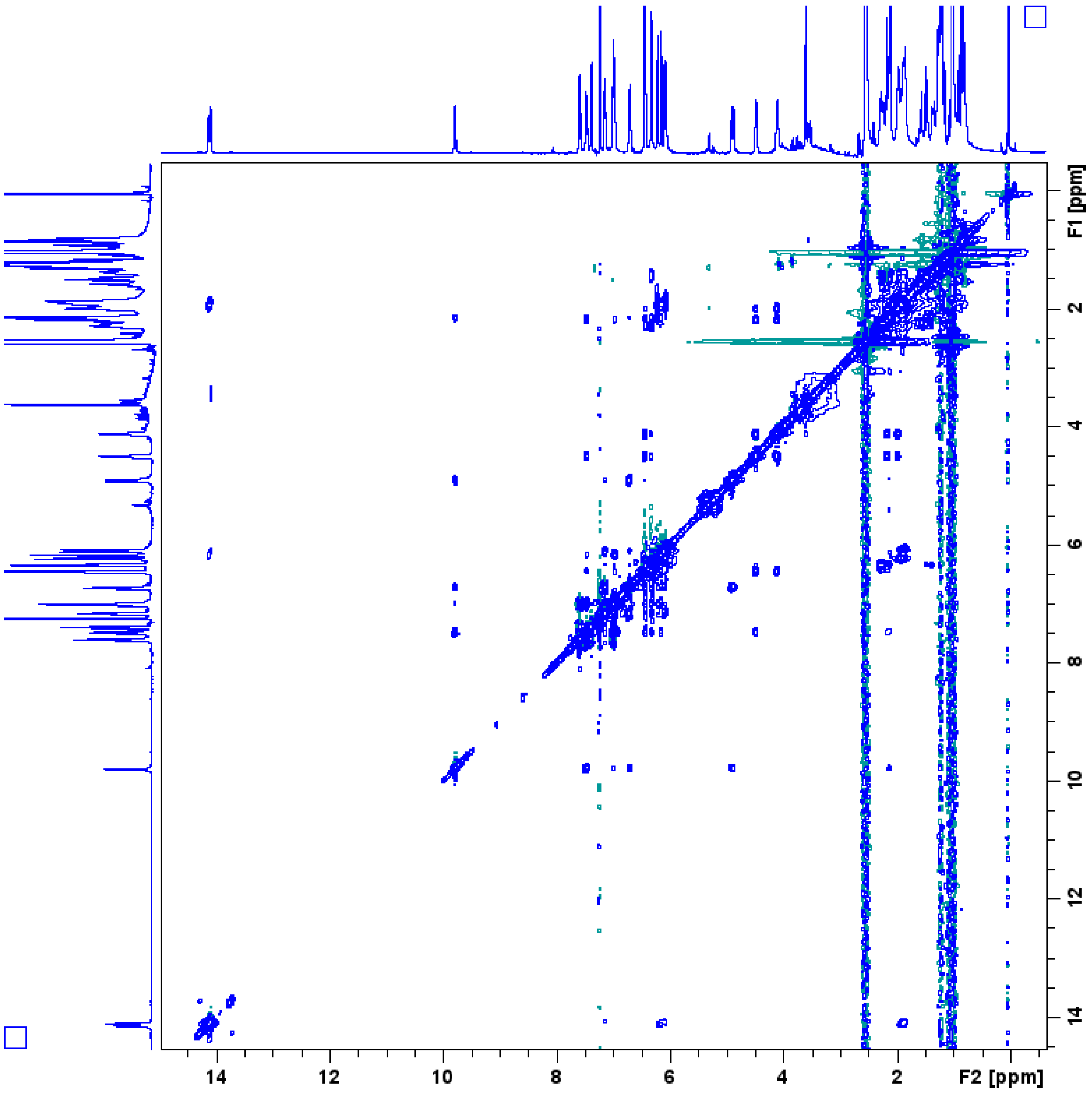

Figure S84. The ^{1}H-^{1}H NOESY NMR spectrum of **[4]cat^{7}** (500 MHz, $CDCl_3$, 300 K).

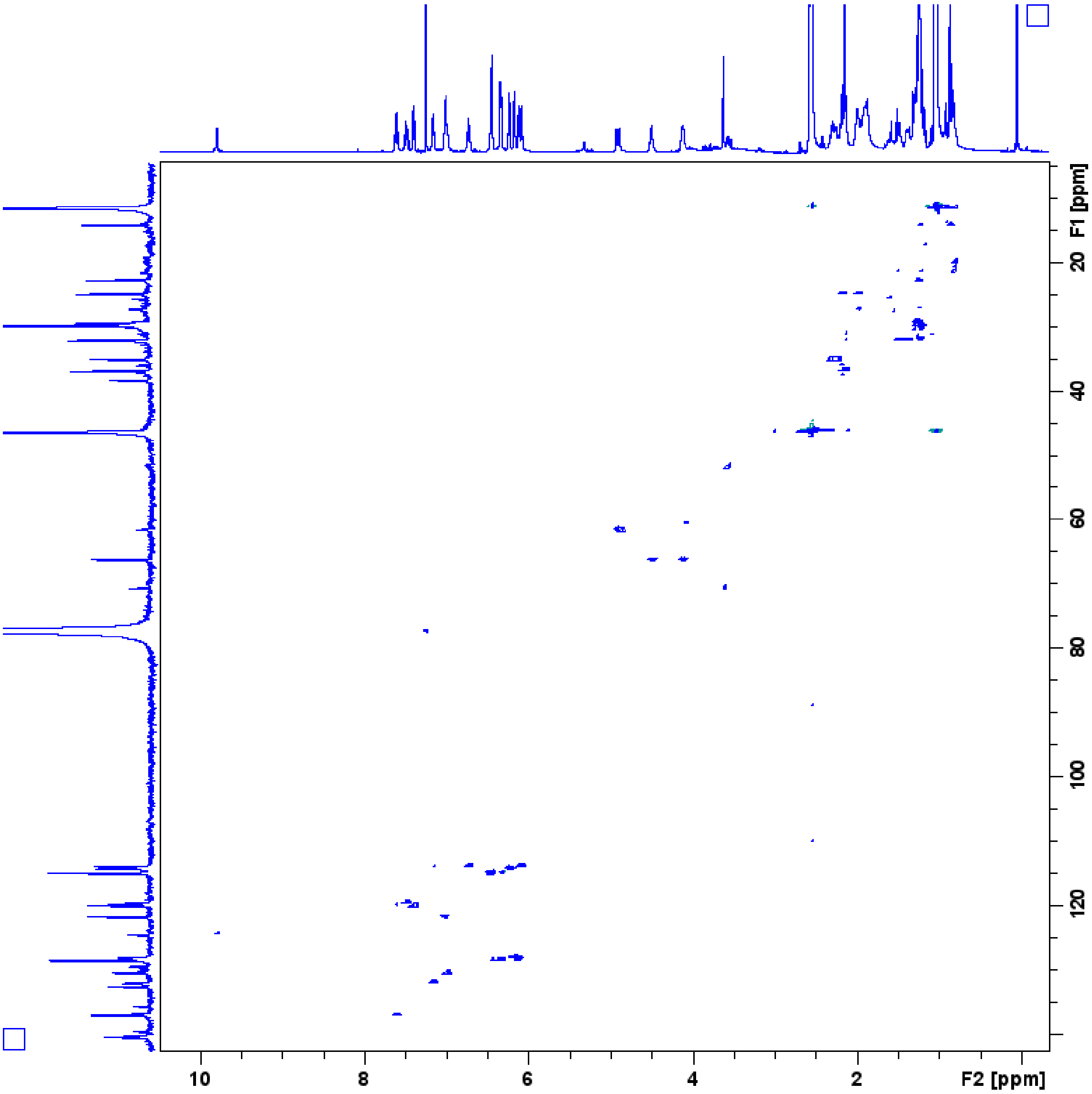

Figure S85. The ^{1}H-^{13}C HSQC NMR spectrum of **[4]cat^{7}** (500 MHz, $CDCl_3$, 300 K).

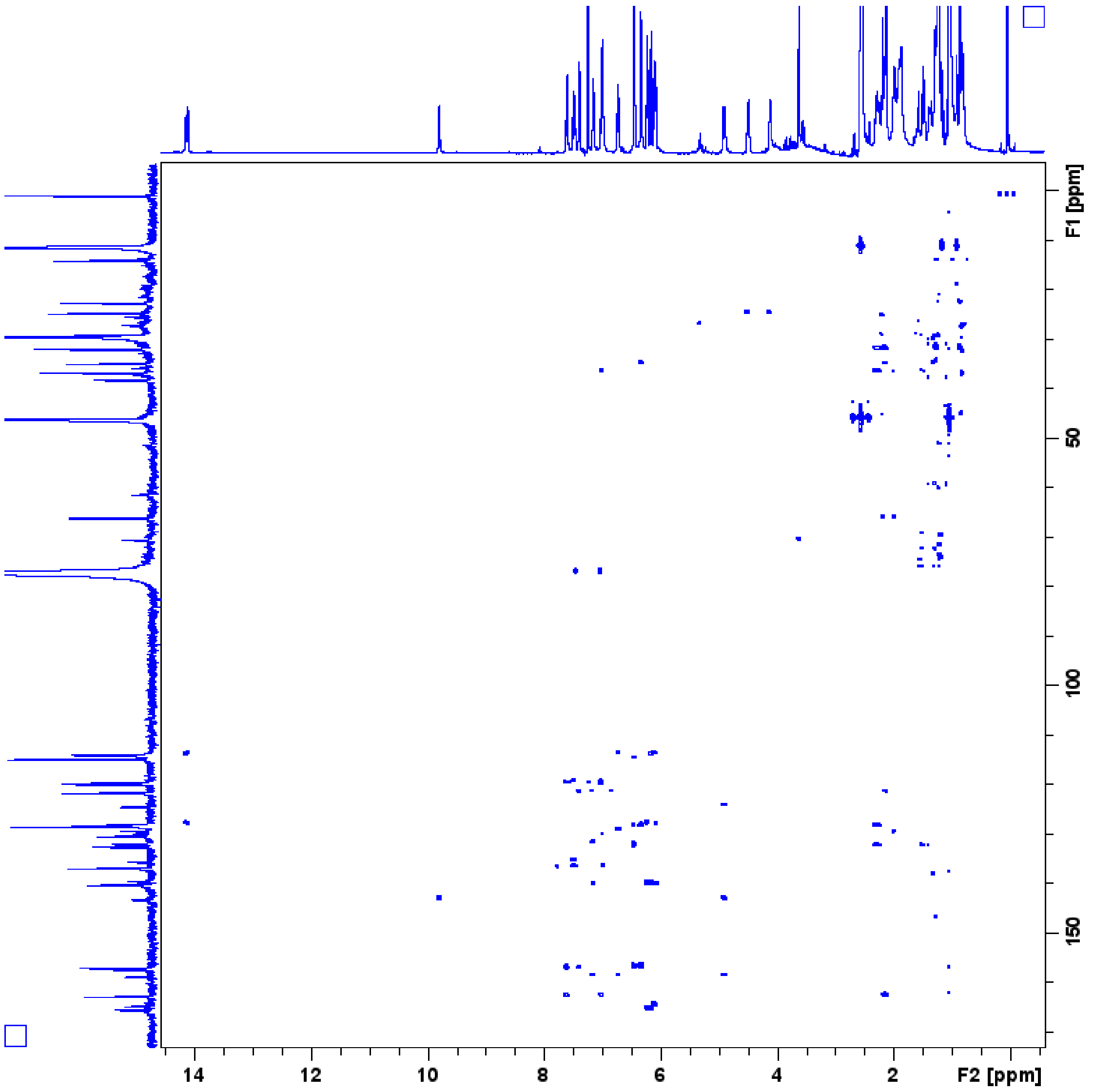

Figure S86. The ^{1}H-^{13}C HMBC NMR spectrum of **[4]cat^7** (500 MHz, $CDCl_3$, 300 K).

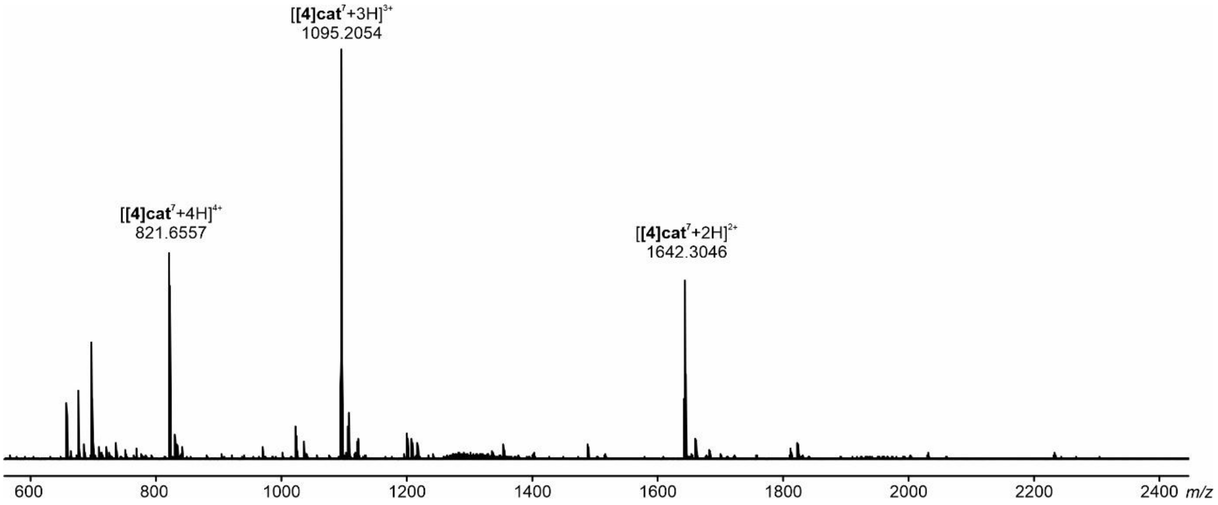

Figure S87. The ESI (TOF) mass spectrum of **[4]cat^7**.

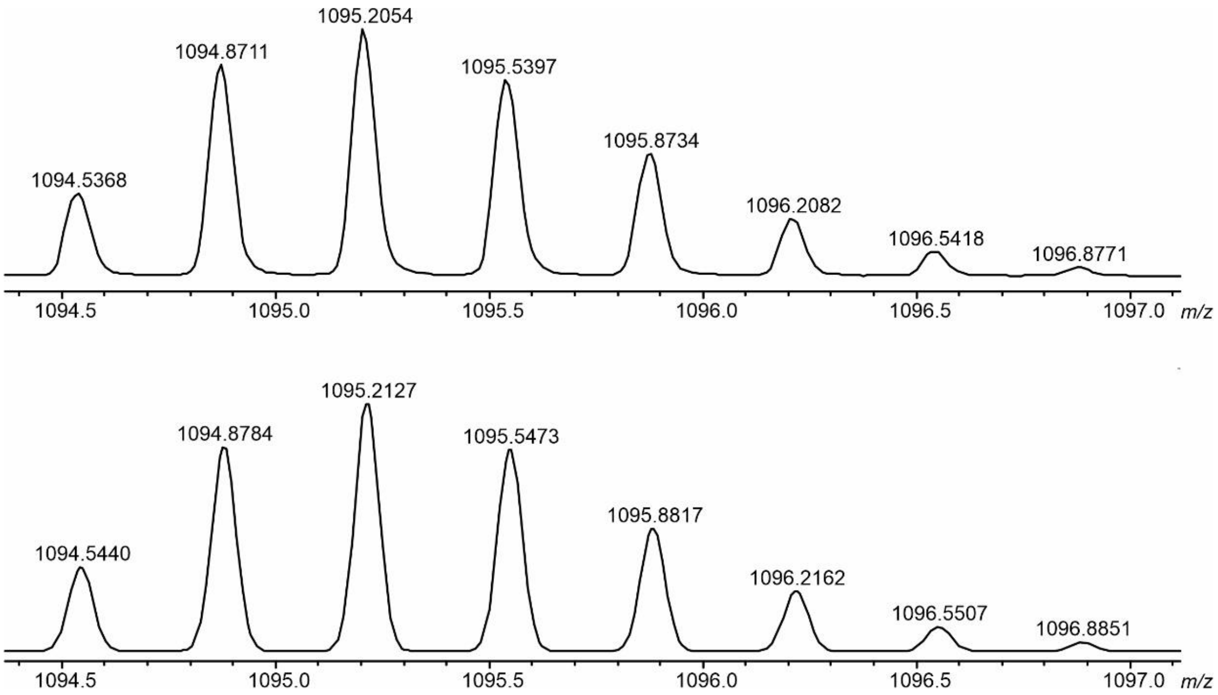

Figure S88. The ESI (TOF) mass spectrum of **[4]cat^{7}**. Top: experimental, bottom: simulated isotopic pattern.

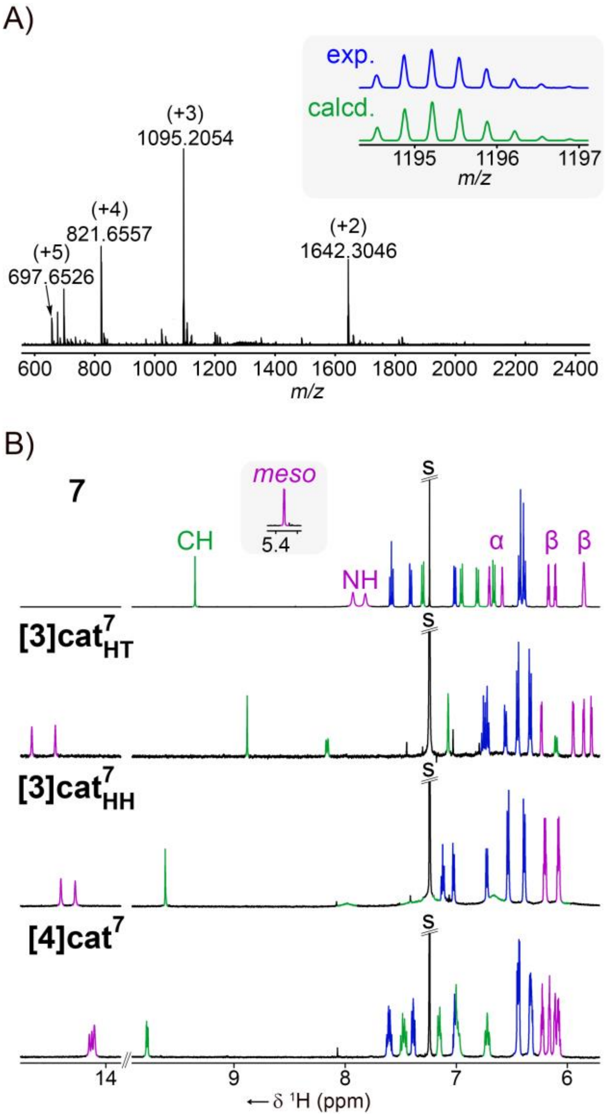

Figure S89. A) The ESI-MS spectrum of **[4]cat^{7}**. B) Parts of the ^{1}H NMR spectrum (500 MHz, 300 K, $CDCl_3$) of [2]rotaxane **7**, **[3]cat$^{7}_{HT}$**, **[3]cat$^{7}_{HH}$**, and a mixture incorporating **[4]cat^{7}**. The signals of the bipyridine macrocycle in blue, a thread in green and dipyrromethane/calix[4]phyrin in magenta.

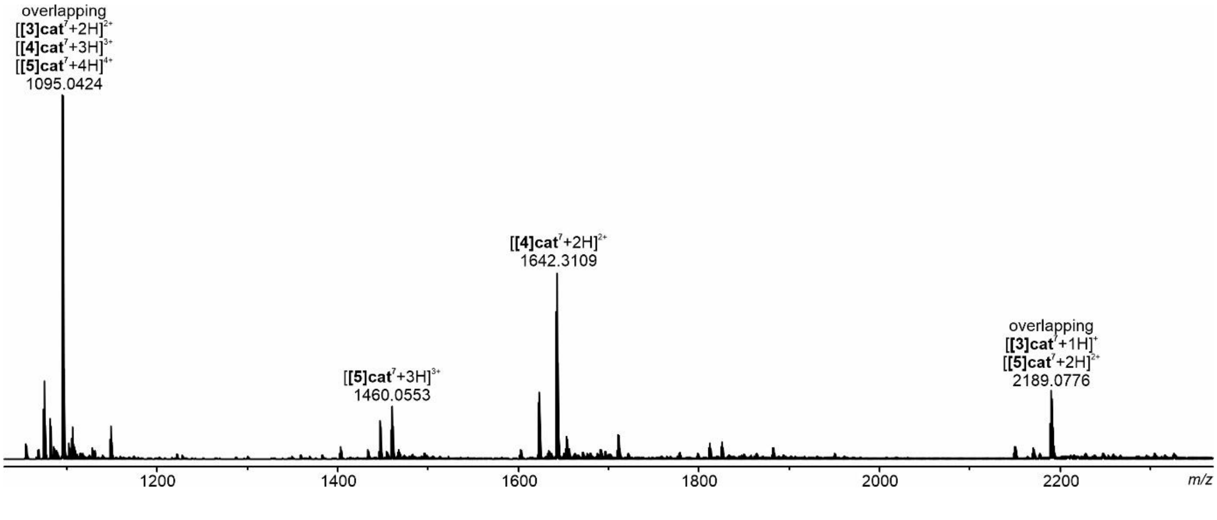

Figure S90. The ESI (TOF) mass spectrum of for mixture of catenanes after condensation of **7**.

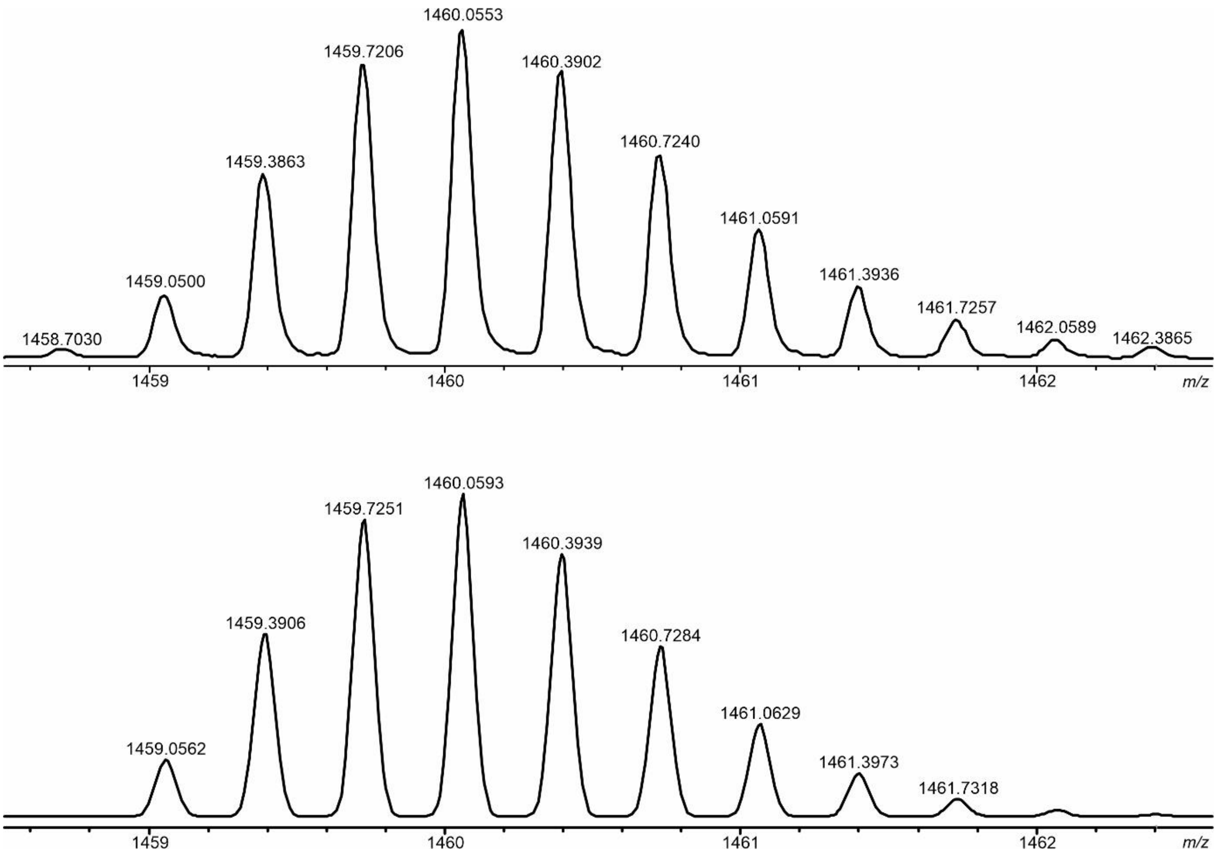

Figure S91. The ESI (TOF) mass spectrum of **[5]cat[7]**. Top: experimental, bottom: simulated isotopic pattern.

References

(1) Rigaku Oxford Diffraction, (2022), CrysAlisPro Software System, Version 1.171.42.74a, Rigaku Corporation.

(2) Sheldrick, G. M. *SHELXT* – Integrated Space-Group and Crystal-Structure Determination. *Acta Crystallogr A Found Adv* **2015**, *71* (1), 3–8.

(3) Sheldrick, G. M. Crystal Structure Refinement with *SHELXL*. *Acta Crystallogr C Struct. Chem.* **2015**, *71* (1), 3–8.

(4) Winn, J.; Pinczewska, A.; Goldup, S. M. Synthesis of a Rotaxane Cu^{I} Triazolide under Aqueous Conditions. *J. Am. Chem. Soc.* **2013**, *135* (36), 13318–13321.

(5) Lewis, J. E. M.; Bordoli, R. J.; Denis, M.; Fletcher, C. J.; Galli, M.; Neal, E. A.; Rochette, E. M.; Goldup, S. M. High Yielding Synthesis of 2,2′-Bipyridine Macrocycles, Versatile Intermediates in the Synthesis of Rotaxanes. *Chem. Sci.* **2016**, *7* (5), 3154–3161.

(6) Cao, S.; Xia, Y.; Shao, J.; Guo, B.; Dong, Y.; Pijpers, I. A. B.; Zhong, Z.; Meng, F.; Abdelmohsen, L. K. E. A.; Williams, D. S.; Van Hest, J. C. M. Biodegradable Polymersomes with Structure Inherent Fluorescence and Targeting Capacity for Enhanced Photo-Dynamic Therapy. *Angew. Chem. Int. Ed.* **2021**, *60* (32), 17629–17637.

(7) Grzelczak, R. A.; Basak, T.; Trzaskowski, B.; Kinzhybalo, V.; Szyszko, B. Multimodal Molecular Motion in the Rotaxanes and Catenanes Incorporating Flexible Calix[*n*]phyrin Stations. *Angew. Chem. Int. Ed.* **2025**, e202413579.

(8) Grimes, K.; Gupte, A.; Aldrich, C. Copper(II)-Catalyzed Conversion of Aryl/Heteroaryl Boronic Acids, Boronates, and Trifluoroborates into the Corresponding Azides: Substrate Scope and Limitations. *Synthesis* **2010**, *2010* (09), 1441–1448.

(9) D'Souza, F.; Smith, P. M.; Zandler, M. E.; McCarty, A. L.; Itou, M.; Araki, Y.; Ito, O. Energy Transfer Followed by Electron Transfer in a Supramolecular Triad Composed of Boron Dipyrrin, Zinc Porphyrin, and Fullerene: A Model for the Photosynthetic Antenna-Reaction Center Complex. *J. Am. Chem. Soc.* **2004**, *126* (25), 7898–7907.

(10) Xiong, R.; Andres, J.; Scheffler, K.; Borbas, K. E. Synthesis and Characterisation of Lanthanide-Hydroporphyrin Dyads. *Dalton Trans.* **2015**, *44* (6), 2541–2553.
